# The earliest unambiguous Neanderthal engravings on cave walls: La Roche-Cotard, Loire Valley, France

**DOI:** 10.1371/journal.pone.0286568

**Published:** 2023-06-21

**Authors:** Jean-Claude Marquet, Trine Holm Freiesleben, Kristina Jørkov Thomsen, Andrew Sean Murray, Morgane Calligaro, Jean-Jacques Macaire, Eric Robert, Michel Lorblanchet, Thierry Aubry, Grégory Bayle, Jean-Gabriel Bréhéret, Hubert Camus, Pascal Chareille, Yves Egels, Émilie Guillaud, Guillaume Guérin, Pascale Gautret, Morgane Liard, Magen O’Farrell, Jean-Baptiste Peyrouse, Edit Thamó-Bozsó, Pascal Verdin, Dorota Wojtczak, Christine Oberlin, Jacques Jaubert

**Affiliations:** 1 Unité mixte de recherche 7324, CItés, TERritoires, Environnement et Sociétés, Laboratoire Archéologie et Territoires, Université de Tours, Tours, France; 2 Equipe d’accueil 6293, GéoHydrosytèmes COntinentaux, Faculté des sciences et techniques, Université de Tours, Tours, France; 3 Department Physics, Technical University of Denmark, Roskilde, Denmark; 4 Nordic Laboratory for Luminescence Dating, Department of Geoscience, Aarhus University, Aarhus, Denmark; 5 Unité mixte de recherche Histoire naturelle de l’Homme préhistorique, Musée de l’Homme, Museum National d’Histoire Naturelle, Centre national de la Recherche Scientifique, Paris, France; 6 Centre national de la Recherche Scientifique, Paris, France; 7 Côa Parque, Fundação para a Salvaguarda e Valorização do Vale do Côa, Vila Nova de Foz Côa, Portugal; 8 Centro de Arqueologia Universidade de Lisboa, Facultade de Letras, Lisboa, Portugal; 9 Institut National de Recherches Archéologiques Préventives, Pantin, France; 10 PROTEE association, Villeneuve-les Maguelone, France; 11 Equipe d’accueil 6298, Centre Tourangeau d’Histoire et d’étude des Sources, Faculté des Arts et Sciences Humaines, Tours, France; 12 Ecole Nationale des Sciences Géographiques, Institut Géographique National, Marne la Vallée, France; 13 Unité mixte de recherche 7209, Archéozoologie, Archéobotanique: Sociétés, Pratiques et Environnements, Centre national de la Recherche Scientifique, Museum National d’Histoire Naturelle, Paris, France; 14 Unité mixte de recherche 6118, Géosciences Rennes, Université de Rennes, Centre national de la Recherche Scientifique, Rennes, France; 15 Unité mixte de recherche 7327, Institut des Sciences de la terre, Université d’Orléans, Centre national de la Recherche Scientifique, Bureau de Recherches Géologiques et Minières, Orléans, France; 16 Laboratoire de Géographie Physique et Environnementale, Université Clermont-Auvergne, Centre national de la Recherche Scientifique, Institut National de Recherches Archéologiques Préventives, Clermont-Ferrand, France; 17 Unité mixte de recherche 5199, De la Préhistoire à l’Actuel: Culture, Environnement et Anthropologie, Université de Bordeaux, GPR Hman Past, Pessac, France; 18 Unité mixte de recherche 7041, équipe Archéologies Environnementales, Archéologie et Sciences de l’Antiquité, Nanterre, France; 19 Mining and Geological Survey of Hungary, Budapest, Hungary; 20 Unité mixte de recherche 7264, Gestion des REssources Naturelles, Environnements et Sociétés, Cultures et Environnements: Préhistoire, Antiquité, Moyen-Age, Centre national de la Recherche Scientifique, Nice, France; 21 Institut National de Recherches Archéologiques Préventives, Nîmes, France; 22 Integrative Prehistory and Archaeological Science, University of Basel, Basel, Suisse; 23 Centre de Datation par le RadioCarbone, Unité mixte de recherche 5138 Archéologie et Archéométrie, Villeurbanne, France Centre national de la Recherche Scientifique, Université Claude Bernard Lyon 1, Université Lumière Lyon 2, Villeurbanne, France; Universita degli Studi di Ferrara, ITALY

## Abstract

Here we report on Neanderthal engravings on a cave wall at La Roche-Cotard (LRC) in central France, made more than 57±3 thousand years ago. Following human occupation, the cave was completely sealed by cold-period sediments, which prevented access until its discovery in the 19^th^ century and first excavation in the early 20^th^ century. The timing of the closure of the cave is based on 50 optically stimulated luminescence ages derived from sediment collected inside and from around the cave. The anthropogenic origin of the spatially-structured, non-figurative marks found within the cave is confirmed using taphonomic, traceological and experimental evidence. Cave closure occurred significantly before the regional arrival of *H*. *sapiens*, and all artefacts from within the cave are typical Mousterian lithics; in Western Europe these are uniquely attributed to *H*. *neanderthalensis*. We conclude that the LRC engravings are unambiguous examples of Neanderthal abstract design.

## Introduction

Since the 1980s, many discoveries have provided evidence of the diversity of Neanderthal behaviour. However, symbolic productions attributed to Neanderthals are few in number; these include, for instance, engravings on bones or pieces of rock [1], variably transformed shells [2, 3], and the possible use of feathers and raptor claws [4]. Use of pigments may also fall in this category, although pigments could have a utilitarian function [5–9]. Other activities are represented by examples with no apparent equivalent, such as a complex object made of stone and bone [10, 11], or the appropriation of the underground environment represented by architectural construction inside a cave. At Bruniquel cave (France), in a large chamber located more than 300 meters from the entrance, many stalagmites have been deliberately broken and placed on the ground to form a large oval structure associated with other smaller structures; traces of fire were found on these structures. U-Th dating gave an age of about 170 ka for this highly organized architectural structure [12]. Other indications of symbolic behaviour (including burials) are subject to debate [13–17]. Taken together, Neanderthal symbolic activities appear quite different from those of later periods [18].

Rare graphic traces, different from functional cut marks, have been observed on fragments of bones, rocks, speleothems or shells from other Middle Palaeolithic sites. An engraved giant deer phalanx has been found in Einhornhoehle (Germany) inside an archaeological level dated to >47 ka. This bone has different well-organised engravings on two sides, which are argued to be intentional [1]. At Krapina I (Croatia), eight eagle talons and one phalanx, associated with Mousterian tools, and Neanderthal bones were discovered [19, 20]. The eagle phalanx and four talons display cut marks, and the talons show burnt areas, residues of ochre, a fiber and polished zones possibly indicating an ornamental use [21]. Among the human bones, an incised frontal has 35 cut-marks (Krapina 3 frontal) [22]. At Fumane cave (Italy), in a level dated to 44.8–42.2 ka cal BP, bird bones display anthropogenic striae, long or short, and transversal deep traces were made on wing bones with flint tools, indicating the intentional removal of large feathers. The human modifications indicate an indisputable non-utilitarian use [4, 23]. In addition, an ochered fossil marine shell was found in a Mousterian layer at Fumane [3]. At the Zaskalnaya VI (Kolosovskaya Crimea) Neanderthal site, a group of parallel notches has been identified on the main axis of a raven bone. The Neanderthal intention appears to have been to produce equidistant notches, not only for utilitarian use but also for the creation of a code [24]. In Los Aviones Mousterian site (Cartagena, Spain) there are different types of potentially symbolic finds related to the use of marine shells: i) shells with umbo-perforated valves, and ii) shells with traces of haematite colourant inside the concavity of the valves [2, 25]. In Anton cave (Murcia, Spain), colourants have also been observed in shells [2, 26]. Quneitra (the Golan Heights) lies in a region where both human types (Neanderthal and anatomically modern human) coexisted; the cortical face of a piece of a Levallois flint core, 8 cm long, displays incised lines which are neither natural nor butchering marks. The engraving (dated to 50 ka BP) is composed of straight parallel lines and semi-circular concentric lines believed to be intentional engravings [27–29]. In Gorham’s cave (Gibraltar), a Mousterian level covered a bedrock surface containing deep and wide traces, probably made with a lithic tool, and created by repeated and careful grooving to give a geometric design. Utilitarian origin is excluded and the pattern is considered to attest to the Neanderthal capacity for abstraction [30]. In the Iberian sites of Ardales, Maltravieso and La Pasiega [31–33], part of the graphic productions (red marks on a stalagmitic dome, a negative hand, and a part of a rectangular sign) has been attributed to Neanderthals by U-Th dating of an overlying calcite crust [34–36]. However the assignment of a Neanderthal authorship is contentious and has raised significant debate in the scientific community [37–42]. Most of the discoveries identified above include objects discovered in archaeological layers which also provided elements of Mousterian lithic industries. Inevitably, these are most often dated by U-Th, thermoluminescence (TL) or optically stimulated luminescence (OSL). Radiocarbon dating is rarely an option, because of its limited age range. Finally, we draw attention to the observation that, among these examples, there is no evidence of graphic productions in series, or organized, on the walls of a cave or a shelter.

In 1846, La Roche-Cotard cave (LRC I) entrance was exposed during quarrying and in 1912, the site owner François d’Achon excavated almost all the inner sedimentary deposits. Only Mousterian lithic artefacts were discovered within the cave [43]; no later-period material was found. Subsequent excavation, in the 1970s [44] and from 2008 onwards, identified three additional loci close to the cave: LRC II (open air site at the foot of a cliff), LRC III (a small shelter) and LRC IV (a trench associated with a very small cave). Excavations of LRC II, III and IV all yielded evidence of Mousterian industries; LRC II also yielded a composite object (made of flint and bone) known as the “Mask of La Roche-Cotard” [10, 11].

On the walls of LRC I, the first observation of seemingly organized digital traces (finger-flutings) were made during field campaigns from 1976 to 1978, and then again from 2008 (all directed by the lead author). In addition, sparsely occurring red ochre spots were identified [45]. Other types of marks are also present: (i) traces left by animal claws, (ii) the smoothing of the very fragile wall surface presumably through repeated contact with animal fur, and (iii) numerous easily recognisable traces caused by the percussion of metal tools. These latter traces presumably result from the excavation in 1912. Cave visits were unusual until 1976, or from 1978 to 2008 (there is only one modern graffito, from 1992).

In the following, we use the term “engravings” for the finger-flutings, as an “engraving” is generally defined as the deliberate removal of material carried out with a tool or a finger. We will show that this removal of material is neither accidental nor utilitarian, but rather that it is intentional and meticulous. In 2008, the digital traces were recognized as ancient traces by M. Lorblanchet and subsequently by P. Paillet and E. Man-Estier (unpublished reports). A first description and survey of these numerous traces on the walls of the cave of La Roche-Cotard was made in 2013 [45] and since 2016, under the direction of E. Robert. The main objectives of this article are (i) to provide a detailed description of the traces, (ii) to prove their anthropic origin and (iii) to prove that they were made by Neanderthals, through an indirect absolute dating.

## Archaeological context

### Geological setting of the cave

**Fig 1:**
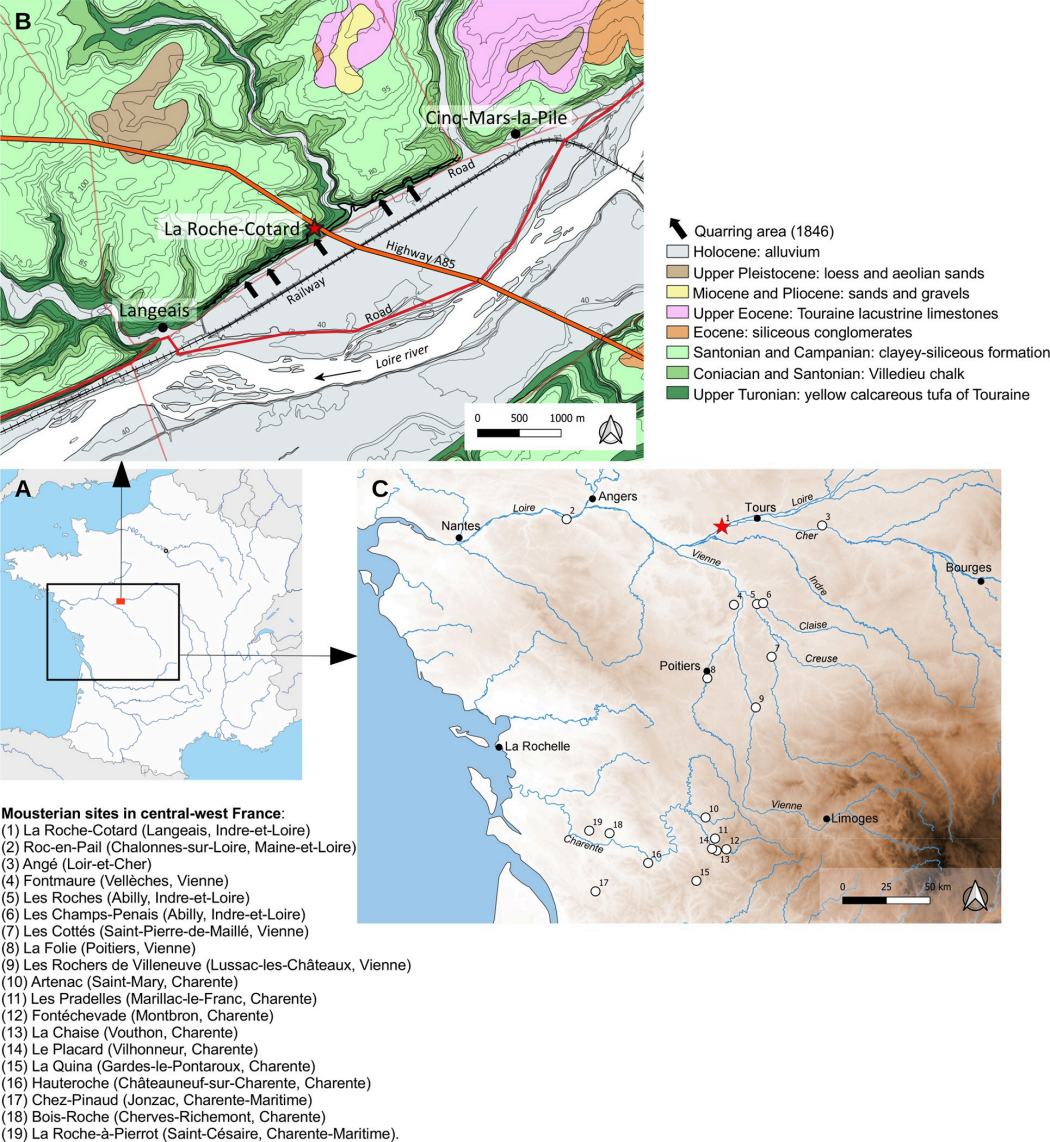
Location and map of La Roche-Cotard. A and B. Geographical and geological location of La Roche-Cotard. C. Map of the main Mousterian sites in central-west France. Sources: geological map redrawn from BRGM; coastlines, relief and rivers: Natural Earth (public domain); Map of France: reprinted from d_maps.com under a CC BY license, with permission from d_maps.com. Maps made with QGIS 3.4.12-Madeira (H. Guillemot).

**Fig 2:**
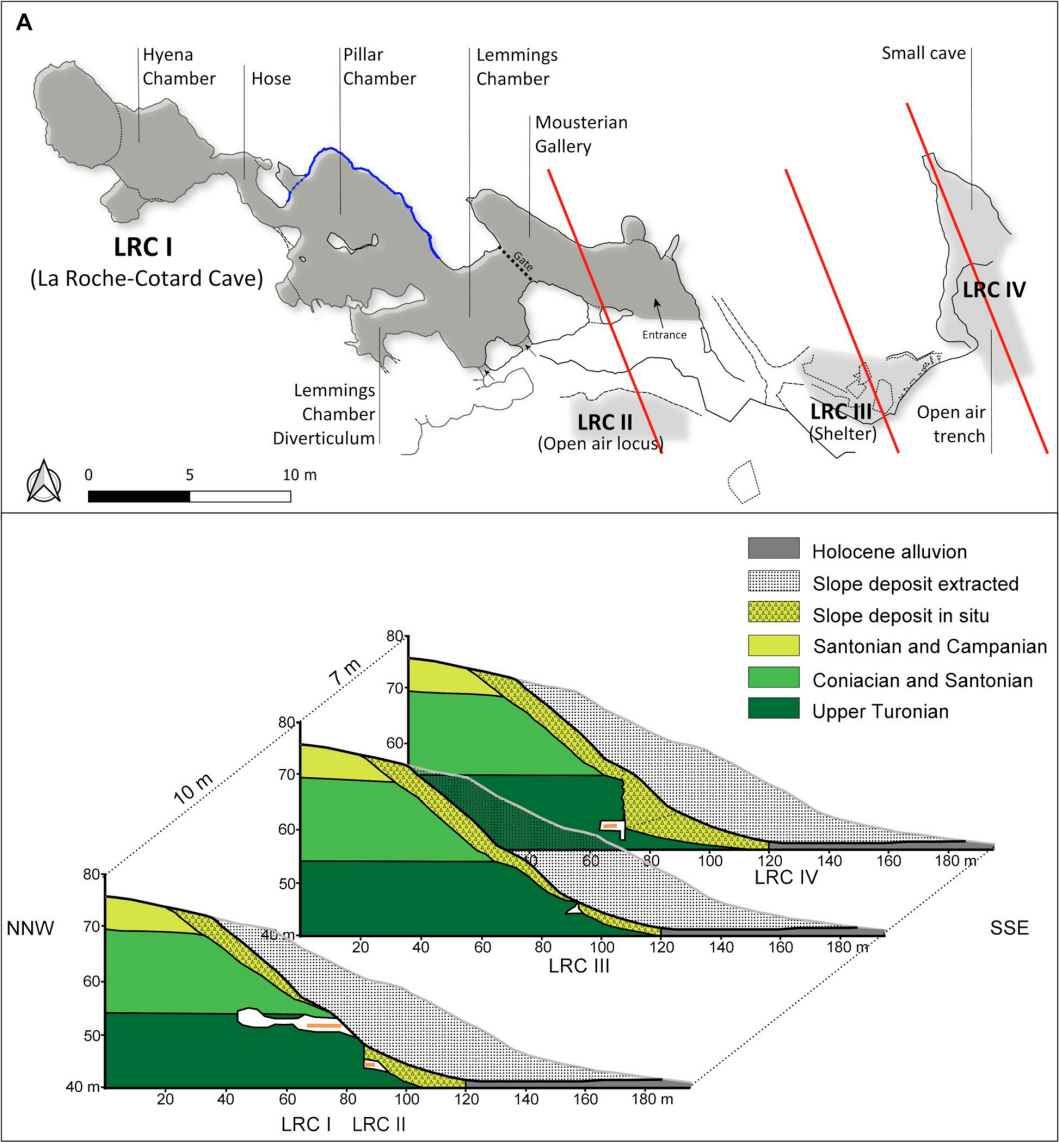
La Roche-Cotard site. A. Map of La Roche-Cotard with its four loci: LRC I, LRC II, LRC III and LRC IV. In blue: location of anthropogenic marks. B. Profiles of slope sections (red lines in A) with location of sediments extracted in 1846.

**Fig 3:**
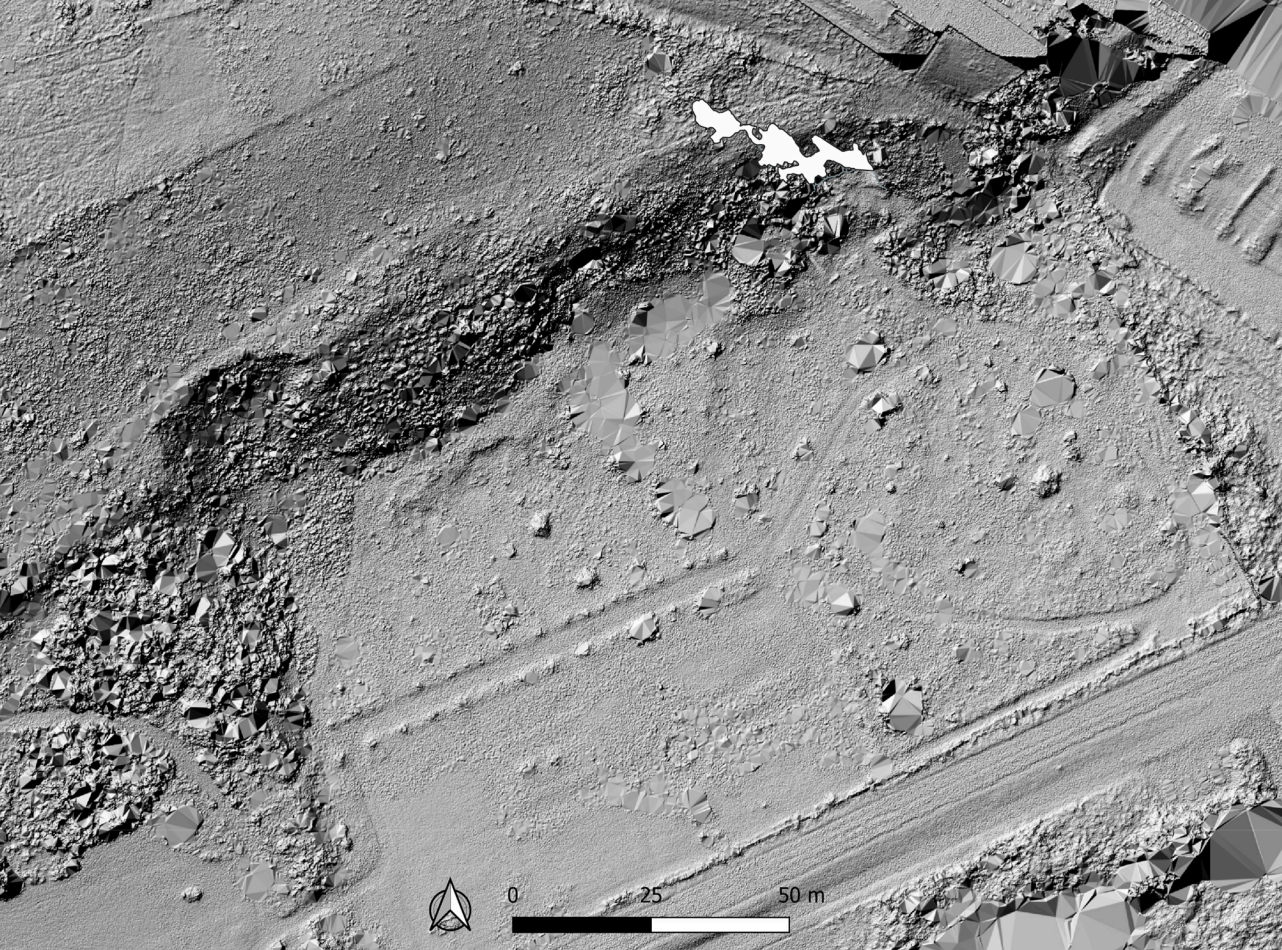
Lidar survey of the site of La Roche-Cotard. Orthophoto. The Lidar image shows the 1846 exploited zone and the cave (LRC I) in white. The cave entrance is in the abrupt northern slope left by workers in 1846. A Lidar drone survey was carried out on the area surrounding the cave using a Yellowscan Mapper, mounted on a Matrice 300 with three passes at a height of 35 m. These scans were then assembled using Yellowscan software. A Digital Terrain Model was generated at 10 cm resolution from these data, along with a hillshade version and contour lines. (Lidar ICONEM).

La Roche-Cotard is located in the Touraine region (47°20’13” N, 0°25’51” E), a plateau area reaching ~100 m altitude and now covered with wood and crop-land (Fig 1). The geological bedrock in and around the site is made up of Upper Cretaceous marine sediments. The down-cutting of what would become the Loire Valley started very early at the end of the Tertiary. At that time, the river abandoned its old course towards the north, and turned west, cutting through the plateau to a depth of approximately 50 m during the Plio-Quaternary. La Roche-Cotard, with its four different loci, lies on the north side of this fluvial valley. The Roche-Cotard cave (LRC I) was formed by karst processes in the Upper Turonian “Tuffeau jaune de Touraine” [46, 47], a yellow, sandy, more or less crumbly limestone that is usually poorly cemented (a biocalcarenite rich in detrital quartz). The plateau was later covered with a discontinuous, thin layer of silty sand (average thickness ~1 m), mainly accumulated as aeolian deposits during the last glacial [48]. Today bedrock is not often visible on the valley sides because it is covered by slope deposits formed by solifluction or runoff (bedrock is, however, exposed as a consequence of anthropogenic activities, such as extraction of tuff for construction purposes). The LRC I cave was flooded by the Loire River, very probably on numerous occasions, and these floodwaters contributed to its formation [45]. At the LRC site today, four loci (LRC I, LRC II, LRC III and LRC IV, Fig 2A) have been identified, all located inside the Turonian stage “Tuffeau jaune de Touraine”. In the past, all these loci were accessible to both animals and humans because the River Loire (then flowing close to the foot of the slope) removed all sediments coming from the plateau or brought by wind, and so prevented accumulation. When the river migrated from the foot of the slope, towards the other side of the river valley, gravity and wind began to accumulate sediment again; these new sedimentary deposits blocked access to the sites; some are still in place today, but the majority was extracted for the construction of the railway in the Loire valley in 1846 (Figs 2B and 3).

### Description of the LRC I cave

Today, the cave of La Roche-Cotard comprises four main chambers (Fig 2A) extending ESE-WNW for 33 m: the Mousterian Gallery, the Lemmings Chamber, the Pillar Chamber and the Hyena Chamber. In the back of the Hyena Chamber, collapse of the ceiling prevents the determination of the exact extent of the ancient cavity. The tuff in which the cave is carved displays highly silicified zones making up lenticular layers or slabs (thickness at a decimetric scale) and massive (at a meter scale) convoluted nodules. These two kinds of chert played an essential role in the formation and in the preservation of the cavity. These dense quartzitic sandstones are often exposed as relics as a result of erosion of the softer tuff during karstification. A continuous silicified horizon forming the cave ceiling is located below a hardground, known as the Langeais Hardground, which marks the boundary between the Turonian and the Coniacian [49].

**Fig 4:**
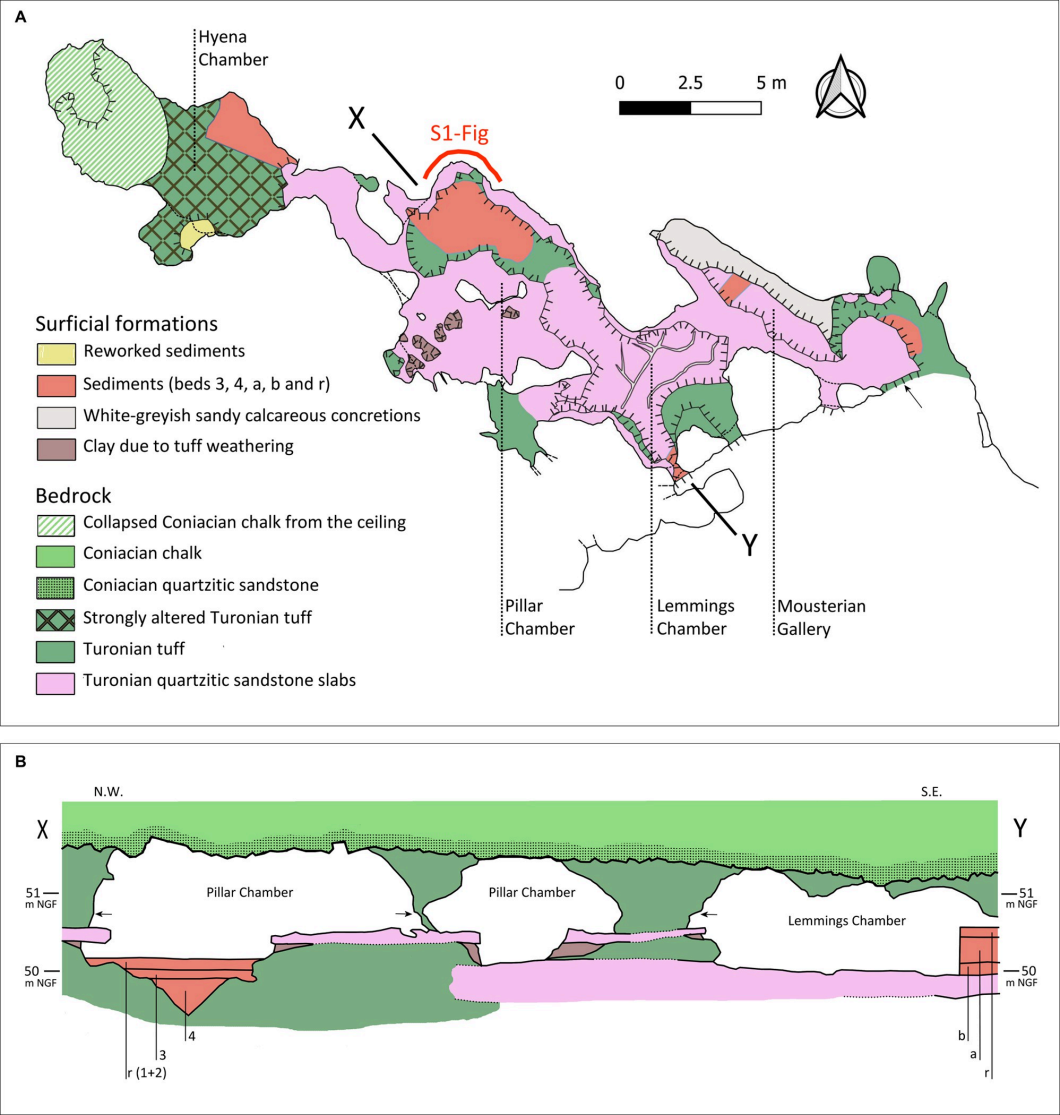
Description of La Roche-Cotard Cave (locus LRC I). A. Lithological map of the cave floor. B. XY section in the Pillar and Lemmings Chambers (location in A). The elevation of the ground surface increases steeply from the entrance to the Lemmings Chamber, and then only very slightly from SE to NW till the Hyena Chamber (1.5 m). 95% of the sediments that occupied a large part of the cave were removed during the 1912 excavation. Layers to the SE: Middle layer (b), Upper layer (a), Disturbed layer (r). Layers to the NW: Compact clayey layer with tuff gravel with bone fragments and coprolites (3). Sandy layer with soft reddish clay pebbles (4). Disturbed layers: (1), (2) and (r). The three arrows show the place of the overhang (50.75 m NGF, Nivellement Général de la France: general levelling of France) extending from the entrance of the cave to the Pillar Chamber.

The cave entrance opens into the Mousterian Gallery, formed within the Upper Turonian “Tuffeau jaune de Touraine” (Fig 4A). The floor of this gallery, at 49.2 m NGF (Nivellement Général de la France: general levelling of France), is mainly composed of quartzite sandstone, and the average elevation of the silicified biocalcarenite ceiling is 51 m NGF. To the west, a passage (width 2 m, height 1.5 m) is located above a 0.7 m thick quartzitic sandstone bed leading to the Lemmings Chamber. This chamber (average height: 1.7 m) includes a diverticulum to the west and, to the south-east, two narrow conduits opening to the outside. A large passage provides easy access to the Pillar Chamber to the north-west. This chamber has a central pillar and includes a 10 to 20 cm thick, continuous quartzite sandstone slab (at an elevation of 50.6 m NGF) several decimeters above the quartzite sandstone floor. This slab is extensively broken in the northern part of the chamber, but it reappears at the base of the north wall. In this area, the tuff outcropping at ground level is partially covered with post-karstification sediment. At the end of the northern part of the Pillar Chamber, there is a very compact stratified layer, 7 cm thick, whose top is at 50.00 m NGF. The Hyena Chamber is then reached via a narrow, sinuous passage with a quartzite sandstone slab floor.

The cross-section of the Pillar Chamber and Lemmings Chamber (Fig 4B) shows the geological structure of the main part of the cave. The siliceous bodies, i.e., the silicified hardground below the Coniacian chalk and the Turonian quartzite sandstone slabs, form the ceiling and floor of these chambers, respectively. The walls are composed of tuff and partly covered with a thin alteration film: the geomorphological characteristics of the walls can be seen in the photograph of the north wall in the Pillar Chamber (S1 Fig). A clear indentation in the walls occurs at 50.75 m NGF (see arrows in Fig 4B and 7 in S1 Fig). This overhang was eroded by the prolonged presence of ponded water at a more or less constant elevation defined by a sill at the entrance or in front of the entrance to the cave. Most of the sediment and alterites in these two chambers were removed during excavations in 1912. No speleothems developed in the cave.

## Sediment stratigraphy

### Archaeological excavations

From 2008, methodical excavation took place (i) in the cave (LRC I) [43–45], (ii) in front of and below the cave entrance (LRC II), (iii) in the small shelter discovered 10 meters away from the cave to the east (LRC III) and (iv) still further east (LRC IV), in a deep trench next to the tuff escarpment. At LRC IV, the very thick colluvial deposits covering the bedrock were not completely removed in 1846 [50] (in contrast to those in front of and above LRC I, II and III). Fig 2B shows the various altitudes of the excavated loci and the likely thickness of the deposits that covered the slope before the sediment extraction in 1846. The excavation of locus IV allowed the study of an 11 m thick section of these deposits that completely covered the slope prior to 19^th^ century extractions.

The rich faunal remains of large, medium and small vertebrates found in several layers were systematically studied [44, 51]. The palynological analyses were not successful because of the complete oxidation of spores and pollen grains due to the porous sediment and oxygenated water. For both large faunal remains (more than 2 cm in length) and all lithic remains, the coordinates were systematically recorded using a tacheometer. Smaller faunal and lithic remains (less than 2 cm) from each 50 x 50 x 5 cm sediment volume were recovered by water sieving using a 1 mm mesh sieve and preserved for study.

#### Deposits in the cave (LRC I)

Although F. d’Achon excavated most of the internal sedimentary deposits in 1912, some remained to be identified in the 1970s [44] (Fig 4B). A small sedimentary sequence in the Pillar Chamber includes, from bottom to top, (i) a sandy layer with soft reddish clay pebbles filling a small natural funnel formed in the tuff by water (layer 4), (ii) a compact clay layer with tuff gravel, bone fragments, coprolites (layer 3), and (iii) two modern, disturbed layers (layer 2 and 1). These layers cannot be assigned with certainty to the major sedimentary units identified below. Most of the sediments excavated by d’Achon were found in the Mousterian Gallery, the Lemmings Chamber and its diverticulum. In 1976, a study of the preserved sediments made it possible to distinguish three sedimentary layers: (i) a very sandy Lower layer, (ii) a Middle layer consisting primarily of silt from overflowing of the Loire, and (iii) an Upper layer formed of gelifracts and aeolian sand (Fig 4B). These three layers are also present in front of the cave entrance.

#### Lithostratigraphy, geometric distribution of the superficial deposits outside the cave

**Fig 5:**
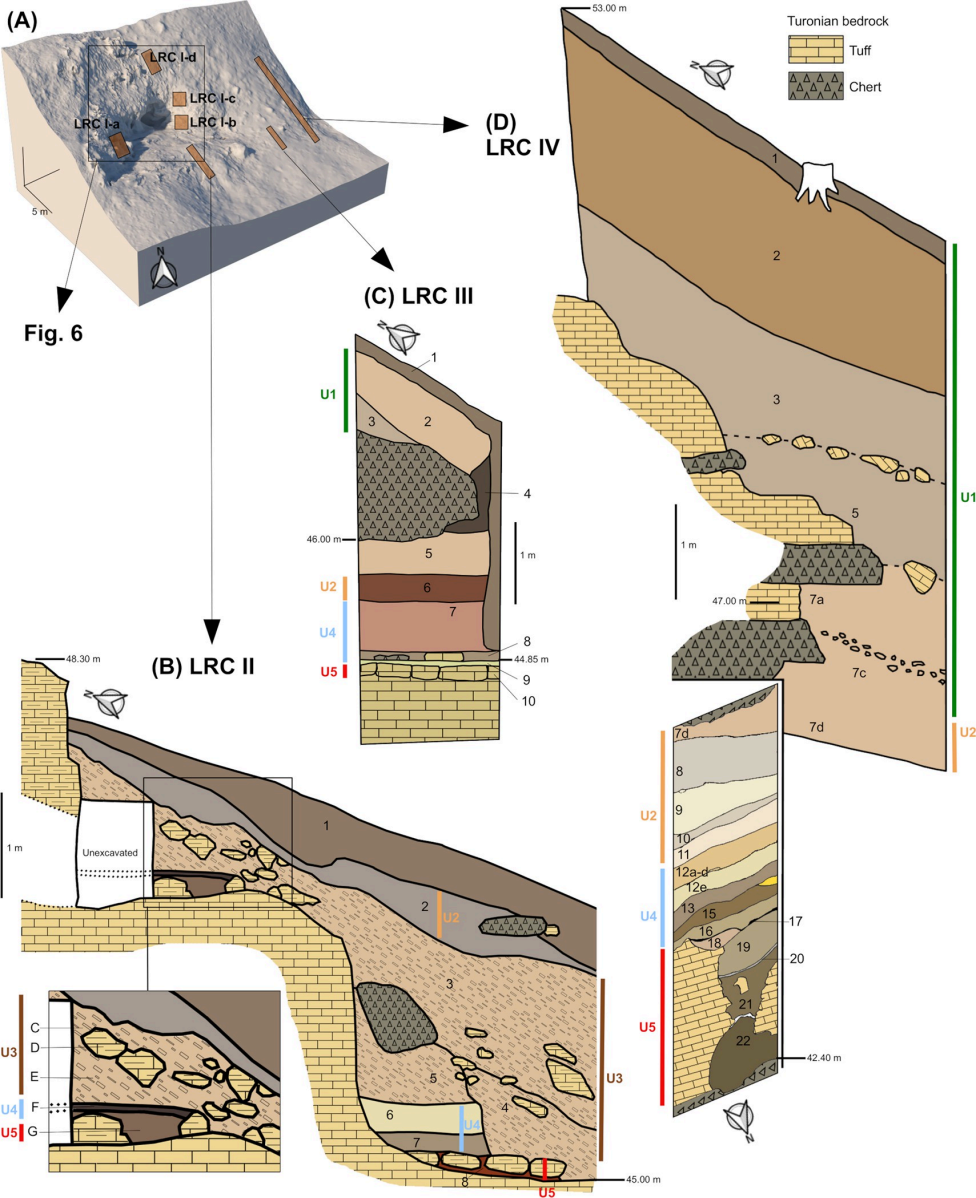
Lithostratigraphy and geometric distribution of the superficial deposits outside the cave. (A). Block diagram with loci positions and in particular the sub-loci LRC I-a to d. The stratigraphy of the layers intersected by LRC II (B), LRC III (C) and LRC IV (D). For each locus, the stratigraphic units (U5/red, U4/blue, U3/brown, U2/orange, U1/green) and their vertical extension is indicated. Each unit comprises several layers.

The deposits in loci LRC I-IV were grouped into five sedimentary units (U5 to U1, from oldest to youngest) based on their sedimentological characteristics and stratigraphic positions (Fig 5).

U5 is mainly composed of tuff blocks separated by voids filled with sandy reddish clay. In places, it also consists of brown to greenish-yellow, fine silty sand with largely subordinate clay. The mineralogical composition of this fine material (quartz, glauconite, cristobalite-tridymite opal and smectites) shows that it originates from the weathering (decarbonation) of the “tuffeau jaune” in a karst context [49]. This material is autochthonous, and sometimes locally reworked (bedded: LRC IV, layers 20 to 17).

U4 consists of sandy to silty brown to greyish layers, which are only slightly or not at all carbonated. The sand, essentially composed of quartz and up to 14% feldspars, also contains muscovite, biotite and pyroxenes. This mineralogical composition and the regular planar lamination indicate low to middle energy deposits from the Loire River [52, 53].

U3 consists of a light brown carbonated matrix (more than 20% CaCO_3_), with abundant frost-fractured quartzitic sandstone slabs and a few sandy blocks. The matrix is dominated by quartz-feldspar sand (layers 5 and 4) or very carbonated quartzose sand (layer 3). Frost-fractured slabs dip 20–25° to the south. This unit was originated by carbonated and siliceous (quartzitic sandstones) bedrock combined with aeolian sand blown from the Loire River alluvial plain (as testified by the presence of feldspar). These materials moved on the valley slope by solifluction and run-off in cold climatic conditions.

U2 is brown and texturally selected. It is made up of slightly carbonated fine-grained sand and silt, and its upper limit is tilted toward the south. The quartz-feldspar composition, lack of coarse sand and gravels and the rounded and matte surfaces of quartzitic sand-grains indicate aeolian transport from the Loire alluvial plain during a very cold and dry period [48] with little or no reworking by slope processes.

U1 was presumably extended everywhere on the slope before 1846. It consists of brown to greyish, very heterometric sedimentary layers composed of a dominant silty sand matrix with variable abundance of flint and limestone fragments. The limits between the layers dip 25–30° to the south, conforming to the topographic slope. These features indicate formation by gravitational processes (solifluction or runoff depending on the paleoenvironmental context) with reworked elements of the Cretaceous bedrock and aeolian inputs.

#### Sedimentary sequence (LRC II, III and IV)

The LRC II locus is located a few meters below the cave entrance (elevation 45 to 48 m). It yielded a 3 m thick sequence truncated by the 1846 quarring and lies against two natural “steps” carved by the Loire River into the Turonian tuff (Fig 5B). The sequence consists of eight layers grouped into four units. The weathered U5 (layer 8) is covered by the fluvial U4 at two elevations 2.3 m apart: layers 7 and 6 to the south, and layers G and F to the north. U3, originating with slope processes, comprises layers 5, 4 and 3. Layers 5 and 4 are separated by an erosive surface, possibly due to the Loire flooding. U3 is covered by U2 aeolian deposits (layer 2) that were partially preserved in 1846 at the bottom of the quarry pit of U1, which is now absent. Layer 1 was recently disturbed, perhaps as a result of d’Achon’s excavations and present pedogenesis.

The LRC III locus reveals ten layers grouped into four units (Fig 5C). U5 (layer 10), U4 (layers 9, 8, and 7), and U2 (layer 6) are preserved inside a small rock shelter carved by the Loire River into the yellow Turonian tuff, at between 44.83 and 46 m elevation. The chert base of the roof of this shelter has a very steep slope towards the north. U3 is absent at this locus. The upper gravity deposits (unit 1, layers 3 and 2), fill the front of the shelter. As in LRC II, the very thin layer 1 has been disturbed by pedogenesis after 1846.

The LRC IV locus contains 22 layers grouped into four units (Fig 5D). As in LRC III (Fig 5C), the lower units (i.e., U5: layers 22 to 18, U4: layers 17 to 12 and U2: layers 11 to 7d) are preserved inside the small cave, up to an elevation of 46.2 m (lower section, oriented E-W). U3 is absent and the ~5 m thick upper unit, U1 (layers 7c to 1) fills the front of the shelter (upper section, oriented N-S). Above the small cave, the elevation of the top of the sedimentary sequence is 53 m, despite being partially truncated by the 1846 extractions. It is thus higher than the ceiling of the LRC I cave (51 m) [50].

#### Residual undisturbed deposits observed inside the cave (LRC I) and around the entrance

**Fig 6:**
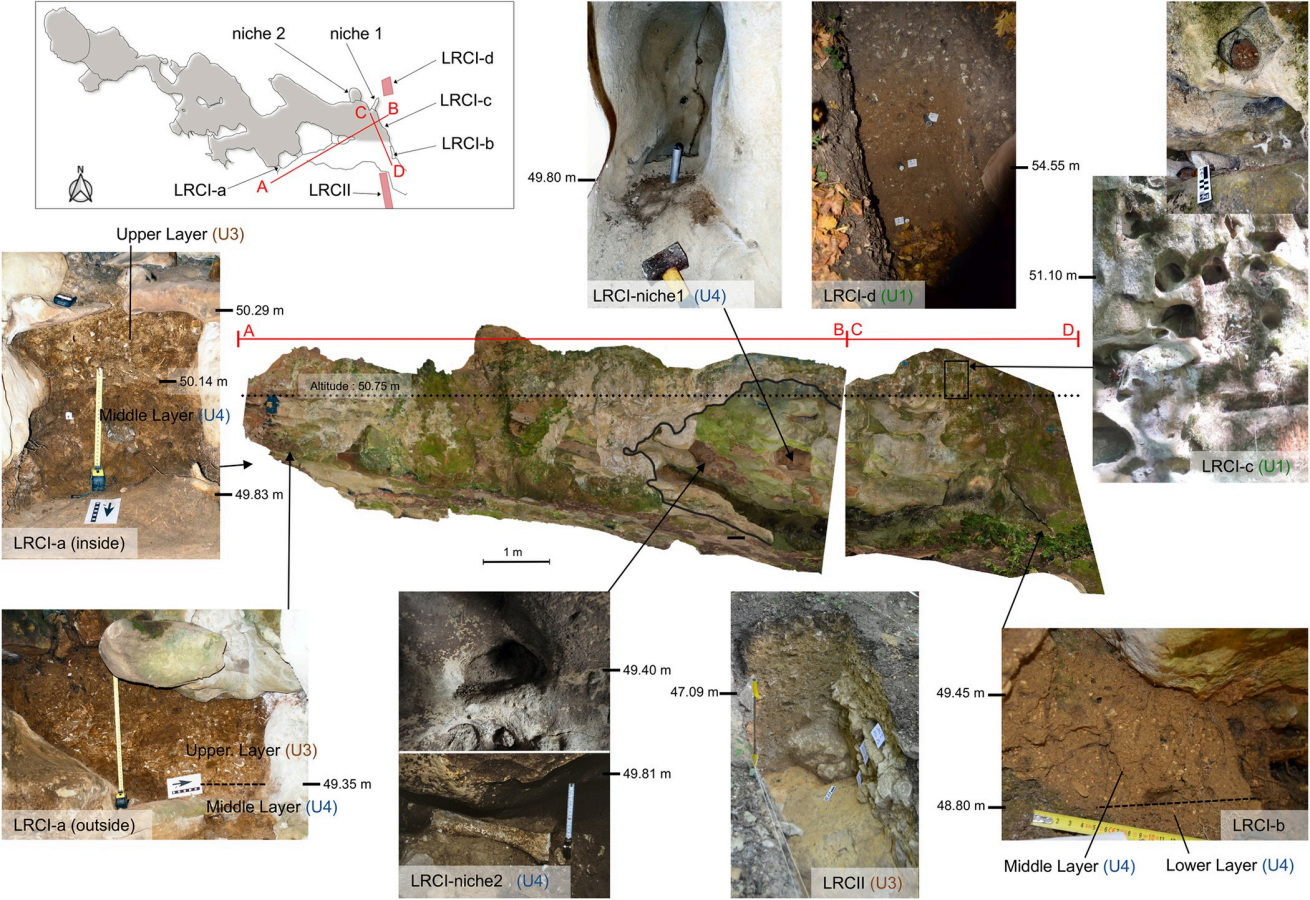
Location of undisturbed deposits near the LRC I cave entrance. The map locates the two orthophotos in the centre of the figure. The entrance of the cave, on the left orthophoto, is underlined in black. The dashed horizontal line corresponds to the altitude of the overhang to the pillar room as well as to the lower limit of the ceiling of the cave entrance. Below, the LRC II photograph shows only the upper part of the stratigraphy of this locus (Fig 5B), marking the period when sediments began to deposit on the slope. On the left, LRCI-a (Fig 5A) shows the middle and upper layers inside and outside the cave. Bottom right, LRCI-b (Fig 5A) also illustrates the same middle layer as found in LRCI-niches 1 and 2, but inside the cave entrance. LRCI-c (Fig 5A) shows the location of sediment remnants trapped in ancient and small galleries created by erosion in the hard cretaceous stone, belonging to the deposit which completed the sealing of the entrance. LRCI-d shows sediments very similar to those of LRCI-c (Fig 5A) which continued to accumulate for some time after the cave was closed. Altitudes are given to clarify the location of these different sections. The lower view of LRC I-niche 2 is from 1975 (Photogrammetry Iconem).

The removal/quarring of sediment in 1846 and the excavation of the cave in 1912 removed a large fraction of the deposits in and around the cave, but remnants are still in situ. Fig 6 gives an overview of the locations of these different strata; from this it is possible to reconstruct the stratigraphy of the deposits before the extractions in 1846. To the west, the conduit (0.60 m wide) connecting the Lemmings Chamber with the outside, contains an undisturbed section (LRC I-a in Fig 6) composed of two layers (the Middle layer, belonging to U4, and the Upper layer, belonging to U3; see Fig 6), accessible from both the inside and the outside of the cave. To the east, in the cave entrance, two niches (niches 1 and 2 in Fig 6) contain intact sediment identified as belonging to U4 (Middle layer). No stratigraphically continuous connection with the sediments outside the cave is available, but the same U4 sediment is protected in situ under a large quartzite sandstone slab in front of the entrance (LRC I-b in Fig 6). Higher up, about ten tunnels (LRC I-c in Fig 6) were filled with sediment from above the cave (i.e., corresponding to U1). Above the entrance of the cave, a test pit (LRC I-d in Fig 6) exposed a colluvium originated from sedimentary unit U1 (elevation 55.2 m to 57 m). In front of, and below the cave entrance, a trench (LRC II, Fig 5B) revealed an important stratigraphic sequence unaffected by the quarring in 1846.

#### Implications regarding sealing of the LRC I cave

**Fig 7:**
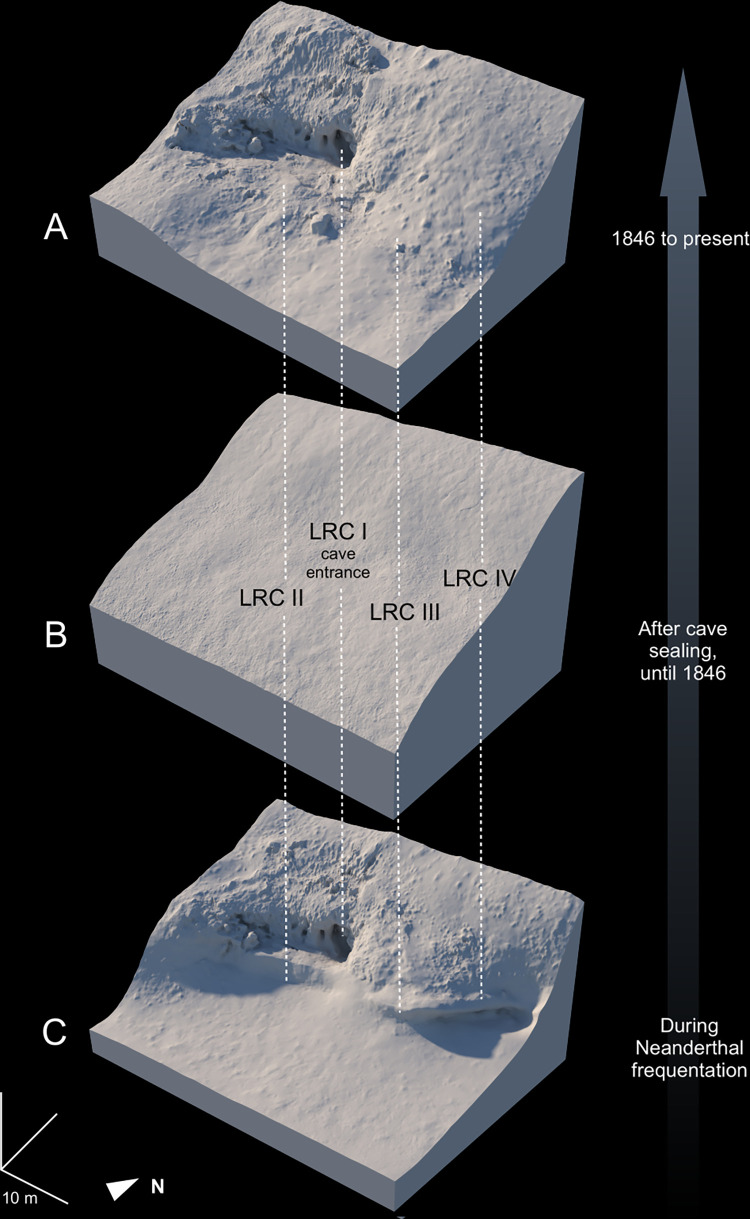
Reconstruction by 3D rendering of the slope of the site at different time periods. A: the diagram shows the state from 1846 till today (photogrammetry based on drone images); B: the slope before quarring in 1846; C: the site during the Neanderthal frequentations. Excavations (at LRC I, LRC II, LRC III and LRC IV) always showed sediments in direct contact with the tuff. The entrance to the cave was probably the same as today. At LRC II, the human settlement was located on the surface of a small bank of the Loire, a few meters below the entrance to LRC I. The cherty roofs of LRC III and IV are continuous, and the presence of anthropic layers makes it possible to trace the shape of the wall when these four spaces were occupied, but it is not possible to know in what chronological order the occupations took place. The Loire was then found close to the foot of the slope and carried downstream the sediments arriving from the plateau during a long period (Drone photos by J.-P. Corbellini from MSH Val de Loire. 3D model by N. Nony).

Although a large quantity of material was lost due to the excavation in 1846, the lithostratigraphic data described above make it possible to show that the entrance of the cave was gradually closed by sediment deposition. From a consideration of the LRC I (LRC I-a to -d) and LRC II sites, we deduce that:

–Some time after cave formation, the Loire River entered the cave during floods. The alluvial U4 unit deposited directly on the floor of the cave (LRC I) reduced the height of the passage to 60 cm. Remnants of this U4 unit are still to be found at the entrance of the cave (LRC I-a and LRC I-b).
–From the inclination and the elevation of the top of U3 at LRC II, it can be supposed that this unit could have been deposited at the front of the cave (floor: 48.75 m; ceiling: 51.65 m), at an elevation of about 50.3–50.5 m, partially closing the 60 cm high entrance.
–Above the fine-grained sediment of U3, the colluvial deposits of U2 and the pebble-rich deposits of U1 accumulated, in association with small gelifracts from the cave ceiling. The preservation of this material at altitudes of up to 56 m (at LRC I-c and LRC I-d) confirms that this unit extended across the entire slope and completely blocked the cave entrance. This material was preferentially extracted for the construction of the embankment in 1846, exposing the cave entrance. The scars of the extraction are still visible above and around the cave entrance. The better-preserved upper sediments at LRC IV (Fig 5D) show that U1 could have been up to ten meters thick until 1846 and would have covered the entire slope. The reconstruction of these three main steps of La Roche-Cotard’s morphological evolution, from Neanderthal occupations until today, are presented in Fig 7.

### Archaeological material at LRC

Faunal remains of large and medium-size mammals were discovered in 1912 but unfortunately the exact locations inside the cave were not recorded. During our more recent excavations, many faunal remains of large mammals were found, especially in LRC III and IV, and these were recorded in terms of both stratigraphy and spatial coordinates. Some of the bones, from all four loci, show anthropogenic traces such as cut marks, some appear to have been burnt and others used for tool production. The large mammals exploited are mainly those occurring during temperate periods, the bovines bison and aurochs, and equids and red deer. The site was occupied firstly by carnivores (cave lion and bear), then by humans, and lastly by hyenas. At LRC I (Upper layer), LRC III (layer 6) and LRC IV (layers 11 to 7d) faunal elements are characteristic of cold climates (e.g., reindeer and marmot, Table 1). Numerous remains of small vertebrates (mammals, fish, amphibians, birds and reptiles) have also been collected by sieving (1 mm mesh sieve) in all *loci*.

**Table 1:** Archaeological material found in LRC I (cave).

Layer	Sedimentary unit	Location	Lithics	Faunal remains
Upper	U3	Lemmings Chamber and outside	No lithics	Wolf, Reindeer, Marmot, Collared Lemming, Tundra Lemming
Middle	U4	Lemmings Chamber, Mousterian gallery and outside	No lithics	Equidae (*E*. *caballus* and *E*. *hydruntinus*), Bovidae (Bos/Bison), Cervidae, Hyena
Lower (Acheulean tradition Mousterian)	U4	Mousterian gallery	Biface, Levallois flakes	Bovidae (Bos/Bison), Equidae (*E*. *caballus* and *E*. *Hydruntinus*), Cervidae, Hyena
Lower (Levallois typical Mousterian)	U4	Mousterian gallery and outside	Levallois flakes	Bovidae (Bos/Bison), *Equus sp*., Cervidae, Hyena
Layers 1 and 2	?	Pillar Chamber North	No lithics	Lepus, Oryctolagus, Vulpes, Meles
Layer 3	?	Pillar Chamber North	1 blade with use-wear traces on the top of the layer	One Hyena tooth and small pieces of bone
Layer under Quartzite sandstone	?	Pillar Chamber South	Triangular biface with use-wear traces	No bones

Human frequentation is attested by lithic artefacts at all loci. It is important to note that only Mousterian lithic artefacts were discovered, either within or outside the cave; no later-period material was found.

#### LRC I

In LRC I, d’Achon’s excavations in 1912 revealed two assemblages (see S2 Fig for a schematic view of the find locations and of the lithics). Unfortunately, d’Achon’s collection was lost; it is only known from ten drawings and a single photograph [43]. These documents show Mousterian lithics, including (i) bifaces (S3 Fig) found in the top part of the Lower layer (in area 1 in S2 Fig) and (ii) flakes, presumably obtained by Levallois debitage, in the bottom part of the same layer [54]. This second set of lithics was also found in the cave entrance (area 2 in S2 Fig), underneath the stones of a structure interpreted by d’Achon as a fireplace [43]. Our excavations revealed more lithics: one Mousterian biface was found in the remnants of an unidentified layer in the South Pillar Chamber (S4A Fig); thin elongated flakes (S4B Fig) were found in the Lower layer, below d’Achon’s excavations, in the northern part of the Mousterian Gallery (areas 3, 4 and 5 in S2 Fig). The flakes appear similar to those found by d’Achon stratigraphically below the location of the bifaces. Finally, one blade (S2 and S5B Figs) was found on the surface of layer 3 in the Pillar Chamber, i.e., under the disturbed layer 2. Unfortunately, because of its location, this artefact cannot be securely placed in stratigraphic context: firstly, because the sediment in the Pillar Chamber cannot be confidently linked with other sedimentary sequences, and secondly because it was lying on the layer 3 surface.

Despite the small size of the assemblage discovered in the *Mousterian Gallery*, it is clear that several Lower Turonian flint varieties were used, all locally available in the form of pebbles in the river terraces. The morphology of five discovered flakes (S4B Fig) and their detachment stigmata correspond to direct percussion with hard hammer. These features enable us to attribute these pieces to the Levallois reduction strategy [55, 56], probably of the recurrent centripetal type.

All lithic artefacts were cleaned in an ultrasonic bath and occasionally with soap and warm water. A Leica MZ 125 stereomicroscope (up to x100 magnification) and a Leica metallurgical microscope (optics ranging from x50 to x500 magnification) were used for the functional study of these lithic implements, with digital photography (Stream-Olympus and Leica Application Suite) to allow the capture and manipulation of detailed images of use-wear from the microscope. Microwear analysis of the incomplete triangular biface (S5A Fig) discovered in the Pillar Chamber reveals a contact with mineral matter (S5 Fig). The distal end of the small blade discovered on the surface of layer 3, was modified by direct abrupt retouch and used to process hide (S5B1 Fig) and abrasive, mineral material (S5B2 Fig). On the other end of the blade, the edges display use-wear traces indicating also engraving of a soft, abrasive, mineral matter (S5B2–S5B5 Fig). Similar traces were reproduced on experimental flint tools used to work fragments of the chalk and the quartzitic sandstone wall fragments collected from the remains of 1912 excavated material.

#### Other loci

A Mousterian industry, based on Levallois debitage, was also found at LRC II in layer 7 (sedimentary unit U4), in the same layer as the “Mask of La Roche-Cotard” [10, 11]. In the LRC III excavation, layers 8 and 9 (sedimentary unit U4) yielded a small Mousterian assemblage with Discoid debitage. Layer 7 contains numerous faunal remains introduced, at least partly, by hyenas. Layer 6 yielded remains of cold climate-adapted rodents (Arctic lemming, narrow-headed vole), but–like layer 7 –no industry, either osseous or lithic. Finally, LRC IV also yielded evidence of a Mousterian industry (Levallois) in layer 13 (unit U4), associated with large mammal remains (temperate to cool climate-adapted species) [50]. In U2, layer 9 contains arctic lemming remains but no artefacts.

Thus, all the lithic artefacts found both within the cave and in the three other nearby loci (LRC II, III and IV) were found in sedimentary unit U4, at least where it can be confidently identified (thus excluding the artefacts found in the Pillar Chamber, since these are not stratigraphically correlated to the layers outside the cave). All lithics are characteristic of Mousterian industries. In Western Europe, such lithic industries are assigned to *H*. *neanderthalensis* [57–59]. In addition, no lithic artefact displays any signs of transport by water.

### Summary of the context and arising questions

The different loci of La Roche-Cotard were occupied by human groups during the period when sedimentary unit U4 was deposited. This presence is attested by the lithic assemblages found in LRC I, II, III and IV; bifaces and Levallois flakes were found in the cave (LRC I). In addition, engravings were made on the walls of the Pillar Chamber at LRC I. Since no other, more recent occupations (until the 19^th^ century) have left traces in the cave, it is tempting to associate the engravings with the lithics found in the cave. However, no direct link can be established between these sets of records. Luckily, the cave entrance was obstructed by sediments after the lithic artefacts were left inside the cave and, as we will show below, also after the engravings were made. To further constrain the time of manufacture of the engravings, we need to know when the entrance of LRC I was sealed.

## Materials and methods

### Observation, photogrammetric coverage and surveys

The entire cave (LRC I) was modelled using photogrammetry to precisely locate the engravings. This protocol initially includes reflex photography with a wide-angle lens (11 mm) to cover the entire area of the cave despite narrow spaces at around 1 mm spatial resolution. Then, a coverage closer to the walls was carried out with a 24 mm focal length to increase the spatial resolution on the areas of interest of the digital panels (less than 1 mm). Coverage using the same focal length was also carried out on the junction zone between the Mousterian Gallery and the exterior of the cave to allow a better connection with the Lidar scan (Fig 3). The first step in the examination of the walls of the cave used 155 spatially located images in surveys to distinguish and locate the different types of parietal traces: (i) natural, geomorphological traces showing either deposition or removal of material from the cave wall; (ii) suspected old and recent animal traces; (iii) suspected ancient and recent anthropogenic traces and (iv) indeterminate traces. The second step consisted of the photographic and photogrammetric coverage (resolution <1 mm) of apparently ancient anthropogenic or animal traces (the antiquity of the marks will be discussed in the following section). Images of the panels were also processed with *Reflectance Transformation Imaging* (RTI). The third step consisted of reproducing, by drawing, the characteristics of the panels and recording on a summary sheet very detailed observations such as location, support and surface condition, description and diagram of traces, dimensions, state of conservation, etc. Three surveys were made separately: first a survey of geomorphological features, then of animal traces and finally of supposedly anthropogenic or indeterminate traces. The work carried out on anthropogenic or indeterminate traces also included the completion of a sheet specifying as many details as possible, i.e., numbering of the unit traces, shapes of the edges, of the starting point and the end of the traces, direction of movement of the supposed finger (when possible), etc. These different surveys were made on site, in front of the wall, on a transparency fixed on an orthophoto of the analysed panel. The three surveys were then redrawn on three new transparencies, and subsequently scanned.

### Morphometric analysis of cave wall traces and experimental engraving

To distinguish between animal and anthropogenic marks, we measured their widths and incision angles. The depth was also measured (sometimes estimated from the first two measurements). We recorded these measurements from sections generated from the 3D model produced with Agisoft metashape from a Sony A55, 4912 x 3264 pixels de 5 μ, focale 55 mm, distance 1.60 m (resolution 0.15 mm). A linear discriminant analysis (LDA) of these three parameters was undertaken with different packages of the R software [60, 61], highlighting the differences between the traces. An experiment (S1 Text) was conducted to test the feasibility of discriminating between the more likely finger- and tool-made engravings. To avoid any risk of damage to the cave walls, this experiment was conducted off site (pilot cave); we selected a nearby cavity dug three or four centuries ago in the same type of rock (i.e., Turonian yellow tuff) whose wall was covered with a soft-surface coating, but not exactly similar to that at La Roche-Cotard (LRC I). The “operators” created marks on the wall of the Turonian tuff, using a variety of tools: flat fingers, edged fingers, bone, wood, antler, flint and metal points. One operator made the marks and an independent “observer” filled in the prepared form. Photogrammetry of the panels was carried out and measurements were made using CloudCompare. These data have also been statistically processed. The comparison of the results obtained with those drawn from the analysis of the traces observed in the cave allows us postulate hypotheses on the latter.

In order to identify ancient marks, we have also compared the structural and physical characteristics of ten visually distinct marks on the LRC cave walls thought to be made with metal tools–and therefore recent–with those presumed to be Palaeolithic traces. These recent marks are attributed to the discoverer, co-workers and excavators of the cave in 1846, and later during the excavations of 1912, and/or to potential visitors who may have entered the cave since 1912. These modern marks are located mostly below the overhang, rarely above it. We used Munsell Soil-Color Charts to record the colours on three surface categories (marks assumed to be modern, those believed to be palaeolithic, and the surface of the wall coating). In addition, a colorimeter (Konica-Minolta CM-600d) measured the colour of the three surfaces. Each measurement provides three independent lines of data, i.e., the values of L* (luminance, between 0 and 100% from black to white), a* (increasing values of green toward red), and b* (increasing values of blue toward yellow). The LDA analysis was carried out using the R software packages mentioned above.

### Dating

To constrain the age of the engravings, we considered a range of dating methods. The calcite film covering some of the finger flutings was very thin, so Uranium-Thorium series (U-Th) dating could not be used to constrain the chronology of the engravings. However, using OSL, it was possible to determine when the cave was sealed by sediment, because two complete stratigraphic sequences (LRC I-II and LRC IV) were found outside the cave. In addition, unexcavated sediment remains immediately (i) inside the cave entrance, (ii) in two niches in the cave entrance and (iii) around the cave entrance (both above and below) (Fig 6). OSL dating determines the time of sediment deposition [62] and so provides information on when the cave entrance was last blocked by deposition of the sediment still present around the entrance and over the escarpment.

#### Radiocarbon

Radiocarbon (14C) [63] was used to date bone fragments. In total, 19 bone samples were selected: in 1978, three samples were measured using conventional counting after classic preparation. More recently, to select the most suitable samples for radiocarbon dating and to avoid unnecessary destruction of material, preliminary analyses were carried out on selected bones, to check the conservation of the collagen, to determine the quantity of sample necessary for dating and to test for contamination. These investigations included elemental analysis, which provides information on the percentage of nitrogen and carbon retained in a bone. The state of conservation of collagen is considered to be satisfactory for a nitrogen content of up to ca. 0.4% [64, 65]. Below this value, radiocarbon dating of the bones should not be attempted. This analysis also makes it possible to detect the presence of carbon contamination (secondary carbonation, humic acids, etc.). Sixteen bones were prepared in the Lyon laboratory, with the collagen extracted by the modified Longin method [66]. Fourteen of the resulting collagen samples were treated with ultrafiltration. The other two samples gave insufficient collagen after ultrafiltration [67], so total collagen was used instead. Radiocarbon measurements were made in the Saclay laboratory using Accelerator Mass Spectrometry (AMS).

#### Optically Stimulated Luminescence (OSL)

Optically stimulated luminescence (OSL) dating is a well-established absolute chronological method that determines the time since sedimentary grains were last exposed to daylight (i.e., the burial age). A total of 50 sediment samples were collected from 2016 to 2022 in the four loci for luminescence dating. Of these, 43 samples were analysed in Denmark (DTU Physics) and the remaining seven samples in Hungary (Department of Geological Basic Research, Mining and Geological Survey of Hungary). Some multi-grain quartz OSL ages (12 of the 43 samples, all from LRC IV, see below) analysed in Denmark, were published in Marquet *et al*. [50] and the seven multi-grain quartz samples analysed in Hungary were published in Marquet *et al*. [45, 50]. In this study we date, for the first time, the 31 remaining samples and also recalculate the previously published quartz ages using an improved assumption concerning water content, and revised methodology to deal with gamma dose rate heterogeneity (see S2 Text for more details). Sediment samples were taken by inserting steel tubes (ø = 4 cm, length = 20 or 15 cm) into cleaned sections. In the laboratory, samples were prepared under subdued red-orange light conditions. The ends (outer 5 cm) of each sample, potentially light-exposed during sampling, were reserved for radionuclide concentration and water content measurements. The inner portions of the samples were used for luminescence measurements. For the latter, standard chemical procedures were used to obtain clean quartz and K-rich feldspar (KF) extracts (S2 Text). Equivalent doses were determined using the single-aliquot regenerative-dose (SAR) procedure [68] on multi-grain quartz and K-rich feldspar aliquots using the 180–250 μm grain size fraction. Twelve of the quartz extracts were also measured using single-grain OSL techniques.

The bleaching, or resetting, rate of K-rich feldspar Infra-Red Stimulated Luminescence (IRSL) signals is at least an order of magnitude slower than that from quartz [69, 70], so if quartz and feldspar ages are comparable, the sediment was most likely well-bleached at burial [71]. The post-Infra Red–IRSL (pIRIR_50,290_) protocol [72] was employed to make multi-grain K-rich feldspar measurements on all samples measured at DTU, Denmark. In addition, single-grain quartz measurements on 18,900 individual grains were undertaken to complement the multi-grain quartz results. The laboratory-measured OSL dose response curves were fitted using a single saturating exponential function and individual equivalent dose (*D_e_*) estimates obtained by interpolation. Uncertainties on individual *D_e_* values are based on counting statistics, fitting uncertainties and an instrument reproducibility of 0.5% per OSL measurement for multi-grain measurements and 2.5% per OSL measurements for single grain measurements [73]. Multi-grain quartz and K-feldspar average doses are derived using the arithmetic mean [74], whereas single-grain dose estimates have been analysed in three different ways using: i) the central age model (CAM) [74], ii) the average dose model (ADM) [75] and iii) the Bayesian central dose model (BayLum) [76–80]. Consistency between the single-grain and multi-grain quartz doses is only achieved when the *D_c_* criterion [81] is applied to select those single grains suitable for estimating the dose of interest.

Additional information concerning sample preparation, detailed experimental procedures, the effect of applying standard rejection criteria and comparison between quartz and K-rich feldspar measurements are given in the S2 Text. Radionuclide concentrations were determined using high-resolution gamma spectrometry [82] for all sediment samples as well as a single bedrock sample. Some of the samples were taken in close proximity to bedrock, which has a gamma dose rate ~3 times lower than the sediment samples, causing a heterogeneity in the gamma field. A model relying on the principle of superposition and the infinite matrix assumption [83] was used to correct for this heterogeneity. In addition, in situ dose rate measurements using a LaBr probe were undertaken in two sediment sample positions and in one hole in the cave wall, and Al_2_O_3_:C pellets were placed in 4 sediment sample positions and in one hole in the cave wall. A comparison between these in situ results and those derived from laboratory modelling demonstrates that the modelling results are satisfactory. In other aspects of dose rate calculations, we argue that the best estimate of long-term fractional water content is based on the present-day values from the least disturbed parts of the site, and this results in the adoption of a water content of 40% of the measured saturated water content (averaged for each of the five units, see description below). Dose rate calculations for the grain size range assume an internal dose rate to quartz of 0.02±0.01 Gy ka^−1^ and to KF of 0.10±0.05 Gy ka^−1^ from U and Th. The cosmic ray dose rate contribution is calculated for each individual sample using a burial depth estimate based on the original excavation records from 1846 [50] (the average contribution for all samples is 0.064±0.003 Gy ka^−1^). In addition, the feldspar ages all include an internal dose rate component of 0.86±0.06 Gy ka^−1^ derived from the measured average K-content of 12.60±0.15% (n = 21) and an assumed Rb-content of 400 ppm. Finally, beta and gamma dose rates are calculated using the conversion factors of Guérin *et al*. (2011) [84]; grain size attenuation of the beta component was accounted for using the factors of Guérin *et al*. (2012) [85]. Further details regarding the dose rate modelling (including how the model compares to Monte Carlo simulations and in situ dose rate measurements) and the water content assumption are given in the S2 Text.

In total, we derived 50 OSL burial ages [62, 86] from LRC Ia to Id (n = 20), LRC II (n = 14), LRC III (n = 1), and LRC IV (n = 15) using the blue-light stimulated luminescence signals from multi-grain (8 mm) quartz aliquots, as well as the infrared stimulated luminescence (pIRIR) [72] signal from multi-grain K-feldspar aliquots (2 mm). Twelve quartz samples were also measured using the green-simulated OSL signal from single-grain quartz aliquots.

## Results

### Discrimination of wall marks

**Fig 8:**
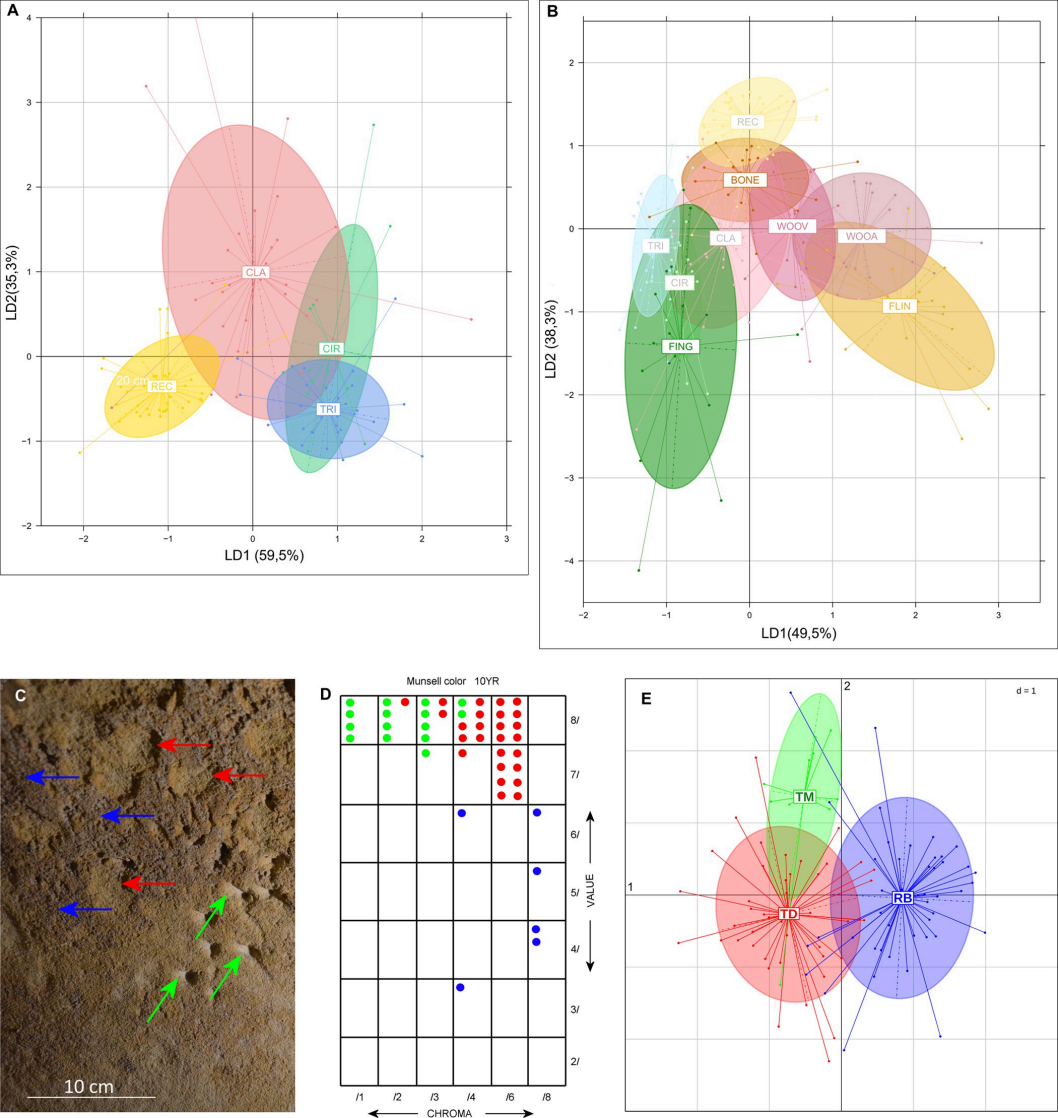
Morphometric discrimination of wall marks. A. Linear discriminant analysis of widths, incision angles and depth of marks on the wall of LRC I cave. “TRI”: Triangular Panel, “CIR”: Circular Panel, “REC”: Rectangular Panel, “CLA”: claw marks surface. S7 Fig gives elements showing how measurements have been made. B. Linear discriminant analysis of widths, incision angles and depth of experimentally created marks on the Turonian yellow tuff wall of a pilot cave. Marks made with finger: “FING”, with bone: “BONE”, with wood: “WOOV”, with antler: “WOOA” and with flint: “FLIN”. S7 Fig shows example results of modern engraving. C. Photograph of a cave wall in LRC I. Modern marks (green arrows), located on the Dotted Panel (traces 27–35), are attributed to the excavation of the cave in 1912. Red arrows point to marks believed to be old, and blue arrows to the surface of the wall coating. D. Table of determinations of Munsell Soil-Color Chart for colours depicted on the three surface categories pointed in (C). E. LDA processing of 99 marks using L* a* b* colour parameters, 45 for Brown coating (RB), 44 for Finger impacts (TD) and 10 for Modern marks (TM).

The numerous marks on the soft surface layers of the walls of LRC I have been categorised according to origin: those made by humans must be distinguished from those made by animals, as well as those arising from local geochemical alteration (surface dissolution, disintegration, dehydration), and minor chemical deposits (concretions). Animal claw marks (S6 Fig), attributable to *Ursus sp*., *Meles sp*. and other species [87], can be identified by their characteristic spacing and incision angle. But alongside these numerous, randomly distributed animal scratch marks, there are also a number of elongated or dotted, spatially organized marks. These organised marks are found only on the 13 m long north-east wall of the pillar chamber (shown with a blue line in Fig 2A). They have distinct geometric shapes and are often grouped into panels separated by groups of smaller marks. LDA analysis (Fig 8A, Table 2) based on the width, incision angle and depth of 116 marks revealed two statistically distinct groups: 32 with features consistent with claw marks [87], and 84 most likely of anthropogenic origin. Those identified as claw marks are thinner, deeper and have a V-shaped cross-section, whereas the presumed ancient spatially organised marks are mostly wider, shallower, and U-shaped, consistent with the morphology of a fingertip or similarly shaped tool. However, the rectangular panel is clearly separated, first from the two panels made with fingers and secondly separated from the claw marks.

**Table 2:** Measures of width, incision angles and depth of the parietal and experimental marks.

ID	Type	location of the measured section on the trace	n° of the trace on the survey	Width (mm)	Depth (mm)	Angle (°)	Section	Depth calculated (*)
TRI-01	TRI	T1a	16	14.3	1.3	156	U	
TRI-02	TRI	T1b	15	11.9	0.7	159	U	
TRI-03	TRI	T1c	14	15.5	0.8	167	U	
TRI-04	TRI	T1d	7	12.7	1.2	162	U	
TRI-05	TRI	T1e	6	16.8	0.9	151	U	
TRI-06	TRI	T1f	5	12.1	0.4	178	U	
TRI-07	TRI	T1g	4	7.0	0.6	170	U	
TRI-08	TRI	T1h	3	17.4	1.2	165	U	
TRI-09	TRI	T2a	17	12.3	0.4	164	U	
TRI-10	TRI	T2b	16	14.0	1.1	159	U	
TRI-11	TRI	T2c	15	8.3	1.1	159	U	
TRI-12	TRI	T2d	14	9.8	0.5	162	U	
TRI-13	TRI	T2e	9	17.8	1.0	162	U	
TRI-14	TRI	T2f	8	21.6	1.2	166	U	
TRI-15	TRI	T2g	7	22.2	0.7	169	U	
TRI-16	TRI	T2h	6	15.9	1.0	166	U	
TRI-17	TRI	T2i	5	18.8	1.5	153	U	
TRI-18	TRI	T2j	3	23.4	2.4	154	U	
TRI-19	TRI	T3a	16	13.9	0.7	169	U	
TRI-20	TRI	T3b	15	15.6	0.4	165	U	
TRI-21	TRI	T3c	14	12.7	0.8	164	U	
TRI-22	TRI	T3d	11	13.2	0.3	177	U	
TRI-23	TRI	T3e	10	15.9	0.6	163	U	
TRI-24	TRI	T3f	9	11.3	0.4	178	U	
TRI-25	TRI	T3g	8	12.0	0.4	178	U	
TRI-26	TRI	T3h	7	15.8	0.7	162	U	
TRI-27	TRI	T4a	8	16.7	1,0	164	U	
TRI-28	TRI	T4b	7	18.7	0.9	166	U	
TRI-29	TRI	T4c	6	15.3	1.1	163	U	
TRI-30	TRI	T4d	5	15.8	0.7	165	U	
TRI-31	TRI	T4e	4	17.1	0.9	166	U	
REC-01	REC	1		13.4	1.6	153	U	*
REC-02	REC	2		2.9	0.5	140	V	*
REC-03	REC	3		10.4	2.1	136	U	*
REC-04	REC	4		3.9	0.7	138	V+	*
REC-05	REC	5		5.5	0.7	151	U	*
REC-06	REC	6		2.3	0.4	143	V	*
REC-07	REC	7		3.4	0.6	140	U	*
REC-08	REC	8		2.7	1.0	107	V	*
REC-09	REC	9		5.0	0.6	154	U	*
REC-10	REC	10		3.0	0.5	141	V+	*
REC-11	REC	11		5.5	0.9	143	U	*
REC-12	REC	12		4.6	1.0	133	V	*
REC-13	REC	13		2.3	0.5	133	V	*
REC-14	REC	14		6.5	0.8	153	U	*
REC-15	REC	15		1.8	0.6	110	V	*
REC-16	REC	16		2.1	0.8	107	V	*
REC-17	REC	17		0.8	0.5	80	V	*
REC-18	REC	18		2.6	0.6	129	V+	*
REC-19	REC	19		1.9	0.5	129	V+	*
REC-20	REC	20		2.6	0.5	140	V+	*
REC-21	REC	21		4.8	0.8	144	V+	*
REC-22	REC	22		2.7	1.3	92	U+	*
REC-23	REC	23		4.0	1.0	127	V+	*
REC-24	REC	24		5.2	0.6	153	V+	*
REC-25	REC	25		3.8	0.5	149	V+	*
REC-26	REC	26		2.2	0.6	123	V+	*
REC-27	REC	27		3.2	0.9	123	V+	*
REC-28	REC	28		2.1	0.8	107	V	*
REC-29	REC	29		3.0	0.5	143	V+	*
REC-30	REC	30		4.5	0.6	148	U+	*
REC-31	REC	31		6.0	1.8	119	V+	*
REC-32	REC	32		7.2	1.3	140	U+	*
REC-33	REC	33		2.6	0.9	113	V	*
REC-34	REC	34		4.5	1.0	130	V+	*
REC-35	REC	35		6.4	1.8	122	V+	*
REC-36	REC	36		2.6	0.7	121	V	*
REC-37	REC	37		5.7	1.5	126	V	*
REC-38	REC	38		2.9	1.4	93	V+	*
REC-39	REC	39		3.9	1.0	126	U	*
CLA-01	CLA	1		14.3	6.4	96	V+	*
CLA-02	CLA	2		16.7	2.1	152	V+	*
CLA-03	CLA	3		11.4	2.5	133	V	*
CLA-04	CLA	4		16.1	3.2	137	V	*
CLA-05	CLA	5		13.5	2.7	136	V	*
CLA-06	CLA	6		13.7	2.4	142	V	*
CLA-07	CLA	7		8.2	1.9	130	V	*
CLA-08	CLA	8		14.5	2.6	141	V	*
CLA-09	CLA	9		15.1	2.7	141	V+	*
CLA-10	CLA	10		9.6	2.1	133	V	*
CLA-11	CLA	11		9.8	4.3	98	V	*
CLA-12	CLA	12		10.9	1.7	145	V+	*
CLA-13	CLA	13		10.0	1.3	151	V+	*
CLA-14	CLA	14		10.0	2.0	137	V	*
CLA-15	CLA	15		7.7	0.7	159	V+	*
CLA-16	CLA	16		9.2	1.6	141	V	*
CLA-17	CLA	17		6.1	0.7	153	V	*
CLA-18	CLA	18		9.4	1.3	149	V	*
CLA-19	CLA	19		13.7	1.2	160	V+	*
CLA-20	CLA	20		12.0	2.0	143	V	*
CLA-21	CLA	21		12.5	2.7	134	V	*
CLA-22	CLA	22		2.0	0.8	103	V+	*
CLA-23	CLA	23		14.5	2.1	147	V+	*
CLA-24	CLA	24		19.9	3.0	146	V	*
CLA-25	CLA	25		10.7	2.1	137	V	*
CLA-26	CLA	26		17.1	1.7	157	V+	*
CLA-27	CLA	27		14.2	3.0	134	V	*
CLA-28	CLA	28		15.6	1.9	152	V	*
CLA-29	CLA	29		14.7	2.2	147	V+	*
CLA-30	CLA	30		29.1	2.4	161	V+	*
CLA-31	CLA	31		12.9	2.2	143	V	*
CLA-32	CLA	32		18.5	4.1	132	V	*
CIR-01	CIR	C1a	16	24.6	4.2	146	U	
CIR-02	CIR	C2a	9	16.0	2.0	151	U	
CIR-03	CIR	C2b	10	14.6	1.1	152	U	
CIR-04	CIR	C3a	9	15.8	1.2	156	U	
CIR-05	CIR	C4a	1	12.6	1.2	154	U	
CIR-06	CIR	C4b	2	15.5	1.4	148	U	
CIR-07	CIR	C5a	1	20.4	1.7	154	U	
CIR-08	CIR	C5b	2	16.6	1.4	153	U	
CIR-09	CIR	C5c	3	18.1	1.3	153	U	
CIR-10	CIR	C5d	4	14.8	1.2	156	U	
CIR-11	CIR	C6a	1	24.5	3.2	148	U	
CIR-12	CIR	C6b	2	18.1	0.7	161	U	
CIR-13	CIR	C6c	3	13.8	0.5	153	U	
CIR-14	CIR	C6d	4	16.8	2.1	147	U	
WOOA-01	WOOA	WA-C1a		12.3	4.9	93		
WOOA-02	WOOA	WA-C1b		11.3	2.9	116		
WOOA-03	WOOA	WA-C1c		10.3	4.2	96		
WOOA-04	WOOA	WA-C1d		12.5	6.3	92		
WOOA-05	WOOA	WA-C1e		10.7	7.8	71		
WOOA-06	WOOA	WA-C1f		10.4	7.1	78		
WOOA-07	WOOA	WA-C1g		10.5	4.2	102		
WOOA-08	WOOA	WA-C1h		12.1	6.0	99		
WOOA-09	WOOA	WA-C1i		12.4	5.3	96		
WOOA-10	WOOA	WA-C1j		17.9	5.2	111		
WOOA-11	WOOA	WA-C1k		14.3	6.2	108		
WOOA-12	WOOA	WA-C2l		6.5	4.5	68		
WOOA-13	WOOA	WA-C2m		6.5	3.2	80		
WOOA-14	WOOA	WA-C2n		6.9	3.8	74		
WOOA-15	WOOA	WA-C2o		7.4	5.2	78		
WOOA-16	WOOA	WA-C2p		6.3	3.6	77		
WOOA-17	WOOA	WA-C2q		9.3	5.5	88		
WOOA-18	WOOA	WA-C2r		7.5	8.3	53		
WOOA-19	WOOA	WA-C2s		6.9	2.3	107		
WOOA-20	WOOA	WA-C2t		6.2	3.8	77		
WOOV-01	WOOV	WV-C1a		15.0	4.4	113		
WOOV-02	WOOV	WV-C1b		10.2	4.4	95		
WOOV-03	WOOV	WV-C1c		10.9	2.9	116		
WOOV-04	WOOV	WV-C1d		6.5	2.2	95		
WOOV-05	WOOV	WV-C1e		10.8	3.5	110		
WOOV-06	WOOV	WV-C1f		9.5	3.3	114		
WOOV-07	WOOV	WV-C1g		8.7	3.0	83		
WOOV-08	WOOV	WV-C1h		7.4	1.7	114		
WOOV-09	WOOV	WV-C1i		8.8	1.5	134		
WOOV-10	WOOV	WV-C1j		5.7	1.6	119		
WOOV-11	WOOV	WV-C1k		9.9	2.9	108		
WOOV-12	WOOV	WV-C1l		8.2	1.3	123		
WOOV-13	WOOV	WV-C1m		6.4	2.9	106		
WOOV-14	WOOV	WV-C2n		11.6	3.9	110		
WOOV-15	WOOV	WV-C2o		11.9	5.1	103		
WOOV-16	WOOV	WV-C2p		13.5	3.3	109		
WOOV-17	WOOV	WV-C2q		20.6	4.7	93		
WOOV-18	WOOV	WV-C2r		15.7	2.8	126		
WOOV-19	WOOV	WV-C2s		11.6	2.6	117		
WOOV-20	WOOV	WV-C2t		13.5	4.2	107		
FLIN-01	FLIN	FL-C1a		13.2	4.0	109		
FLIN-02	FLIN	FL-C1b		11.6	2.4	110		
FLIN-03	FLIN	FL-C1c		15.3	3.7	116		
FLIN-04	FLIN	FL-C1d		13.6	6.7	82		
FLIN-05	FLIN	FL-C1e		10.2	7.7	64		
FLIN-06	FLIN	FL-C1f		18.1	7.1	104		
FLIN-07	FLIN	FL-C1g		15.9	8.4	84		
FLIN-08	FLIN	FL-C1h		11.4	6.6	83		
FLIN-09	FLIN	FL-C1i		13.1	8.1	62		
FLIN-10	FLIN	FL-C1j		11.7	4.4	100		
FLIN-11	FLIN	FL-C2k		7.4	4.5	57		
FLIN-12	FLIN	FL-C2l		12.9	9.6	81		
FLIN-13	FLIN	FL-C2m		18.2	13.4	83		
FLIN-14	FLIN	FL-C2n		13.8	10.2	80		
FLIN-15	FLIN	FL-C2o		11.9	8.8	90		
FLIN-16	FLIN	FL-C2p		12.5	9.2	94		
FLIN-17	FLIN	FL-C2q		12.3	9.1	84		
FLIN-18	FLIN	FL-C2r		16.0	11.8	82		
FLIN-19	FLIN	FL-C2s		20.2	14.9	114		
BONE-01	BONE	BO-C1a		5.8	1.3	128		
BONE-02	BONE	BO-C1b		7.6	1.4	137		
BONE-03	BONE	BO-C1c		9.9	2.4	138		
BONE-04	BONE	BO-C1d		10.3	0.5	123		
BONE-05	BONE	BO-C1e		7.7	1.9	139		
BONE-06	BONE	BO-C1f		6.2	0.8	145		
BONE-07	BONE	BO-C1g		8.1	1.2	153		
BONE-08	BONE	BO-C1h		10.4	2.1	125		
BONE-09	BONE	BO-C1i		13.8	2.3	117		
BONE-10	BONE	BO-C1j		6.9	1.6	135		
BONE-11	BONE	BO-C1k		7.5	1.7	141		
BONE-12	BONE	BO-C1l		12.9	0.6	172		
BONE-13	BONE	BO-C1m		9.3	1.2	156		
BONE-14	BONE	BO-C2n		9.6	3.0	108		
BONE-15	BONE	BO-C2o		6.9	0.8	115		
BONE-16	BONE	BO-C2p		8.2	1.2	133		
BONE-17	BONE	BO-C2q		8.4	3.2	101		
BONE-18	BONE	BO-C2r		6.9	1.1	124		
BONE-19	BONE	BO-C2s		5.0	3.0	79		
BONE-20	BONE	BO-C2t		6.2	1.2	129		
FING-01	FING	FI-C1a		39.5	4.0	153		
FING-02	FING	FI-C1b		19.1	2.6	141		
FING-03	FING	FI-C1c		25.2	4.6	148		
FING-04	FING	FI-C1d		10.4	0.6	154		
FING-05	FING	FI-C1e		22.8	2.5	152		
FING-06	FING	FI-C1f		11.2	1.6	159		
FING-07	FING	FI-C1g		16.9	0.9	153		
FING-08	FING	FI-C1h		16.2	1.2	156		
FING-09	FING	FI-C1i		23.6	2.1	149		
FING-10	FING	FI-C1j		19.0	1.9	149		
FING-11	FING	FI-C2k		22.2	2.1	163		
FING-12	FING	FI-C2o		18.1	5.6	118		
FING-13	FING	FI-C2p		32.2	5.7	136		
FING-14	FING	FI-C2q		31.3	2.9	159		
FING-15	FING	FI-C2r		24.8	1.5	157		
FING-16	FING	FI-C2s		18.8	0.9	157		
FING-17	FING	FI-C2t		21.2	2.2	156		

Measurements made on the marks found in LRC I on the four surfaces: triangular (TRI), circular (CIR), rectangular (REC) and with *Ursus* claw marks (CLA), and on experimental mark groups, made with fingers (FING), bones (BONE), wood (WOOV), antler (WOOA) and flint (FLIN). See S3 Text for the R Code used and statistical data processing. The 1^st^ column gives the trace ID for the diagrams, the 2^nd^ gives the type, the 3^rd^ indicates location of the measured section on the trace. For example, T1a indicates that it is trace a, on section n°1 of the Triangular Panel (T). The 4^th^ column indicates, for the Triangular and Circular Panels, on which line of the survey the measurements were made. On some lines, more than one measurement could be made. For the Rectangular Panel and the scratched surface, the measured sections are not located as indicated in the legend of S7 Fig. In the following four columns, the width (l), depth (d), angle of incision (a) of the trace and the general shape of the trace section are indicated (U: U-shaped section, V: V-shaped section; “+” indicates perfection of form). The last column marks, with an asterisk, traces whose depth has not been measured but calculated with a simple formula (S7 Fig).

The results of the LDA processing of the morphometric characteristics of the experimental marks created off-site on the wall of a nearby cavity show a clear separation of the experimentally created marks into 2 groups (Fig 8B, Table 2, S7 Fig): the group of traces made using fingers (FING) which coincides with the ellipses of the two Circular and Triangular Panels and a relatively homogeneous group of ellipses of the traces made with the other four tools: antler (WOOA), vegetable wood (WOOV), bone (BONE) and finally flint (FLIN). This second group does not coincide with the ellipse of the Rectangular Panel. In summary, the experiments using fingers support the hypothesis that our parietal tracings are indeed digital tracings, apart from the Rectangular Panel. Because of the considerable alteration of the surface of this panel, our experiments do not help in identifying the tools used there, even if its ellipse has a common part with the one obtained by bone tool. A retouched blade (S5B Fig), of which the traceological analysis indicates that the use-wear traces are consistent with the processing of mineral matter, was found in front of the Undulated Panel, 2.5 m from the Rectangular Panel, but it remains impossible to hypothesize, at this stage, if this blade was used for the production of this panel.

We have compared the presumed modern and ancient anthropogenic engravings. In Fig 8C and 8D, the depiction of the three categories of surface colours is given with Munsell Soil-Color Charts. LDA processing of 99 marks using L*, a* and b* colour parameters are presented in Fig 8E and Table 3. The results from both Munsell Soil-Color Charts and LDA processing of L*, a* and b* data used for discrimination of colours on the three surface categories show a clear distinction between the three groups: the modern marks (green arrows) made during the cave’s excavation in 1912, those believed to be old (red arrows), and the surface of the wall coating (blue arrows).

**Table 3:** Measures given by spectrocolorimeter on the wall coating, anthropogenic and modern marks.

Brown coating (RB)				Finger flutings (TD)				Modern traces (TM)			
n°	L*	a*	b*	n°	L*	a*	b*	n°	L*	a*	b*
RB-01	51.94	6.66	20.29	TD-01	65.47	6.04	22.55	TM-01	59.56	7.03	23.21
RB-02	57.03	6.59	15.23	TD-02	55.97	5.71	21.48	TM-02	42.00	3.81	13.95
RB-03	47.60	2.76	10.79	TD-03	55.82	5.91	20.49	TM-03	48.67	3.87	12.71
RB-04	56.37	6.67	20.33	TD-04	63.24	6.23	22.76	TM-04	76.35	4.13	16.53
RB-05	62.55	7.21	17.70	TD-05	52.08	3.42	14.41	TM-05	53.75	4.43	15.69
RB-06	48.57	7.56	18.69	TD-06	54.77	6.08	20.27	TM-06	43.96	3.62	11.49
RB-07	58.96	7.78	21.02	TD-07	52.79	5.08	18.34	TM-07	41.31	4.78	13.52
RB-08	54.47	5.33	14.15	TD-08	48.07	5.68	16.97	TM-08	46.58	4.75	13.66
RB-09	52.11	6.96	19.14	TD-09	51.02	6.29	16.55	TM-09	43.80	3.66	10.92
RB-10	48.34	6.69	15.96	TD-10	46.41	8.22	22.47	TM-10	59.78	4.26	16.70
RB-11	48.05	8.50	22.93	TD-11	51.83	6.20	19.02				
RB-12	52.26	7.33	19.13	TD-12	57.97	5.73	19.52				
RB-13	55.12	7.54	22.10	TD-13	46.99	5.19	14.75				
RB-14	52.16	7.41	19.06	TD-14	53.72	6.80	20.33				
RB-15	58.03	7.19	16.74	TD-15	58.56	7.62	24.65				
RB-16	58.72	7.55	18.71	TD-16	67.27	6.04	23.37				
RB-17	55.24	7.98	19.70	TD-17	52.67	5.64	17.00				
RB-18	50.45	6.53	16.64	TD-18	51.36	6.16	16.14				
RB-19	50.92	6.69	14.86	TD-19	55.89	6.84	18.76				
RB-20	49.57	6.98	15.84	TD-20	53.15	5.94	16.34				
RB-21	52.85	9.23	21.16	TD-21	57.34	4.75	18.09				
RB-22	47.63	7.61	17.13	TD-22	54.21	5.37	17.60				
RB-23	47.24	6.66	14.01	TD-23	53.80	7.17	20.33				
RB-24	57.06	6.28	15.98	TD-24	46.29	5.08	16.67				
RB-25	57.63	6.90	17.64	TD-25	49.55	5.43	17.70				
RB-26	40.31	6.55	13.58	TD-26	52.01	4.38	18.67				
RB-27	56.57	6.05	16.39	TD-27	59.72	5.47	23.67				
RB-28	37.01	3.99	9.11	TD-28	57.10	4.71	21.41				
RB-29	50.23	7.14	14.14	TD-29	55.50	6.48	22.04				
RB-30	49.52	7.10	17.66	TD-30	65.59	6.75	23.16				
RB-31	55.17	7.09	21.78	TD-31	54.93	8.57	24.49				
RB-32	47.52	7.38	16.42	TD-32	55.47	7.29	21.55				
RB-33	54.65	6.62	14.76	TD-33	46.34	6.72	17.62				
RB-34	52.45	7.00	14.34	TD-34	46.87	6.64	17.98				
RB-35	58.21	7.40	17.46	TD-35	65.89	5.56	21.55				
RB-36	55.46	6.88	18.11	TD-36	57.72	5.63	20.76				
RB-37	41.25	4.68	13.26	TD-37	63.92	5.58	19.82				
RB-38	41.43	6.07	13.30	TD-38	48.79	4.09	16.58				
RB-39	51.30	5.89	14.85	TD-39	59.94	5.83	22.78				
RB-40	52.94	6.60	13.89	TD-40	58.69	5.84	20.84				
RB-41	56.87	6.27	14.49	TD-41	58.62	6.36	21.15				
RB-42	56.76	7.75	18.07	TD-42	55.26	7.54	22.91				
RB-43	50.51	7.26	14.61	TD-43	60.72	6.69	22.98				
RB-44	50.78	7.28	15.16	TD-44	57.89	7.00	21.12				
RB-45	56.20	8.15	16.91								

RB: brown coating (*revêtement brun*); TD: finger flutings (*tracés digitaux*); TM: modern traces (*traces modernes*).

### Spatial distribution of the panels

**Fig 9:**
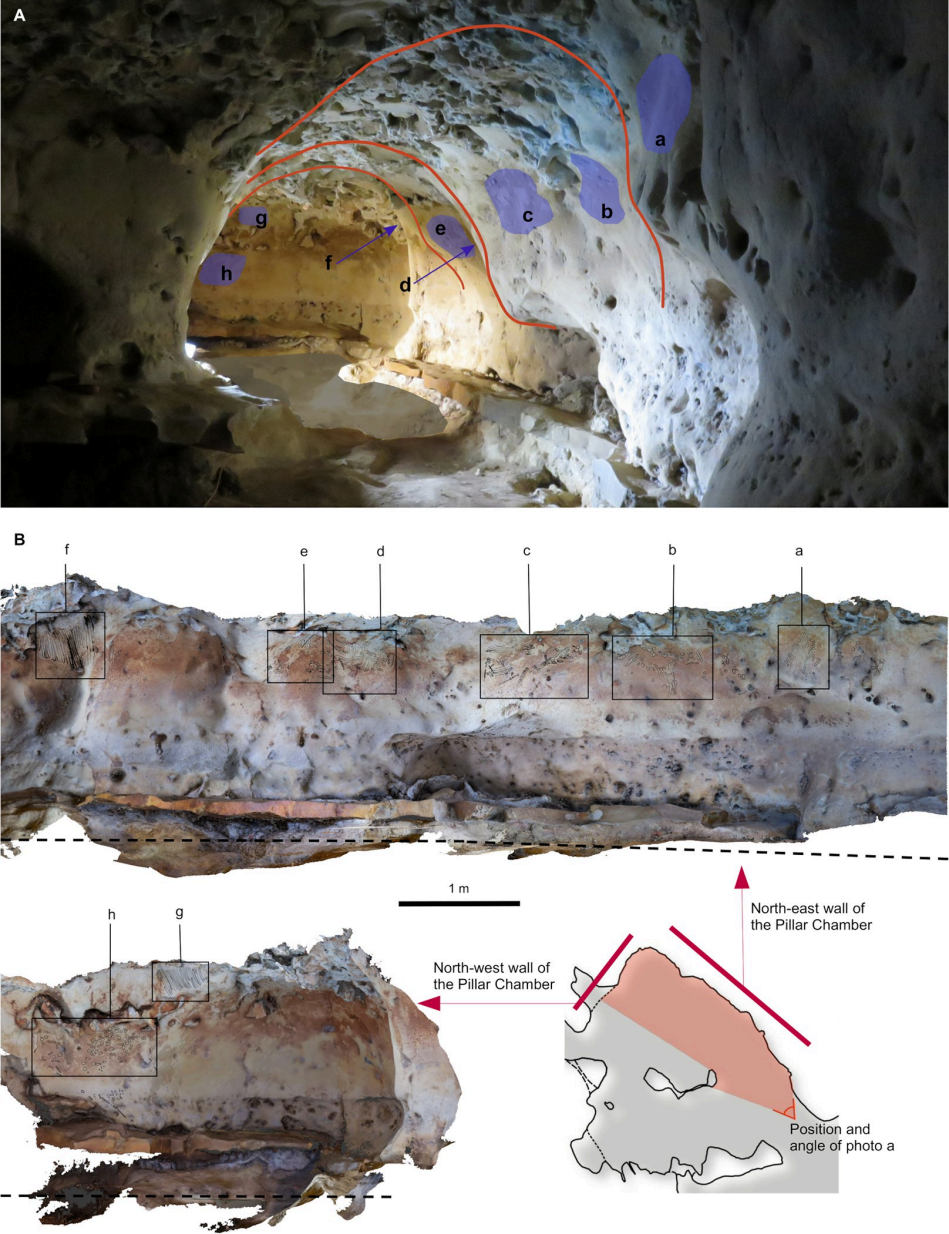
Spatial organisation of the marked panels in the Pillar Chamber. A. View of the Pillar Chamber from the entrance, showing the location of panels with markings. Sections and ridges of the ceiling are indicated by red lines. Numbered panels are indicated by blue areas or arrows. The horizontal grey area on the ground is at the altitude (50.05 m NGF) of the top of the very compact layer 3, in front of the last five digital trace panels. B. Orthophoto of the north-west and north-east walls of the Pillar Chamber, with the location of the panels with plots. The dashed line represents the probable ground level. a: Entrance Panel; b: Fossil Panel; c: Linear Panel; d: Undulated Panel; e: Circular Panel; f: Triangular Panel; g: Rectangular Panel; h: Dotted Panel (Photogrammetry Y. Egels).

All the anthropogenic traces presented here were made on those parts of the tuff wall covered with a thin layer of chemically altered material (Fig 9), and always above the horizontal overhang attributed to prolonged ponding of water. Below this overhang, the tuff is affected by numerous dissolution niches but has no observable alteration layer covering the surface. The alteration layer (maximum thickness 3 to 4 mm), present from above the overhang to the base of the siliceous ceiling (S1 Fig 4 and 5), is still plastic today, and consists of two superposed layers. The outer layer is a brownish film composed of very fine quartz grains and small shell fragments agglomerated by very small amounts of clays; the inner yellow layer has a composition closer to that of the very light yellow tuff bedrock. This altered layer is missing over large areas for various reasons, e.g. rubbing by animal fur in the lower and middle parts of the wall and especially in the upper parts, water condensation and drip erosion.

The eight main panels containing the anthropic engravings appear on the upper part of the wall, and with two exceptions (panels g and h, Fig 9) are all composed of finger flutings. The first six panels (a to f) are at an average height of 1.50 to 1.70 m above the probable Neanderthal floor. The majority of the traces on these panels were made by fingers laid flat (based on the characteristic width of the traces, the presence of lifted particles on edges and ends, as described in S8 Fig), while a few rare traces appear to have been made by a finger on edge (on the side). Panel g is located immediately below the base of the ceiling (at a height of 1.80 m), in the terminal part of the Upper Turonian, very rich in quartz and fossil fragments. It is completely devoid of any remains of an altered surface layer and the tracings do not seem to have been made with a flat finger. Panel h is only 1 m high, much lower than the other seven panels and it shows only traces of punctual removal (S1 Video).

The sediment composition of the yellow tuff making up the walls, and its altered surficial layer, played an essential role in the structure and appearance of the finger flutings, the characteristics of which are very different from tracings made on a clay support. The digital tracings have cut into the altered film, stopping at the more resistant tuff. The nature of the materials that form this surficial film undoubtedly gave, at the time of the drawing, a smooth and regular groove. Some tracings show a well-defined start and end and have very thin ridges along both edges. Water condensation and drip erosion as well as the air circulation within the cavity have locally damaged the altered surface, and so lines with perfectly fine, clean boundaries are now only exceptional. The dotted lines drawn on the measurements of the finger tracings correspond to these imprecise limits, the details of which are often difficult to define.

### Description of the panels

#### Entrance Panel (a)

This set of 36 digital traces (S9 Fig) was made on a flat surface of 50 by 50 cm with a thin layer of wall alteration. The traces are roughly parallel and oriented from top right to bottom left. Some of these traces are simply the result of impact. The length of the lines varies from 5 cm to just under 10 cm (the latter for traces 10 and 12); width is about 1.5 cm. The diameters of the impact traces (3, 4, 14, 18) correspond to the typical width of a finger. In contrast to the lower extremities of the engravings, the upper extremities are well defined here, reaching or even exceeding the second film of the wall alteration. A few traces of animal scratches exist, but only in the right part of the panel (coloured in blue in S9 Fig), the edge of line 12 has clearly been overimpressed by three short younger scratches. The surrounding surface shows only a thin layer of wall alteration; the position of the panel close to the entrance of the room may explain likely significant erosion by air circulation in the cave.

#### Fossil Panel (b)

The Fossil Panel (S10 Fig), 80 cm long, contains a set of eight clearly visible finger traces (1, 2, 9, 11, 12, 13, 24, 42), and, at the bottom, a set of 21 finger traces (from 63 to 83), only slightly accentuated (contours noted in dashes). These ~10 to 13 mm wide lines are often oriented obliquely, either to the right or to the left. This panel shows above all numerous grouped punctuations, on the right from trace 43 to 62, on the left from trace 3 to 8 and from trace 68 to 74, finally all along the section of a large fossil 41 cm in length (*Bimorphoceramus turonensis* Tröger 2006). These mainly circular punctuations vary from 12 to 22 mm diameter; some are more oval in shape. To the right, intersecting the fossil shell in the bedrock, an isolated finger fluting (42) is recorded as a perfectly preserved groove with a very narrow raised bead-like ridge along each side of the trace. Animal traces are rare on this panel, but there is a one very well-preserved trace composed of four claw marks from a currently unidentified species [87]. There are no modern anthropogenic traces. The structure of this panel seems linked to the form of the longitudinal section of the bivalve fossil. It could, perhaps, have attracted the attention of the authors of these traces; all around the hinge of the shell, on both sides of the thickest part, we find numerous impacts and lines which seem to underline its presence.

#### Linear Panel (c)

**Fig 10:**
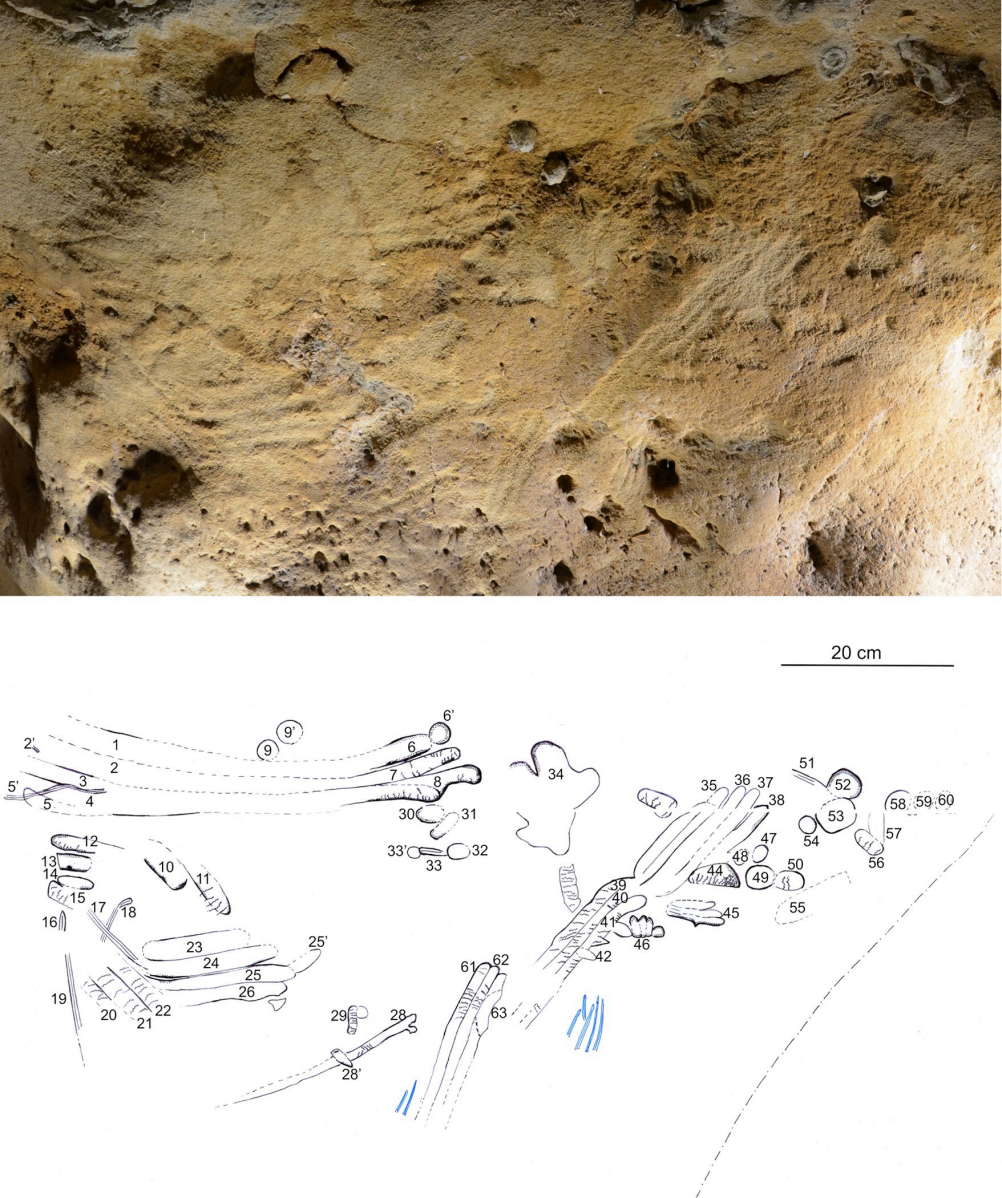
The Linear Panel. The limits of the finger flutings are shown in black. When the edges are well cut, the line is thicker. When the line is not clearly legible, the line is dashed. Animal tracks are in blue. This legend also applies to all other panels. The survey (J. Esquerre and H. Lombard) gives the numbering of the traces.

This large panel, 1.50 m long and 0.50 m high, is made up of 63 ancient anthropogenic traces (Fig 10). The right half of the panel consists of a main motif that begins with the tracings of four fingers (35 to 38) descending 45° to the left; a second stage only shows the traces of three fingers (39 to 41). Further down, a third stage includes the marks of three fingers (61 to 63) traced more vertically. Each of these three traces measures 20 cm in length, with digital widths of ~10 to 12 mm (35 to 38 and 61 to 63). At the top and to the right of this structure, there are 13 punctiform traces (47 to 60) well grouped, with variable diameters from 15 (47) to 52 mm (52). Animal traces are limited to two small groups of two and four traces, and there are no modern anthropogenic traces. The left half of this panel includes 34 lines (1 to 34) in several mostly horizontal sets. The three highest horizontal traces (6 to 8) are 40 cm long. Below, other traces, in the same direction, are shorter, from 15 cm to 10 cm long. This part of the panel has very few punctuation marks, but also contains, at the bottom left, four ancient traces (16 to 19) from 2 to 10 cm in length, of V-shaped sections. This part of the panel is marked by some erosion of the lines, unlike the right part, which is much more intact.

#### Undulated Panel (d)

**Fig 11:**
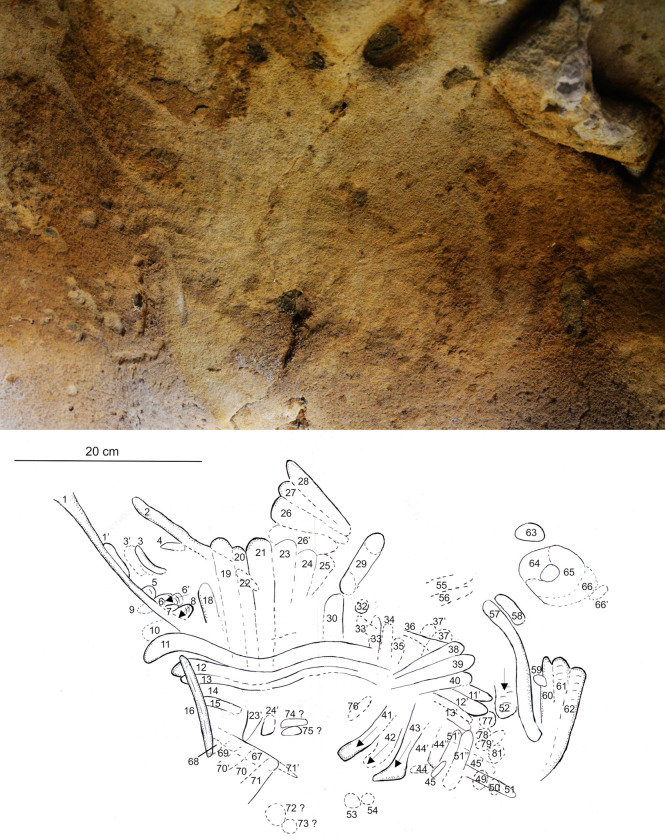
The Undulated Panel. The survey (O. Spaey and G. Alain) gives the traces numbering. The arrows indicate the direction of the passage of the finger.

This panel is 70 cm long and 50 cm high (Fig 11). It includes 84 traces of variable lengths, from 33 to 10 cm. Many of the shorter traces are grouped together and are sometimes associated with dots (44 to 51). There are also somewhat scarce and isolated dots that complete the set (53, 54, 63). This panel does not include any animal or modern anthropogenic traces. The altered surface film on the tuff lacks on this panel, presumably because of erosion. The three long longitudinal traces (11, 12 and 13), probably made from left to right, show double undulation. At the top left of these traces are two long subparallel traces (1 and 2). The upper trace (2) seems to have been started at its highest point, but this is less obvious for the other trace (1) that widens upwards. Associated with these two sets is a series of finger traces (18 to 25) that appear to be oriented toward a wavy axial band. Finally, there are two finger traces that may play a complementary role by seeming to close the entity, one located in front (16), almost vertical, and the other, slightly longer and curved (57). The five lines (1, 2 and 11 to 13) seem to give this panel a kind of unity. It seems more deliberately composed than the previous examples.

#### Circular Panel (e)

**Fig 12:**
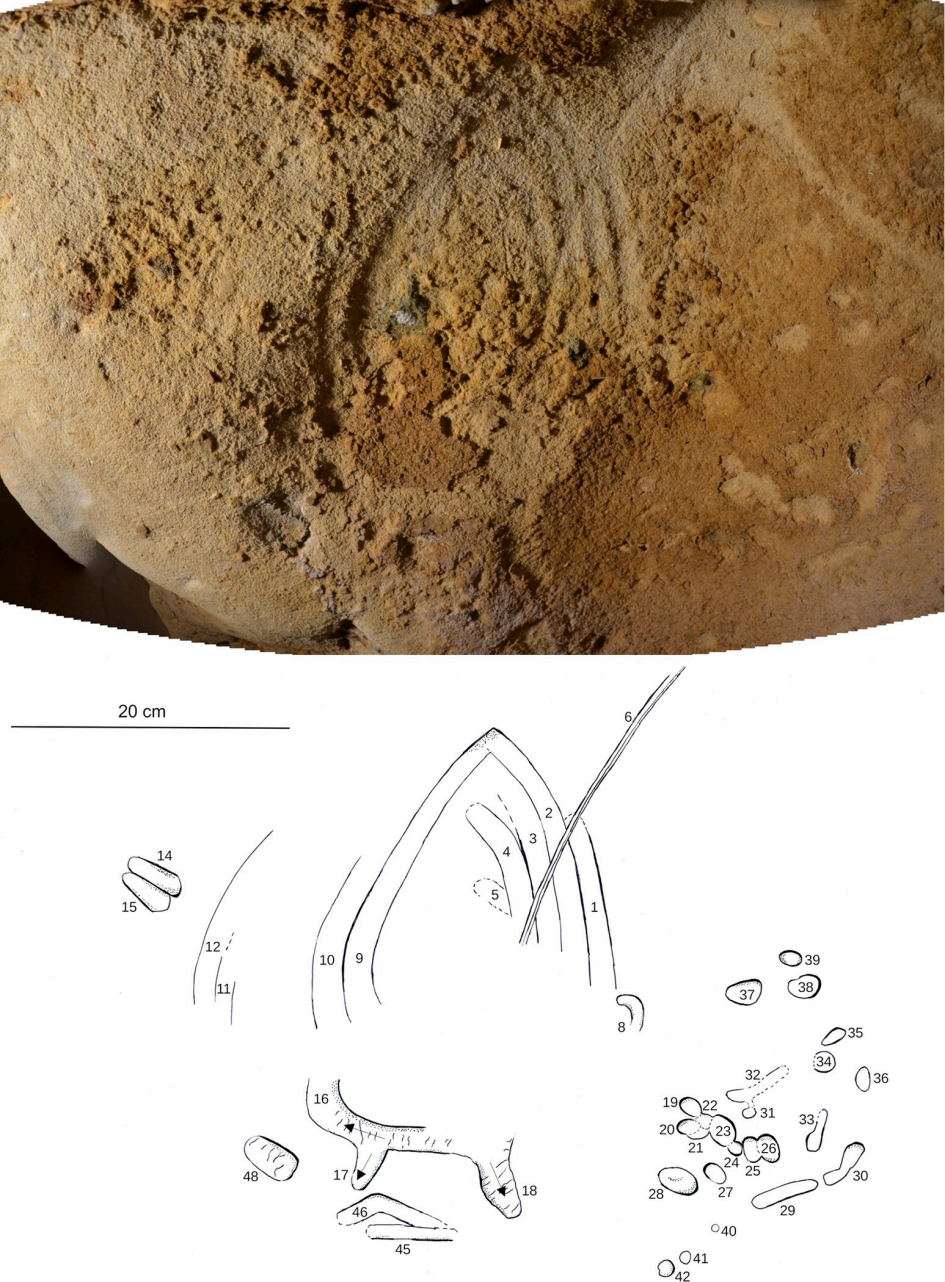
The Circular Panel. The survey (O. Spaey and G. Alain) gives the numbering of the traces. The arrows indicate the direction of the traces. The alteration of the central lower part is very strong and worrying. S11 Fig shows the Undulated Panel and the Circular Panel together.

This panel (Fig 12) measures 50 cm wide by 60 cm high. It is composed of two sets: the main centre set, comprising 17 finger tracings (1 to 17) and, on the right, a set of 31 dots (19 to 42). The longest of the lines (9) measures 25 cm in length and 2 cm in width. The lines (2 and 9), drawn from top to bottom, have a common origin, and are curved to give an ogive. This deliberately closed, and therefore composed, character leads to a kind of pattern. The surveys of the curves 11 and 12, of the same shape as 9 and 10, may be part of the same set. Below, there is a very strong horizontal trace (16) whose direction, from right to left, is indicated by the lifting of small scales giving the direction of movement of the finger (S8 Fig). This large trace 16 is associated with two vertical traces 17 and 18. Finally, to the right of the main part of this panel, another set of traces (from 19 to 42) is made up of dots arising from impacts of items of various shapes and sizes. The main part of this panel, remarkable for its ogival shape, seems to be the result of a deliberate composition. This panel is immediately adjacent to Undulated Panel (d) and one may wonder if the two panels together do not constitute a single entity (S11 Fig and S1 Video).

#### Triangular Panel (f)

**Fig 13:**
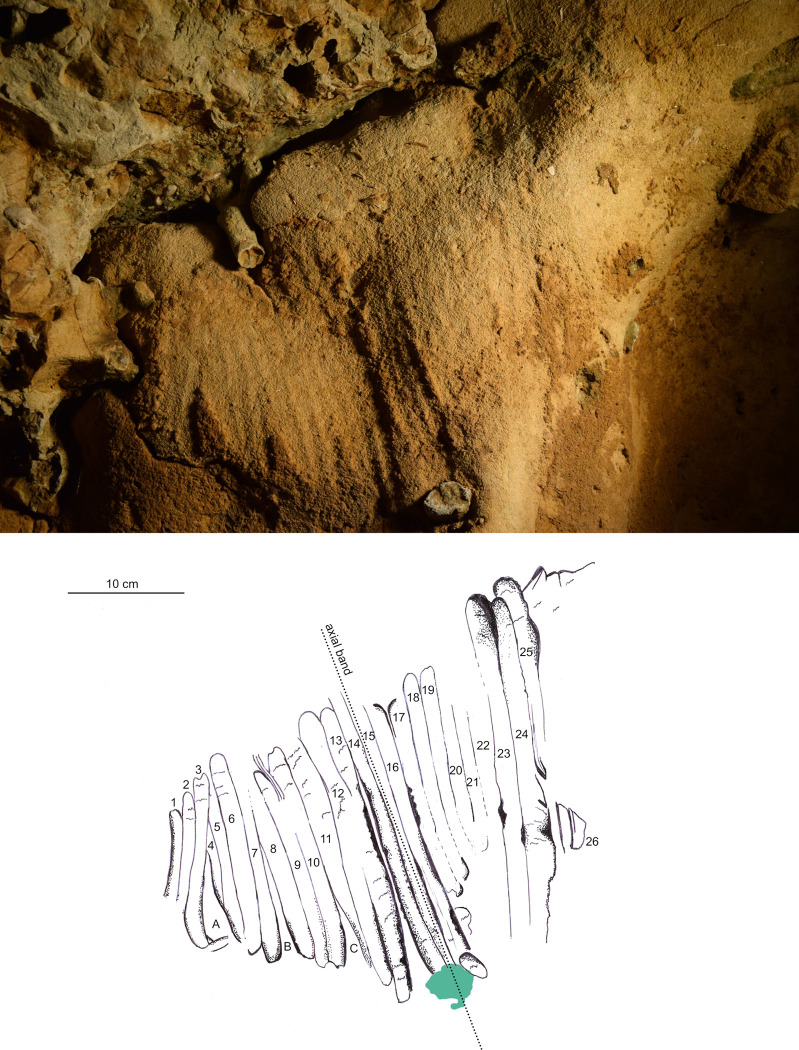
The Triangular Panel. The survey (M. Calligaro) gives the numbering of the traces. The green zone corresponds to the surface of the break of a natural cylinder of chert in its natural place. There are complements in S12 Fig about that panel.

The triangular Panel (Fig 13 and S12 Fig) is 60 cm wide and 50 cm high. It is composed of 25 finger flutings, made from top to bottom, parallel to each other. Their length varies, from 8 cm for the shortest (1), to 32 cm for two or perhaps three very long finger flutings on the right edge of the panel (23, 24 and 25). The width of these engravings is also variable: from less than 1 cm wide (15), to more than 1.5 cm (24, 25).

This panel is located immediately at the entrance of a large, regular alcove. It is placed on an inverted isosceles triangular surface (base at the top), bounded by the space that marks the change from the top of the tuff wall to the siliceous ceiling of the cavity. The two equal sides of the triangle correspond to the vertices of two slight recesses in the wall, which give rise to a triangular surface that is not completely flat. The main vertex of the triangle, at the bottom of the panel, is highlighted by the presence of a small cylindrical chert embedded in the wall. The whole of this triangular surface must have been completely covered by a surface wall film, 3 to 4 mm thick, before the tracings were made.

Although the tuff here is rich in fine quartz sand and small shell fragments, both the initiation and termination of the traces are often visible. The grooves made by fingers have not completely removed the thick double film presumed to have covered the entire surface. Along the axis of the triangle (Fig 13), from its base (top) to the summit chert (bottom), two very narrow parallel bands of altered surface covering, widening slightly as they approach the chert, have been preserved, separated by three very regular finger lines (14 to 16). In the left part of the isosceles triangle, thirteen parallel finger lines (1 to 13) have removed the surface coating, but the original coating has been preserved intact in three triangles (A, B and C). The oblique bases of these three triangles are connected to one of the two equal sides of the isosceles triangle. On the right side of the axial band of the triangle, the parallel finger traces (17 to 24) remain visible, but erosion, probably due to air streams, seems to have removed at least part of the altered surface film.

Detailed examination of that preserved area leads us to suggest that the author of this drawing set out to use totality of this triangular surface, our hypothesis is that this preservation is in no way by chance but a deliberate intention A model giving curvatures of the surface of the left part of the panel is presented on S12D and S12E Fig. Around the largest triangle A, we see that concave curves become orange and red allowing us to make hypothesis on the work of the finger. Each strong pressure episode (red) is followed by a lighter (orange) one because the finger moves irregularly forward drawing the border of the triangle. The yellow border around the red band is asymmetrical: on the side of the triangle it is narrow, the curvature being pronounced to better delineate the side of the triangle; on the other side there is a wider slope. These characteristics are repeated on the other two triangles B and C, but are less clearly visible because of the wall surface alteration.

#### Rectangular Panel (g)

This panel is 30 cm long and 25 cm high (S13 Fig). It consists of 22 anthropogenic traces drawn using a tool or with the edge of a finger, and seven digital traces made with a flat finger (1 and 2, 18 and 19, 24, 27 and 29). The traces of the first group have V-shaped cross-section while the others have U-shaped cross-section. The lines of this panel are all almost vertical and sub-parallel, forming a slight fan shape. The lengths of all these tracks are between 6 and 20 cm, the widths of the V-shaped tracks are between 2 and 6 mm. No animal or modern anthropogenic traces are visible on this panel which, as with the Triangular Panel f, is located on the highest and terminal part of the tuff wall; at this point the wall is very rich in fine quartz sand and small angular fossil fragments. Statistical analysis (Fig 8A) of this panel, based on width, angle of incision and depth of traces, showed a very large difference between these traces and those of the Circular and Triangular Panels. As mentioned in LDA processing of the morphometric characteristics of the experimental marks, the experimentation did not allow to determine the tool used for the realization of these traces. Some rares traces on the left and right, rather U-shaped, could be due to digital tracings.

#### Dotted Panel (h)

**Fig 14:**
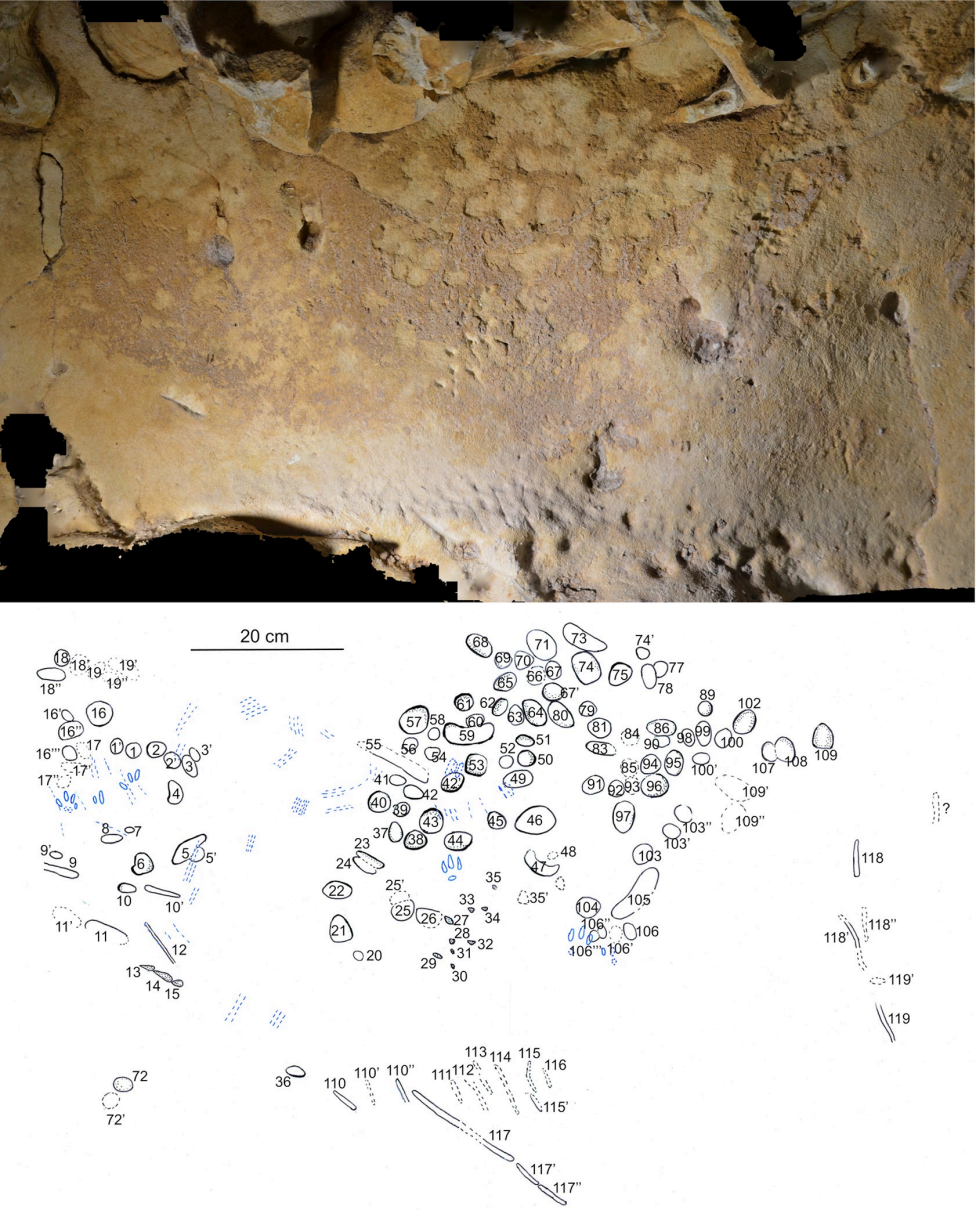
The Dotted Panel. The survey (O. Spaey and G. Alain) gives the numbering of the traces. Animal marks are in blue. Traces 13 to 15 and 27 to 35 are modern anthropic traces made with metal tool.

This panel is one meter long and 60 cm high (Fig 14) and contains a total of 119 traces. There are 110 old anthropogenic traces, including a large number of circular traces 12–15 mm in diameter (50 to 96), some more oval, reaching 20–25 mm (97, 46), some elongated (5, 59, 71, 73, 105) up to 8 cm (106). Finally there is a series of oblique lines varying in length from 3 to 12 cm (110 and 117), downwards and to the right, mainly in the lower part of the panel. Recent anthropogenic traces were probably made in 1912: these are small triangular traces forming a small cloud (27 to 35) and a series of three aligned, closely spaced traces (13 to 15), all made with a pointed metal tool. Animal marks are quite numerous in a central vertical strip of the panel, and these may have destroyed any anthopogenic traces in this strip. The ancient anthropogenic traces have removed the outer brown altered layer on the original wall surface, without destroying the underlying yellow layer; they have not reached the tuff, unlike the modern metallic impacts. They seem to have been made by finger contact, which could, in some cases, slide a little on the surface. Some of the circular impacts have partly straight edges, but it is difficult to determine whether a finger nail or tool was used in when creating them.

### Dating

The ages of the different lithostratigraphic units were investigated using Radiocarbon and OSL dating.

#### Radiocarbon ages

The bones chosen for dating had nitrogen levels between 0.55% and 2% and none had excessive contamination levels. All the AMS dates (Table 4) gave ages close to or above 40 ka BP except those where the bones were not treated by ultrafiltration. This is undoubtedly the case for the dates Lyon-6962(SacA-19431) and Lyon-9087(SacA-28354) and, perhaps, for the conventional ages of Gif-4383 and Gif-4447. Since these ages were obtained, techniques for the separation and purification of certain amino acids, especially hydroxyproline, have been developed. This amino acid, specific to mammalian collagen, cannot be derived from later carbon contamination. In the future, it would be interesting to redate some samples of the series [88]. The four ^14^C ages of the bone fragments of U4 in LRC II, and of U4, U2 and U1 in LRC IV, gave non-finite ages (i.e., older than about 45 ka BP) (Table 4). The only finite ages given by radiocarbon for ultrafiltered samples are clustered around 40 ka BP. These ages are close to the saturation limit of the technique and the comparison with independent OSL ages (see below) suggests that these radiocarbon ages underestimate the true age of the samples. Therefore, these ages are omitted from the subsequent chronological modelling.

**Table 4:** ^14^C ages.

Locus/Square	Level	Unit	Dated material	Laboratory code	Date year	Measure	Ultra-filtration	Age ^14^C BP	Calibrated age (OxCal v4.1.7)
LRC I Ext.	up	U3	Bone chip of LM (0,4 g)	Lyon-6962 (SacA 19431)	2014	AMS	no	34790 ±870	From 41745 to 37892 BP.
LRC I/Y08	mid	U4	Basal part of a Bison axis	Lyon-11276 (SacA37649)	2014	AMS	yes	40700 ±1400	From 44675 to 43826 BP.
LRC I/I14	inf	U4	Bone chip of MM	Lyon-7864 (SacA23350)	2014	AMS	yes	38060 ±940	
LRCI/C12	inf	U4	Bone chip of LM	Lyon-11273 (SacA37646)	2014	AMS	yes	41500 ±1500	From 45361 to 44542 BP
LRC I/Q17	3	?	Bone chip of LM	Lyon-7865 (SacA23351)	2014	AMS	yes	>45000	
LRC I int.	mid	?	Several small bone chips	Lyon-6961 (SacA19430)	2014	AMS	yes	>40000	
LRC I S hyène	1	?	Tooth Equus	Lyon-10161 (SacA 32828)	2012	AMS	yes	>40000	
LRC II	7 (inf cut)	U4	Bone chip of MM	Lyon-6963 (SacA 19432)	2014	AMS	yes	>40000	
LRC II/H12	8 (inf cut)	U4	Bone chips of MM	Lyon-9086 (SacA 28353)	2011	AMS	yes	>45000	
LRC III/J6	XIII	U4	Bone chip of LM	Lyon-10163 (SacA32830)	2012	AMS	yes	>40000	
LRC III/I13	XIII	U4	Bone chip Ofb LM	Lyon-10162 (SacA32829)	2012	AMS	yes	>45000	
LRC IV/S.1/O9	7a	U2	Marmota bone	Lyon-9087 (SacA28354)	2011	AMS	no	27920 ±310	From 32998 to 31436 BP
LRC IV/Q2	13b	U4	Bone chip of MM	Lyon-7863 (SacA23349)	2013	AMS	yes	>45000	
LRC IV/Q3	13d	U4	Bone chip of MM	Lyon-7862 (SacA 23348)	2013	AMS	yes	>45000	
LRC IV/Q3	15	U4	Bone chip of MM	Lyon-9088 (SacA 28355)	2011	AMS	yes	>40000	
LRC IV/P3	16a	U4	Bone chip of MM	Lyon-9089 (SacA 28356)	2011	AMS	yes	>40000	
LRC III	XIII	U4	Complete rib of LM	Gif-4384	1980	Classic	abundant collagen	≥45000	
LRC II	7	U4	Complete rib of LM (0,8 g)	Gif-4383	1980	Classic	abundant collagen	≥32100	
LRC I Ext.	mid	U4	Ulna of Bos/Bison	Gif-4447	1980	Classic	abundant collagen	≥38400	

In LRC I, the three main layers are referred as lower, middle, and upper (Figs 4 and 6). The stratigraphic units of the outer layers and those connecting the Lemmings Chamber to the outside are noted as U5 to U1. Inside the cave, far from the entrance, the layers cannot be correlated to these stratigraphic units, so their units are not determined. The 4^th^ column gives the sample codes. SM: Small mammal, MM: Medium mammal, LM: Large mammal.

#### OSL ages

The doses and burial ages from single-grain quartz and K-rich feldspar aliquots show that the quartz OSL signals from Units 1–5 in LRC I and LRC II, and from Units 1–4 in LRC III and LRC I, were very likely to have been well bleached before burial and can thus be used to provide accurate burial ages (see S2 Text). In general, the OSL ages increase with decreasing elevation and with deeper deposition units. Table 5 summarises the multi-grain OSL results from quartz. Tables SI.5 and SI.7 in S2 Text summarise the multi-grain feldspar and single-grain quartz results, respectively.

**Table 5:** Summary of multi-grain quartz (Q) OSL results.

Lab. code	Fig. code	Locus	Unit	Level	Elevation	w.c.	Dose rate (Q)			Dose (Q)			n	In sat	Sample age (Q)			Bayesian age (Q)		
					[m]	[%]	[Gy ka^-1^]			[Gy]				[%]	[ka]			[ka]		
187301	1	LRC I-d	1	3	54.95	17	1.87	±	0.08	79	±	3	19	0	42	±	3	43	±	3
187302	2	LRC I-d	1	4	54.55	17	2.74	±	0.12	129	±	6	35	0	47	±	3	44	±	3
187303	3	LRC I-d	1	6	54.10	17	2.11	±	0.10	97	±	6	20	0	46	±	4	46	±	3
197332	4	LRC I-c	1	a5	51.28	17	1.92	±	0.09	105	±	3	34	0	55	±	3	54	±	3
197328	5	LRC I-c	1	a1	51.10	17	1.93	±	0.09	97	±	3	19	0	50	±	3	54	±	3
197333	6	LRC I-c	1	a6	50.97	17	1.93	±	0.09	101	±	4	35	0	53	±	3	55	±	3
197340	7	LRC I	E.C.	-	50.78	25	1.42	±	0.08	449	±	69	4	69	>317				-	
197338	8	LRC I	E.C.	-	50.66	25	1.37	±	0.08	228	±	19	40	17	>167				-	
197339	9	LRC I	E.C.	-	50.57	25	1.30	±	0.09	320	±	11	33	25	>247				-	
167806	10	LRC I-a	2	up	50.54	15	2.29	±	0.09	132	±	6	22	0	58	±	4	58	±	3
167809	11	LRC I	I.C.	-	50.20	25	1.63	±	0.09	321	±	26	14	32	>197				-	
167817	12	LRC I	I.C.	-	50.20	25	1.52	±	0.09	311	±	49	14	42	>204				-	
167818	13	LRC I	I.C.	-	50.20	25	1.52	±	0.09	449	±	22	13	42	>295				-	
227801	14	LRC I	4	mid	50.05	17	2.63	±	0.12	154	±	6	23	4	58	±	4	62	±	3
167805	15	LRC I-a	4	mid	50.04	17	2.06	±	0.10	137	±	6	21	8	66	±	4	62	±	3
187312	16	LRC I-a	2	up	49.79	15	2.27	±	0.11	155	±	4	34	3	69	±	4	64	±	3
227802	17	LRC I	4	mid	49.71	17	2.32	±	0.11	146	±	5	23	4	63	±	4	65	±	3
187311	18	LRC I-a	2	up	49.55	15	2.12	±	0.09	135	±	5	38	5	64	±	4	67	±	4
161267*	19	LRC I-b	4	mid	49.40	17	2.06	±	0.09	163	±	7	24	-	79	±	5	68	±	4
167812	20	LRC I-b	4	mid	49.40	17	1.75	±	0.09	134	±	8	22	4	76	±	6	68	±	4
187321	21	LRC II	3	C	47.59	15	1.28	±	0.06	112	±	3	34	0	88	±	5	85	±	4
187322	22	LRC II	3	D	47.18	15	1.28	±	0.06	122	±	3	33	0	95	±	6	87	±	4
187320	23	LRC II	3	E	47.09	15	1.89	±	0.08	159	±	5	35	0	84	±	5	87	±	4
187323	24	LRC II	5	G	46.90	18	1.32	±	0.07	159	±	6	34	3	121	±	8	88	±	4
187310	25	LRC II	3	3	46.76	15	1.49	±	0.07	132	±	4	23	0	88	±	5	89	±	4
187306	26	LRC II	3	3	46.64	15	1.39	±	0.06	121	±	4	33	6	87	±	5	90	±	4
187309	27	LRC II	3	5	46.03	15	1.10	±	0.08	113	±	4	23	4	103	±	9	94	±	5
187305	28	LRC II	3	3	45.96	15	1.51	±	0.06	140	±	5	33	3	92	±	5	94	±	5
181362*	29	LRC II	4	6	45.70	17	1.77	±	0.08	138	±	4	33	-	78	±	4	96	±	5
187308	30	LRC II	4	6	45.70	17	1.43	±	0.07	142	±	3	47	0	99	±	6	96	±	5
207307	31	LRC II	4	7	45.52	17	1.61	±	0.08	156	±	4	66	6	97	±	6	97	±	5
181361*	32	LRC II	4	7	45.50	17	1.64	±	0.07	156	±	3	33	-	95	±	5	97	±	5
187307	33	LRC II	4	7	45.18	17	0.74	±	0.04	243	±	10	25	17	>328				-	
187304	34	LRC II	5	8	45.13	18	1.50	±	0.08	182	±	13	33	8	121	±	11	99	±	5
161263*	35	LRC III	4	7	45.20	17	2.09	±	0.09	135	±	4	24	-	65	±	4		-	
167831	36	LRC IV	1	2	49.80	17	2.52	±	0.12	70	±	3	21	4	27.8	±	1.7	30	±	3
167830	37	LRC IV	1	3	48.20	17	2.38	±	0.11	121	±	4	23	0	51	±	3	51	±	3
161264*	38	LRC IV	1	7a	47.20	17	1.86	±	0.08	104	±	4	27	-	56	±	3	60	±	4
167829	39	LRC IV	1	7b	46.50	17	1.78	±	0.09	115	±	7	24	0	65	±	5	66	±	4
167828	40	LRC IV	2	7c	46.20	15	1.95	±	0.08	134	±	7	24	0	69	±	5	69	±	4
167827	41	LRC IV	2	7d	46.00	15	1.91	±	0.09	137	±	7	23	0	71	±	5	70	±	4
167826	42	LRC IV	2	11	44.90	15	1.61	±	0.08	127	±	6	21	8	79	±	6	76	±	4
161266*	43	LRC IV	4	12cd	44.60	17	1.70	±	0.07	156	±	2	22	-	92	±	5	78	±	4
167823	44	LRC IV	4	12	44.60	17	1.98	±	0.09	148	±	7	21	13	74	±	5	78	±	4
167825	45	LRC IV	4	12	44.60	17	2.12	±	0.12	157	±	11	21	13	74	±	7	78	±	4
167822	46	LRC IV	4	12e	44.50	17	2.18	±	0.10	172	±	7	24	7	79	±	5	79	±	4
167821	47	LRC IV	4	15	44.10	17	1.98	±	0.09	192	±	11	24	8	97	±	7	81	±	5
167820	48	LRC IV	5	21	43.30	18	1.58	±	0.09	291	±	19	18	31	>184				-	
161265*	49	LRC IV	5	22	42.50	18	1.68	±	0.08	295	±	9	19	-	>176				-	
167819	50	LRC IV	5	22	42.50	18	1.83	±	0.10	281	±	19	18	28	>153				-	

**Fig 15:**
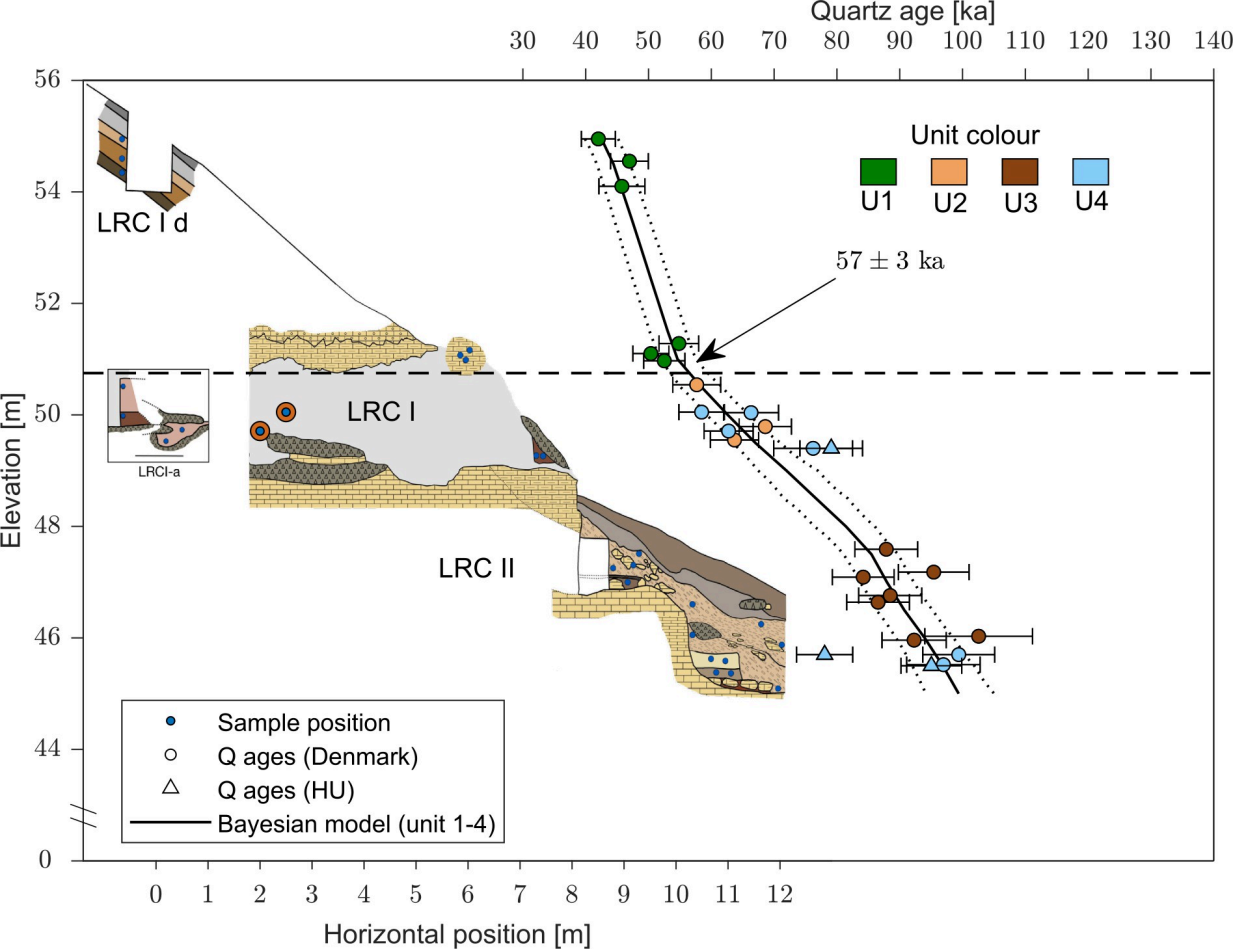
Schematic drawing of LRC I and LRC II lithographic section and multi-grain quartz ages from units 1–4. A Bayesian model (Bacon script [89]) using the elevation as prior and only random uncertainties for the individual ages is shown (black line). Dotted lines show the total uncertainty (including both random and systematic uncertainties) at the 68% confidence interval. The OSL ages measured in Hungary are not included in the Bayesian model. The dashed black horizontal line indicates the cave ceiling elevation in LRC I (50.75 m). The insert shows LRC I-a (see Fig 6) including two samples inside the cave (167805 and -06) and two outside the cave (187311 and -12). LRC I-d includes three samples on a trench on the slope, on top of the cave. The adopted water content is 40±10% of the average measured saturated water content for each deposition unit.

**Fig 16:**
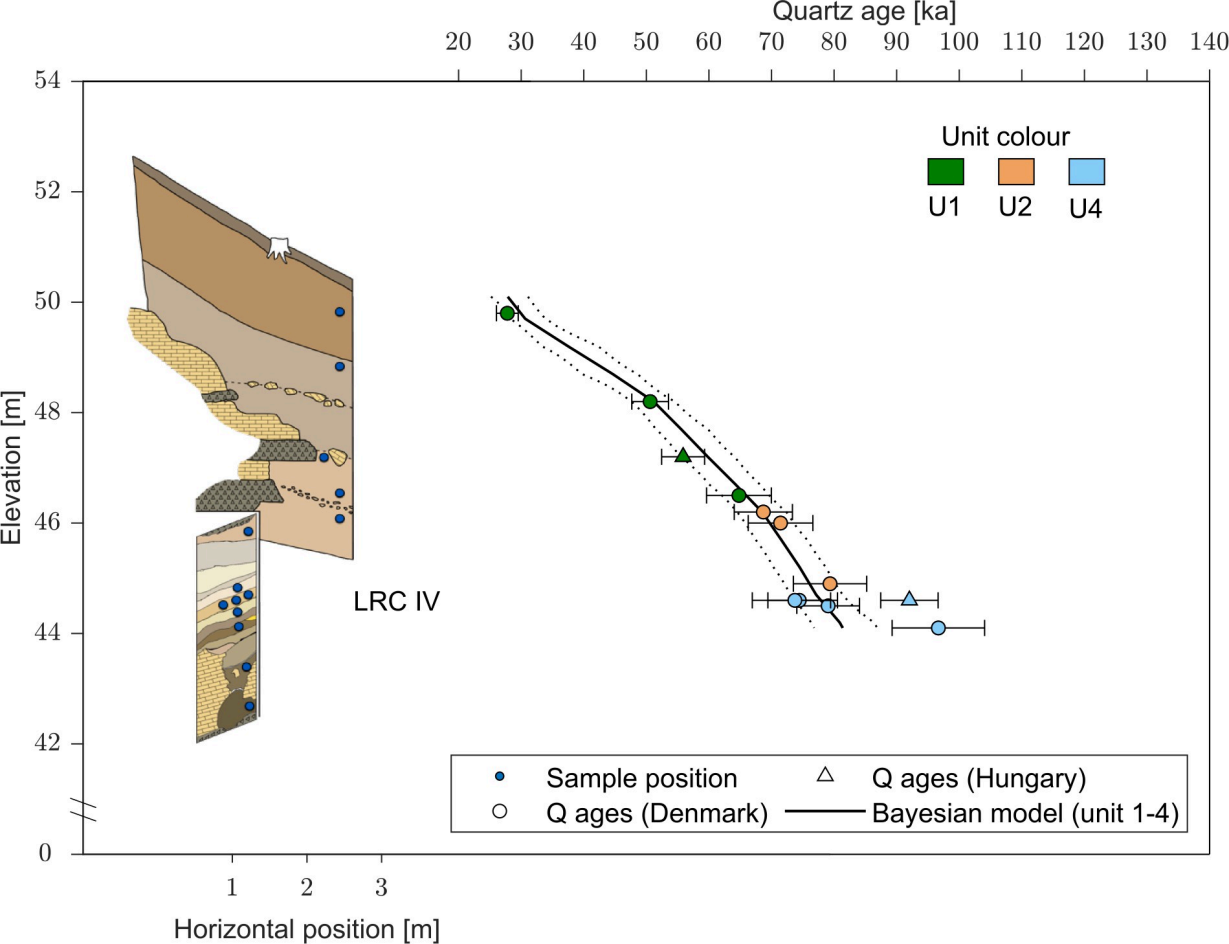
Schematic drawing of LRC IV lithographic section and multi-grain quartz ages from unit 1, 2 and 4. The bottom two samples (167819, 167820) are shown on the diagram but not included in the Bayesian fit (using only random uncertainties; black full line, see S2 Text for further details). Dotted black lines show the total uncertainty (including systematics) of the age model. Uncertainties shown on each data point are at 68% confidence. A water content of 40±10% of the average saturated water content for each deposition unit has been assumed.

Samples prepared and measured in Hungary (Marquet *et al*., 2019) are marked with an asterisk (*). “Fig. code’” refers to the sample codes given in S14 and S15 Figs. Locus “a,b,c,d” refers to locations in LRC I. “Unit” is the lithotratigraphic unit. “I.C.” and “E.C.” represents samples from inner cave of LRC I and around the entrance of LRC I, respectively (not identified with a specific unit). Note that samples 227801 and 227802 were taken from two niches in the entrance of LRC 1 and identified as Unit 4. “Level” is the level from which the sample is collected. “Elevation” is the elevation (m) above sea level of the sample locations (NGF). “w.c.” is the water content employed in age calculations. “Dose rate” is the total dose rate to quartz. “Dose” is the arithmetic average equivalent dose after application of the IQR rejection criterion (see SI.16 Fig and Table SI.5 in S2 Text). All uncertainties are reported at the 68% confidence interval. “n” is the number of aliquots included in the equivalent dose estimation, i.e., the total number of measured aliquots less the aliquots which gave unbounded dose estimates and the number of dose estimates rejected by the IQR rejection. “In sat” is the relative number of aliquots giving unbounded dose estimates. “Age” is the equivalent dose divided by the total dose rate. Samples with more than 15% of the individual multi-grain aliquots appearing to be in saturation are regarded as providing minimum burial ages, i.e., samples 197338–40, 167809,-17-19, 187307, 167820, and 161265. “Bayesian Age” are ages derived from Bayesian modelling (Figs 15 and 16) for altitudes corresponding to sample positions.

All ages derived from inside LRC I (except for samples 227801 and 227802 taken from Niche 1 and 2, see Fig 6) and from unit 5 in LRC IV are considered to be minimum ages due to saturation effects. Bayesian modelling [89] was applied as a function of elevation to those multi-grain quartz ages from LRC I and II unaffected by saturation issues (Fig 15), to determine when the accumulating sediments blocked the entrance. An independent Bayesian model was built using ages from LRC IV (Fig 16), since the elevation difference between LRC IV on the one hand, and LRC I and II on the other, prevents combined modelling; elevation differences are inherent in the development of surficial slope deposits, and are strongly linked to the local morphology [89]. The modelled sediment ages range from 99±5 ka to 30±3 ka (Table SI.9 in S2 Text, based on Table 5).

#### Time constraints for human occupations and closure of the cave

Evidence for the presence of Neanderthals at La Roche-Cotard is associated with the alluvial deposits of U4 (except for two artefacts found in the Pillar Chamber for which we cannot confidently determine the stratigraphic unit). This sedimentary unit, according to the dating results, was deposited between ~99 and ~65 ka (Table 5 and Figs 15 and 16).

In LRC I, the layers containing Mousterian lithic industry (Lower layer) are directly covered by texturally selected fluvial sediments transported into the cave by the Loire River floods (Middle layer); the modelled ages for this Middle layer are between 68±4 and 62±3 ka. At LRC II, ~2 m below the cave entrance, the presence of Neanderthals (attested by a Levallois industry) on a sandy beach of the Loire is dated to 97±5 ka (Bayesian age for layer 7 –alt. 45.50 m NGF). Other sandy flood deposits covering these artefacts are dated to between 95±6 and 88±5 ka (Bayesian ages for layers 6 to 1). In the lower lying shelter, LRC III, the Neanderthals left Discoïd Mousterian artefacts associated with the base of the alluvial deposits (level 7 –alt. 45.20 m NGF, ~65 ka). In LRC IV, they left Levallois Mousterian artefacts, later covered by a sandy river deposit dated to 79±4 ka (Bayesian age for layer 13/ U4 –alt. 44.50 m NGF.). The altitude of the two levels of occupation in LRC I is higher than those of the levels of the three other loci (Figs 15 and 16 and Table 5).

**Fig 17:**
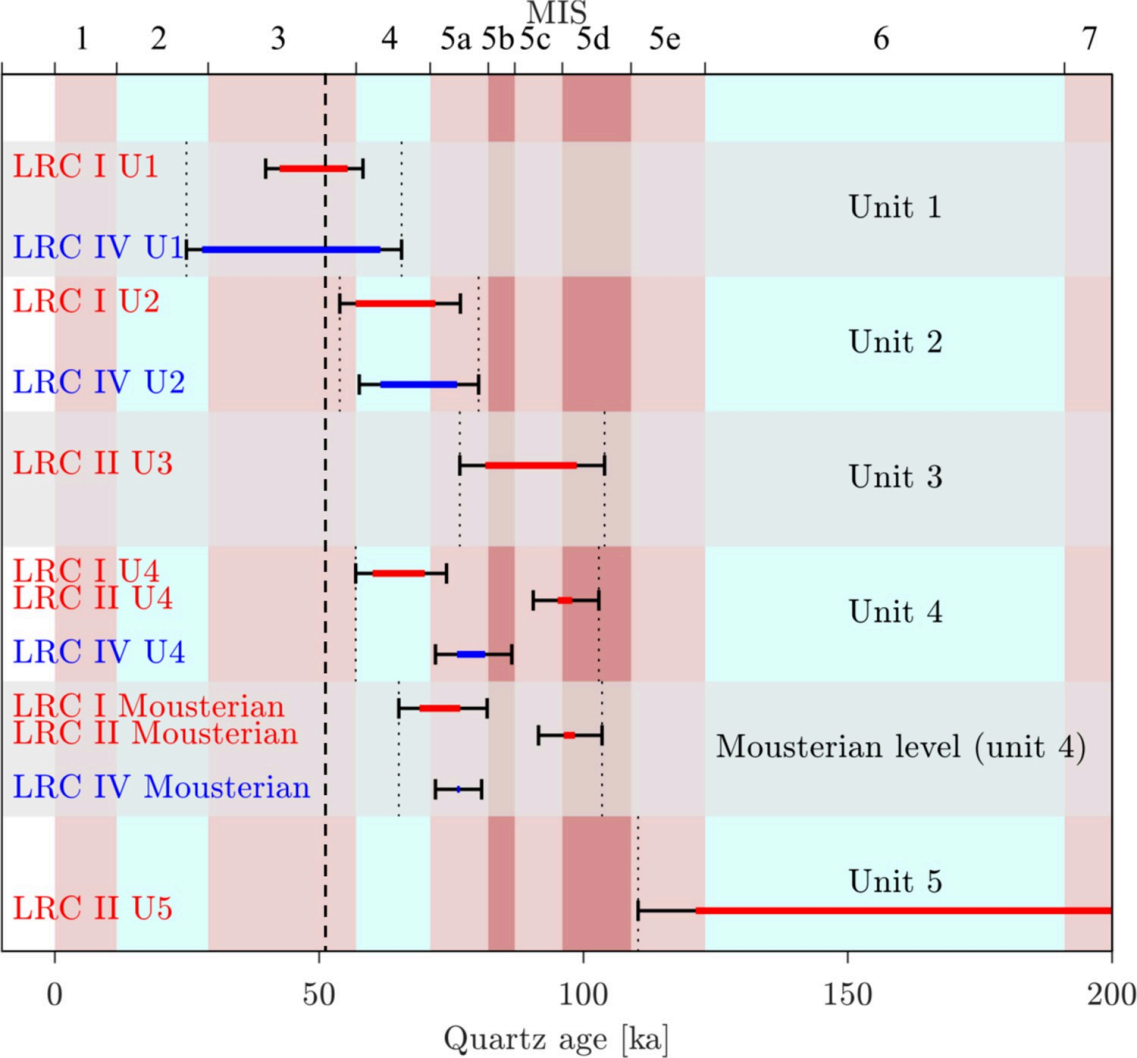
Average limiting ages derived from Bayesian modelled of multi-grain quartz OSL ages using elevation limits for LRC I and II (red lines) and IV (blue lines). The uncertainty (68% confidence level) of these ages is shown with black error bars. The minimum and maximum ages in each unit and in the Mousterian levels are indicated with vertical black dotted lines. For unit 5 (n = 4 samples), the lower age range is derived from sample 187304. No upper age limit is given for this unit as other samples in unit 5 were in saturation. Marine isotope stages 1–7 (MIS) are indicated as red and blue vertical bands (top x-axis). The vertical dashed line indicates the minimum age of the cave entrance closure.

The intercalation of fluvial deposits (U4) and slope deposits with abundant gelifracts (U3) at the same altitude (45–50 m NGF) and over the same time range (MIS 5c to MIS 5a) at LRC II presumably reflects the alternating stadials and interstadials during this period (Fig 17). The small number of lithic artefacts suggests that, during the ~35,000 years of discontinuous Neanderthal presence at the site, human occupations occurred only during short periods, because of highly fluctuating climatic conditions at the regional scale, but also due to the lack of raw material (flint) available at the site for tool production [44, 45, 48].

The geological history (post karstification) of the cave (LRC I) is not fully understood. An important chronological marker is the presence of limited remains of a significant layer of silt from flooding of the Loire (LRC I, Middle layer, U4). This layer (1 to 1.5 m thick according to the discoverer of the cave [43] and also our observations, see S14 Fig / LRCI-b.) directly covers the two Mousterian levels in LRC I. Today, the base is measured at 48.80 m (~76±6 ka) and the top at 50.05 m (58±4 ka). The Loire floodwaters began to enter the cave at ~70 ka (bottom of U4, LRC Ib) and must have impeded human occupation, although the resulting 1 to 1.5 m of alluvial deposits [43] did not reach the elevation of the engravings on the cave wall. At this time, the cave entrance was partially obstructed, with only about 70 cm clearance below the cave entrance lintel. No lithic artefacts were found within or on top of the alluvium inside the cave, and so the engraved marks were probably made before the alluvial infill. However, many bones and teeth from large mammals were carried into the cave by hyenas, so access to the cave was clearly possible.

Finally, the aeolian deposits (U2) of the very cold and dry period of the Lower Pleniglacial (MIS 4) and the gravitational deposits of U1 (LRC I-c and d) during the MIS 3 completely sealed the cave. Complete closure of the cave entrance is precisely defined from the lowest elevation of the natural ceiling in the entrance of the cave (50.75 m). By interpolating this elevation onto the Bayesian age model (Fig 15) we determine that sediment deposition closed the cave > 51 ka ago (95% confidence), or at 57 ± 3 ka (68% confidence interval). This is the last time Humans and other large animals could have accessed the cave, until it was rediscovered the beginning of the 20^th^ century.

## Discussion

The different types of marks visible on the cave walls (geomorphological, animal, ancient or post-1912 anthropogenic) were discriminated macroscopically (profile, cross-section, colour, conservation) and statistically from their dimensions (width, angle of incision and depth). To obtain these measurements, the marks were modelled in 3D using photogrammetry. Animal claw marks are distinguished from traces made by human fingers or tools by their distribution, arrangement, morphology and the distance between two consecutive tracks. LDA processing has shown that the finger patterns created during recent experiments are very similar to the engravings on the Circular and Triangular Panels; these ancient engravings were thus most likely created using fingers. The LRC finger flutings cannot be linked to a collection of material for functional or other different purposes. On the one hand, the depth of the lines does not offer a substantial volume, and therefore does not appear in a material search. If one had wanted to collect the powder corresponding to the accessible film on the wall, surface scrapings would have been more appropriate than the narrow and neat traces observable on the walls of the LRC. Furthermore, the organisation and care taken with the three main panels described on the walls of the LRC show an approach that is more graphic than functional. This type of collection has also been discarded in other contexts related to Neanderthal man [90]. Finally, we compared the structural and physical characteristics (state of preservation, degree of alteration, relief, colour) of the modern traces left on the cave walls by the 1912 excavations (mostly made with a metal tool) with those supposed to be Palaeolithic traces. The two types of traces can clearly be distinguished from one another.

The eight panels of digital traces form a seemingly organised set on the longest and most regular wall away from the cave entrance. There even seems to be a progression in the complexity of these graphic entities, particularly from the first to the sixth panel. These traces were meticulously made only on selected surfaces and most often exploiting the shape of the cave wall. The spatially close Circular and Undulated Panels also demonstrate the care taken in making these engravings: the former is composed of deep (forcefully made) digital traces of a slightly oblong circular shape, and the latter is composed of wavy axial traces around which numerous other traces have been added. These two entities could be considered as one. The Triangular Panel has a shape that exploits the shape of the surface used. It has preserved areas that retain the brown altered film that originally covered the entire triangular surface of the wall: two narrow parallel bands and three triangles (see S12 Fig). This careful avoidance of damage to the unused surface cannot be by chance. For example, we hypothesise that when the finger approaches the largest triangle, it deviates very slightly from its original line. When it reaches the summit of the triangle, its speed is slowed, a stronger pressure applied and the finger tilted on the side of the triangle to create a very abrupt boundary (detectable on three of the views in the figure in S12 Fig), the opposite side of the trace is much less inclined. It is not possible that the shape obtained here and found on the other sides of the three triangles is due to chance, it is clearly intentional. The layout of these non-figurative graphic entities is an organised, deliberate composition, and is the result of a thought process giving rise to conscious design and intent.

OSL dating indicates that the sediment deposition closed the cave > 51 ka (95% confidence) ago, or at 57 ± 3 ka (68% confidence interval). This age makes access to the cave interior by anatomically modern humans (AMH) highly unlikely, as we believe that evidence for their arrival in western Europe prior to 45 ka (Bacho-Kiro) is not yet demonstrated [58, 91, 92]. The non-figurative engraved marks at La Roche-Cotard are necessarily older than 57 ± 3 ka, and can be, therefore, confidently stated to be of Neanderthal origin. The graphic productions identified on the walls of La Roche-Cotard demonstrate a deliberate creative process visible in the spatial arrangement of the engraved marks on the cave wall. This is perhaps one of the most remarkable aspects evidenced by the creative ensemble at La Roche-Cotard. As discussed above, there is little graphic evidence associated with Neanderthals, and that is mainly on **mobile** objects (pebbles, slabs, bones…), rather than walls. In contrast, the walls of La Roche-Cotard testify to something different: the frequent repetition of thoughtful gestures, organised in space both on the wall surfaces and with respect to the cave as a whole.

**Fig 18:**
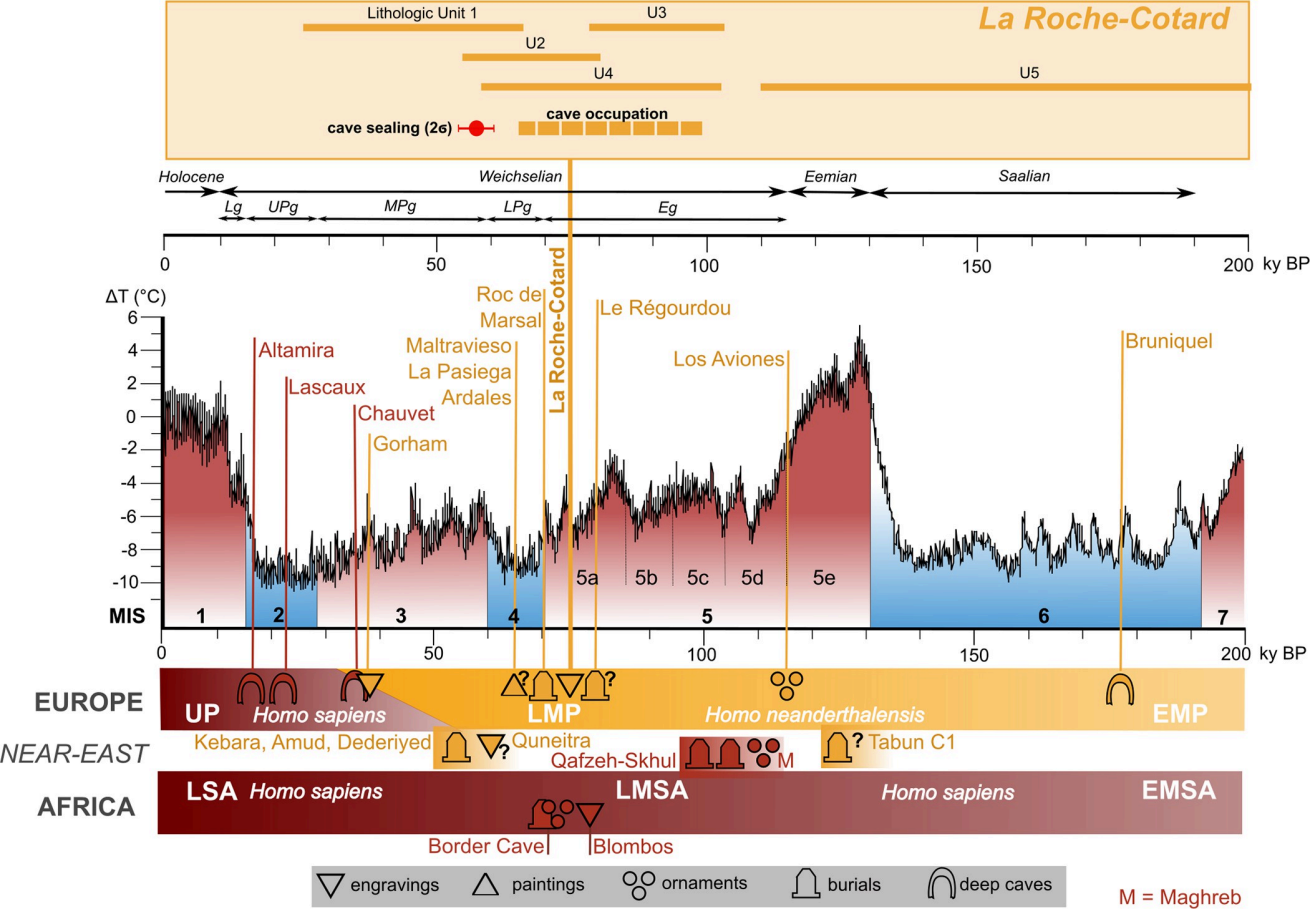
The chronological position of La Roche-Cotard in comparison with other sites yielding artefacts of a symbolic nature. The curve [100] indicates climatic variations from 200 ka to the present (x-axis); on the y-axis, in degree Celsius, the difference in the average temperature between a given period and today is indicated. Eg = Earlyglacial, MPg = Middle Pleniglacial, UPg = Upper Pleniglacial, Lg = Lateglacial, MIS = Marine Isotope Stage. Yellow and red colors indicate the presence of *Homo neanderthalensis* and *Homo sapiens*. The caves of Chauvet, Lascaux, Roc de Marsal, Le Régourdou and Bruniquel are in France, Altamira Maltravieso, La Pasiega, Ardales and Los Aviones are in Spain, Gorham in Gibraltar, Qafzeh, Tabun and Skhul are in Israel, Border Cave and Blombos are in South Africa. EMSA = Early Middle Stone Age, LMSA = Late Middle Stone Age, LSA = Late Stone Age, EMP = Early Middle Palaeolithic, LMP = Late Middle Palaeolithic, UP = Upper Palaeolithic.

Developed graphic intention is shown by these gestures, as is clear from our analysis of the Triangular Panel. The attention paid to the location and the succession of each organised layout testifies to an undeniable formal, graphic and spatial composition, although the intention behind this composition escapes us. On the other hand, these traces are not figurative (any more than the other graphic productions identified for this period). As far as we know, in the examples unquestionably accepted by the research community, hominins did not produce figurative art in this time period all around the world; this is shown in Europe and Western Asia by Neanderthal sites in Crimea [24], the Balkans [20], the Golan Heights at Quneitra [27, 93], or north Germany at Einhornhöhle [1] and as the same time by contemporary Anatomically Modern Humans in southern Africa [94]. Although the finger tracings at La Roche-Cotard are clearly intentional, it is not possible for us to establish if they represent symbolic thinking (Fig 18). Nevertheless, our understanding of the relationship between Neanderthals and the symbolic and even aesthetic realms has undergone a significant transformation over the past two decades and the traces preserved in the cave of La Roche-Cotard make a new and very important contribution to our knowledge of Neanderthal behaviour.

La Roche-Cotard cave also adds a contribution to the record of prehistoric graphic productions; these are contemporary with those made by anatomically modern humans in South Africa but prior to the figurative graphics of the European Upper Palaeolithic (end of MIS 3 and MIS 2), such as the masterpieces from the cave of Chauvet-Pont d’Arc [95], or those recently discovered in the Sulawesi caves, more than 45 ka old [96–98]. Our research demonstrates that the engraved marks at the LRC are clearly attributable to an earlier period, >51 ka. This time interval corresponding to Mode 3 technologies, as defined by Clark [99], encompasses the European Middle Palaeolithic (the Neanderthal "domain"), the contemporary cultures of the eastern Mediterranean (the Levantine Mousterian, where Anatomically Modern Humans and Neanderthals coexisted and interacted), and the Mousterian of the Maghreb and the Middle Stone Age of Sub-Saharan Africa (where only Anatomically Modern Humans were present) (Fig 18). The attribution to Neanderthal of the graphic productions at La Roche-Cotard pays tribute to this lost humanity, whose role in the biological and cultural evolution of humans is undergoing profound revision. In terms of culture, we now have a better understanding of the plurality of Neanderthal activities, attesting to elaborate and organized social behaviours that show no obvious differences from those of their contemporaries, Anatomically Modern Humans, south of the Mediterranean.

## Supporting information

S1 Text Experimental protocol to identify the tool used to execute the tracings of the Rectangular Panel. (PDF)

S2 Text Optically stimulated luminescence. Method details.
(PDF)

S3 Text R Code–statistical data processing. (PDF)

S4 Text Curvature analysis of the Triangular Panel and source code (in Pascal). (PDF)

S1 Video Animated 3D Model: The main decorated wall of the Roche-Cotard cave. (WMV)

S1 Fig Photograph of the north wall of the Pillar Chamber. 1: Coniacian quartzitic sandstone; 2 and 3: Graphic entities; 4 and 5: tuff wall still covered with a light brown film showing local removal of some of the film due to erosion. The six traces (5) are due to a metal tool used by the excavators in 1912; 6: tuff wall with the brown film removed; 7: overhang; 8: small decarbonation recesses; 9: chert layer; 10: yellow Turonian tuff; 11: cavity filled with compact red decarbonation clay; 12: modern sedimentary layer covering the compact layer.
(TIF)

S2 Fig Map showing the location of lithic industry discoveries. The two brown zones 1 and 2 locate F. d’Achon’s discoveries (1. typical Mousterian with Levallois flaking, surmounted by the Mousterian of Acheulean tradition, 2. typical Mousterian with Levallois flaking). The three green zones locate recent discoveries (3, 4 and 5. Typical Mousterian with Levallois debitage). S3 indicates the place of lithic industry drawings in S3 Fig. S4B indicates the place of typical Mousterian with Levallois flaking in S4 Fig. S4A indicates the place where the Mousterian of Acheulean tradition triangular broken biface has been discovered as well as its drawing and photograph in S4 Fig. S5B indicates the place where the flint blade has been discovered and its macro and microphotography in S5 Fig.
(TIF)

S3 Fig The Mousterian industries of La Roche-Cotard discovered in 1912. Mousterian of Acheulean Tradition bifaces (1, 2, 3) discovered in 1912 (drawing M. Lajudie in Dubreuil-Chambardel, La Touraine préhistorique. 1923). Pictures of ten bifaces [19].
(TIF)

S4 Fig The Mousterian industries of La Roche-Cotard discovered in 2009. A. Acheulean Tradition Mousterian biface discovered in the South Pillar Chamber (S2 Fig), photo and drawing T. Aubry. B. Typical Levallois flakes discovered in the Mousterian Gallery and in front of the entrance (S2 Fig Zones 3, 4 et 5), drawing L.A. Millet-Richard.
(TIF)

S5 Fig Traceology of the broken biface and of the small blade discovered in 2009. A. Broken triangular flint biface discovered not deeply in the sediment accumulated in a window of the chert layer. It shows significant rounding of its distal part (1, 2, 3), creating a dull, abrupt edge most probably produced by transverse contact with mineral matter. The other edges of this implement do not display such characteristics. B. Second stone implement, made on a blade, used to process mineral matter and hide. Three zones of use are identified: the first suggests hide processing (1). The second and third use-areas present features which indicate scraping soft, abrasive, mineral matter (2, 3).
(TIF)

S6 Fig Clawmarks of *Ursus spelaeus* in the Pillar Chamber. The shape and distance between marks are consistent with traces of *Ursus sp*.
(TIF)

S7 Fig Approach used to measure traces. The first four images show the Triangular Panel (TRI), the Rectangular Panel (REC), the bear scratched area (CLA) and finally the Circular Panel (CIR); at the bottom of each picture, the cross-sections made with CloudCompare on the photogrammetries. Four cross-sections were made on the Triangular Panel, T1 to T4 and six on the circular, C1 to C6. Only T1 and C6 are presented below each panel. On these two sections the limits and names of the different plots are indicated, their measurements can be found in Table 2 (T1a corresponds to section a of cut 1 of the Triangular). The cross-sections on the Rectangular Panel have too little relief to allow measurements. The measurements of the width and the angle of incision of the line were carried out using the CloudCompare application and the depth was calculated using a simple mathematical formula: depth = width / 2x tangent (incision angle/2). The same method was used for the scratched space because of the multiple crossings of the traces on the wall. The following five photos show experimental traces made to hypothesise which tool or tools might have been used to make the tracings of the Rectangular Panel. Among the 7 tools that were used for this experiment (S1 Text), we present here only 5 panels, WOOA traced with an antler point, WOOV with a wood point, FLIN with a flint point, BONE with a bone point and FING with a finger positioned flat. In each image the location of the cross-sections that have been made can be seen; only one is presented below the photo. The iron point tracings were less interesting and the finger positioned on edge was used very little as blood was easily lost when the finger passed over the very aggressive surface of the wall. On these 5 panels, all measurements were made directly on the sections that appear on the sections made. All the scales are in meter.
(TIF)

S8 Fig Experimental determination of the direction of finger flutings. A. Experiment: some crushed is prepared tuff, then placed in a small flat container, moistened a bit, beat and grooved with a finger on its surface. Result: on the bottom of the trace, some reliefs like scales lifted up in the opposite direction of the finger passage are observable. The black arrow indicates the direction of the trace; white arrows the scales. B. Circular Panel, trace C1a. The scales are visible on the bottom of trace (white arrows) and show the direction of the finger fluting.
(TIF)

S9 Fig Panel a. (panel in entrance of Pillar Chamber). From top to bottom, photograph and survey of the ancient anthropic traces in black and animal traces in blue, numbering of the anthropic traces. The clear traces are in continuous line, when the trace is deep the line is thicker. Traces that are more difficult to read are dashed.
(TIF)

S10 Fig Panel b. (panel of the fossil). From top to bottom, photograph and survey of ancient anthropic traces in black, animal traces in blue, surface of the fossil section in green, numbering of the traces. The clear traces are in continuous line, when the trace is deep the line is thicker. Traces that are more difficult to read are dashed.
(TIF)

S11 Fig Photograph of the Undulated and Circular Panels (panels d and e). The close proximity of these two panels suggests a connection between them. We do not rule out possible contemporaneity between them. Photo E. Lesvignes.
(TIF)

S12 Fig Analysis of the Triangular Panel and especially of its left part. a). Survey of the finger flutings of the totality of the panel. It permit to situate the left part of the panel which has been studied particularly and specially the three preserved triangles A, B and C. b). Orthophoto from the photogrammetry of the left part of the panel. The two triangles A and B are clearly visible, the triangle C with some difficulty due to its alteration. c). The same surface with its contour lines which give the surface relief. The lines are equidistant sections (1mm) parallel to the average plane of the panel, not horizontal. d). A coloured model representing the microrelief of the panel. Red indicates concave surface (relative to the observer’s axis of vision), blue indicates convex surface. Thick line at 0 of the scale indicates flatness of the surface. The units of bending intensity are given in colour range from -0.18 (convex) to +0.08 (concave) for curvature, i.e., from 8 mm to 10 mm for radii of curve. The colour range on the right shows the range and gradation of the panel’s colouration: red and yellow are the concave surfaces (for the observer), green and blue the convex surfaces and, at the limit of yellow and green, the areas without curvature. e). Detail of the groove along the left side of the triangle A. Arrow 1 shows the beginning of the strong slope, Arrow 2 shows a narrow band corresponding to the part of the groove on the side of the triangle. Arrow 3 shows a red band corresponding to the deep part of the groove, Arrow 4 shows a wide yellow stripe corresponding to the other side of the groove with a very gentle slope. (Y. Egels, see S4 Text).
(TIF)

S13 Fig The Rectangular Panel (panel g). Top, photograph of the Rectangular Panel in oblique light from the right. Bottom, sketch of the survey of the ancient anthropic traces of the panel and numbering. The continuous lines depict finger traces, the long dashed lines depict finger traces that are difficult to recognise. The short dashed lines are the lines of the pointed base of the V-section of most traces that are not made with the flat finger. Dotted lines are the ridge lines between two parallel V-section traces (survey S. Audouy).
(TIF)

S14 Fig Location of undisturbed layers or remaining portions of undisturbed layers, still present near the LRC I cave entrance with OSL ages. Same figure than Fig 5 in main text integrating numbering of the samples dated by OSL and results of the datings (Table 5).
(TIF)

S15 Fig Lithostratigraphy, geometric distribution of the superficial deposits outside the cave with OSL ages. Same figure than Fig 5 in main text integrating numbering of the samples dated by OSL and results of the datings (Table 5).
(TIF)
