# Supplementary material for: The earliest unambiguous Neanderthal engravings on cave walls: La Roche-Cotard, Loire Valley, France
Source: PLoS One. 2023 Jun 21;18(6):e0286568. doi: 10.1371/journal.pone.0286568 (PMC10284424; doi:10.1371/journal.pone.0286568)
Supplement: S1 Text — (PDF) [file pone.0286568.s001.pdf]

Experimental Protocol

to identify the tool used to execute the tracings of the rectangular panel

To carry out this experiment,

- a modern anthropic cavity was sought. It had to be dug in the terminal part of the Upper Turonian so that we could experiment on a wall whose consistency had to be as close as possible to that of La Roche-Cotard.
- 21 rectangular zones, the same size as the rectangular panel, were selected and marked out on the walls of the cavity
- seven tools were chosen and prepared so that three panels were traced with the same type of tool by three different operators

The seven tools chosen were

1). a cut flint with a point (FLIN),
2). a fragment of freshly cut and pointed pine branch (WOOV),
3). a fragment of a recently collected deer antler (WOOA),
4). a large bone splinter from a fresh bone and pointed on a hard flat stone (BONE),
5). a long metal point (MET),
6). one of the three middle fingers of the operator's hand (FIN),
7). one of the three middle fingers of the hand used on the side (FINS).

The number of traces to be made to cover the central part of the panel was 18 and each trace could be ironed several times but always with the same tool.

Each team was made up of two people. The « operator » who traced using only the tool determined for each panel and the « observer » who had to note on the sheet provided the method the operator used to make his traces:

- the way the tool was held
- the angle of attack of the tool on the wall
- the angle of attack of the line
- the angular position of the tool in relation to the axis of the trace
- the speed at which a trace is made
- the position of the operator in relation to the panel
- and any other particular remark

Photogrammetry of each of the 21 panels was carried out. The Cloud Compare application was used to measure the width, depth and angle of incision of each trace on the cuts made.
