# Supplementary material for: The earliest unambiguous Neanderthal engravings on cave walls: La Roche-Cotard, Loire Valley, France
Source: PLoS One. 2023 Jun 21;18(6):e0286568. doi: 10.1371/journal.pone.0286568 (PMC10284424; doi:10.1371/journal.pone.0286568)
Supplement: S2 Text — Method details. (PDF) [file pone.0286568.s002.pdf]

Supplementary text

Optically stimulated luminescence

Optically stimulated luminescence (OSL) dating is an absolute chronological method that determines when sediments were last exposed to daylight. When sediment is buried and thus shielded from light, the latent luminescence signal begins to accumulate in mineral grains (quartz and feldspar) contained in the sediment due to the interaction with ionising radiation emitted mainly from the naturally occurring U- and Th-series, K-40 and cosmic rays. By optical stimulation, the intensity of the OSL signal can be determined and calibrated in terms of absorbed dose (the equivalent dose, D_e, [Gy]). The burial age is subsequently determined by dividing the D_e by the environmental dose rate ($\dot{D}$, [Gy.ka^{-1}], which is determined using independent measurements (e.g. high resolution gamma spectrometry).
Here we present the OSL ages for 50 samples using the blue stimulated signals from multi-grain (8 mm) quartz aliquots as well as the infra-red stimulated luminescence (pIRIR) signal from multi-grain K-feldspar aliquots (2 mm). 12 quartz samples were also measured using the green OSL signal from single-grain quartz aliquots.

In this study, we test the hypothesis that the main cave and the shelters were accessible for some time after the arrival of *Homo sapiens* in the area, around 37,000 years ago[(1)].

Sampling and context

A total of 50 OSL samples were collected at La Roche-Cotard (LRC), from 2016 to 2022. 43 samples were analysed in Denmark (DTU Physics) and the remaining seven samples in Hungary (Mining and Geological Survey of Hungary). Multi-grain quartz ages from 12 of the 43 samples (all from LRC IV, see below) analysed in Denmark, were published in Marquet et al.[(2)] and the seven multi-grain quartz samples analysed in Hungary (marked with asterisks in Fig. 6 and Table SI.5) were published in 2016 and 2019[(2,3)]. In this study we date the 31 new samples and revise the previously published quartz ages using the new calculations described here (i.e. water content assumptions and dose rate modelling). On average, these recalculations increase the age of the samples measured in Hungary by 11±4 % (n=7) and 16±6 % (n=3) compared to the publications in 2019 and 2016, respectively, and those measured in Denmark by 11.0±1.6 % (n=12). ED 7 shows a schematic overview of all 50 sample positions.

La Roche-Cotard is located in the Loire Valley in France and consists of a small cave and two nearby shelters. The cave itself is referred to as LRC I, the sediments in front of and below the cave entrance as LRC II and the two nearby shelters as LRC III and LRC IV (ED 7). Samples were taken from five different deposition units (U1-5) and from within the inner cave of LRC I (I.C.) and from the entrance to the cave in LRC I (E.C). U5 is located in the karstic sediments. U4 is of fluvial origin (the Loire River). U3 is colluvium deposited during a colder climate[(4)]. U2 is from aeolian transport, also during a colder climate, and U1, the uppermost and thickest unit, is a mixture of primary and reworked colluvium.
In addition, three samples were taken from the inner part of the cave in LRC I (167809, -17 and -18). These samples are referred to as I.C. Within the entrance to LRC I, five samples were taken from holes in the entrance wall (197340, -38, -39, 227801 and 227802). These are referred to as E.C and have not been identified with a specific unit, except for samples 227801 and 227802, which are identified as belonging to unit 4. In addition, three important samples collected from holes in the rock wall outside and just above the cave entrance (samples 197332, -28 and -33). Based on elevation, these three samples should yield ages younger than the closure age. The two samples (167806 and -05) taken just below the closure elevation should yield ages older (or equal to) the closure age. The levels associated with human occupation (Mousterian levels) are all in unit 4.
Units 1-5 have different elevations at the four sites LRC I-IV. The elevation (NGF, General Levelling of France) for the individual units and the Mousterian occupation levels are given in Table SI.1.

Table SI.1: Elevation limits (NGF) for depositions units 1-5 and the Mousterian occupation levels in the four sites LRC I, LRC II, LRC III and LRC IV.

	Unit elevation limit [m]			
Unit/level	LRC I [m]	LRC II [m]	LRC III [m]	LRC IV [m]
Unit 1	50.90 - 55.35	-	-	47.00 - 50.10
Unit 2	49.00 - 50.70	-	45.80 - 46.00	44.90 - 47.00
Unit 3	-	45.13 - 48.00	-	-
Unit 4	49.20 - 50.29	45.30 - 45.80	44.85 - 45.80	44.10 - 44.90
Unit 5	-	44.80 - 45.13	-	42.40 - 43.90
Mousterian level	48.50 - 49.30	45.20 - 45.60	44.85 - 45.00	44.80 - 44.90

Sediment samples were taken by inserting steel tubes (ø=4 cm, length=20 or 15 cm) into cleaned sections. In the laboratory, the samples were prepared under subdued red-orange light conditions. The ends (outer 5 cm) of each sample, potentially light-exposed during sampling, were reserved for radionuclide concentration and water content measurements. The inner portions of the samples were used for OSL measurements.
For the latter, the material was first wet-sieved to 180-250 μm and then processed through several standard laboratory steps to extract clean quartz and potassium-rich feldspar grains. The samples were first treated in 10% HCl for one hour to remove carbonates. After rinsing, 10% hydrofluoric acid was added for 20 min to clean grain surfaces and remove any attached clay particles. Residual fluoride contamination from the hydrofluoric treatment was removed using 10% HCl for 40 min. Quartz and K-rich feldspar grains were separated using heavy liquid separation (sodium heteropolytungstate "LST Fastfloat'") with a density of 2.58 g/cm^3. Finally, the quartz-rich extract was treated with a 40% HF solution for 40 min to remove any remaining feldspar grains and the outer ~10 μm affected by alpha radiation. Any residual fluoride contamination from the HF treatment was removed using 10% HCl for 40 min. The luminescence purity of the resulting quartz extract was examined by testing for IRSL sensitivity (a measure of feldspar contamination, see section: *Multi-grain quartz rejection criteria*).

Experimental details

Instrumentation

All luminescence signals were measured using automated TL/OSL Risø Readers[5] equipped with blue (470±30 nm) og IR (870±40 nm) stimulation LEDs, providing power densities of approximately 80 and 150 mW/cm^2 at the sample position, respectively. Single-grain quartz OSL signals were measured using a single grain attachment to the OSL reader[6], fitted with a steerable green 10 mW Nd:YVO_4 solid-state diode-pumped laser beam (532 nm) as the stimulation light source.

Quartz and feldspar have strong luminescence emissions centred at 365 nm and 410 nm, respectively[7]. These luminescence signals were detected using EMI 9635QA photomultipliers in combination with either 7.5 mm Hoya U-340 filters (quartz) or a filter pack consisting of 2 mm Schott BG-39 in combination with 4 mm Corning 7-59 (feldspar).

In situ beta irradiations used calibrated ^{90}Sr/^{90}Y beta sources[8]. The beta dose uniformity across the sample area for all sources was better than 5% and thus no correction for spatial non-uniformity was applied[9].

Radionuclide concentrations were determined using high-resolution gamma spectrometry[10,11] and converted to infinite matrix dose rates using the conversion factors of Guérin et al.[12]. The sediment samples were crushed and homogenised before mixing with wax and cast in a fixed cup-shaped geometry to retain ^{222}Rn[11]. Equilibrium between ^{222}Rn and ^{226}Ra was ensured by storing the cups for more than 20 days (i.e., >5 half-lives of ^{222}Rn) before counting. Cosmic ray dose rates were calculated following Prescott and Hutton[13] assuming that the burial depths recorded in 1846[2] represents the life-time burial depths.

In situ gamma dose rates were measured for eight of the samples. These measurements were made because spatial heterogeneity in the gamma field was expected. These in situ gamma dose rates were measured in two different ways: 1) a 1.5" LaBr probe was inserted into the hole left after the OSL sample had been taken. The in situ spectrum was recorded for ~20 minutes at each sample location. The acquired spectra were then converted into dose rates using the energy threshold technique [14] and the calibration curve established by Miallier et al. [15]. 2) Al_2O_3:C pellets, inserted in aluminium tubes with a wall thickness of 3 mm, were placed in the OSL sample holes for for 215 days. The protocol of Kreutzer et al. [16] was used to measure the absorbed dose, which enabled the calculation of in situ gamma and cosmic dose rates.

An XRF attachment to the Risø reader was used to measure the K-contents, relative to the sum of K+Na+Ca, in seven samples [17,18].

OSL measurements

Equivalent dose determination used the single-aliquot regenerative-dose (SAR) procedure [19]. Laboratory dose response curves (DRCs) were determined using a minimum of three sensitivity-corrected regeneration dose points (L_x/T_x) bracketing the sensitivity corrected natural signal (L_n/T_n) as well as a recuperation point (zero dose point) and a recycling point. The sensitivity corrected IR depletion ratio [20] was measured at the end of each DRC.

The DRCs were fitted using a single saturating exponential function of the form $L_x/T_x = I_0[1 - \exp(-D/D_c)]$, where L_x/T_x is the sensitivity corrected OSL response, I_0 is the saturation value and D_c is a measure of the curvature (note that D_c is often termed "D_0" in the literature).
Equivalent dose estimates were derived from individual aliquots using either "BayLum" [21] (single-grain only) or "Analyst" [22] and uncertainties are based on counting statistics, fitting uncertainties and an instrument reproducibility of 0.5% per OSL measurement for multi-grain measurements and 2.5% per OSL measurement for single-grain measurements [23].

Multi-grain quartz OSL measurements

The OSL signals from multi-grain quartz aliquots were obtained using blue light stimulation at 125 °C for 100 s. A preheat temperature of 260 °C for 10 s, a cutheat temperature of 220 °C and a test dose of ~30% of the natural dose was used for multi-grain quartz dose measurements, unless otherwise specified. In addition, a high-temperature blue light stimulation at 280 °C for 40 s was inserted between SAR cycles to minimise potential recuperation effects [24]. The light sum over the initial 0.5 s of optical stimulation, less that from the subsequent 0.5 s of stimulation, was used for dose estimation, i.e., early background subtraction (EBG) to isolate the fast component [25]. Aliquots were prepared on stainless steel discs using an 8 mm spot of silicon oil, giving approximately 2,000 grains per aliquot [26]. In OSL dating it is common to reject results from individual aliquots according to certain rejection criteria, e.g., the recycling and IR depletion ratios are expected to be consistent with unity and the recuperation is usually only accepted if it is less than 5% of the sensitivity corrected natural signal. In this study, the application of these criteria did not result in significant changes to either equivalent doses or distribution width (see section: *Multi-grain quartz rejection criteria*). Here we also examine the effect of the InterQuartile Rejection (IQR) criterion, as described by Medialdea et al. [27]; this identifies and rejects individual dose values if they are more than 1.5 interquartile ranges above the upper quartile (75 percent), or below the lower quartile (25 percent).

Single-grain quartz OSL measurements

A similar single-aliquot regenerative-dose (SAR) protocol [19] was used for single-grain equivalent dose determination; it employed a preheat of 260 °C (for 15 s), a cutheat of 220 °C and a test dose of 50 Gy. An IR stimulation at 50 °C (for 100 s) was inserted prior to each OSL stimulation at 125 °C (0.9 s) to minimise any effects of potential feldspar contamination [28]. After each SAR cycle a high-temperature blue bleach [24] at 280 °C (for 100 s) was inserted to minimise potential recuperation effects. For single-grain measurements, the OSL signal was summed over the initial 60 ms and the background was summed over the final 150 ms.
Here, single-grain laboratory dose response curves (DRCs) consist of a minimum of six sensitivity-corrected regeneration dose points, a recuperation point (i.e., zero dose point), two recycling points and an IR depletion point [20]; i.e., a ratio between sensitivity corrected L_x/T_x values measured with and without the prior IR stimulation before the L_x measurement).

Several methods of data analysis are applied to the single-grain data: i) the central age model (CAM)[29], ii) the average dose model (ADM)[30] and iii) the Baysian central dose model (BayLum)[21,31–33]. For the multi-grain quartz data, the DRCs are fitted using a single saturating exponential passing through the origin. In i) an ii) uncertainties are assigned to individual doses using "Analyst 4.52"[22] and consist, as a minimum, of contributions from Poisson counting statistics, instrument reproducibility and curve fitting errors. In iii) the OSL data is analysed in a Bayesian framework, where individual equivalent doses are not parameterised independently, but the selected grains are analysed simultaneously assuming they all belong to the same dose distribution.

Only grains with a relative uncertainty on the natural OSL test dose signal (T_n) of less than 20% ($s_{Tn} < 20\%$) were included in the analysis. Application of the following rejection criteria was tested: a) the recycling ratios must be consistent with unity within two standard deviations, b) the IR depletion ratio[20] with sensitivity correction must be consistent with unity within two standard deviations and (c) the recuperation dose must be consistent with zero Gy. On average, these criteria did not result in significant changes to either equivalent doses or distribution width (see section: *Effect of single-grain rejection criteria*).

Multi-grain feldspar IRSL measurements

A preheat temperature of 320 °C for 100 s was used for all K-rich feldspar dose measurements unless otherwise specified (e.g., preheat plateau measurements in section: *Multi-grain K-rich feldspar preheat plateau and dose recovery*). Multi-grain K-rich feldspar aliquots were stimulated with IR light at 50 °C for 200 s followed by IR stimulation at 290°C for 200 s, i.e., a pIRIR(50,290) protocol[34]. A test dose approximately equal to the size of the natural dose was used and a high-temperature IR bleach at 325 °C for 200 s inserted between SAR cycles to minimise potential recuperation effects. The light from the initial 2 s of stimulation less that from the last 10 s of stimulation was used for dose estimation, i.e., late background subtraction, LBG. Aliquots were prepared on stainless steel discs using a 2 mm spot of silicon oil, giving approximately 100 grains per aliquot[26].

Dose rate measurements

This section describes the dose rate measurements in the study and the corrections for heterogeneity due to the bedrock material at the site.

The radionuclide concentrations derived from the 50 OSL sediment samples and a single bedrock sample (167804R) are summarised in Table SI.2. The dry infinite matrix beta and gamma dose rates are also given, derived assuming a 20±10% loss of ^{222}Rn compared to its parent ^{226}Ra. Note that the dose rate for sample 187307 is significantly lower than other sediment samples and gives the same dose rate as the bedrock material.

Table SI.2

Lab Code	Fig. code	Unit	Water content (percent)			Radionuclide concentration (Bq kg^{-1})				Infinite matrix dry dose rates (Gy ka^{-1})		R_{corr} dry dose rate	Total dose rates (Gy ka^{-1})		R_{tot} wet total DR	
			Current	Saturated	Assumed	^{238}U	^{226}Ra	^{232}Th	^{40}K	Gamma	Beta	Gamma	Q	KF	Q	KF
167804R			1	10	4	4±6	11.6±0.9	12.3±0.8	88±10	0.296±0.017	0.42±0.03	1	0.74±0.04	1.68±0.12	1	1
187301	**1**	1	1	34	17	24±6	25.9±0.6	28.6±0.5	387±11	0.82±0.02	1.43±0.03	1	1.87±0.08	2.82±0.17	1	1
187302	**2**	1	3	30	17	31±9	27.1±0.7	42.9±0.7	643±11	1.20±0.03	2.18±0.04	1	2.74±0.12	3.7±0.2	1	1
187303	**3**	1	7	51	17	-5±23	30.0±1.9	33.3±1.8	430±24	0.94±0.04	1.61±0.07	1	2.11±0.10	3.06±0.19	1	1
197332	**4**	1	15	63	17	19±5	29.4±1.1	58.3±0.9	429±12	1.23±0.03	1.77±0.04	0.52	1.92±0.09	2.86±0.18	0.80	0.85
197328	**5**	1	10	45	17	14±6	29.1±0.9	53.5±0.9	453±13	1.19±0.03	1.80±0.04	0.52	1.93±0.09	2.87±0.18	0.80	0.86
197333	**6**	1	12	50	17	27±16	31.1±1.2	54.7±1.3	443±20	1.21±0.04	1.80±0.06	0.52	1.93±0.09	2.88±0.18	0.80	0.86
197340	**7**	E.C.	23	77	25	32±13	30.3±1.0	48.1±1.0	242±12	0.97±0.03	1.24±0.04	0.65	1.42±0.08	2.36±0.18	0.84	0.90
197338	**8**	E.C.	23	77	25	32±13	30.3±1.0	48.1±1.0	242±12	0.97±0.03	1.24±0.04	0.58	1.37±0.08	2.31±0.17	0.81	0.88
197339	**9**	E.C.	27	70	25	43±15	29±3	46±2	222±30	0.92±0.05	1.16±0.09	0.59	1.30±0.09	2.24±0.18	0.82	0.88
167806	**10**	2	15	46	15	20±3	31.9±0.6	53.8±0.6	392±9	1.17±0.03	1.67±0.03	0.96	2.29±0.09	3.23±0.18	0.98	0.99
167809	**11**	I.C.	16	50	25	18±10	20.7±0.8	49.1±1.0	265±10	0.93±0.02	1.21±0.03	1	1.63±0.09	2.57±0.19	1	1
167817	**12**	I.C.	16	50	25	18±10	20.7±0.8	49.1±1.0	265±10	0.93±0.02	1.21±0.03	0.85	1.52±0.09	2.47±0.18	0.93	0.96
167818	**13**	I.C.	16	50	25	18±10	20.7±0.8	49.1±1.0	265±10	0.93±0.02	1.21±0.03	0.85	1.52±0.09	2.47±0.18	0.93	0.96
227801	**14**	4	14	57	17	34±7	39.1±1.4	49.8±1.1	613±17	1.34±0.04	2.27±0.06	0.79	2.63±0.12	3.6±0.2	0.92	0.94
167805	**15**	4	11	44	17	40±16	30.3±1.2	47.6±1.1	388±18	1.08±0.03	1.61±0.05	0.89	2.06±0.10	3.01±0.19	0.96	0.97
187312	**16**	2	5	37	15	60±31	26±2	47.0±1.9	452±32	1.09±0.04	1.72±0.09	0.97	2.27±0.11	3.2±0.2	0.99	0.99
227802	**17**	4	9	41	17	76±7	43.1±1.5	35.2±1.2	593±17	1.18±0.04	2.17±0.06	0.66	2.32±0.11	3.3±0.2	0.87	0.91
187311	**18**	2	3	45	15	32±10	25.9±0.8	47.2±0.9	389±15	1.04±0.03	1.56±0.04	0.98	2.12±0.09	3.06±0.18	0.99	0.99
161267*	**19**	4	29	37	17	31.7±0.5	31.7±0.5	41.3±0.4	424±5	1.04±0.03	1.67±0.03	0.82	2.06±0.09	-	0.93	-
167812	**20**	4	22	51	17	39±20	31.9±1.5	38.6±1.2	325±20	0.94±0.04	1.40±0.06	0.83	1.75±0.09	2.70±0.18	0.93	0.95
187321	**21**	3	10	39	15	21±7	17.5±1.5	13.0±1.1	305±16	0.52±0.03	1.03±0.05	0.95	1.28±0.06	2.22±0.15	0.98	0.99
187322	**22**	3	10	39	15	21±7	17.5±1.5	13.0±1.1	305±16	0.52±0.03	1.03±0.05	0.95	1.28±0.06	2.22±0.15	0.98	0.99
187320	**23**	3	13	41	15	26±7	16.5±1.5	29.2±1.1	447±18	0.81±0.03	1.49±0.05	0.99	1.89±0.08	2.83±0.17	1.00	1.00
187323	**24**	5	7	33	18	23±6	15.6±1.3	22.4±1.0	307±15	0.61±0.02	1.08±0.04	0.88	1.32±0.07	2.26±0.16	0.96	0.97
187310	**25**	3	2	36	15	19±14	16.4±1.1	20.5±1.1	343±16	0.63±0.02	1.17±0.04	1.00	1.49±0.07	2.44±0.16	1.00	1.00
187306	**26**	3	2	34	15	24±10	19.7±0.8	19.9±0.8	303±12	0.61±0.02	1.10±0.04	0.94	1.39±0.06	2.34±0.15	0.98	0.99
187309	**27**	3	1	46	15	66±35	32±2	11.6±1.8	171±29	0.50±0.04	0.83±0.08	0.95	1.10±0.08	2.05±0.15	0.98	0.99
187305	**28**	3	3	26	15	13±3	16.7±0.6	20.3±0.4	366±8	0.644±0.017	1.23±0.02	0.94	1.51±0.06	2.46±0.15	0.98	0.99
181362*	**29**	4	17	35	17	15.2±0.3	15.2±0.3	22.3±0.4	451±4	0.724±0.014	1.441±0.016	1.00	1.77±0.08	-	1.00	-
187308	**30**	4	1	64	17	28±13	26.2±1.0	22.8±0.8	269±13	0.66±0.03	1.09±0.04	1.00	1.43±0.07	2.37±0.16	1.00	1.00
207307	**31**	4	4	45	17	17±8	14.5±1.7	22.7±1.3	403±20	0.69±0.03	1.32±0.06	1.00	1.61±0.08	2.55±0.17	1.00	1.00
181361*	**32**	4	18	33	17	16.1±0.3	16.1±0.3	22.1±0.4	413±4	0.698±0.015	1.353±0.016	0.93	1.64±0.07	-	0.98	-
187307	**33**	4	0	64	17	11±10	8.6±0.8	9.9±0.8	165±13	0.307±0.017	0.57±0.03	0.97	0.74±0.04	1.69±0.13	0.99	1.00
187304	**34**	5	0	31	18	13±14	16.0±1.1	21.3±1.1	382±21	0.66±0.03	1.27±0.06	0.92	1.50±0.08	2.44±0.17	0.97	0.98
161263*	**35**	4	11	33	17	24.2±0.4	10.0±1.0	26.3±0.3	567±5	0.827±0.012	1.710±0.018	1	2.09±0.09	-	1	-
167831	**36**	1	2	35	17	28±17	30.6±1.2	40.6±1.2	574±21	1.14±0.03	2.03±0.06	1	2.52±0.12	3.5±0.2	1	1
167830	**37**	1	7	37	17	19±9	27.5±0.7	39.0±0.9	547±12	1.08±0.03	1.92±0.04	1	2.38±0.11	3.3±0.2	1	1
161264*	**38**	1	9	42	17	22.9±0.4	22.9±0.4	29.8±0.3	404±4	0.83±0.02	1.45±0.02	1	1.86±0.08	-	1	-
167829	**39**	1	7	43	17	23±26	22.5±1.8	31±2	384±23	0.83±0.04	1.41±0.06	1	1.78±0.09	2.73±0.18	1	1
167828	**40**	2	9	30	15	20±10	24.0±0.8	33.2±0.9	419±12	0.89±0.03	1.52±0.04	1	1.95±0.08	2.89±0.17	1	1
167827	**41**	2	8	40	15	30±10	23.4±1.7	27.9±1.5	439±22	0.84±0.03	1.53±0.06	1	1.91±0.09	2.85±0.18	1	1
167826	**42**	2	3	34	15	39±24	15.9±1.5	20.2±1.2	405±23	0.67±0.03	1.32±0.06	1	1.61±0.08	2.55±0.17	1	1
161266*	**43**	4	5	37	17	22.2±0.4	22.2±0.4	44.0±0.4	263±3	0.881±0.019	1.184±0.019	1	1.70±0.07	-	1	-
167823	**44**	4	7	32	17	17±6	21.2±1.1	30.4±0.8	480±13	0.89±0.02	1.63±0.04	1	1.98±0.09	2.93±0.18	1	1
167825	**45**	4	8	43	17	19±40	22±3	37±3	491±36	0.98±0.05	1.71±0.10	1	2.12±0.12	3.1±0.2	1	1
167822	**46**	4	8	42	17	26±7	29.1±1.0	45.5±1.1	426±13	1.08±0.03	1.67±0.04	1	2.18±0.10	3.12±0.19	1	1
167821	**47**	4	7	37	17	15±11	26.8±0.9	38.4±0.9	403±16	0.96±0.03	1.55±0.05	1	1.98±0.09	2.92±0.18	1	1
167820	**48**	5	17	54	18	-1±23	15.6±1.5	54.9±1.7	224±22	0.93±0.03	1.09±0.06	0.96	1.58±0.09	2.53±0.17	0.98	0.99
161265*	**49**	5	26	57	18	20.2±0.4	20.2±0.4	31.2±0.3	356±3	0.789±0.018	1.312±0.018	0.97	1.68±0.08	-	0.99	-
167819	**50**	5	27	53	18	17±25	30.9±1.7	59.7±1.6	213±21	1.09±0.04	1.25±0.06	0.97	1.83±0.10	2.77±0.18	0.99	0.99

Table SI.2: Dose rate summary for the 50 sediment samples and a single bedrock sample (167804R). Samples marked with * were prepared and measured in Hungary by Dr. Edit Thamóné Bozsó (Department of Geological Basic Research, Mining and Geological Survey of Hungary). "Fig. code" refers to the sample label used in Fig. 6 and SI.11. "Unit" is the deposition unit. "I.C." and "E.C." represents samples from the inner cave of LRC I and the entrance of LRC I, respectively (no specific deposition). Both current (field) and saturated "Water content" percentages are given. "Assumed" is the assumed water content (see section: *Water content correction*) used in the calculation of total dose rates. Dry infinite matrix dose rates are derived from radionuclide concentrations measured using high resolution gamma spectrometry. "R_{corr}" is the ratio of the modelled gamma dose rate to the infinite matrix dry dose rate (see section: *Effect of dose rate modelling in LRC*). Total dose rates include the contribution from cosmic rays, internal dose rates, and the effects of water content as well as the modelled correction for dose rate heterogeneity. "Q" and "KF" refer to dose rates to quartz and K-rich (12.60±0.15%) feldspar, respectively. "R_{tot}" is the ratio of the total dose rate (modelled) to the unmodelled total dose rate (see section: *Effect of dose rate modelling in LRC*).

We assume an internal alpha dose rate due to ^{238}U and ^{232}Th to quartz of 0.02±0.01 Gy.ka^{-1} [35] and to K-rich feldspar of 0.10±0.05 Gy.ka^{-1} [36]. For K-rich feldspar extracts, an internal beta dose rate from ^{40}K and ^{87}Rb of 0.86±0.03 Gy.ka^{-1} was calculated assuming a mean grain diameter of 215 μm, a rubidium concentration of 400±100 ppm [37] and an effective potassium concentration of 12.60±0.15%. The latter was measured using a Risø XRF attachment [17,18] (see Figure SI.1) for seven of the samples. This is close to the 14.05% stoichiometric maximum for orthoclase, and consistent with the recommendation of Huntley and Baril [38].

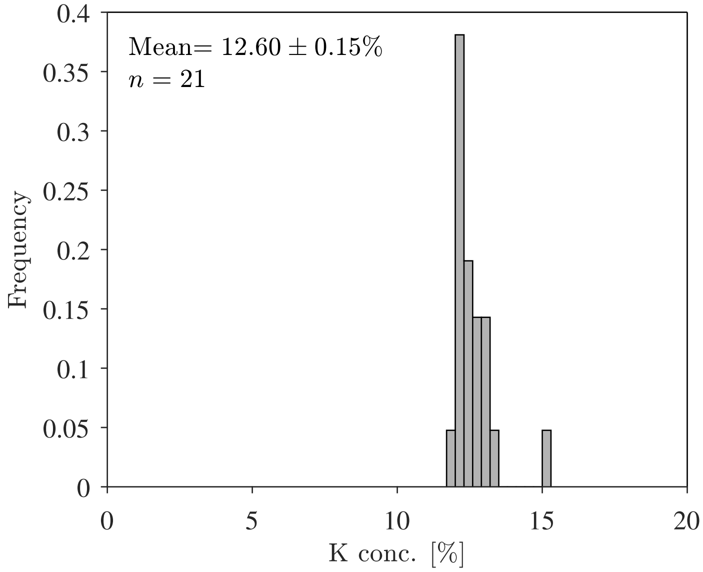

Figure SI.1: K concentration distribution measured with XRF on 7 KF extracts from samples 167805, -06, -12, -19, -20, -21, -22. Three multi-grain (2 mm) aliquots were measured for each sample.

The average saturated water content (w.c.) for each unit is summarised in Table SI.2. We assume a water content of 40±10% of the saturated value averaged for each deposition unit. The w.c. values employed in dosimetric calculations for individual samples are given in Table SI.2 ("Assumed"). In section: *Water content correction*, we examine the significance of this assumption on the OSL ages.

Several of the sediment samples, particularly samples 197332, 197328, 197333, 197340, 197338 and 197339, were taken in close proximity to bedrock. As the gamma dose rate from bedrock at this site is about three times lower than that from sediment (see Table SI.2), the gamma radiation field in the vicinity of at least these samples must be heterogeneous. This is likely to affect the dose rate experienced by the sample significantly and thus the effect must be taken into account when calculating the sample-specific gamma dose rate based on radionuclide concentrations (before adding internal dose rates, cosmic dose rates and accounting for water content and grain size attenuation).

This can for instance be done using Monte Carlo simulations [e.g., 39] or simple explicit models [e.g., 40]. In this study, the modifications for heterogeneity are based on modelling, and rely on the principle of superposition and the infinite matrix assumption[41]. Such model corrections are not as rigorous as those derived from Monte Carlo simulations, but they are considered adequate approximations and are more readily incorporated into age calculation software and spreadsheets.

Correction for heterogeneity in the gamma field

In general, the total gamma dose rate, $\dot{D}_{\text{total}}$, to a sample derives from the sample matrix itself, and from any material in the vicinity of the sample (in this case bedrock). Thus, the total gamma dose rate can be expressed as the sum of contributions from the external, $\dot{D}_{\text{ext,inf}}$, and internal $\dot{D}_{\text{int,inf}}$, infinite matrix dose rates, where the relative contributions are given by correction factors C_{int} and C_{ext}, i.e.,

$$\dot{D}_{\text{total}} = C_{\text{int}}\dot{D}_{\text{int,inf}} + C_{\text{ext}}\dot{D}_{\text{ext,inf}} \quad \text{(Eq. SI.1)}$$

To determine the appropriate correction factors C_{int} and C_{ext} in Eq. SI.1, we first derive the general equations for the simple case where the sample material is surrounded by another material in all directions (see section: *3D Correction factors for a simple case*). This model is then compared to the Monte Carlo simulation of Riedesel and Autzen[39]. Based on these general equations, we derive the correction factors relevant for this study, e.g., where a sample is surrounded by sediment on one side and bedrock on the other (section: *Correction factors for life-time burial*). To further test the model, the derived dose rates are compared with in situ dose rate measurements (section: *Comparison with in situ dose rate measurements*). Finally, the significance of our dose rate modelling is evaluated by comparing it to the unmodelled dose rate data (see section: *Effect of dose rate modelling in LRC*).

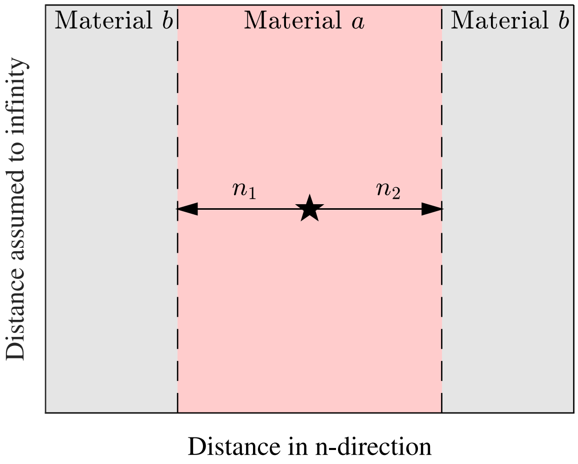

Figure SI.2: Schematic drawing of a simple 2D case, where material a (pale red) is surrounded by material b (grey) on both sides. Material b is assumed to be infinitely thick. The distances from the point of interest (the star) to the boundary between material a and b in direction n_i are shown as arrows.

To derive the correction factors C_{int} and C_{ext}, we use the principle of superposition as in Aitken[41]. For parallel infinite sheets (see Figure SI.2), $N_{a,b}$ describes the dose rate attenuation in material a in the n direction due to the active material b:

$$N_{a,b} = f_{b1}e^{-\mu_{a1}n_1} + f_{b2}e^{-\mu_{a2}n_2} \quad \text{(Eq. SI.2)}$$

Where f_{b1} and f_{b2} are the fractional dose rate factors for the active material b, and μ_{a1} and μ_{a2} are the attenuation factors in material a. The numbers 1 and 2 relate to the distances n_1 and n_2. The factors f_{b1}, f_{b2}, μ_{a1} and μ_{a2} depend both on the type of material and on the distances in the n-direction from the point of interest to the boundary to the active material b, e.g., n_1 and n_2 in Figure SI.2. In the same way the attenuation of the internal dose rate from material a itself is described by

$$1 - N_{a,a} = 1 - (f_{a1}e^{-\mu_{a1}n_1} + f_{a2}e^{-\mu_{a2}n_2}) \quad \text{(Eq. SI.3)}$$

In real situations, material a may be surrounded by b on more than two parallel sides and the net attenuation in material a is then more complicated. Below we develop a model describing such situations by using Eq. SI.2 and Eq. SI.3 for the x, y and z directions with N being X, Y or Z.

3D Correction factors for a simple case

In Figure SI.3, a material s (pale red) is surrounded by another material r (grey) in all directions. The grey material extends to infinity in all directions from the pale red material. The internal and external correction factors for this 3D geometry are derived below based on the principle of superposition.

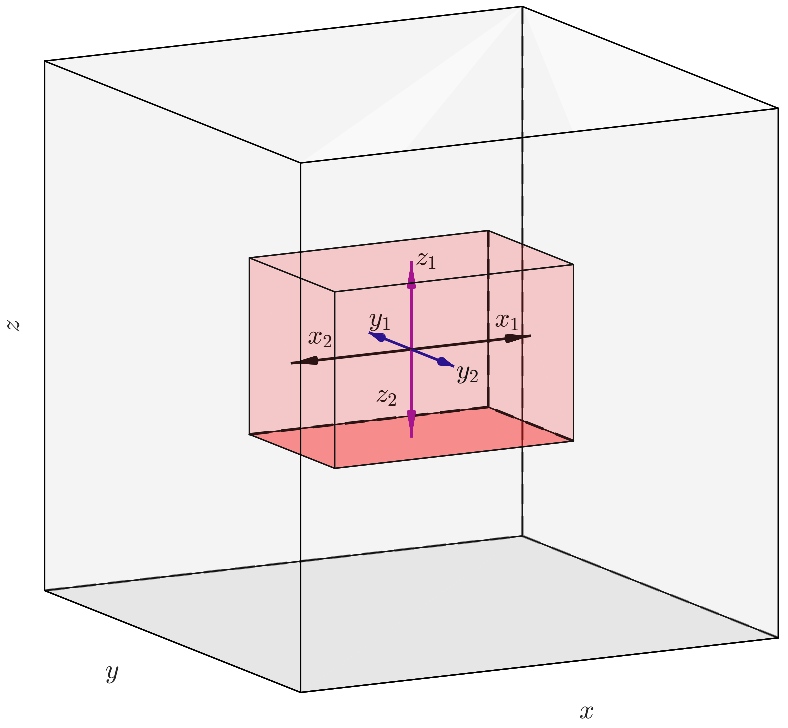

Figure SI.3: Material s (inner box: pale red) surrounded by material r (outer box: grey), on all sides. The grey material is assumed to continue to infinity. The distances from the point of interest (used as the origin of the coordinate system) to the boundary between material s and r in all directions are shown as arrows.

3D Internal correction factors

If material s is the active material, the correction factor, C_{int}, for the dose rate from material s to itself is

$$C_{\text{int}} = (1 - X_{s,s})(1 - Y_{s,s})(1 - Z_{s,s}) \qquad \text{(Eq. SI.4)}$$

where $(1 - X_{s,s})$, $(1 - Y_{s,s})$ and $(1 - Z_{s,s})$ are defined in Eq. SI.3 with $a = s$ and n_1 and n_2 being the distances from the point of interest to the outer boundaries of material s in the x, y, and z-directions (i.e., x_1, x_2, y_1, y_2, z_1 and z_2 in Figure SI.3).

In Figure SI.4, we test the validity of our mathematical formulation for the internal correction factor, C_{int}, by comparing the results obtained using Eq. SI.4 to those obtained by Riedesel and Autzen[(39)] using Monte Carlo simulation for a granite cobble. Riedesel and Autzen[(39)] used a spherical geometry for their calculations, so we have calculated the internal fractional dose rate using Eq. SI.4 at the centre of a cube with the same volume as that of the sphere. We use the same μ and f values for granite for the Th series as Riedesel and Autzen[(39)] did.

In this study, the critical samples (from the point of view of dose rate heterogeneity) are located at distances ranging between ~6 cm and 30 cm to the nearest bedrock. Most of the samples are placed >100 cm away from bedrock.

For diameters of ∼12 cm to ∼24 cm our calculation of the internal fractional dose rate underestimates the Monte Carlo simulations by less than 25% (see inset in Figure SI.4). For diameters ranging between 24 and 120, cm our model overestimates less than 10%. For larger diameters (>120 cm), the internal fractional dose rate is unity (i.e., the internal dose rate corresponds to the infinite matrix dose rate).
In this study, although some samples are located inside 12 cm diameter holes in the wall, they are not surrounded by bedrock from all sides and the underestimation compared to Monte Carlo simulation of the sphere is not critical for these samples.

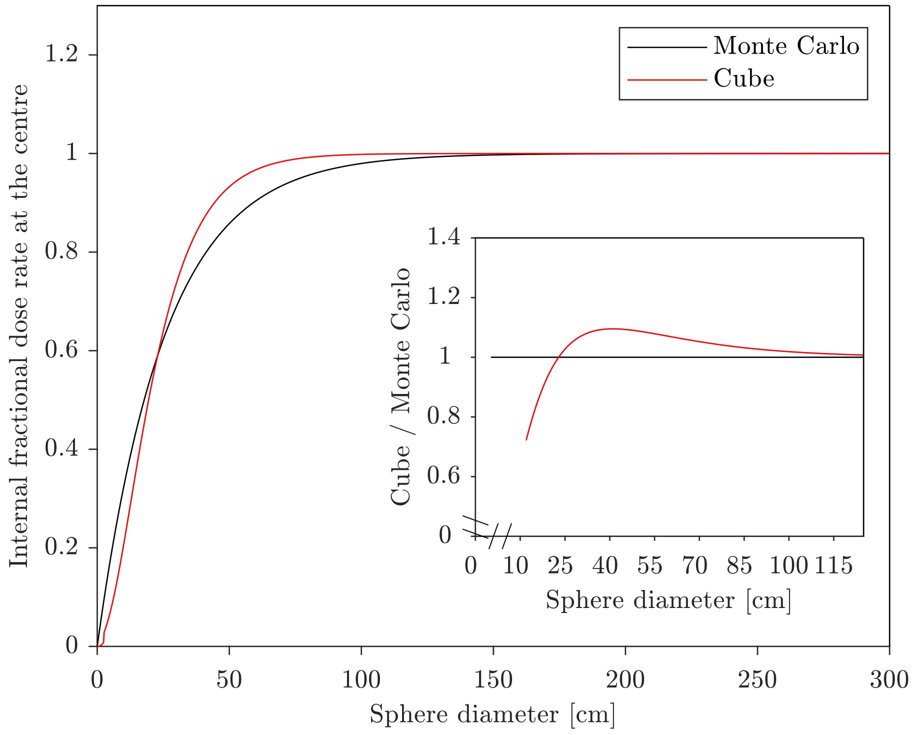

Figure SI.4: Internal fractional dose rate at the centre of a granite sphere simulated using Monte Carlo (data reproduced from Riedesel and Autzen [39], black line). Internal fractional dose rate calculated using Eq. SI.4 (red line) at the centre of a cube with the same volume as that of a sphere. Values for μ and f are taken from Riedesel and Autzen [39] for Th. The corresponding curves for U and K are similar to the ones shown here for Th. The inset shows the ratio of the red to black line for diameters ranging from 12 cm to 125 cm.

3D external correction factors

Now letting the surrounding material r being the active material, the correction factor for the external dose rate, C_{ext}, from material r to material s is given by Eq. SI.5 below.

$$C_{\text{ext}} = X_{s,r} + (1 - X_{r,r})Y_{s,r} + (1 - X_{r,r})(1 - Y_{r,r})Z_{s,r} \qquad \text{(Eq. SI.5)}$$

where $X_{s,r}$, $Y_{s,r}$ and $Z_{s,r}$ given by Eq. SI.2 and $(1 - X_{r,r})$ and $(1 - Y_{r,r})$ by Eq. SI.3 with x_1, x_2, y_1, y_2, z_1 and z_2 defined as for the internal correction factor (see Figure SI.3).

The terms $(1-X_{r,r})$ and $(1-X_{r,r})(1-Y_{r,r})$ in the second and third terms, respectively, have been included to avoid double counting. This is a consequence of the fact that some parts are geometrically included in the calculations in more than one direction if the sum of $X_{s,r}$, $Y_{s,r}$ and $Z_{s,r}$ were used instead of Eq. SI.5.
The issue of double counting is illustrated for a 2-dimensional case in Figure SI.5. Here material s (pale red) is surrounded by active material r (grey) in x- and y-directions. Material r is assumed to continue to infinity. The contributions from the external dose rate from material r in the x-direction comes from the two full line-striped areas (east/west of material s in Figure SI.5), including the corners, and are given from Eq. SI.2 as $X_{s,r} = f_{r1}e^{-\mu_{s1}x_1} + f_{r2}e^{-\mu_{s2}x_2}$.
The contributions in the y-directions come from the two broken line-striped areas (north/south of material s in

Figure SI.5), but the corners (striped with both full and broken lines) were already accounted for in $X_{s,r}$. The contribution in the y-direction must therefore be corrected for this, by an "internal" correction for the material r in the x-direction, given as $(1 - X_{r,r})$ with μ and f defined for material r.

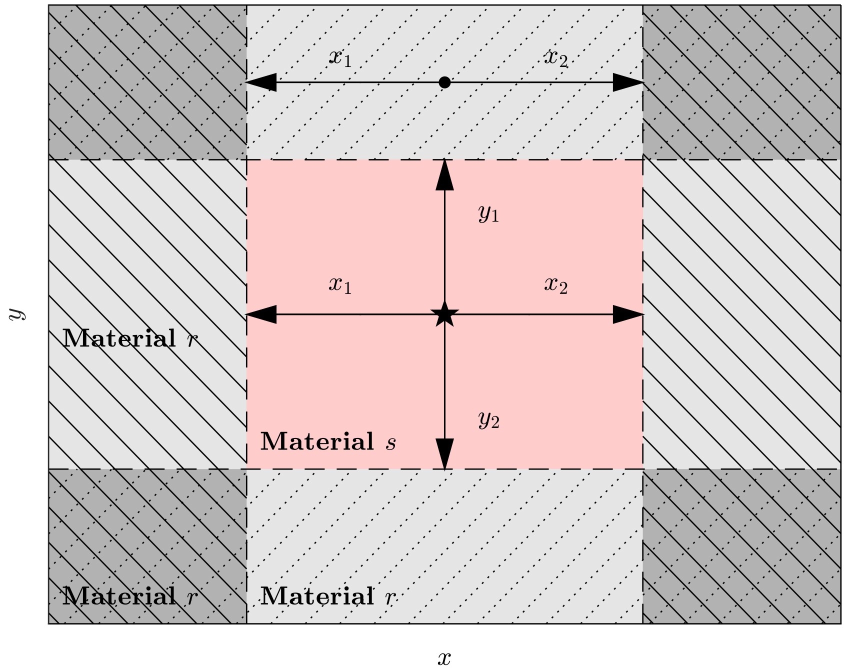

Figure SI.5: Illustration of material s (red) surrounded by active material r (grey) in all directions (assumed to continue to infinity) in two dimensions. The contributions from the external dose rate to material s from material r in the x-direction come from the two striped areas (including the corners). The contributions in the y-direction comes from the two broken striped areas, but the corners (striped and broken striped) must be omitted such that they are only counted once.

For the 3-dimensional case, this "internal" correction (in regard to material r itself) in the 3rd dimension is also necessary because two parts of the volume contributing in the z-direction have already been accounted for in the x- and y-directions, respectively. This results in the last term in Eq. SI.5 $(1 - X_{r,r})(1 - Y_{r,r})Z_{s,r}$. This is illustrated in Figure SI.6, where the external contributions to the red volume in the x-, y- and z-directions come from the white, blue, and green volumes respectively, corresponding to the first, second, and third term in Eq. SI.5, respectively. Notice how the contribution in the x-direction (from the white volume) includes regions that would otherwise be counted as contributing in the y- and z-directions if the sum of $X_{s,r}$, $Y_{s,r}$ and $Z_{s,r}$ was used instead of Eq. SI.5. Similarly, the contribution in the y-direction (from the blue volume) includes regions that would otherwise be counted in the z-direction.

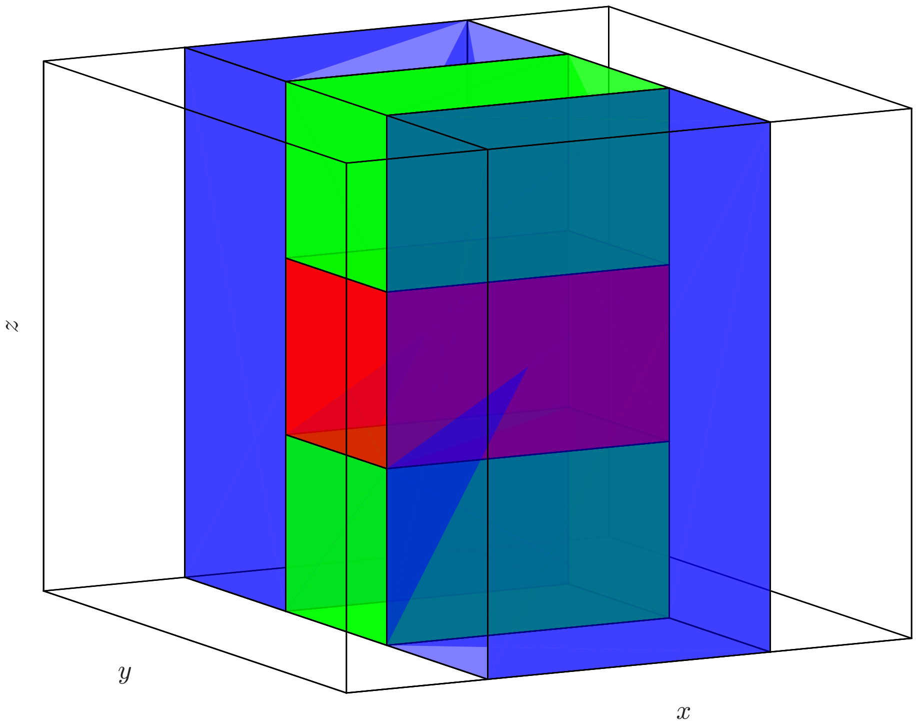

Figure SI.6: Illustration of the geometry relevant for external dose rate calculations to material s (red volume). The external material r (white, blue, and green volumes) all contribute to the total external dose rate. The white volume has already been included, and so is subtracted from the contribution in the y-direction (blue volume). Similarly, the white and blue volumes have already been included, and so must be subtracted from the contribution in the z-direction (green volume, see Eq. SI.5).

The total dose rate to the sample in this simple symmetrical case is then given as the sum of external and internal infinite matrix dose rates, Eq. SI.1, both corrected for attenuation by the correction factors C_{int} and C_{ext} from Eq. SI.4 and Eq. SI.5, respectively

$$\dot{D}_{\text{total}} = C_{\text{int}} \dot{D}_{\text{int,inf}} + C_{\text{ext}} \dot{D}_{\text{ext,inf}} \qquad \text{(Eq. SI.6)}$$

Correction factors for life-time burial

In this study, the sediments samples were not surrounded by bedrock in all directions. During burial, sediments are assumed to fill the space on one side of the sample (see Figure SI.7). This gives an extra term in the correction factors C_{int} and C_{ext} compared to the simple case described above.
If x_1 is the distance from the middle of the sample volume to this main sediment body (see Figure SI.7), the correction factor for the internal dose rate, C_{int}, in the sediment volume due to the sediment s itself is

$$C_{\text{int}} = (1 - X_{s,s})(1 - Y_{s,s})(1 - Z_{s,s}) + f_{s1} e^{-\mu_{s1} x_{s1}} \qquad \text{(Eq. SI.7)}$$

where $(1 - X_{s,s})$, $(1 - Y_{s,s})$ and $(1 - Z_{s,s})$ are defined in Eq. SI.3 with μ_{s1} being the attenuation factor in sediment and x_1, x_2, y_1, y_2, z_1 and z_2 define the boundaries of the sediment volume from the point of interest (see Figure SI.7).

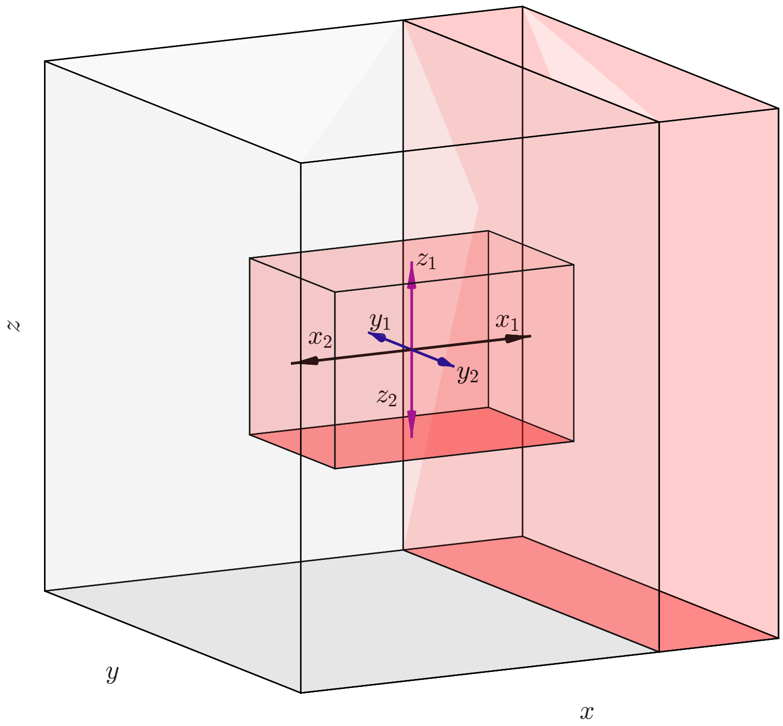

Figure SI.7: Illustration of the 3D geometry relevant for some samples dated in this study. Sediment is shown in pale red and bedrock in grey. During burial, sediments are assumed to fill the space in front of the sample hole to infinity. All outer boundaries in the figure are assumed to go to infinity.

The correction factor C_{ext}, for the external dose rate from bedrock material, r, to the sediment volume, is derived from Eq. SI.5. But that formulation assumed the bedrock to be on both sides of the sample; in this case it is only on one side, and so this extra contribution must be subtracted:

$$C_{\text{ext}} = X_{s,r} + (1 - X_{r,r})Y_{s,r} + (1 - X_{r,r})(1 - Y_{r,r})Z_{s,r} - f_{r1}e^{-\mu_{s1}x_{s1}} \quad \text{(Eq. SI.8)}$$

For sediment samples with no bedrock nearby (i.e., more than 30 cm away), C_{int} and C_{ext} effectively become 1 and 0 respectively, and the infinite matrix dose rate can be applied without correction.

In the derived corrections factors the parameters f_s, f_r, μ_s and μ_r are different for K-, Th- and U-series and thus each series must be corrected individually. By using Eq. SI.7 and Eq. SI.8 in Eq. SI.1, dry dose rates corrected for nearby rock material are obtained.

The bedrock material in this site is sandstone and the value for μ_r for the bedrock material and the value for μ_r for sediment material are calculated using the sediment values of Riedesel and Autzen [39], corrected for the density of the sandstone measured to 1.3 g.cm^{-3} and sediment measured to 1.8 g.cm^{-3}. The values used are summarised in Table SI.3 and SI.4, respectively.

Table SI.3: Beta and gamma attenuation factors (μ) and the fractional dose factor (f) for different distances (d) in sandstone with density 1.3 g.cm^{-3}, to the boundary of the active material. Adapted from Riedesel and Autzen [39] after density correction.

**Bedrock**	Beta			Gamma		
Decay chain	μ [mm^{-1}]	f	d [mm]	μ [mm^{-1}]	f	d [mm]
^{40}K	1.83	0.50	all	0.014	0.50	<10
				0.009	0.45	>10
^{232}Th	3.03	0.50	<0.15	0.013	0.50	<10
	1.20	0.34	>0.15	0.009	0.42	>10
^{238}U	2.29	0.50	<0.15	0.018	0.50	<10
	0.98	0.37	>0.15	0.010	0.45	>10

Table SI.4: Beta and gamma attenuation factors (μ) and the fractional dose factor (f) for different distances (d) in sediment with density 1.8 g.cm^{-3}, to the boundary of the active material. Adapted from Riedesel and Autzen [39] after density correction.

**Sediment**	Beta			Gamma		
Decay chain	μ [mm^{-1}]	f	d [mm]	μ [mm^{-1}]	f	d [mm]
^{40}K	2.53	0.50	all	0.019	0.50	<10
				0.013	0.45	>10
^{232}Th	4.19	0.50	<0.15	0.018	0.50	<10
	1.66	0.34	>0.15	0.013	0.42	>10
^{238}U	3.17	0.50	<0.15	0.024	0.50	<10
	1.35	0.37	>0.15	0.014	0.45	>10

In Figure SI.8, the two-dimensional life-time geometry is illustrated for a) a sample taken close to a cave wall and b) for a sample taken inside a hole in the cave wall. The distances from the centre of the sample hole (marked with a star) to the sediment boundary in the y directions are assumed to be infinite in a) and 20 cm in b). The latter is chosen to show the effect on samples taken inside holes in the cave wall.
Gamma correction factors vary as a function of distance in the x-direction corresponding to different positions in the sample hole (rectangle). Correction factors for both the internal dose rate (red) and the external dose rate (blue) are shown.

If the sample position is at the centre of the sample hole (at x =15 cm, i.e., the location of the star) the gamma correction factors are 0.93 and 0.07 for sediment and rock, respectively for the geometry in Figure SI.8a and 0.88 and 0.12 for the geometry in Figure SI.8b where the sample is located in a hole in the bedrock material.
The correction factors for beta irradiation are essentially unity for the sediment contribution, and zero for the bedrock contribution for all distances from the boundary relevant to this study because of the short range of the beta particles.

The dose rates used for age calculation are given in Table SI.2 as "Total dose rates". These are based on beta and modelled gamma dose rates, both adjusted for water content and grain size attenuation, and include internal dose rates and a cosmic ray contribution. The latter has been calculated using the depths reported in the excavation report from 1846 [2]; these are likely to represent the life-time burial depth more accurately than the present-day burial depths, since a considerable amount of sediment has been removed from the slope during the last 150 years.

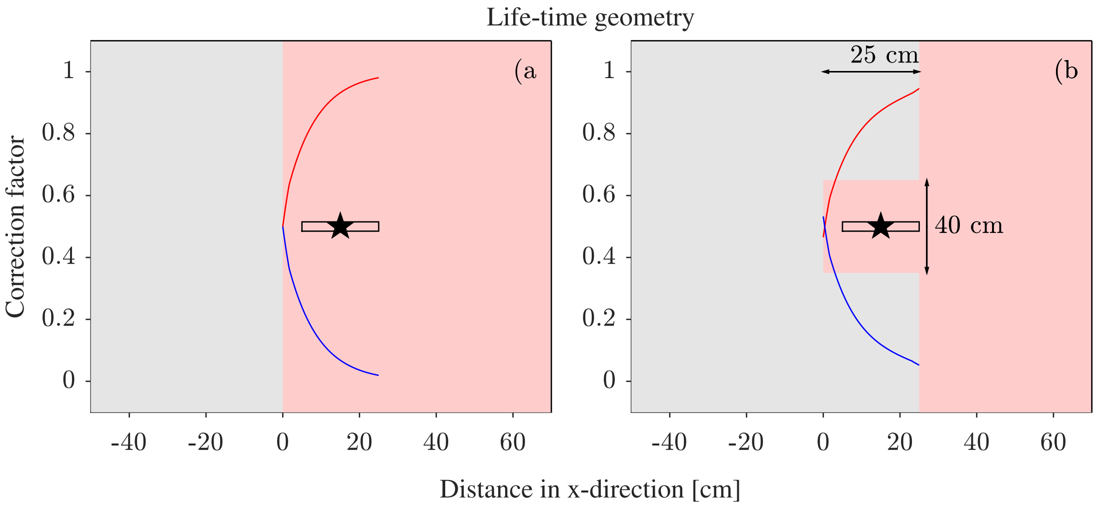

Figure SI.8: Gamma correction factors for two typical life-time geometries a) a sample taken close to a cave wall and b) for a sample taken inside a hole in the cave wall. Correction factors for the internal sediment gamma dose rate, C_{int} is shown in red, whereas that for the external bedrock dose rate, C_{ext} is shown in blue for x varying between 0 and 25 cm for a fixed y position. Pale red represents sediment whereas bedrock is represented in grey. Both materials are assumed to be infinite in the z-direction and assumed to be infinite outside the ranges shown on the axes. The rectangle in the centre illustrates the volume from which the sample is taken. The star illustrates the position from which the correction factors for age calculations are derived, i.e., in the middle of the sample tube.

Comparison with in situ dose rate measurements

The gamma dose rates derived from radionuclide concentrations, corrected for heterogeneity in the gamma field (section: *Correction factors for life-time burial*), current water content and cosmic dose rate are compared with the in situ dose rate measurements made with a LaBr probe and Al_2O_3:C pellets (see section: *Instrumentation*).

To enable comparison between the in situ dose rate measurements and the modelled gamma dose rates, the correction factors have to be modified slightly to take the different excavation geometry into account (see Figure SI.9). The excavation geometry, which applies to the in situ measurements, is different from the life-time geometry, because the in situ dose rate measurements were made by inserting the pellets or probe in the holes left after sampling, i.e., there is no sediment inside the sample hole, the in situ measurements were made at the end of the sample hole and most importantly there was no sediment in front of the sample hole.

The location at the end of the sample hole H (see star in Figure SI.9), receives radiation from the surrounding sediment in volume S and from the surrounding bedrock material. The two corrections factors due to these dose rates acting at the end point of the sample hole is given by Eq. SI.9 and Eq. SI.10

$$C_{\text{int}} = I_s(S) - I_s(H) \qquad \text{(Eq. SI.9)}$$

$$C_{\text{ext}} = E_{s(r)}(H) - f_{r1}e^{-\mu_{s1}x_{1s,\text{hole}}} \qquad \text{(Eq. SI.10)}$$

with $I_s(S)$ and $I_s(H)$ defined as internal correction factors of the sediment geometry and the hole geometry respectively (Eq. SI.11 and Eq. SI.12 below). $E_{s(r)}(H)$ is defined as an external correction factor with respect to the hole geometry (Eq. SI.13 below).
S refers to the distances to the outer sediment boundaries (i.e., the distances from the star to the outer boundary of the pale red area in Figure SI.9. H refers to the distances to the boundary of the hole (i.e., the distances from the star to the outer boundary of the sample hole in Figure SI.9).

$$I_s(S) \equiv (1 - X_{s,s}^{\text{sed}})(1 - Y_{s,s}^{\text{sed}})(1 - Z_{s,s}^{\text{sed}}) \qquad \text{(Eq. SI.11)}$$

$$I_s(H) \equiv (1 - X_{s,s}^{\text{hole}})(1 - Y_{s,s}^{\text{hole}})(1 - Z_{s,s}^{\text{hole}}) \qquad \text{(Eq. SI.12)}$$

$$E_{s(r)}(H) \equiv X_{s,r}^{\text{hole}} + (1 - X_{r,r}^{\text{hole}})Y_{s,r}^{\text{hole}} + (1 - X_{r,r}^{\text{hole}})(1 - Y_{r,r})Z_{s,r}^{\text{hole}} \qquad \text{(Eq. SI.13)}$$

The red and blue lines in Figure SI.9 (C_{int} and C_{ext}, respectively) show the corrections factors for different lengths of the sample hole (i.e., varying the length of the white rectangle). The white rectangles illustrate the sample hole

and the star shows the position that was used in calculation of the correction factor. The in situ dose rate meter is assumed to have been positioned where indicated by the star (i.e., at the end of the sample hole).

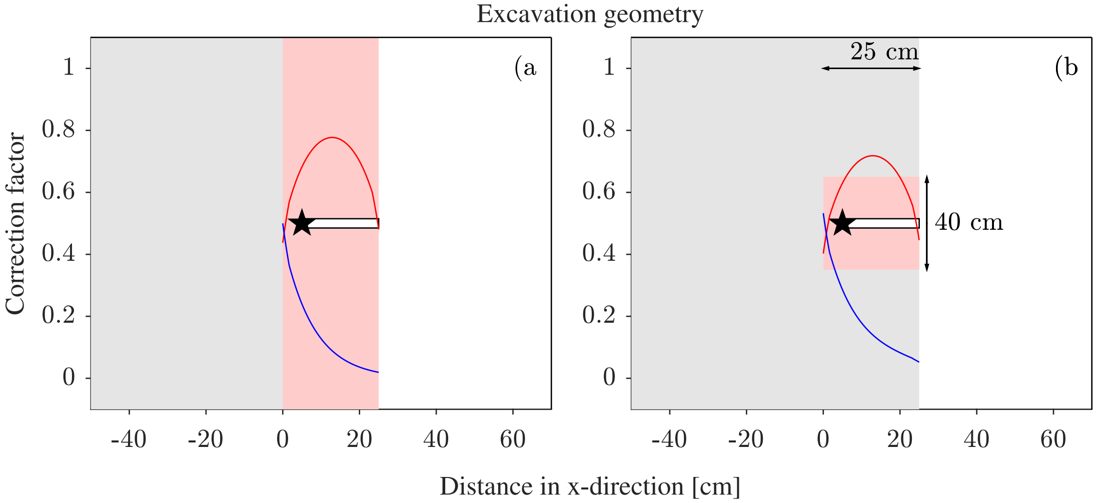

Figure SI.9: Gamma correction factors relevant to the present-day site (excavated geometry) for two different geometries: a) a sample taken close to a cave wall and b) for a sample taken inside a hole in the cave wall. Correction factors for the internal sediment gamma dose rate, C_{int} is shown in red, whereas that for the external bedrock dose rate, C_{ext} is shown in blue. The sample position is assumed to be at the end of the sample hole (where the star is shown) the depth of which varies between 0 and 25 cm. Pale red represents sediment, grey represents bedrock, white represents air. All materials are assumed to be infinite outside the axis ranges shown. The white rectangle illustrates a 20 cm long sample hole. The star illustrates the position from which the correction factors are derived, i.e., at the end of the sample hole.

By using Eq. SI.9 and Eq. SI.10, corrected laboratory calculated wet gamma dose rates are derived for comparison with in situ dose rate measurements.

In situ dose rate measurements were done using two different methods. i) Al_2O_3:C pellets were placed in four sediment sample positions and in one hole in the cave wall (black circles in Figure SI.10). ii) LaBr probe measurements were undertaken in two sediment sample positions and in one hole in the cave wall (red triangles in Figure SI.10). Since the LaBr probe does not record the cosmic dose rate, this term has not been included in the laboratory dose rate for the purpose of this comparison (red triangles in Figure SI.10).

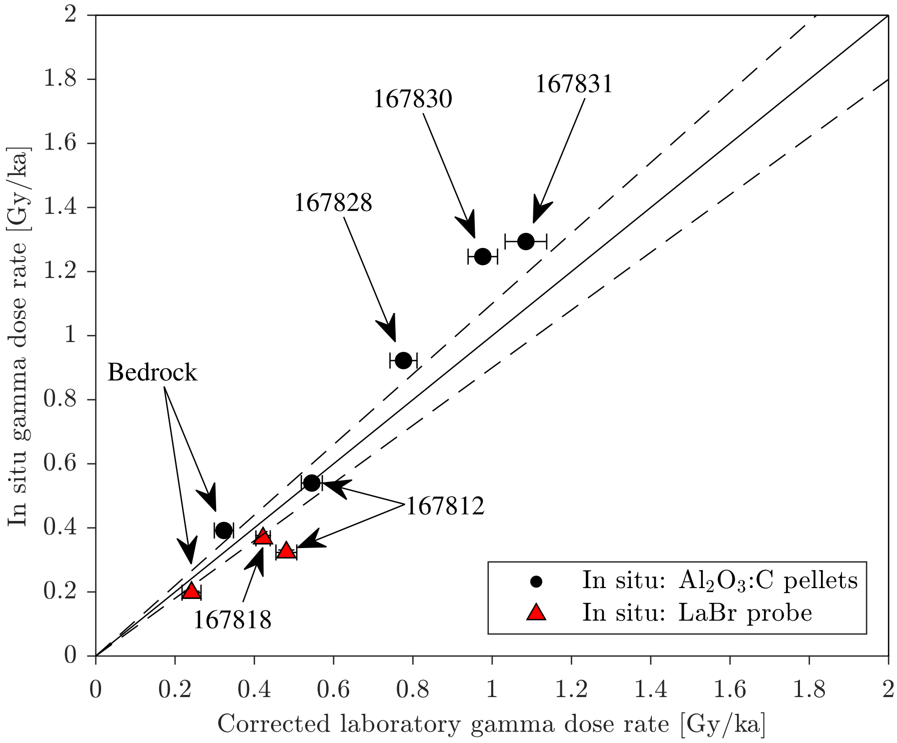

Figure SI.10: In situ measured gamma dose rates from a LaBr probe (red triangles) and Al_2O_3:C pellets (black circles) compared to wet (current w.c.) gamma dose rates derived from radionuclide concentrations and corrected for nearby rocks using Eq. SI.9 and Eq. SI.10. The cosmic dose rate is not included in the laboratory gamma dose rate and in the Al_2O_3:C pellets, since the LaBr probe does not measure cosmic dose rate. The numbers indicate the corresponding sediment sample number. Also shown is the 1:1 line (solid line) with a ±10 % uncertainty (dashed lines).

On average, the results from the Al_2O_3:C pellets tend to overestimate the calculated dose rates, whereas the LaBr probe results tend to underestimate. Note that both sample 167818 and the rock sample are measured using both in situ methods. The two in situ methods are not consistent with each other, and the corrected calculated dose rates lie between the two in situ methods. We conclude that there is no evidence that the in situ field dose rate measurements are more accurate than the dose rates from radionuclide concentrations (corrected for heterogeneity).
The mean ratio of corrected laboratory dose rate to in situ dose rate is 1.02 ± 0.09 (n=8) for the eight sample positions under consideration (Figure SI.10). This indicates that the laboratory corrected dose rate are, on average, indistinguishable from the measured values, and that the correction for heterogeneity is likely to give reliable dose rates when applied to the burial conditions assumed to apply before the beginning of excavation in 1846.

Effect of dose rate modelling in LRC

The effect of the dose rate modelling is illustrated using the ratio of the corrected dose rate to the infinite matrix dose rate (see Table SI.2) for both the dry gamma dose rate (R_{corr}) and the total dose rate (R_{tot}). R_{corr} varies between 0.52 and 1, whereas R_{tot} varies between 0.80 and 1. R_{tot} for quartz is on average 0.961 ± 0.009 (n=50), i.e., close to unity because for most samples the sediment volume is effectively infinite and thus the dose rate for these samples do not require correction. In Figure SI.11 we plot R_{corr} (open squares) and R_{tot} (closed circles) for each sample. For 42 of the 50 samples, R_{tot} is >0.93, but for eight samples (197328, -32, -33, -38, -39 and -40, and 227801, -02) the correction for total dose rate is ~20% and significant. Samples 197328, -32, -33, -38, -39 and -40 are all taken from holes in the cave wall with a diameter of ~12 cm and length of ~15 cm, but assuming an infinite sediment body. Sample 227801, -02 are taken from small cavities with distances to nearby bed rock material close, or less than, 30 cm. Samples 197332, -28 and -33 and 227801 an 227802 are particularly important for determining the chronology for the closure of LRC I.

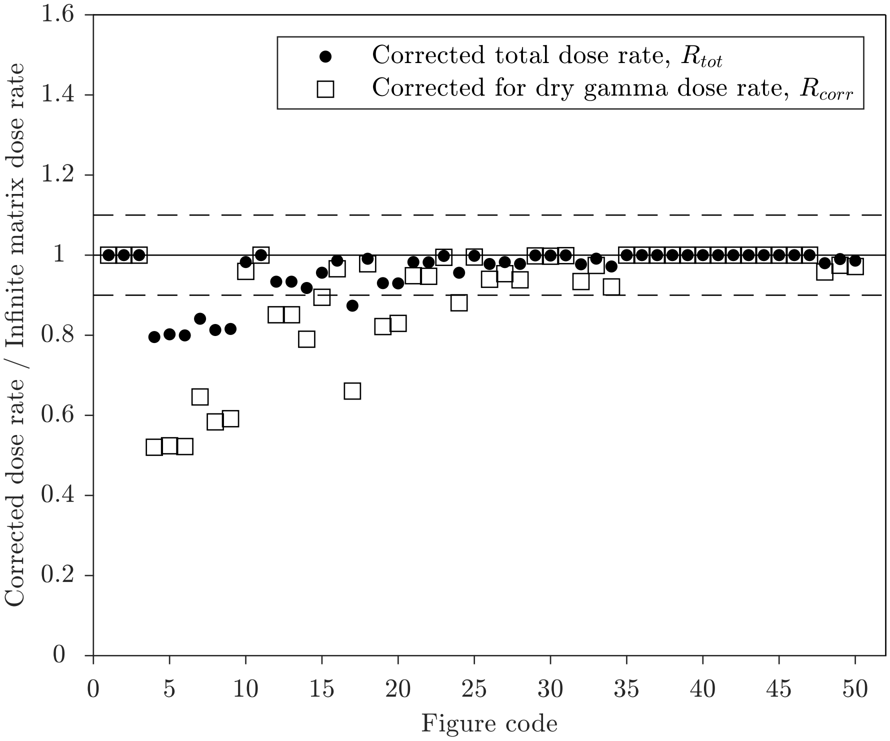

Figure SI.11: Ratio of corrected to infinite matrix dose rates for total dose rate to quartz (R_{tot}, black circles) and to dry gamma dose rate (R_{corr}, squares), plotted against figure codes (see Table SI.2). Six samples (197332, 197328, 197333, 197340, 197338, 197339, 227801, and 227802 (Figure code 4-9, 14, 17 in Fig. 6 and 7) have R_{tot} values below 0.93 and below 0.7 for R_{corr}. These samples were all taken from holes (diameter of ~12 cm and length of ~15 cm) or small cavities in the cave wall and clearly illustrate the necessity of correcting for heterogeneity in the gamma field in such cases.

Our dose rate correction model has been tested against the Monte Carlo simulations of Riedesel and Autzen [(39)] (see Figure SI.4) and against in situ dose rate measurements. From the results, we conclude that we have confidence in our corrections for heterogeneity in the dose rate.

Our dose rate modelling is simple to implement, does not require Monte Carlo simulations, and can be readily incorporated into age calculation software and spreadsheets. It has considerable potential in sites such as LRC where the heterogeneity is significant and the position of the bedrock material can be most easily categorised by distances in the six directions from the sample position (front, back, left, right, top, bottom).

The dose rates used for age calculation are given in Table SI.2 as "Corrected total wet dose rates". These are based on infinite matrix beta dose rates and modelled gamma dose rates, both adjusted for water content and grain size attenuation, and include internal dose rates and a cosmic ray contribution calculated using reported (1846) burial

depths[2] (i.e., life-time burial depths).

Multi-grain Quartz luminescence results

Multi-grain quartz dose response and stimulation curves

To investigate whether multi-grain quartz measurements are suitable for OSL analysis the OSL signal characteristics were first analysed. Figure SI.12 (data from sample 167828) serves as an illustration of the basic OSL characteristics displayed by the quartz multi-grain measurements. The main graph shows a typical DRC, while the inset shows the natural OSL stimulation curve from the same aliquot. Also shown is the OSL stimulation curve from Risø calibration quartz[8], which is known to be dominated by the fast component. These stimulation curve shapes are very similar, implying that the OSL signals from these sedimentary samples are fast-component dominated. The main graph shows a typical quartz dose response curve with a recycling value of 0.96 ± 0.03, a recuperation value of 0.06 ± 0.01 Gy, and an IR depletion ratio of 0.96 ± 0.03. The DRC has a D_c value of 96 ± 4 Gy (average D_c is 114.4 ± 1.2, n=1251).

Thus, these multi-grain quartz measurements generally show that the dose response curve of this sedimentary quartz appears to be suitable for OSL analysis and that we can have confidence in our ability to measure multi-grain quartz doses up to approximately 225 Gy (i.e., $\sim 2D_c$).

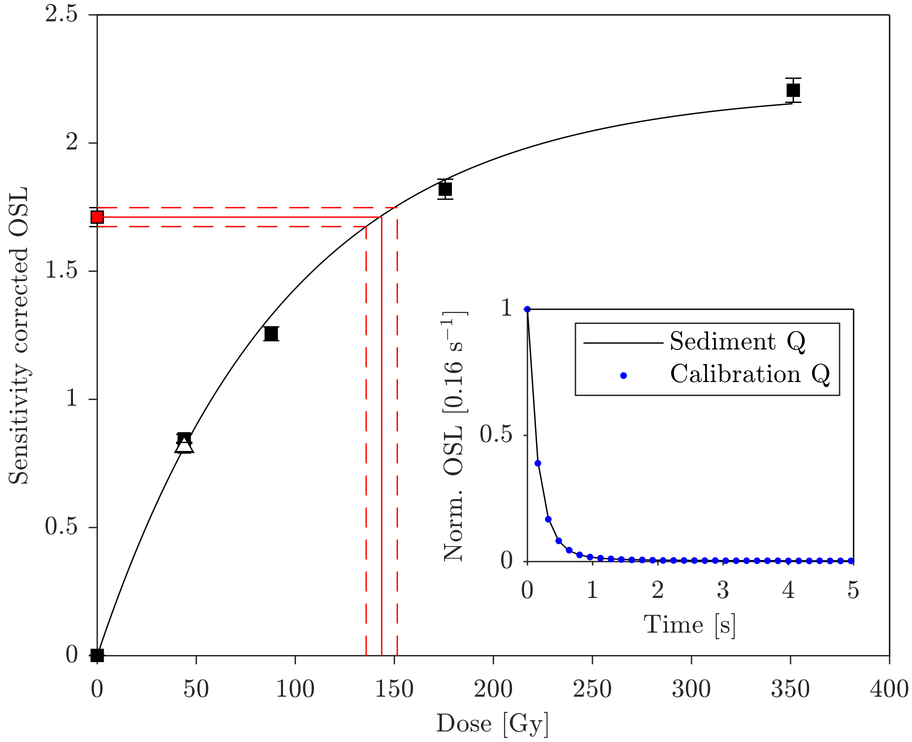

Figure SI.12: A typical quartz dose response curve (black squares) from sample 167828 fitted with a single saturating exponential function (black line). The sensitivity corrected natural signal (red square) is interpolated onto the dose response curve to obtain the estimate of equivalent dose. The recycling point is shown as an open triangle. The insert shows the initial 5 s of the natural OSL signal from the same aliqout (black line), as well as the OSL signal from calibration quartz (blue dots).

Multi-grain quartz rejection criteria

It is standard practice to test [e.g., 42]: 1) if the chosen measurement protocol accurately corrects for sensitivity change (i.e., the recycling ratio should be close to unity), 2) if the quartz signal is affected by presumed feldspar contamination (i.e., the IR depletion ratio should be close to unity), and 3) if there is significant carry-over between individual SAR measurement cycles (i.e., recuperation should be close to zero). For these samples, the average

recycling ratio is 0.957 ± 0.002 (n=840), the average IR depletion ratio is 0.967 ± 0.002 (n=453) and the average recuperation is $0.132 \pm 0.012\%$ of the relevant natural signal (n=840). Based on this we conclude that the overall laboratory sample characteristics are satisfactory. However, in the literature it has been argued that individual aliquots must be discarded if the individual recycling and/or IR depletion ratios are significantly different from unity, i.e., the dose estimate from aliquots where, at least, one of these ratios is more than two standard deviations away from unity, should be rejected.

In Figure SI.13 and SI.14, we show the individual dose estimates for 840 aliquots as a function of the corresponding recycling and IR depletion ratio, respectively. The individual doses have been normalised to the mean for each sample. There is no correlation between these quantities and the measured dose, and applying such rejection criteria does not significantly change the average dose or the relative standard error (RSE) at the 95% confidence level. Thus, there is no justification for applying such rejection criteria to these samples [(42)].

As stated in section: *Multi-grain quartz OSL measurements*, we have used the objective Inter quartile Range (IQR) criteria to reject outlying dose estimates. The natural quartz multi-grain dose distributions are shown as simple frequency histograms in Figure SI.16 with the rejected doses indicated. Applying the IQR criterion (to samples processed in Denmark) rejects a total of 46 dose estimates for all samples out of the 1190 derived, i.e., ∼4% are rejected. The ratio of dose calculated with and without the IQR for individual samples range between 0.87 ± 0.09 and 1.07 ± 0.08 (see Table SI.5) with an average for all samples of 0.980 ± 0.005 (n=43 samples), and the ratio for all individual samples is consistent with unity at 95% confidence. However, the average relative standard error (at 68 % level) is reduced by 16 ± 3 % (n=43 samples). Thus, application of the IQR results mainly in a more precise dose estimate but does not significantly change the average dose values.

Table SI.5

					Multi-grain quartz				Multi-grain K-feldspar								Ratios				
									IR_{50}				$pIRIR_{290}$				Age		IQR ratio		
Lab code	Fig. code	Locus	Unit	Elevation [m]	Dose [Gy]	Q age [ka]	n**	In sat (%)	Dose [Gy]	KF age [ka]	n**	In sat (%)	Dose [Gy]	KF age [ka]	n**	In sat (%)	IR_{50}/Q	$pIRIR_{290}$/Q	Q	IR_{50}	$pIRIR_{290}$
187301	**1**	LRC I-d	1	54.95	79±3	42±3	19	0	52±4	24±3	8	0	100±4	36±3	7	0	0.56±0.07	0.85±0.06	1.07	1	0.96
187302	**2**	LRC I-d	1	54.55	129±6	47±3	35	0	68±7	24±3	8	0	136±4	37±2	9	0	0.51±0.06	0.78±0.05	1	1	1
187303	**3**	LRC I-d	1	54.10	97±6	46±4	20	0	37±7	16±3	4	0	104±7	34±3	8	0	0.34±0.06	0.74±0.07	1	1	1.11
197332	**4**	LRC I-c	1	51.28	105±3	55±3	34	0	57±2	26±2	10	0	142±5	50±4	12	0	0.46±0.04	0.91±0.05	0.87	0.92	1
197328	**5**	LRC I-c	1	51.10	97±3	50±3	19	0	56.8±1.8	25±2	11	0	138±4	48±3	12	0	0.51±0.04	0.96±0.05	0.98	0.95	1
197333	**6**	LRC I-c	1	50.97	101±4	53±3	35	0	61.5±1.9	28±2	11	0	137±3	48±3	11	0	0.52±0.04	0.91±0.05	0.98	1.03	0.97
197340	**7**	LRC I	E.C.	50.78	449±69	317±53	4	69	346±57	195±39	11	8	1347±199	571±95	6	50	0.61±0.16	1.8±0.4	1	1	1
197338	**8**	LRC I	E.C.	50.66	228±19	167±18	40	17	404±20	233±23	10	0	1474±63	638±57	5	58	1.39±0.18	3.8±0.4	1	0.90	1
197339	**9**	LRC I	E.C.	50.57	320±11	247±20	33	25	405±19	241±22	12	0	1868±86	834±78	10	17	0.97±0.09	3.4±0.2	1.05	1	1
167806	**10**	LRC I-a	2	50.54	132±6	58±4	22	0	86±3	34±2	11	0	182±7	56±4	12	0	0.60±0.05	0.98±0.07	0.93	0.91	1
167809	**11**	LRC I	I.C.	50.20	321±26	197±20	14	32	317±22	163±16	12	0	1330±54	517±44	10	17	0.83±0.10	2.6±0.2	0.91	1	1
167817	**12**	LRC I	I.C.	50.20	311±49	204±34	14	42	263±39	141±26	11	0	802±82	325±41	8	25	0.69±0.17	1.6±0.3	1	0.90	0.86
167818	**13**	LRC I	I.C.	50.20	449±22	295±23	13	42	398±16	214±19	11	0	1786±59	725±60	9	25	0.73±0.08	2.46±0.16	1.04	0.96	1
227801	**14**	LRC I	4(E.C.)	50.05	154±6	58±4	23	4	94.7±1.6	22.3±1.9	14	0	181.5±1.9	51±3	14	0	0.38±0.04	0.87±0.04	1	1	1
167805	**15**	LRC I-a	4	50.04	137±6	66±4	21	8	100±6	43±4	12	0	214±10	71±6	12	0	0.64±0.06	1.07±0.07	0.97	1	1
187312	**16**	LRC I-a	2	49.79	155±4	69±4	34	3	85±11	34±5	5	0	141±4	44±3	8	0	0.50±0.08	0.64±0.03	0.98	1	0.97
227802	**17**	LRC I	4(E.C.)	49.71	146±5	63±4	23	4	95±3	22.5±1.8	13	0	188±4	58±4	13	0	0.36±0.03	0.92±0.05	1	0.93	0.94
187311	**18**	LRC I-a	2	49.55	135±5	64±4	38	5	66±18	28±8	4	0	130±10	43±4	9	0	0.43±0.12	0.67±0.06	0.96	1	1
161267*	**19**	LRC I-b	4	49.40	163±7	79±5	24	-	-	-	-	-	-	-	-	-	-	-	-	-	-
167812	**20**	LRC I-b	4	49.40	134±8	76±6	22	4	78±6	37±3	12	0	175±14	65±7	12	0	0.49±0.05	0.85±0.09	0.92	1	1
187321	**21**	LRC II	3	47.59	112±3	88±5	34	0	69±3	40±3	12	0	156±5	70±5	12	0	0.45±0.04	0.80±0.04	0.96	1	1
187322	**22**	LRC II	3	47.18	122±3	95±6	33	0	75±4	44±4	12	0	147±4	66±5	12	0	0.46±0.04	0.69±0.04	0.94	1	1
187320	**23**	LRC II	3	47.09	159±5	84±5	35	0	89±3	41±3	11	0	166±3	59±4	12	0	0.48±0.04	0.70±0.03	0.98	0.96	1
187323	**24**	LRC II	5	46.90	159±6	121±8	34	3	99±3	57±5	23	0	256±13	113±10	24	0	0.47±0.04	0.94±0.07	0.95	0.98	1
187310	**25**	LRC II	3	46.76	132±4	88±5	23	0	78±4	41±3	9	0	151±4	62±4	9	0	0.47±0.04	0.70±0.03	0.98	1	1
187306	**26**	LRC II	3	46.64	121±4	87±5	33	6	73±5	41±4	9	0	149±5	64±5	9	0	0.47±0.05	0.74±0.04	0.98	1	1
187309	**27**	LRC II	3	46.03	113±4	103±9	23	4	73±2	46±4	9	0	137±4	67±6	9	0	0.45±0.04	0.65±0.03	1	1	1
187305	**28**	LRC II	3	45.96	140±5	92±5	33	3	87±3	46±4	9	0	159±3	65±4	8	0	0.50±0.04	0.70±0.03	0.98	1	0.95
181362*	**29**	LRC II	4	45.70	138±4	78±4	33	-	-	-	-	-	-	-	-	-	-	-	-	-	-
187308	**30**	LRC II	4	45.70	142±3	99±6	47	0	83±6	45±5	9	0	179±8	76±6	9	0	0.45±0.05	0.76±0.05	0.96	1	1
207307	**31**	LRC II	4	45.52	156±4	97±6	66	6	93.0±1.9	47±3	12	0	182±4	71±5	12	0	0.48±0.04	0.74±0.03	0.97	1	1
181361*	**32**	LRC II	4	45.50	156±3	95±5	33	-	-	-	-	-	-	-	-	-	-	-	-	-	-
187307	**33**	LRC II	4	45.18	243±10	328±23	25	17	257±13	201±19	7	0	798±76	473±59	7	22	0.61±0.06	1.44±0.16	1	0.84	1
187304	**34**	LRC II	5	45.13	182±13	121±11	33	8	98±8	52±6	10	0	201±15	82±9	10	0	0.43±0.06	0.68±0.07	1	1	1
161263*	**35**	LRC III	4	45.20	135±4	65±4	24	-	-	-	-	-	-	-	-	-	-	-	-	-	-
167831	**36**	LRC IV	1	49.80	70±3	27.8±1.7	21	4	36±3	13.3±1.5	6	0	88±6	26±2	6	0	0.48±0.06	0.92±0.07	0.94	1	1
167830	**37**	LRC IV	1	48.20	121±4	51±3	23	0	69±5	27±3	6	0	151±6	45±3	6	0	0.53±0.06	0.90±0.05	0.97	1	1
161264*	**38**	LRC IV	1	47.20	104±4	56±3	27	-	-	-	-	-	-	-	-	-	-	-	-	-	-
167829	**39**	LRC IV	1	46.50	115±7	65±5	24	0	59.9±1.1	28±2	5	0	140.8±1.6	52±4	5	0	0.44±0.04	0.80±0.05	1	0.96	1.01
167828	**40**	LRC IV	2	46.20	134±7	69±5	24	0	71±3	32±2	12	0	156±2	54±3	12	0	0.46±0.04	0.78±0.05	1	1	1
167827	**41**	LRC IV	2	46.00	137±7	71±5	23	0	66±3	30±2	12	0	154±4	54±4	12	0	0.42±0.04	0.76±0.05	0.95	1	1
167826	**42**	LRC IV	2	44.90	127±6	79±6	21	8	62±5	31±3	12	0	143±6	56±4	12	0	0.40±0.05	0.71±0.05	0.96	1	1
161266*	**43**	LRC IV	4	44.60	156±2	92±5	22	-	-	-	-	-	-	-	-	-	-	-	-	-	-
167823	**44**	LRC IV	4	44.60	148±7	74±5	21	13	98±6	43±4	12	0	187±7	64±5	12	0	0.58±0.06	0.86±0.06	1	1	1
167825	**45**	LRC IV	4	44.60	157±11	74±7	21	13	89±3	37±3	10	0	180±3	59±4	11	0	0.51±0.05	0.80±0.06	1	1	0.97
167822	**46**	LRC IV	4	44.50	172±7	79±5	24	7	106±6	44±4	12	0	222±7	71±5	11	0	0.55±0.06	0.90±0.05	0.97	1	0.97
167821	**47**	LRC IV	4	44.10	192±11	97±7	24	8	130±4	58±4	12	0	260±6	89±6	11	0	0.60±0.06	0.92±0.06	1	1	0.97
167820	**48**	LRC IV	5	43.30	291±19	184±16	18	31	452±41	238±28	12	0	1486±18	588±42	6	33	1.29±0.17	3.2±0.2	1	1	0.99
161265*	**49**	LRC IV	5	42.50	295±9	176±10	19	-	-	-	-	-	-	-	-	-	-	-	-	-	-
167819	**50**	LRC IV	5	42.50	281±19	153±14	18	28	387±35	185±18	12	0	1397±78	504±45	9	25	1.21±0.14	3.3±0.3	1	1	1

**$n = n_{tot} - n_{sat} - n_{IQR}$

Table SI.5: Summary of multi-grain OSL results. Samples prepared and measured in Hungary [(2)] are marked with an asterisk (*). “Fig. code” refers to the sample codes given in Fig. 6. “a,b,c,d” refers to different locations (“Locus’ in LRC I see Fig. 6). “Unit” is the deposition unit. “I.C.” and “E.C.” represent samples from inner cave of LRC I and the entrance of LRC I, respectively (not identified with a specific unit). “Unit 4 (E.C.)” represent samples from inside the entrance of LRC I identified to U4. “Elevation” is the elevation [m] above sea level of the sample locations (NGF). “Q” and “KF” are quartz and K-rich feldspar, respectively. “Dose” is the arithmetic average equivalent dose after application of the IQR rejection criterion. All uncertainties are reported at the 68% confidence interval. “Age” is the equivalent dose divided by the total dose rate (see Table SI.2). “n” is the number of aliquots included in the equivalent dose estimation, i.e., the total number of measured aliquots less the aliquots which gave unbounded dose estimates and the number of dose estimates rejected by the IQR criterion. Dose estimates rejected by the IQR criterion are shown in Figure SI.16. “In sat” is the relative number of aliquots giving unbounded dose estimates. The IR_{50} ages have been fading corrected using a g-value of $2.66 \pm 0.12\%$/decade. The $pIRIR_{290}$ ages have not been fading corrected. “IQR ratio” is the ratio between the average equivalent dose derived with and without application of the IQR rejection criterion.

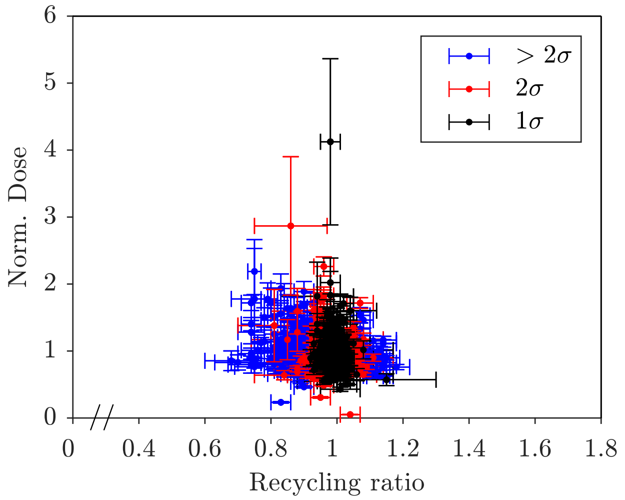

Figure SI.13: Individual doses normalised to the sample mean plotted against individual recycling ratios for every aliquot (n=840). Aliquots with recycling ratios consistent with unity at 68%, 95% and > 95% are shown in black, red, and blue, respectively.

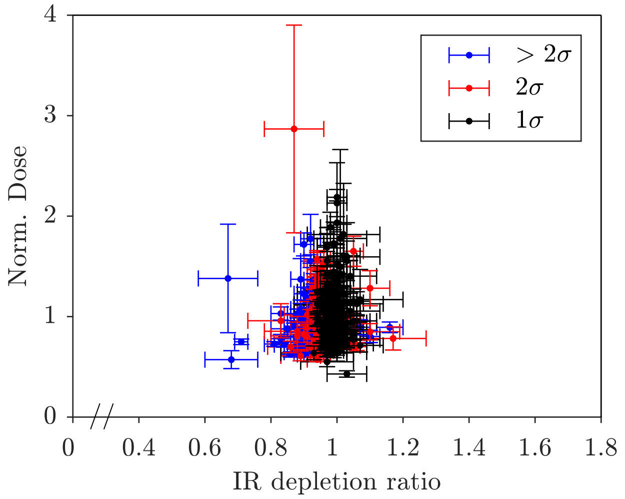

Figure SI.14: Individual doses normalised to the sample mean plotted against individual IR depletion ratios (n=453). Aliquots with recycling ratios consistent with unity at 68%, 95% and > 95% are shown in black, red, and blue, respectively.

Multi-grain quartz dose recovery

Dose recovery experiments aim to assess whether a known laboratory dose, given prior to any thermal treatment of the sample, can be recovered accurately using the given measurement protocol. A dose recovery ratio within 10% of unity is generally considered to be satisfactory [43].

A SAR protocol with a preheat of 260 °C (220 °C for the test dose) was used. In these experiments, aliquots were bleached twice for 100 s using blue light stimulation at room temperature, separated by a pause of 10 ks to allow charge transferred to the 110°C TL peak to decay to negligible levels. A known dose, ranging from 40 to 500 Gy, was given to batches of 6 to 48 aliquots from all samples from LRC IV and samples 187301-12, -20,-22,-23 and samples 167805,-06,-12 from LRC I and II. The given doses were chosen to be similar to the natural equivalent doses. The average dose recovery ratio of all experiments is 0.965 ± 0.012 (n=171) indicating that a laboratory dose given before the first thermal treatment can be measured using the chosen SAR protocol with sufficient accuracy. Note that this does not guarantee that natural doses can be measured with the same accuracy because we do not know whether the laboratory and natural dose response curves have the same shape [44,45].

In Figure SI.15 the average dose recovery ratios are shown as a function of given doses for sample 167819. Individual

aliquots were given doses ranging between 40 and 500 Gy. It is worth noting that the three dose recoveries done at high doses, i.e., at 300, 400 and 500 Gy, all give low dose recovery ratios of 0.85 ± 0.06 (n=6, 0% of aliquots in saturation), 0.81 ± 0.06 (n=4, 33% of aliquots in saturation) and 0.82 ± 0.06 (n=12, 20% of aliquots in saturation), respectively.

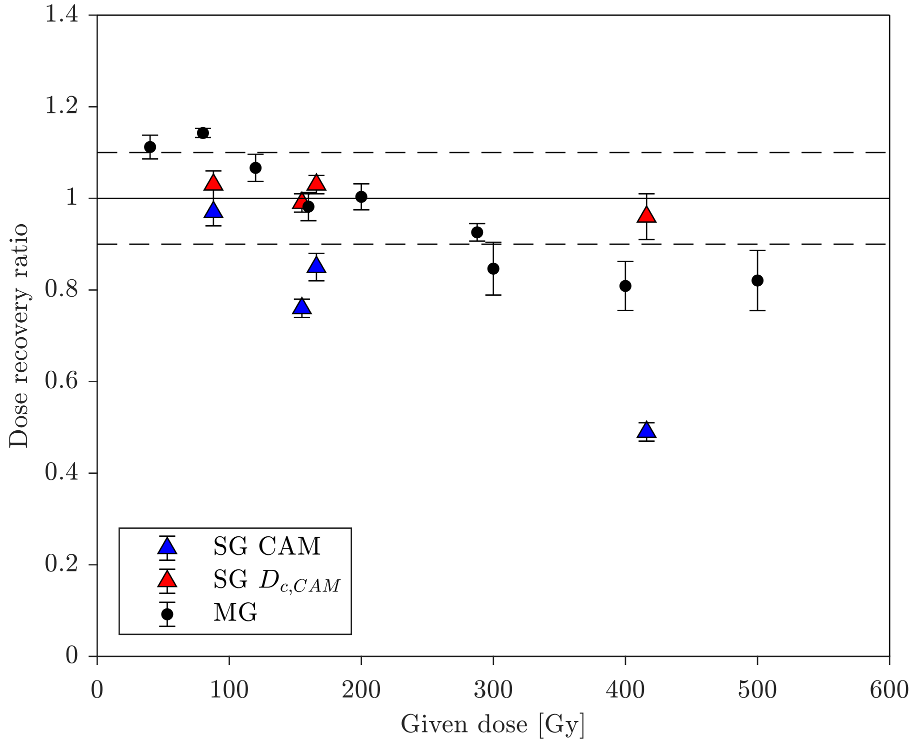

Figure SI.15: Average multi-grain (MG, black circles) and single-grain (SG, red and blue triangles) quartz dose recovery ratios for given doses ranging between 40 and 500 Gy. For MG (sample 167819), each point is an arithmetic average of 4-12 individual aliquots. For SG (samples 187302, 167806, 207307, 167319) each point is based on CAM and is shown both with (red triangles) and without (blue triangles) application of the D_c criterion (see section: *Single grain quartz dose recovery* for further details). The number of included SG dose estimates in each point range between 102 and 388 (SG CAM) and between 16 and 95 (SG $D_{c,CAM}$)

Multi-grain quartz natural dose distributions

Between 12 and 72 quartz multi-grain aliquots were measured for each sample and the estimated doses range between 70±3 Gy (167831) and 449±69 Gy (197340) (see Table SI.5). Figure SI.16 shows quartz multi-grain dose distributions processed in Denmark (ordered as in Table SI.5). The relative standard deviations of these range between 13% (sample 197328) and 59% (sample 167817) with an average value of ∼24%. There is a tendency for the relative standard deviations to increase with measured dose. We could not derive bounded dose estimates for a significant fraction of the aliquots (∼10% on average). Not surprisingly, the relative number of aliquots appearing to be in saturation increases with the measured dose.

When a significant number of aliquots appear to be in saturation, the accuracy of the resulting estimate of the burial dose must be questioned as it is likely that the estimate is significantly underestimated. There is no established method to determine when significant underestimation might be an issue due to saturation issues, but in line with Singh et al.[(46)] we regard samples with more than 15% of the individual multi-grain aliquots appearing to be in saturation as providing minimum burial dose estimates. This means that all samples from unit 5 in LRC IV, sample 187307 in LRC II and six samples from inside LRC I (I.C. and E.C.) are regarded as providing minimum burial ages. Note that these samples all give quartz multi-grain doses > 225 Gy (i.e., $> 2D_c$).

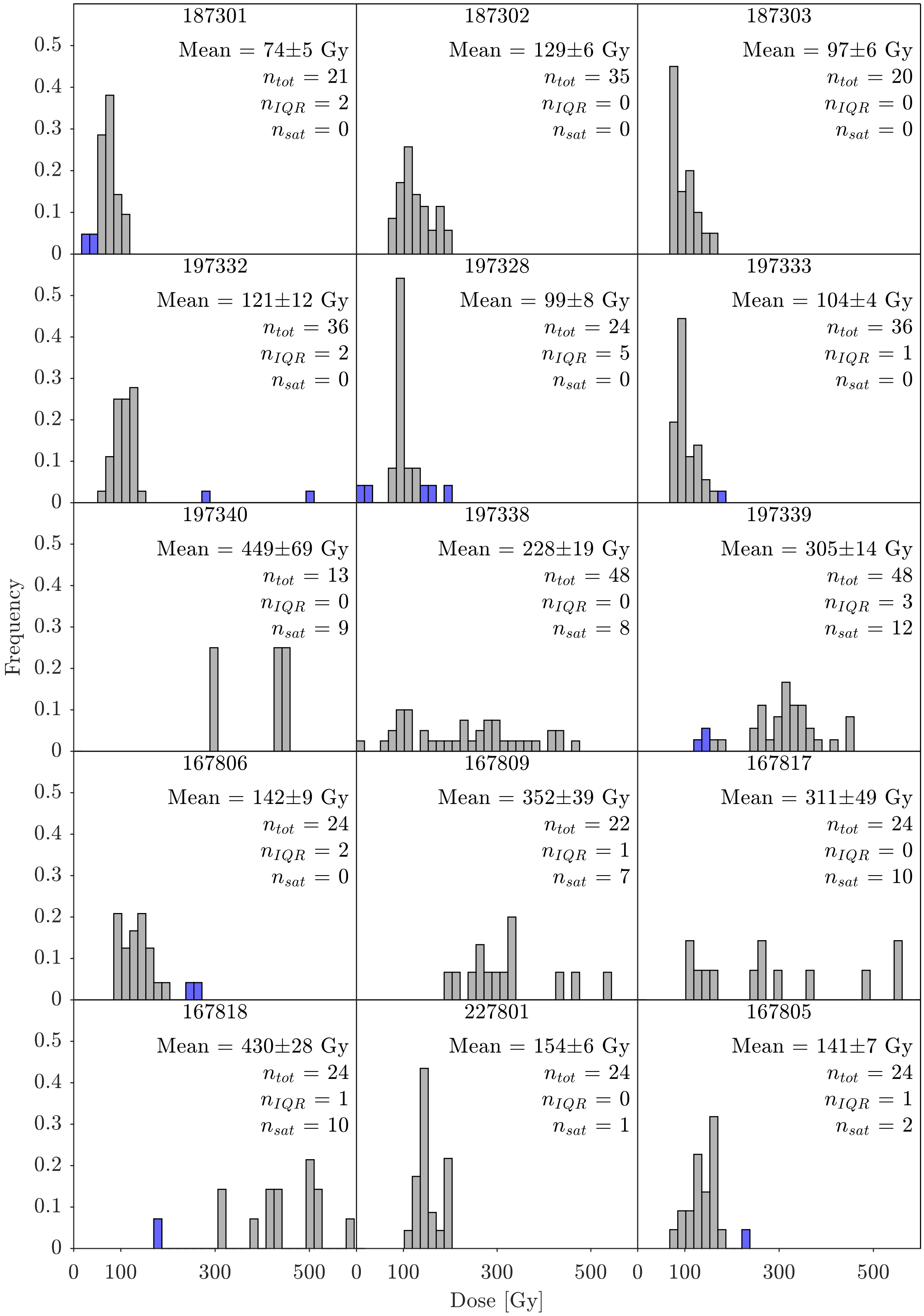

187301
Mean = 74±5 Gy
n_{tot} = 21
n_{IQR} = 2
n_{sat} = 0
187302
Mean = 129±6 Gy
n_{tot} = 35
n_{IQR} = 0
n_{sat} = 0
187303
Mean = 97±6 Gy
n_{tot} = 20
n_{IQR} = 0
n_{sat} = 0
197332
Mean = 121±12 Gy
n_{tot} = 36
n_{IQR} = 2
n_{sat} = 0
197328
Mean = 99±8 Gy
n_{tot} = 24
n_{IQR} = 5
n_{sat} = 0
197333
Mean = 104±4 Gy
n_{tot} = 36
n_{IQR} = 1
n_{sat} = 0
197340
Mean = 449±69 Gy
n_{tot} = 13
n_{IQR} = 0
n_{sat} = 9
197338
Mean = 228±19 Gy
n_{tot} = 48
n_{IQR} = 0
n_{sat} = 8
197339
Mean = 305±14 Gy
n_{tot} = 48
n_{IQR} = 3
n_{sat} = 12
167806
Mean = 142±9 Gy
n_{tot} = 24
n_{IQR} = 2
n_{sat} = 0
167809
Mean = 352±39 Gy
n_{tot} = 22
n_{IQR} = 1
n_{sat} = 7
167817
Mean = 311±49 Gy
n_{tot} = 24
n_{IQR} = 0
n_{sat} = 10
167818
Mean = 430±28 Gy
n_{tot} = 24
n_{IQR} = 1
n_{sat} = 10
227801
Mean = 154±6 Gy
n_{tot} = 24
n_{IQR} = 0
n_{sat} = 1
167805
Mean = 141±7 Gy
n_{tot} = 24
n_{IQR} = 1
n_{sat} = 2
Frequency
0 0.1 0.2 0.3 0.4 0.5
0 100 300 500
Dose [Gy]

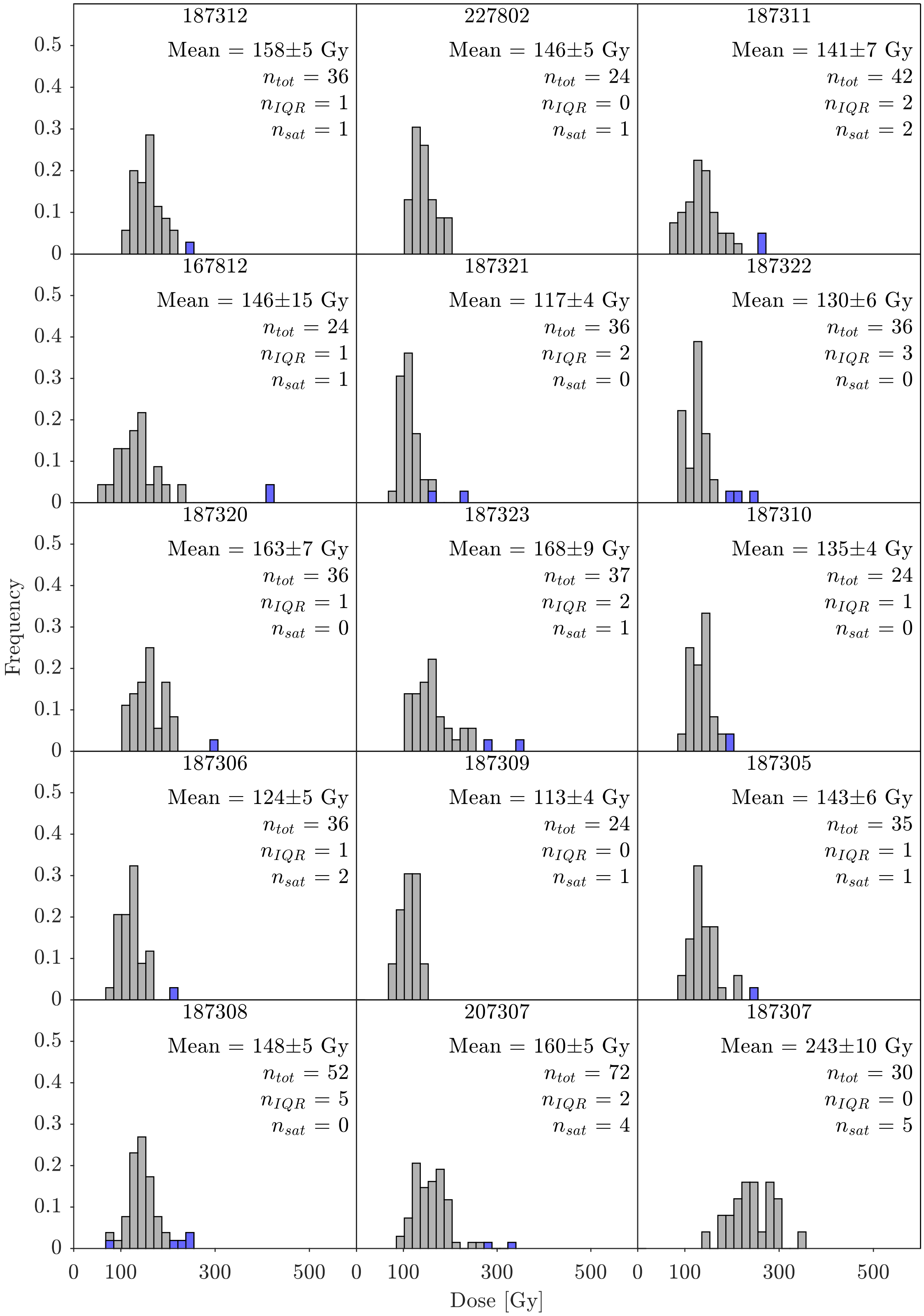
187312
Mean = 158±5 Gy
n_{tot} = 36
n_{IQR} = 1
n_{sat} = 1
227802
Mean = 146±5 Gy
n_{tot} = 24
n_{IQR} = 0
n_{sat} = 1
187311
Mean = 141±7 Gy
n_{tot} = 42
n_{IQR} = 2
n_{sat} = 2
167812
Mean = 146±15 Gy
n_{tot} = 24
n_{IQR} = 1
n_{sat} = 1
187321
Mean = 117±4 Gy
n_{tot} = 36
n_{IQR} = 2
n_{sat} = 0
187322
Mean = 130±6 Gy
n_{tot} = 36
n_{IQR} = 3
n_{sat} = 0
187320
Mean = 163±7 Gy
n_{tot} = 36
n_{IQR} = 1
n_{sat} = 0
187323
Mean = 168±9 Gy
n_{tot} = 37
n_{IQR} = 2
n_{sat} = 1
187310
Mean = 135±4 Gy
n_{tot} = 24
n_{IQR} = 1
n_{sat} = 0
187306
Mean = 124±5 Gy
n_{tot} = 36
n_{IQR} = 1
n_{sat} = 2
187309
Mean = 113±4 Gy
n_{tot} = 24
n_{IQR} = 0
n_{sat} = 1
187305
Mean = 143±6 Gy
n_{tot} = 35
n_{IQR} = 1
n_{sat} = 1
187308
Mean = 148±5 Gy
n_{tot} = 52
n_{IQR} = 5
n_{sat} = 0
207307
Mean = 160±5 Gy
n_{tot} = 72
n_{IQR} = 2
n_{sat} = 4
187307
Mean = 243±10 Gy
n_{tot} = 30
n_{IQR} = 0
n_{sat} = 5
Frequency
0
0.1
0.2
0.3
0.4
0.5
0
100
300
500
Dose [Gy]

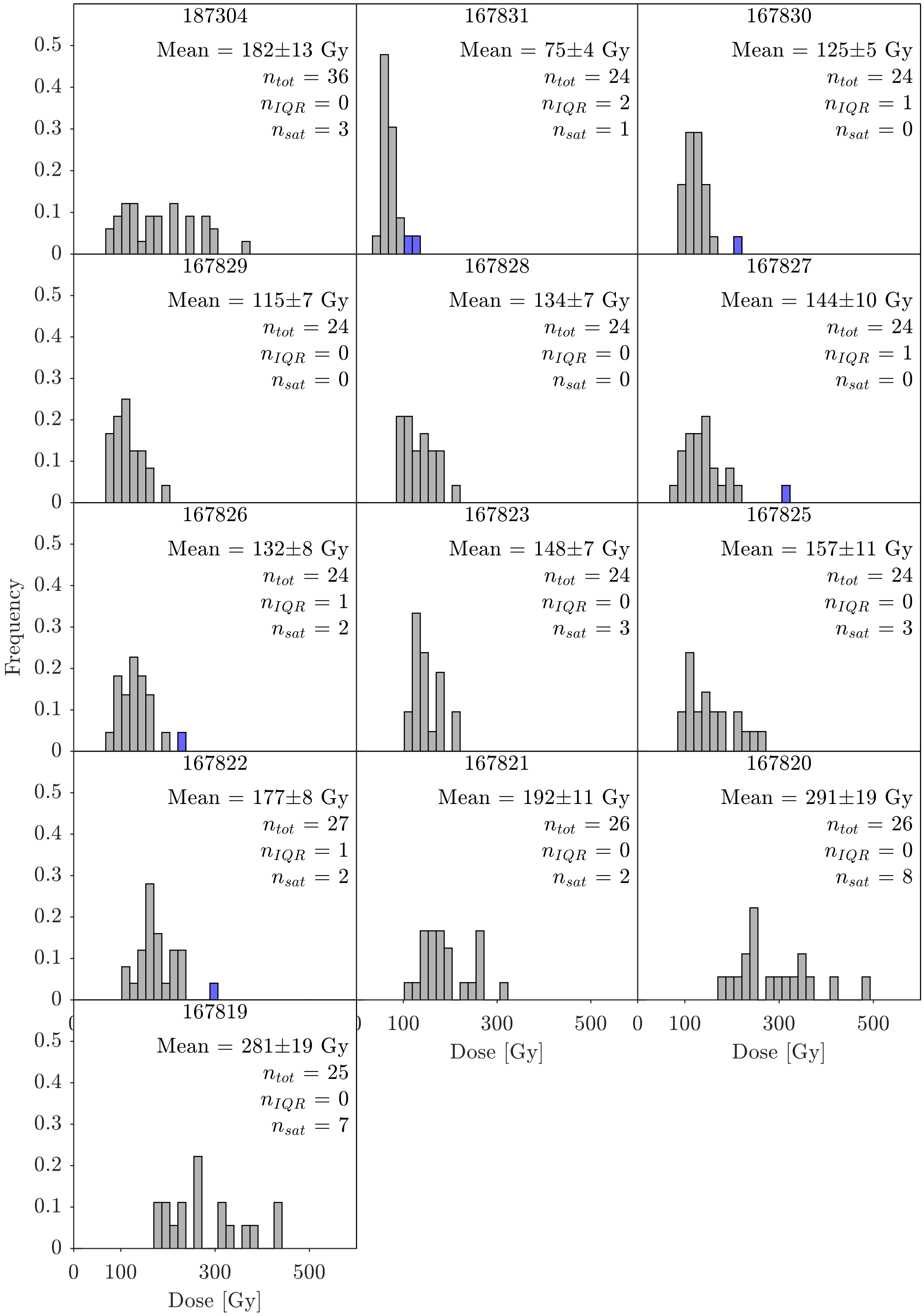

Figure SI.16: Multi-grain quartz dose distributions measured at Risø DTU. Grey bars: Doses after application of the IQR criterion. Blue bars: dose estimates rejected by the IQR criterion. The legend for each sample gives the arithmetic mean dose and its standard error of all measured aliquots giving a bounded dose estimate, the total number of measured aliquots (n_{tot}), the number of aliquots rejected by the IQR criterion (n_{IQR}), and the number of aliquots for which no bounded dose estimate could be derived (n_{sat}), i.e., the number of aliquots appearing to be in saturation on the laboratory dose response curve.

Single grain quartz results

Quartz single-grain analysis was undertaken for 12 samples to investigate whether the samples are likely to have been significantly incompletely bleached, mixed after deposition, and/or suffer from saturation effects. In the literature, it is also argued that single-grain OSL data are superior to multi-grain data, because it is possible to reject individual grains with poor luminescence characteristics (e.g., 47). We assume that a good agreement between multi-grain and single-grain quartz dose estimates means that none of these concerns affect our multi-grain results significantly.

Single grain quartz dose recovery

Single grain dose recovery experiments, similar to those carried out for multi-grain aliquots (i.e., grains were bleached twice at room temperature with the blue LEDs for 100 s with an intervening pause of 10,000 s before giving a known laboratory dose), were undertaken for four samples (187302, 167806, 207307 and 167819). A total of 8,100 individual grains were measured and 17% of these were detectable, i.e., gave natural test dose responses with $s_{Tn} < 20\%$ (see Table SI.6). The given doses were 88, 166, 155 and 416 Gy for samples 187302, 167806, 207307 167819, respectively. The corresponding individual CAM dose recovery ratios (Figure SI.15) were 0.97±0.03 (n=102), 0.85 ± 0.03 (n=142), 0.76 ± 0.02 (n=388) and 0.49 ± 0.02 (n=259) with over-dispersion (OD) values of 17 ± 3, 29 ± 3, $36 \pm 2\%$ and $46 \pm 3\%$, respectively (see Tables SI.6 and SI.7)[1]. Only the dose recovery at a given dose of 88 Gy is acceptable (i.e., consistent with unity at two standard deviations). It is worth noting that for all experiments a significant number of grains (varying between 17 and 57%) with detectable T_n signals did not give bounded dose estimates. There is a strong linear correlation ($R^2 = 0.996$) between the given dose and the number of grains giving no bounded dose estimates due to saturation effects. However, applying the Bayesian approach the dose recovery ratios are 1.08 ± 0.04, 1.00 ± 0.02, 0.89 ± 0.02 and 0.90 ± 0.02, respectively, i.e., two of the four dose recovery ratios are acceptable. The average dose recovery ratios for all samples are 0.77 ± 0.10 and 0.97 ± 0.05 for CAM and BayLum, respectively. Thus we can, on average, recover the given dose when using BayLum analysis, whereas using CAM underestimates the given dose. This suggests that Bayesian modelling may be less biased by grains giving unbounded dose estimates (see below).

Figure SI.17a shows the measured dose distribution for sample 207307. For this sample, 23% of the detectable grains gave unbounded dose estimates. As has previously been reported in the literature (e.g., 48) the average D_c value for grains with a natural OSL signal in saturation (i.e., no bounded dose estimate can be derived) is significantly lower than for those grains giving bounded dose estimates. The weighted average ratio of D_c values for grains in saturation to those giving bounded dose estimates is 2.0 ± 0.3 (n=4 samples) for the single-grain dose recovery data sets. Thomsen et al. (49) showed that, for their samples, individual grains should have D_c values $\gtrsim$ to the dose to be measured, before they are likely to act, on average, as accurate dosimeters. This condition is shown as the intersection of the solid line of unit slope with the measured data in Figure SI.17b, in this case measured to given dose ratio. Applying the approach of Thomsen et al. (49), i.e., the "D_c criterion" in which grains, irrespective of equivalent dose, D_e, are filtered based on individual D_c values, changes the CAM dose recovery ratios to 1.03 ± 0.03 (n=81), 0.99±0.02 (n=95), 1.03±0.02 (n=71) and 0.96±0.05 (n=16) and reduces the proportion of grains appearing in saturation to 2, 1, 5 and 0%, respectively. The resulting CAM OD values are 12 ± 4, 10 ± 2, $8 \pm 3\%$ and $7 \pm 9\%$, respectively.

[1]Note that the ADM should only be applied to dose distributions where extrinsic factors contribute to the observed variability

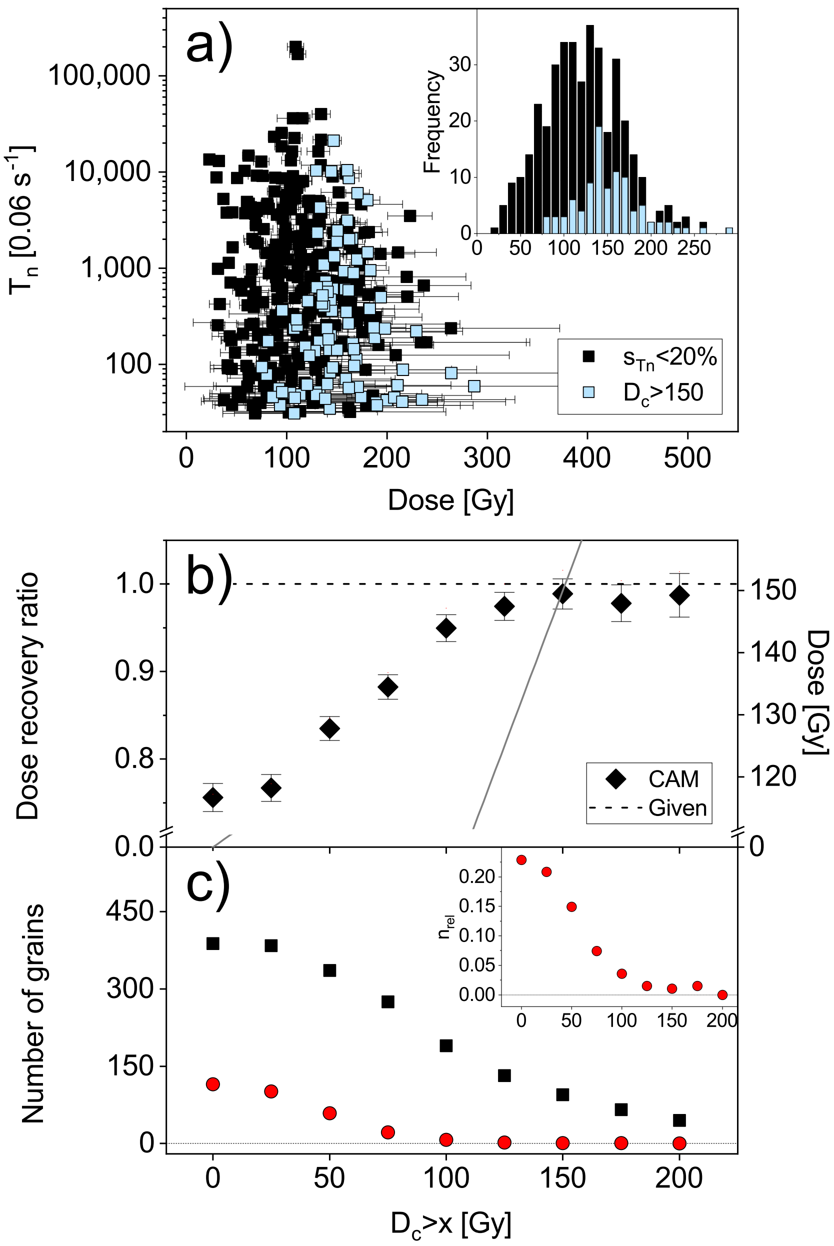

Figure SI.17: Single grain dose recovery data from sample 207307. a) Scatter plot showing intrinsic brightness (T_n, "natural" test dose response) against estimated dose. The inset shows a simple frequency diagram of the dose values. The black squares show all dose estimates (with $s_{Tn} < 20\%$), whereas the light blue data only show dose estimates which also have a D_c value greater than 150 Gy. b) Effect of rejecting grains according to their D_c values on the dose recovery ratio (left axis) and average dose (right axis) using CAM (black diamonds). The horizontal dashed line indicates a dose recovery ratio of unity. The 1:1 line (right axis) intersects the unit dose recovery ratio at a D_c of ~150 Gy indicating that, for this sample, the dose recovery is satisfactory if only grains with a D_c equal to greater than the given dose of 150 Gy are accepted. c) Effect of rejecting grains according to their D_c values on the number of grains with bounded estimates (black squares) and unbounded dose estimates (red circles). The inset shows the proportion of grains with unbounded dose estimates as a function of $D_c > x$.

Figure SI.17a also shows the remaining dose distribution from the dose recovery experiment on sample 207307 after all grains with a D_c value equal to or less than 150 Gy have been rejected (blue symbols). Figure SI.17b shows the effect on the dose recovery ratio and average dose as the D_c threshold is increased. The dose recovery ratio is consistent with unity when only grains with a D_c value larger than 125 Gy are included in the distribution. Following Thomsen et al.[(49)] and Singh et al.[(46)], the D_c threshold is determined as the value when the average

dose of the remaining distribution is approximately equal to the minimum D_c value. These results strongly suggest that D_c filtering is both appropriate and necessary.

The average dose recovery ratios for the four dose recovery samples after application of the D_c criterion are 1.00 ± 0.02 and 1.01 ± 0.03 for CAM and BayLum, respectively. It is interesting to note that the Bayesian approach to data analysis appears to be insensitive to including grains, which in conventional analysis (CAM) are incapable of recording the dose of interest accurately [50].

Figure SI.17c shows how the number of grains (with both bounded and unbounded dose estimates) decreases with the D_c threshold. Thus, applying the D_c criterion is expensive in terms of grain rejection, but does remove the apparent underestimation observed at given doses greater ~100 Gy caused by grains not able to record the dose of interest. The dose recovery ratios for the four samples are all acceptable using either of the two analytical approaches, when the D_c criterion is applied.

Single grain quartz dose estimation

The natural OSL signals from 18,900 individual quartz grains were measured. Of these grains, 18% gave test dose responses with $s_{Tn} < 20\%$, and ~13% had natural sensitivity-corrected signals which gave bounded dose estimates by interpolation on to the laboratory regenerated dose response curves.

Effect of single-grain rejection criteria

Figures SI.18a and SI.18b show the measured single-grain dose distribution for samples 207307 and 197339, respectively. The black bars/points represent all grains giving bounded dose estimates with $s_{\mathrm{Tn}} < 20\%$, whereas the white bars/points represent the bounded dose estimates, which also fulfil the rejection criteria given in section: *OSL measurements*, i.e., recycling and IR depletion ratio within two standard deviations of unity and recuperation within 2 standard deviations of zero. Application of these rejection criteria for these two samples does not significantly change the average dose or the observed over-dispersion but does reduce the grain population by 55% and 41%, respectively. For all samples, the ratio of CAM doses, ODs and reduction in grain population with and without application of the rejection criteria is 0.97 ± 0.02, 0.95 ± 0.04 and 0.49 ± 0.03, respectively. Since the only significant effect of applying these rejection criteria is to reduce the accepted grain population, these rejection criteria are not considered further. Similar conclusions were reached by e.g., Thomsen et al. [48].

Table SI.6: Summary of single-grain quartz results. "Nat" is the natural signal and "DR" is dose recovery. The given doses were 88, 166, 155 and 416 Gy, respectively. "N_{tot}" is the total number of measured single grains. "s_{Tn}" is the uncertainty assigned to OSL response for the test dose for the natural SAR cycle. "n" is the number of accepted grains. "OD CAM" and "D_e CAM" is the over-dispersion and average equivalent dose, respectively, calculated using the Central Age Model (CAM, Galbraith, 1999). "D_e ADM" is the equivalent dose calculated using the average dose model [30] with an internal over-dispersion of 17% . Note that The ADM should only be applied to dose distributions where extrinsic factors contribute to the observed variability. "D_e BayLum" is the equivalent dose estimate calculated using the R-package BayLum [30]. Here, for simplicity, the dose is given as the midpoint of the 68% confidence interval. No doses are reported for the BayLum analysis after application of the D_c criterion to samples 197338 and 167819, since the analysis did not converge; presumably caused by the low number of accepted grains.

Sample	Signal	Site	Unit	N_{tot}	s_{Tn}<20%						s_{Tn}<20% and D_c>$D_{e,av}$					
					n	n_{sat} (%)	OD CAM (%)	D_e CAM (Gy)	D_e ADM (Gy)	D_e BayLum (Gy)	n	n_{sat} (%)	OD CAM (%)	D_e CAM (Gy)	D_e ADM (Gy)	D_e BayLum (Gy)
197338	Nat	LRC I	E.C.	1,600	83	57%	71 ± 7	140 ± 13	178 ± 13	825 ± 154	20	41%	28 ± 9	278 ± 28	285 ± 24	-
197339	Nat	LRC I	E.C.	1,800	119	59%	52 ± 5	164 ± 10	186 ± 10	399 ± 24	15	21%	43 ± 11	264 ± 35	293 ± 46	410 ± 59
167812	Nat	LRC I	4	1,000	153	12%	42 ± 3	101 ± 4	109 ± 5	120 ± 5	101	5%	45 ± 4	110 ± 6	144 ± 10	125 ± 6
187311	Nat	LRC I	2	1,700	167	25%	36 ± 3	104 ± 3	110 ± 3	135 ± 5	65	10%	36 ± 4	126 ± 7	133 ± 7	157 ± 11
187312	Nat	LRC I	2	1,100	105	33%	33 ± 3	117 ± 5	121 ± 5	166 ± 10	50	12%	46 ± 5	127 ± 7	132 ± 8	164 ± 14
167805	Nat	LRC I	4	1,700	262	26%	37 ± 2	115 ± 3	122 ± 3	139 ± 3	108	7%	32 ± 3	134 ± 5	139 ± 6	143 ± 6
167806	Nat	LRC I	2	1,700	287	21%	29 ± 2	104 ± 2	107 ± 2	122 ± 3	147	6%	25 ± 2	115 ± 3	117 ± 4	127 ± 4
187302	Nat	LRC I	1	1,100	146	17%	36 ± 3	94 ± 3	99 ± 3	116 ± 4	92	3%	34 ± 3	97 ± 4	101 ± 5	110 ± 5
207307	Nat	LRC II	4	1,700	275	22%	32 ± 2	104 ± 2	108 ± 2	123 ± 3	114	5%	27 ± 3	119 ± 4	121 ± 4	132 ± 5
167819	Nat	LRC IV	5	1,800	126	64%	60 ± 5	198 ± 12	233 ± 16	3174 ± 827	12	0%	38 ± 11	384 ± 51	404 ± 66	-
167823	Nat	LRC IV	4	1,400	276	21%	32 ± 2	119 ± 3	123 ± 3	143 ± 3	122	3%	28 ± 2	125 ± 4	128 ± 4	137 ± 4
167831	Nat	LRC IV	1	2,300	409	5%	30 ± 1	72 ± 1	75 ± 1	79 ± 1	314	0%	28 ± 2	76 ± 1	78 ± 2	80 ± 2
187302	DR	LRC I	1	1,900	102	17%	17 ± 3	86 ± 2	-	96 ± 3	81	2%	12 ± 4	91 ± 3	-	93 ± 3
167806	DR	LRC I	3	1,100	142	25%	29 ± 3	141 ± 4	-	166 ± 4	71	5%	8 ± 3	171 ± 4	-	176 ± 5
207307	DR	LRC II	4	1,900	388	23%	36 ± 2	117 ± 2	-	138 ± 2	95	1%	10 ± 2	153 ± 3	-	151 ± 5
167819	DR	LRC IV	5	3,200	259	57%	46 ± 3	205 ± 7	-	374 ± 10	16	0%	7 ± 9	400 ± 22	-	396 ± 22

Application of the D_c criterion to single-grain dose distributions

The OD values from samples from unit 1-4 range between 29 ± 2 and $42 \pm 3\%$ with an average of $34.2 \pm 1.4\%$ (n=9, see Table SI.6). OD values of this magnitude is commonly reported for dose distributions obtained from samples expected to be well-bleached and not disturbed by post-depositional mixing [(51)]. The OD values of the three samples from unit 5 and E.C. are significantly higher, ranging between 52 ± 5 and $71 \pm 7\%$, which could indicate that these samples are suffering from significant incomplete bleaching, gross post-depositional mixing and/or saturation effects. Using the three methods of analysis (see section: *OSL measurements*) the average ratios of the single-grain dose estimates to that derived from multi-grain analysis for the samples from unit 1-4 are 0.79 ± 0.03 (CAM), 0.83 ± 0.04 (ADM) and 0.97 ± 0.03 (BayLum). Interestingly, the single-grain doses estimated by BayLum for samples 167819 and 197338 (unit 5 and E.C.), are > 800 Gy ($> 300\%$ higher than those estimated by multi-grain analysis) which may imply that these samples cannot be dated accurately using quartz OSL.

The dose recovery experiments indicate that single-grain measurements on these samples may underestimate the burial dose for doses larger than ~100 Gy unless saturation effects are appropriately dealt with. The number of single grains appearing to be in saturation (i.e., no bounded equivalent dose estimate could be derived) varies between 5% and 64% and correlates with sample dose. Of particular concern, are the samples from unit 5 and E.C. (167819, 197338 and 197339), which have 64%, 57% and 59%, respectively, of the light-giving grains giving unbounded dose estimates. This is cause for serious concern as it may lead to significant age underestimation for single-grain data sets [(e.g., 46,49)].

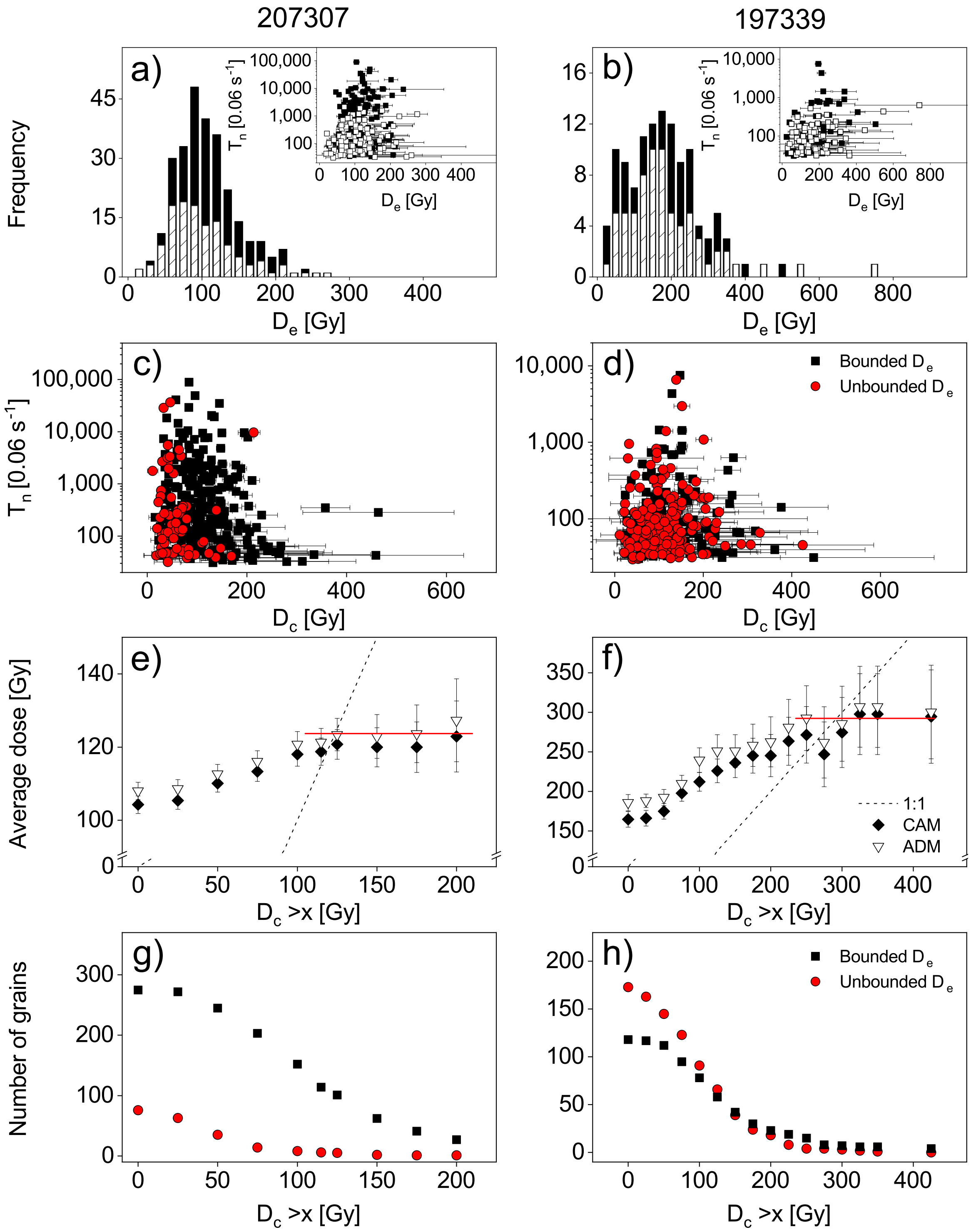

Figure SI.18: Natural single-grain quartz results for samples 207307 (a,c,e and g) and 197339 (b,d,f and h). a),b) Natural dose distributions for $s_{Tn} < 20\%$ (black data) and $s_{Tn} < 20\%$, recycling and IR depletion ratio consistent with unity at two standard deviations and recuperation consistent with zero at two standard deviations. c),d) D_c values plotted against natural OSL test dose response (T_n). Grains giving bounded dose estimates are shown as black squares, whereas grains giving unbounded dose estimates are shown as red circles. e),f) Effect on selecting grains based on their individual D_c values for CAM (closed symbols) and ADM (open symbols). Also shown is the 1:1 line (dashed). The average dose in the sample is determined as the point where the 1:1 line intersects the average dose curve. The red horizontal line indicates a plateau region in which the average dose is independent of tightening the D_c threshold further. g),h) The number of grains included for different D_c thresholds.

Figure SI.18c shows the D_c distribution for individual grains for sample 207337 (unit 4) and a clear overlap between bounded and unbounded estimates is observed. The grains with bounded (78%) and unbounded dose estimates (22%) have weighted average D_c values of 104 ± 3 Gy and 58 ± 3 Gy, respectively, i.e., the ratio between these D_c values is 1.80 ± 0.10. Figure SI.18d shows the same data for sample 197339 (E.C.), but for this sample the D_c distribution of the bounded dose estimates is very similar to the one for the unbounded dose estimates, i.e., weighted average D_c values of 114 ± 5 Gy and 102 ± 4 Gy, respectively, i.e., with a ratio of 1.12 ± 0.06. Figure SI.18e and SI.18f show the effect of increasing the D_c threshold for samples 207307 and 197339, respectively. As for the dose recovery data, the intersection of the dashed line with the equivalent dose data gives an estimate of the minimum acceptable D_c value. For sample 207307 the equivalent dose estimate increases by ~15% (at $D_c > 125$ Gy) but for sample 197339 by ~60% (at $D_c > 250$ Gy). Application of the D_c criterion decreases the number of grains included in the calculation by 59% (from n=275 to 114) and 87% (from n=119 to 15), for samples 207307 and 197339, respectively (see Table SI.6).

For the two other single-grain samples from E.C. and unit 5 (samples 197338 and 167819), the accepted grain population is reduced by ~90% (from n=126 to 12) and 75% (from n=83 to 20), respectively. For unit 5 and E.C., the number of accepted dose estimates is small (i.e., 15, 12 and 20) and thus caution must be exercised when interpreting the resulting burial doses. However, it is worth noting that the dose recovery experiment for sample 167819 (given dose of 416 Gy) was satisfactory after application of the D_c criterion even all though only 16 grains remained. Before application of the D_c criterion, the average OD value for these three samples is 61±6 %. However, after application of the D_c criterion, the average OD value decreases to 36±5 %, which is entirely consistent with the average OD values observed for the samples from units 1-4 (average 34.1±1.4 % or 34±3 % when the D_c criterion has been applied, see Table SI.6).

On average, the effect of using the D_c criterion is to increase the equivalent dose (estimated using either CAM or ADM) by ~11% (unit 1-4) and by ~80% (unit 5 and E.C.). Using the Bayesian approach, there is no significant effect of applying the D_c criterion for samples from unit 1-4, i.e., the average dose ratio is 1.03 ± 0.02. For units 5 and E.C., the Bayesian analysis only converged for one out of three samples. For the sample that did converged (sample 197339) the ratio is 1.03 ± 0.16.

Comparison of single-grain and multi-grain quartz dose estimates

As mentioned above, single-grain analysis was undertaken to examine whether the quartz samples suffer from significant incomplete bleaching, post-depositional mixing and/or saturation effects. If the samples are significantly affected by any of these processes or contain a significant number of grains with unsuitable luminescence characteristics, it is possible that the multi-grain results are inaccurate. If, on the other hand, good agreement between multi-grain and single-grain quartz dose estimates is found, the multi-grain results are unlikely to be affected by the processes given above, and as a result, are more likely to be accurate.

In Table SI.7, we compare the individual single-grain dose estimates to those obtained from multi-grain measurements. For the samples from unit 1-4 (n=9), the ratio of the single-grain doses (selection criterion $s_{Tn} < 20\%$) to the corresponding multi-grain doses are 0.79 ± 0.03 (CAM), 0.83 ± 0.03 (ADM) and 0.97 ± 0.03 (BayLum). However, if the D_c criterion is applied to reduce the effect of grains not capable of recording the absorbed dose accurately, these ratios are 0.87 ± 0.04 ($D_{c,CAM}$), 0.93 ± 0.04 ($D_{c,ADM}$) and 0.99 ± 0.04 ($D_{c,BayLum}$). Thus, on average, both ADM and BayLum give doses consistent with those obtained using multi-grain aliquots, whereas the CAM doses appears to underestimate by ~10%. For the three samples from units 5 and E.C., the multi-grain measurements are regarded as providing minimum burial ages due to likely saturation, but it is interesting to note that after application of the D_c criterion there is no significant difference between multi-grain and single-grain dose estimates, i.e., the single-grain to multi-grain ratios are 1.1± 0.2 ($D_{c,CAM}$) and 1.2±0.2 ($D_{c,ADM}$). Note that for samples 197338 and 167819, no BayLum D_c dose estimate is given as the analysis did not converge. For sample 197339 the ratio is 1.3±0.2.

CAM essentially calculates a weighted geometric mean of individual equivalent doses and it has been argued that this increasingly causes CAM to underestimate the true dose as the OD increases [30,31]. Using quartz OSL samples with independent chronological age control, these studies showed that the accuracy of single-grain CAM doses appeared to decrease with increasing dose/age. Using the same samples, Guérin et al. [30] showed that using the ADM on the other hand gave good agreement with the independent age control, but that the ADM also showed a small but increasing age underestimation with increasing age. By contrast, BayLum calculated doses that, on

average, were in agreement with the expected value in laboratory-controlled experiments and did not appear to show a trend with increasing dose/age [50].

In this study, we have no independent age control available against which to test our OSL ages, but we do observe the same trends as Guérin et al. [30,31] and Heydari and Guérin [50], i.e., the CAM doses tend to underestimate, whereas ADM and BayLum doses agree with multi-grain doses. We conclude that the single-grain quartz measurements, on average, agree with those obtained using the multi-grain approach, when the D_c criterion is applied. Rejecting individual grains with "poor" luminescence characteristics does not change the dose or observed variability in the single-grain dose distributions and it thus cannot be stated that single-grain quartz data is superior to multi-grain quartz data. Quartz ages (see section: *Luminescence ages*) are thus based on the multi-grain quartz dose measurements. The ultimate test for whether incomplete bleaching is an issue is to compare quartz ages with K-rich feldspar ages (see sections *Multi-grain K-rich feldspar results* and *Luminescence ages*).

Table SI.7: Ratios of quartz single-grain (SG) and multi-grain (MG) equivalent doses. "Nat" is the natural signal (burial dose) and "DR" is the dose recovery signal (dose given in the laboratory). Multi-grain equivalent doses are calculated as an arithmetic mean (see Table SI.5), whereas the single-grain equivalent doses are calculated using various approaches (see Table SI.6).

Sample	Signal	Site	Unit	SG (s_{Tn}<20%) to MG or given			SG (s_{Tn}<20% and D_c>$D_{e,av}$) to MG or given		
				CAM	ADM	BayLum	CAM	ADM	BayLum
197338	Nat	LRC I	E.C.	0.62 ± 0.08	0.78 ± 0.09	3.6 ± 0.7	1.22 ± 0.16	1.25 ± 0.15	-
197339	Nat	LRC I	E.C.	0.51 ± 0.04	0.58 ± 0.04	1.25 ± 0.08	0.82 ± 0.11	0.91 ± 0.15	1.28 ± 0.19
167812	Nat	LRC I	4	0.76 ± 0.06	0.82 ± 0.06	0.90 ± 0.07	0.82 ± 0.07	1.07 ± 0.10	0.94 ± 0.08
187311	Nat	LRC I	2	0.77 ± 0.04	0.81 ± 0.04	1.00 ± 0.05	0.94 ± 0.06	0.98 ± 0.07	1.17 ± 0.09
187312	Nat	LRC I	2	0.75 ± 0.04	0.78 ± 0.04	1.07 ± 0.07	0.81 ± 0.05	0.85 ± 0.06	1.05 ± 0.10
167805	Nat	LRC I	4	0.84 ± 0.04	0.89 ± 0.04	1.01 ± 0.05	0.98 ± 0.05	1.01 ± 0.06	1.05 ± 0.06
167806	Nat	LRC I	2	0.79 ± 0.04	0.81 ± 0.04	0.92 ± 0.05	0.87 ± 0.05	0.89 ± 0.05	0.96 ± 0.05
187302	Nat	LRC I	1	0.73 ± 0.04	0.77 ± 0.04	0.90 ± 0.05	0.75 ± 0.05	0.79 ± 0.05	0.85 ± 0.05
207307	Nat	LRC II	4	0.67 ± 0.02	0.69 ± 0.02	0.79 ± 0.03	0.76 ± 0.03	0.78 ± 0.03	0.85 ± 0.04
167819	Nat	LRC IV	5	0.70 ± 0.07	0.83 ± 0.08	11 ± 3	1.4 ± 0.2	1.4 ± 0.3	-
167823	Nat	LRC IV	4	0.80 ± 0.04	0.83 ± 0.04	0.97 ± 0.05	0.84 ± 0.05	0.86 ± 0.05	0.93 ± 0.05
167831	Nat	LRC IV	1	1.03 ± 0.04	1.07 ± 0.04	1.13 ± 0.05	1.08 ± 0.04	1.11 ± 0.05	1.14 ± 0.05
187302	DR	LRC I	1	0.97 ± 0.03	-	1.08 ± 0.04	1.03 ± 0.03	-	1.06 ± 0.03
167806	DR	LRC I	3	0.85 ± 0.03	-	1.00 ± 0.02	1.03 ± 0.02	-	1.06 ± 0.03
207307	DR	LRC II	4	0.76 ± 0.02	-	0.89 ± 0.02	0.99 ± 0.02	-	0.97 ± 0.03
167819	DR	LRC IV	5	0.49 ± 0.02	-	0.90 ± 0.02	0.96 ± 0.05	-	0.95 ± 0.05

Multi-grain K-rich feldspar results

One of the main assumptions in OSL dating is that any latent OSL signal was reset at burial. If this was not the case then the OSL age is likely to overestimate the true burial age. The bleaching rate of the quartz OSL signal is at least an order of magnitude faster than that from K-rich feldspar [e.g., 52] and so a robust way to determine whether the quartz OSL signal was sufficiently reset at burial is to compare quartz and feldspar ages to each other [e.g., 53]. To facilitate such a comparison, multi-grain K-rich feldspar measurements using a pIRIR(50,290) protocol [54] (here referred to as pIRIR290) were done on all samples measured at DTU, Denmark. All reported IR_{50} doses/ages have been measured using the pIRIR290 protocol unless otherwise specifically stated.

Multi-grain K-rich feldspar characteristics

Multi-grain K-rich feldspar measurements generally show that the sedimentary K-feldspar is suitable for OSL analysis, i.e., the dose response curves are reproducible (average recycling ratio of 1.040 ± 0.004, n=435 (IR_{50}) and 1.014 ± 0.002, n=450 ($pIRIR_{290}$) with a small recuperation (on average 3.5% of the natural dose). Figure SI.19 shows a typical K-rich feldspar dose response curve. For this aliquot (from sample 167830 KF), the recycling ratios are 1.01±0.03 for both (IR_{50}) and ($pIRIR_{290}$), the recuperations 2.33±0.11% (IR_{50}) and 4.50±0.14% ($pIRIR_{290}$), and the D_c values 351 ± 31 Gy (IR_{50}) and 254 ± 19 Gy ($pIRIR_{290}$). The inset shows the natural OSL stimulation curve from the same aliquot. The $pIRIR_{290}$ signal was on average 13.6 ± 0.4 (n=435) times more intense than the IR_{50} signal. Average D_c values were 395 ± 4 Gy (n=435) and 390 ± 5 Gy (n=450) for IR_{50} and $pIRIR_{290}$, respectively. Based on the average dose response curve shapes, we would thus not expect to be able to accurately measure natural doses significantly greater than $\sim$ 800 Gy.

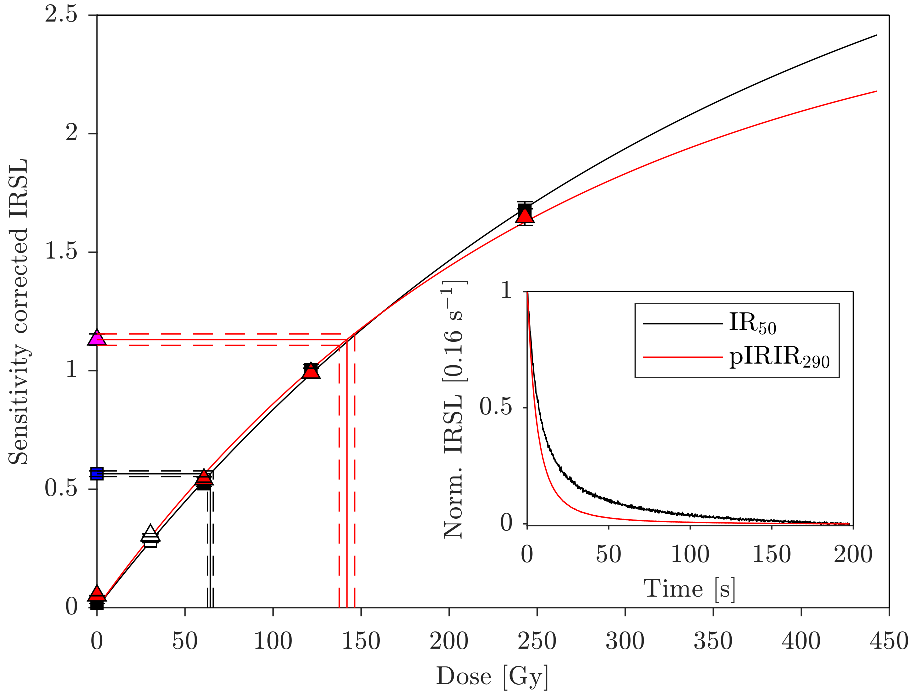

Figure SI.19: A typical dose response curve for a K-rich feldspar aliquot from sample 167830 for the IR_{50} signal (black squares) and the $pIRIR_{290}$ signal (red triangles). Recycling points are shown as corresponding open symbols. Natural signals are shown as blue and magenta symbols for the IR_{50} and $pIRIR_{290}$ signals, respectively. The data have been fitted using single saturating exponential functions. The insert shows the normalised natural OSL signals from this aliquot.

Multi-grain K-rich feldspar preheat plateau and dose recovery

To investigate the dependence of the equivalent dose on our choice of thermal pre-treatment and pIRIR stimulation temperature in the SAR protocol, we measured a preheat plateau for sample 187323. In this experiment the first IR

stimulation temperature was kept constant at 50°C, while the stimulation temperature for the second IR stimulation, i.e., the pIRIR$_T$ stimulation temperature, was increased in steps from 150°C to 290°C. The corresponding preheat temperature was increased from 170°C to 320°C. In Figure SI.20a, the measured average dose is shown as a function of the second stimulation temperature, T. Each point is the arithmetic mean of three aliquots corrected for average thermal transfer. It appears that our equivalent doses are insensitive to the temperature T of the 2nd IR stimulation and the preheat temperature.

To investigate our ability to recover a known dose given in the laboratory a pIRIR(50,T) dose recovery experiment using three different 2nd stimulation temperatures (i.e., T=180, 225 or 290 °C) was undertaken. The preheat temperatures were 200 °C, 250 °C and 320 °C, respectively. In these experiments, aliquots from samples 187323, 167825, 167827, 167830 were bleached for 48 hours in a solar simulator (Hönle SOL2) at room temperature. The residual dose in half of these aliquots were measured and these ranged between 2 and 24 Gy for the pIRIR$_T$ signals and between 0.8 and 2.9 Gy for the IR$_{50}$ signal. A known dose of 110 Gy (similar to the natural equivalent doses) were given to the remaining aliquots. The resulting dose recovery ratios (measured dose corrected for residual dose by subtraction and subsequently divided by given dose) are calculated using the arithmetic mean dose. The average dose recovery ratios are shown in Figure SI.20b. The dose recovery ratio for the pIRIR$_T$ signal appears to increase with stimulation temperature, whereas it for the IR$_{50}$ signal appears to decrease with temperature. Here we choose a standard preheat of 320°C for 60 s, a first IR stimulation temperature of 50°C and a post IR stimulation temperature of 290°C.

In addition, a pIRIR$_{290}$ dose recovery with a given dose of 1150 Gy (n=6) was undertaken to investigate if we are able to recover doses in the high dose range as well. Dose recovery ratios of 0.60 ± 0.02 and 0.95 ± 0.03 were obtained for IR$_{50}$ and pIRIR$_{290}$, respectively. The corresponding light level ratios (i.e., the ratio between the sensitivity corrected "natural" signal to a sensitivity corrected signal from a regeneration dose equal to the "natural" dose) were 0.840 ± 0.012 and 1.009 ± 0.007, respectively. For these measurements, the D_c values were 524 ± 10 and 488 ± 9 (n=6) for IR$_{50}$ and pIRIR$_{290}$, respectively. So in the laboratory, we are for the pIRIR$_{290}$ signal able to recover doses up to at least $2.4D_c$. However, it is worth noting that all aliquots gave bounded dose estimates.

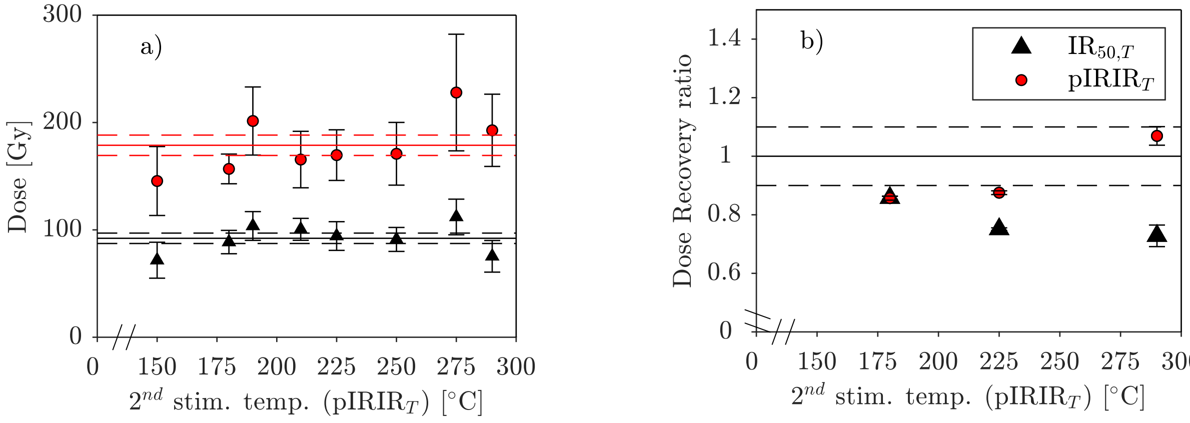

Figure SI.20: a) Equivalent doses (red circles, corrected for residual dose) for sample 187323 as a function of pIRIR stimulation temperature T (the first IR stimulation temperature was kept constant at 50 °C). The preheat temperature was 20 °C higher than the pIRIR stimulation temperature, except for pIRIR$_{290}$, where the preheat temperature was 320 °C. Also shown are the equivalent doses from the first IR stimulation at 50 °C (grey triangles). Each point is an average of three aliquots. The full horizontal lines show the average equivalent doses for pIRIR$_T$ (red) and IR$_{50}$ (black), respectively. The dashed lines show the standard error on the mean. b) Dose recovery ratios (corrected for thermal transfer) for different post IR stimulation temperatures for samples 187323, 16725, 167827, 167830: pIRIR$_{180}$ (n=12), pIRIR$_{225}$ (n=48), pIRIR$_{290}$ (n=12).

Multi-grain K-rich feldspar dose estimation

Between 6 and 24 multi-grain K-rich feldspar aliquots were measured for all samples analysed at DTU, Denmark. The results are summarised in Table SI.5. As for the quartz multi-grain measurements, we used the IQR criterion to objectively remove outliers. A total of 17 and 13 dose estimates (for IR$_{50}$ and pIRIR$_{290}$, respectively) were removed in this way, from a total of 462 and 446 dose estimates, respectively. The average doses measured for the

IR_{50} signal range between 36±3 Gy (sample 167831, unit 1) and 452±41 Gy (sample 167820, unit 5), whereas for the $pIRIR_{290}$ signal they range between 88±6 Gy (sample 167831, unit 1) and 1868±86 Gy (sample 197339, I.C.). The resulting dose distributions for the IR_{50} signal have relative standard deviations ranging between 4% (sample 167829) and 56% (sample 187311) with an average value of 20%. For this signal, we could not derive bounded dose estimates for a small fraction of the aliquots (∼0.2% on average for IR_{50} signal). The resulting dose distributions for the $pIRIR_{290}$ signal have relative standard deviations ranging between 2% (sample 167829) and 36% (sample 197340) with an average value of 13%. For this signal, we could not derive bounded dose estimates for ∼7 % (on average) of the measured aliquots. In fact, for the $pIRIR_{290}$ signal, nine samples (two samples from unit 5 in LRC IV, all six samples from inside LRC I (I.C. and E.C.) and sample 187307 from unit 4) give doses greater than 800 Gy. The fraction of saturated aliquots in these samples ranges between 17% and 58% and we regard the KF doses for these samples to be significantly affected by saturation effects, i.e., the ages from these samples are regarded as minimum ages (as are the corresponding quartz ages).

In Figure SI.21, we plot the IR_{50} doses against the $pIRIR_{290}$ doses. The dose recorded by the IR_{50} signal results from a competition between charge storage (from dose rate) and charge loss (from fading). The charge storage rate is (assumed) constant, but the loss due to fading depends on the total stored charge, and so the loss rate increases with dose. This eventually results in a constant IR_{50} dose, at some level well below the laboratory saturation (for which fading is negligible). This is what is seen at higher doses in Figure SI.21 (compared with the typical laboratory dose response curve shown in Figure SI.19). In Figure SI.21, the data have been fitted using a saturated exponential function and show, as expected, a significant deviation from the 1:1 line even at low doses, because the IR_{50} signal is prone to significant anomalous fading. The bleaching rate of the $pIRIR_{290}$ signal is known to be approximately 1 order of magnitude slower than that of the IR_{50} signal [(e.g., 34)] and thus by comparing the IR_{50} and $pIRIR_{290}$ doses it may be possible to determine whether a given sample is suffering from incomplete bleaching. Buylaert et al.[(55)] also fitted a saturating single exponential to their data and rejected all data below 10% of their fitted line. This arbitrary but objective approach removed stratigraphic age outliers. In our data, the IR_{50} dose estimates all underestimate those from the $pIRIR_{290}$ data, but there are no significant outliers below the fitted line. The exponential fit suggests that at low doses the underestimate is about 40%, consistent with typical IR_{50} fading rates (see also next section). From these data we conclude that we have no evidence for significant differential bleaching between the two feldspar signals. Given that the IR_{50} signal bleaches approximately ten times faster than the $pIRIR_{290}$ signal, this observation is most likely explained by both signals being completely bleached before burial. This conclusion is supported by the observation that the feldspar ages are below or equal to the quartz ages, in the dose range over which quartz is considered reliable (see section *Multi-grain K-rich feldspar ages compared to multi-grain quartz ages*).

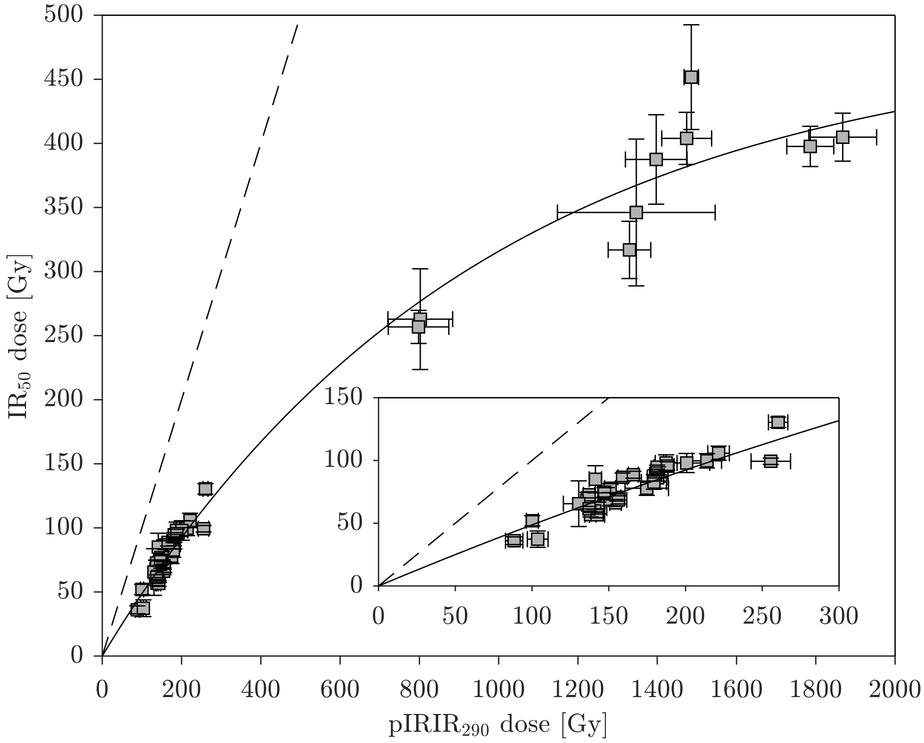

Figure SI.21: IR_{50} doses plotted as a function of $pIRIR_{290}$ doses. The solid line is a saturating exponential fitted to the data, whereas the dashed line represents the 1:1 line. The inset shows the same data for $pIRIR_{290}$ doses less than 300 Gy.

Multi-grain K-rich feldspar fading correction

K-rich feldspar luminescence signals are known to suffer from anomalous (athermal) fading [(e.g., 56)] and several models have been developed to correct for instability in the IRSL signal [(e.g., 57)]. Anomalous fading is usually quantified by the g-value (signal loss per decade of logarithmic normalised storage time) and we measured g-values for samples 207307 and 197328 following the approach of Auclair et al. [(58)] using given doses of 190 Gy. We determined average g-values of 2.66 ± 0.12, n=34 (IR_{50}) and 1.30 ± 0.05, n=34 ($pIRIR_{290}$). In accordance with Buylaert et al. [(34)] we fading correct the IR_{50} ages but not the $pIRIR_{290}$ ages. The IR_{50} ages are on averaged increased by 30% as a result of this fading correction. If we were to fading correct the $pIRIR_{290}$ ages, they would on average increase by 13%.

Luminescence ages

All burial ages derived from sediment samples are derived from the equivalent dose divided by the total dose rate for each sample. A summary of all multi-grain doses, dose rates and burial ages are found in Tables SI.2 and SI.5. The multi-grain OSL quartz ages processed in Hungary are included in these tables as well.

In the following sections, we first evaluate the quartz ages derived from LRC I, II and IV, compare KF and quartz multi-grain ages and then evaluate the effect of K-concentration on the KF to quartz age ratio (section: *Multi-grain K-rich feldspar ages compared to multi-grain quartz ages*). The effect of water content on burial ages is considered in section: *Water content correction.* In section: *Bayesian modelling*, age-depth modelling is performed using Bayesian Statistics (Bacon script [(59)]), combining the multi-grain quartz ages for LRC I and II to find the best estimate of the closure of the cave (LRC I). Bayesian age-depth modelling is performed separately on the samples from LRC IV.

Multi-grain quartz ages

In LRC I, the multi-grain quartz ages measured in Denmark (n=13) range between 42±3 ka and 76±6 ka (excluding the samples taken inside the cave labelled I.C. and E.C. identified with no specific unit, respectively) and they are all in stratigraphic order (with respect to elevation and unit identification).
The multi-grain quartz ages measured from the samples from inside the cave (except samples 227801 and 227802) range between 167±18 ka and 317±53 ka. For these samples bounded dose estimates could not be derived for between 17% and 69% of the measured aliquots and are all considered to be minimum ages. These samples are not in stratigraphic order and may be relicts from previous cave filling events. This is further supported by the fact that these samples could not be identified as belonging to a specific unit. However, two samples (227801 and 227802) taken from two niches at the back of the entrance (see Fig. 7) could clearly be identified as belonging to unit 4 and give ages of 58 ± 4 ka and 63 ± 4 ka, respectively.

In LRC II, the multi-grain quartz ages measured in Denmark (n=12) range between 84±5 ka and 327±23 ka; they are in stratigraphic order except for two samples: 187323 (unit 5, 121±8 ka) and 187307 (unit 4, 327±23 ka).
With respect to sample 187323, then in LRC II, there is one other sample (187304) also collected from unit 5. This sample is located almost two meters below sample 187323 at the bottom of the section and gives an age of 121±11 ka, which is consistent with the age derived from sample 187323. From Fig. 6 it is apparent that sample 187323 was taken in a pocket of relict unit 5 sediment, and thus one would expect this sample to yield an age significantly older than the ages derived from the unit 4 samples located immediately above and below, e.g., sample 187320 (Fig. code 23) just above has an age of 84±5 ka and sample 187310 (Fig. code 25) has an age of 88±5 ka.
The multi-grain quartz age of 327±23 ka derived for sample 187307 (Fig. code 33) is considered to be a minimum age, because bounded dose estimates could not be derived for 17% of the measured aliquots. This sample was collected close to bedrock but in the same layer as sample 207307 (Fig. code 31); the latter gives an age of only 97±6 ka. However, in the sample description notes, sample 187307 is described as being a mix of fluvial sand and with sand possibly derived from disaggregated bedrock, whereas sample 207307 is described as a pure fluvial sand. If bedrock is present in sample 187307, it is reasonable to assume that the estimated equivalent dose will overestimate the burial dose. Since the bedrock also has a lower radionuclide concentration, the infinite matrix dose rate would also be expected to be lower. Indeed, the infinite matrix dose rate derived for sample 187307 is only ∼40% of that derived for the pure sediment sample (see Table SI.2).
Thus, there are good reasons for discarding the two samples 187323 and 187307. Then the remaining multi-grain quartz ages for LRC II are in stratigraphic order.

In LRC IV, the multi-grain quartz ages measured in Denmark (n=12) range between 27.8±1.7 and 184±16 ka, with all samples appearing in stratigraphic order with the possible exception of sample 167819 (153±14 ka), which was collected 80 cm below sample 167820 (184±16 ka). However, both these sample are from unit 5 and are regarded as minimum ages as bounded dose estimates could not be derived from ∼30% of the measured aliquots.

Thus, in summary, the multi-grain quartz ages for the individual sites in LRC are in stratigraphic order.

Multi-grain K-rich feldspar ages

In LRC I, the KF ages range between 16±3 ka and 43±4 ka, and between 34±3 ka and 71±6 ka, for IR_{50} and $pIRIR_{290}$, respectively (excluding the samples taken inside the cave itself). With respect to elevation, they are in stratigraphic order except for samples 187311 and -12. The ages derived from these samples are ∼35% lower than would be expected based on the other ages in the section - particularly sample 167805 (71±6 ka) and sample 167812 (65±7 ka).
The KF ages measured from the samples from inside the cave with no identified unit(I.C. and E.C.) range between 141±24 ka and 241±24 ka and between 325±41 ka and 834±78 ka for IR_{50} and $pIRIR_{290}$, respectively. Only the three samples from the entrance (E.C.) are in stratigraphic order (with respect to elevation). For the $pIRIR_{290}$ measurements bounded dose estimates could not be derived for between 17% and 58% of the measured aliquots and thus they are all considered to be minimum ages. Between 0% and 8% of the measured IR_{50} doses gave bounded dose estimates.

In LRC II, the KF ages (n=12) range between 40±3 ka and 201±19 ka and between 59±4 ka and 473±59 ka for IR_{50} and $pIRIR_{290}$, respectively, and are in stratigraphic order except for two samples: 187323 and 187307. Based on

the arguments given in section *Multi-grain quartz ages*, it is expected that these sample will be out of stratigraphic order.

In LRC IV, IR_{50} and $pIRIR_{290}$ ages are in stratigraphic order, but the ages from the bottom samples (167820 and -19) are regarded as minimum ages because of saturation effects (i.e., $\sim 30\%$ of the aliquots did not give bounded dose estimates).

Multi-grain K-rich feldspar ages compared to multi-grain quartz ages

In this section, feldspar ages are compared to quartz ages to evaluate whether the sediments were well-bleached at the time of deposition.
In Figure SI.22, feldspar ages (using a measured average K-content of 12.6%) are plotted against quartz ages. The feldspar to quartz age (FQ) ratio is on average 0.57 ± 0.04 (n=43) for ages based on the IR_{50} signal (corrected for fading using a g-value of 2.66 ± 0.12) and 1.19 ± 0.13 (n=43) for ages based on the $pIRIR_{290}$ signal. However, all the samples from I.C. and E.C., from unit 5 in LRC IV and sample 187307, have quartz ages above 150 ka (and $> 15\%$ of the measured aliquots in saturation and doses above 225 Gy) and are likely to be significantly affected by saturation effects (see section: *Multi-grain quartz dose response and stimulation curves*). When these samples are removed from the average FQ ratios, they become 0.482 ± 0.011 (n=34) for IR_{50} and 0.811 ± 0.018 (n=34) for $pIRIR_{290}$. Thus, the feldspar ages underestimate the quartz ages significantly. Possible causes for this underestimation are considered below.
However, for the main question addressed here, concerning the closure of the cave entrance, seven samples are of particular importance, i.e., samples 197332, -28, -33 and 167806 and -05, 227801 and 227802. These are located between 1 m above and below the elevation at which the cave was closed. For these crucial samples, the FQ ratio for the $pIRIR_{290}$ signal is 0.94 ± 0.02.

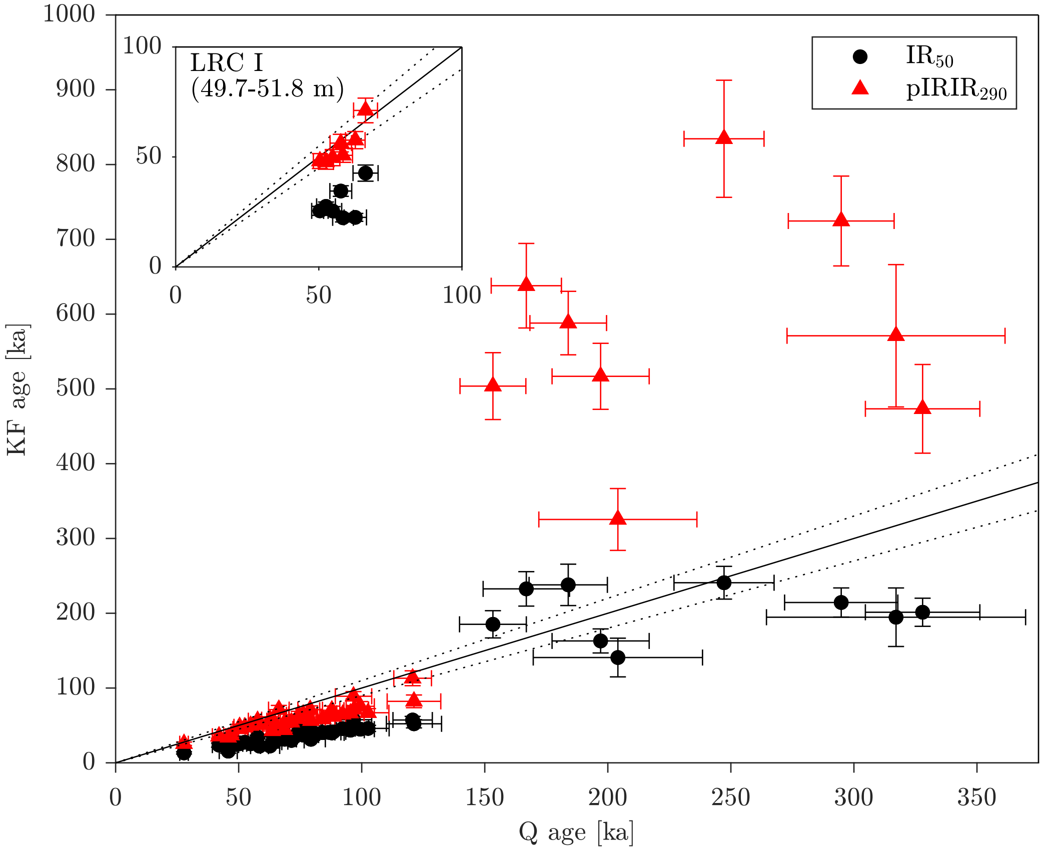

Figure SI.22: Feldspar (KF) ages plotted against quartz (Q) ages. IR_{50} ages (black symbols) are corrected for fading using a g-value of 2.66 ± 0.12. $pIRIR_{290}$ ages (red symbols) are not corrected for fading. Also shown is the 1:1 line (solid line) with a ±10% uncertainty interval (dashed lines). Inset shows same data for samples located 1 m above and below the elevation of 50.75 m (i.e., the elevation at which the entrance in LRC I was sealed).

One possible explanation for this underestimation could be that the assumed K-content (used in the dose rate calculation) is incorrect. The effect of changing the K-concentration on the FQ ratios is shown in Figure SI.23a. Note that the ^{87}Rb concentration changes in proportion to the K-concentration [57] and that samples with quartz doses above 225 Gy (expected to be significantly affected by saturation effects) have been excluded in Figure SI.23. A FQ ratio of unity cannot be obtained for the IR_{50} signal by simply changing the assumed K-concentration, but is obtained if a K-concentration of 5% were to be used for the $pIRIR_{290}$ signal (see Figure SI.23a).

Given the variability in the external dose rates (Table SI.2) one would expect the variability in the FQ ratio to be at a minimum when the correct K concentrations are employed. Since we employ a single (average) K-concentration for all samples in our analysis this would only be true if all feldspar samples have, in fact, a similar effective K concentration. In Figure SI.23b, we show the relative standard deviation (RSD) of the FQ ratio as a function of K-concentration only using samples with Q doses $<$ 225 Gy. A minimum RSD is found for a K-concentration of ∼1% and ∼2% for the $pIRIR_{290}$ and IR_{50} ratios, respectively. These K-concentrations correspond to internal dose rates from ^{40}K and ^{87}Rb of only 0.069 ± 0.002 Gy.ka^{-1} (1% ^{40}K, 32 ppm ^{87}Rb) and 0.137 ± 0.005 Gy.ka^{-1} (2% ^{40}K, 64 ppm ^{87}Rb), respectively , and is very different from the 0.86 ± 0.03 Gy.ka^{-1} derived from the measured concentration of 12.6% K and 400 ppm ^{87}Rb.

Another possible explanation for the underestimation of the KF ages is that the used grain size is incorrect, since reducing the grain size in the dose rate calculation would result in smaller internal dose rates from ^{40}K and ^{87}Rb in feldspar. In fact, an internal dose rate of 0.069 Gy.ka^{-1} is derived if a grain size of 10-20 µm is used (as opposed to the actual fraction of 180-250 µm) in combination with a K-concentration of 12.6% and 400 ppm of ^{87}Rb. In this case, the FQ ratio would be 1.14 ± 0.02 ($pIRIR_{290}$ signal).

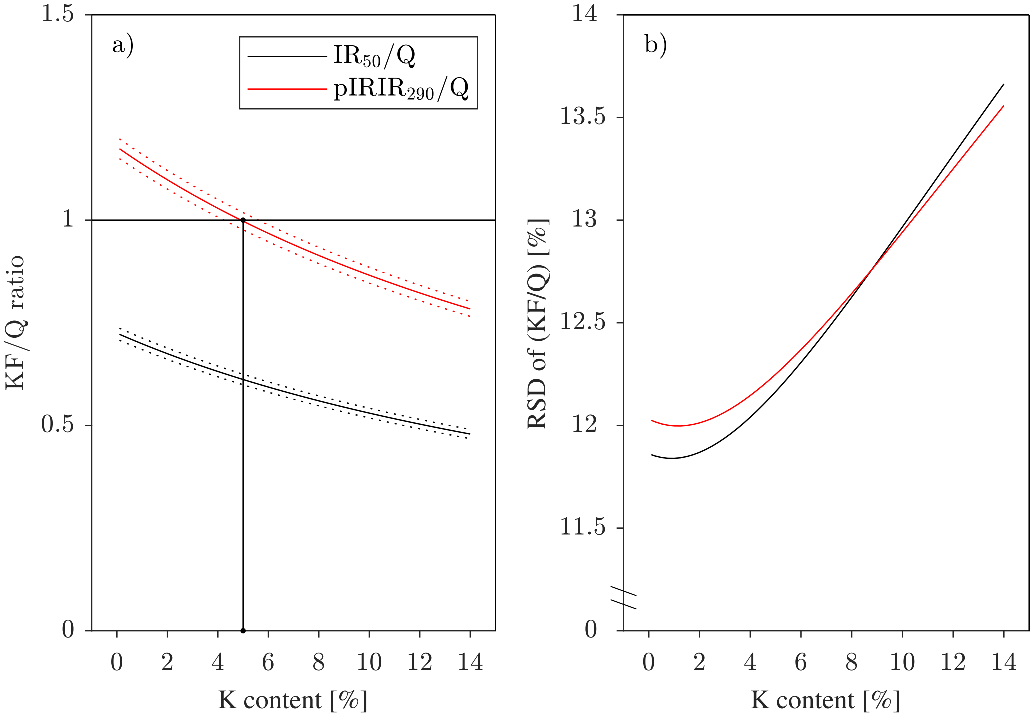

Figure SI.23: The average effect on the feldspar to quartz age (FQ) ratio when changing the assumed ^{40}K and ^{87}Rb concentrations. The ^{87}Rb concentrations are changed in proportion to the ^{40}K concentration. Only samples with quartz doses < 225 Gy have been included ($n = 34$). FQ ratios using the fading corrected IR_{50} ages are shown as solid black lines, whereas those based on the $pIRIR_{290}$ ages are shown as solid red lines. a) Average FQ ratio. Standard errors are shown as dotted lines. Also shown is the K-concentration value for which the FQ ratio is unity b) Relative standard deviation of the average FQ ratio.

Whichever K-concentration or K-rich feldspar grain size is appropriate, we can in any case conclude that, for samples with a quartz $D_e < 225$ Gy (i.e., those unlikely to be affected by saturation issues) the feldspar ages do not overestimate the quartz ages, and so it is very likely that the quartz (and the feldspar) was well-bleached at the time of deposition.

Water content correction

Current and saturated water contents (w.c.) were measured for all samples and the results are shown in Figure SI.24. The current water contents are likely to underestimate the average life-time water content, mainly because of significant drying after excavation - both on site and in the laboratory. Therefore, the true water content is assumed to lie in between the current and saturated water content and it is assumed to be the same fraction of saturation for all samples. Two samples (167819 and 167820) from LRC IV were surrounded by bedrock underneath and on the sides (see Figure 9) and these samples are therefore more likely not to have dried significantly after excavation, i.e., the measured current water content is likely to better represent the present day w.c. These samples are marked by red (open and closed) squares in Figure SI.24. The ratios between the current and saturated water contents are 0.51 and 0.32 for samples 167819 and 167820, respectively, with an average of 0.41±0.10 (n=2). Thus, in the dose rate calculations we assume that a fractional water content of 40% of the laboratory-measured saturated water content taken as a mean of each unit (1-5). Below, we examine the consequences of using different water content assumptions on the derived ages.

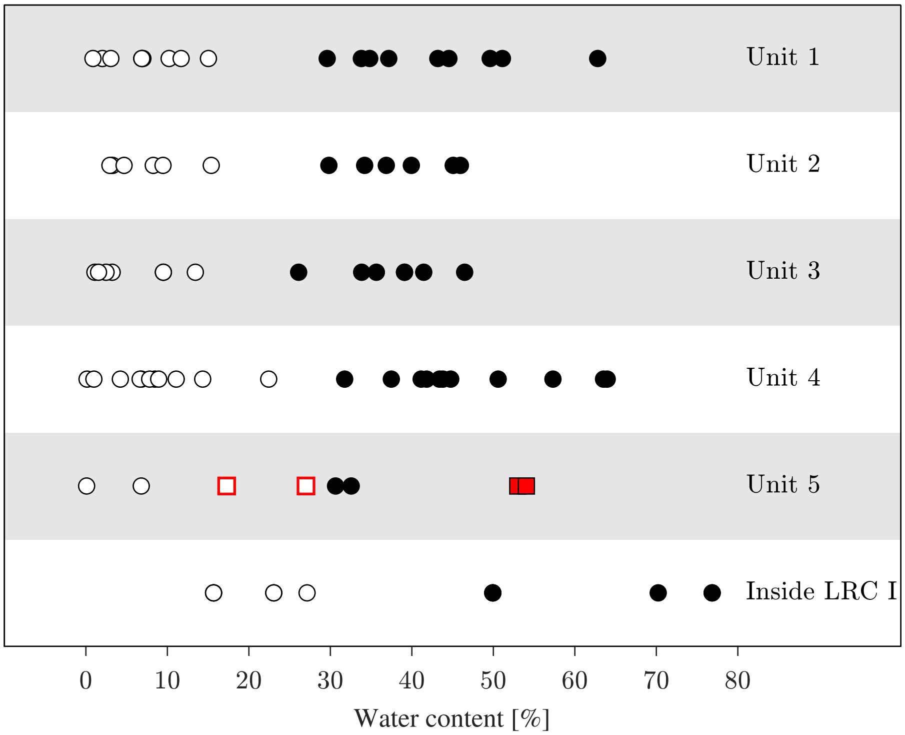

Figure SI.24: Measured current (open symbols) and saturated (closed symbols) water content. Squares indicate samples 167819 and 167820, which were taken from the bottom of LRC IV, where they were surrounded by bedrock on three sides. It is therefore less likely that these sample have experienced significant drying since excavation.

To investigate the effect of the choice of w.c. on the age calculation, the average ratio ($Q_{x,w.c.}/Q_{40,w.c.}$) of the quartz ages for different fractional saturation w.c. (x) to the quartz ages with a fractional saturation w.c. of $40 \pm 10\%$ have been calculated.

Figure SI.25a shows $Q_{x,w.c.}/Q_{40,w.c.}$ as a function of fractional saturation w.c. ranging between zero and unity, i.e., the w.c. ranges between 0 and 100% of the saturated water content. The ratio is shown both for fractional saturation w.c. using the measured saturated w.c. for individual samples (black line) as well as the average saturated w.c. for each of the five depositional units and the samples inside LRC I identified in the field (red line). Only samples with quartz doses less than 225 Gy are used in this analysis, to ensure that proximity to dose saturation does not influence the ratios. In the investigated range, the average relative quartz age changes between 0.84 and 1.23, but there is no significant difference between using the individual measured saturated w.c. or the average saturated w.c. for each depositional unit when calculating the fractional saturation w.c.

Also in the relative standard errors (RSE) of the mean of quartz ages for different fractional saturation w.c. (Figure SI.25b) there is not a significant difference between using the unit mean saturated w.c. or the individual saturated w.c. for calculating the assumed fractional w.c.

As we see no minimum value, we can not from the analysis of the RSE justify whether the choice of w.c. plays a significant role in the observed variability of the ages, but it is clear that using the current w.c. would increase the variability between the ages, i.e., the RSE is higher when using the current w.c. compared to using the value chosen (40% of saturation).

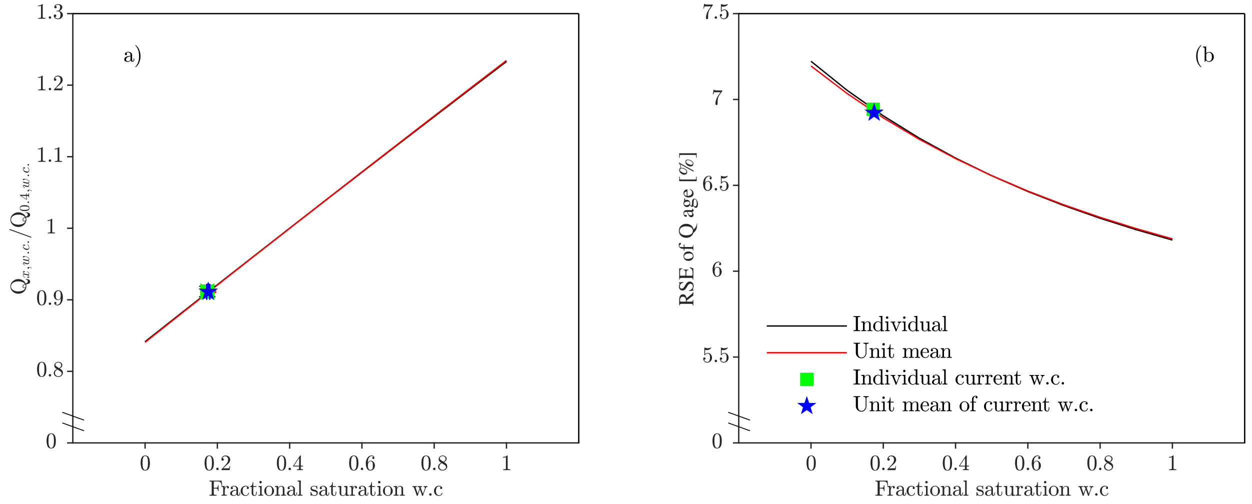

Figure SI.25: a) Effect of water content on a) the average ratio of quartz ages using fractional w.c. x to quartz ages for a fractional w.c. 0.4 ± 0.1, and on b) relative standard error (RSE) of quartz ages for different fractional w.c. x (i.e., fraction of saturated w.c.) Only samples with a quartz dose < 225 Gy have been included in the analysis (n=34, i.e., samples where the quartz signal is suspected of being in saturation has been removed, see section: *Multi-grain quartz OSL measurements* for further details). Red line: using the average saturated water content for each unit. Black line: using individual saturated water contents. The result of using the current w.c. for individual samples or the average of current w.c. in each unit are shown as a green square and a blue star, respectively. Uncertainties on the mean i a) are small compared to the with of the lines.

We have chosen to use a fractional water content of 0.4±0.1. The uncertainty of 0.1 corresponds to a ± 4% uncertainty in age. We are confident that the average life-time water content is higher than the measured current w.c. which, on average, is 7.3 ± 0.9% (n=34). If the measured current w.c. is used (instead of the fixed fractional w.c. of 0.4), the quartz ages will on average be reduced by 9%. In contrast, using a (very unlikely) fractional w.c. of 1 (i.e., saturated) increases the ages by 23%.

In any case, we assume it is more likely that each unit has had the same fractional w.c. during burial and we use a fractional w.c. of 40 ± 10% of the saturated value averaged over each unit in the age calculations.

Quartz OSL ages from samples from LRC IV and the seven samples processed by the Hungarian laboratory have been published in 2019 [(2)] and 2016 [(3)], respectively. In these publications current water contents were used. However, as argued previously, the current water content almost certainly underestimates the average water content during the burial life-time, and thus we recalculated these ages here using what we consider to be the more realistic water content assumption (see above and Figure SI.6). In addition, the ages presented in the previous publications were not corrected for the dose rate heterogeneity - although it must be recognised that the samples from LRC IV are not significantly affected by this heterogeneity. Finally, in previous publications the internal dose rates to quartz were considered to be 0.06 Gy.ka^{-1} as opposed to the more current value of 0.02±0.01 Gy.ka^{-1} [(35)] used here. The effect of using the higher w.c. (around 10% higher) and dose rate model corrections is an average increase in age of 12% (n=15) and 11% (n=7) compared to the ages published in 2019 and 2016, respectively.

OSL ages of La Roche-Cotard

Both single-grain quartz and multi-grain pIRIR(50,290) measurements show that the multi-grain quartz ages are likely to have been well-bleached at burial and thus, in the following we only consider the quartz multi-grain ages.

In Figure SI.26 all multi-grain quartz ages from the four LRC sites, grouped into the five different deposition units (U1-5), are shown. For a given unit, the ages obtained from the different sites agree well with each other. Note that ages considered to be a minimum value due to quartz saturation are marked with open symbols in Figure SI.26). Ages from LRC IV agree with ages from LRC I and LRC II. Also the single age from LRC III agrees with the other ages in same unit (unit 4). This implies a connection in the deposition of the sites.

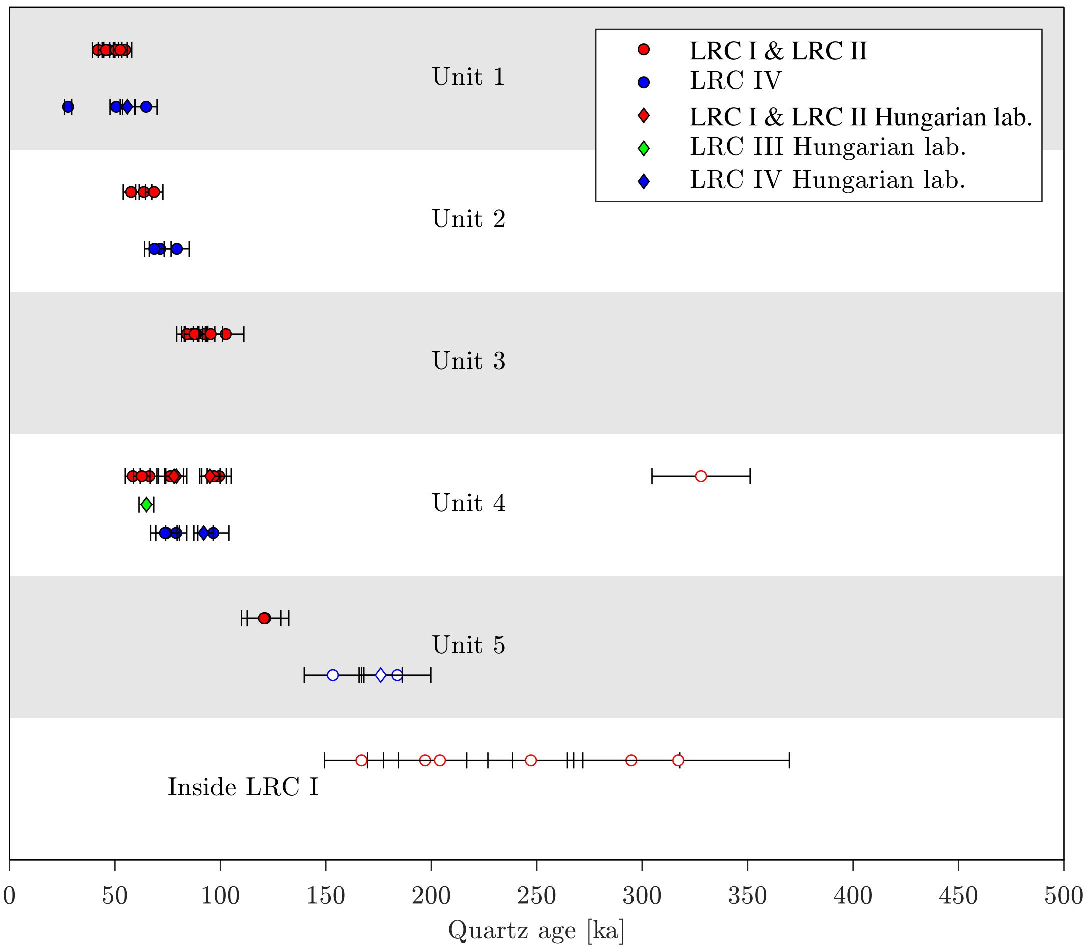

Figure SI.26: Multi-grain quartz ages grouped according to deposition unit (U1-5) and for samples from inside LRC I (not identified as belonging to a unit, i.e., I.C. and E.C.). For each unit, the individual ages have been grouped vertically according to site. LRC I & II (red symbols), LRC IV (blue symbols) and LRC III (green symbol). The samples processed in Denmark are shown as circles. The samples processed in Hungary are shown as diamonds. Open symbols indicate minimum ages due to dose saturation effects. Uncertainties on individual ages are given at 68% confidence.

Bayesian modelling

To obtain a more precise estimate of the closure age, we model the age/depth relationship using Bayesian statistics (Bacon script [59]) with the elevation of the individual samples as priors. This is undertaken using only multi-grain quartz ages, as these are considered the most reliable in this age range.

Because of the relative lateral and vertical positions of the four sites (LRC I, II, III and IV), data from LRC I and II are combined for modelling, whereas LRC IV is modelled separately. LRC III does not overlap the other sites and the single sample measured in Hungary for this site is not included. Modelling only makes use of the samples processed in Denmark, but those processed in Hungary are subsequently compared with the modelled results.

The results of the Bayesian modelling are shown in Figure SI.27 for LRC I and II (and Figure 8), and Figure 9 for LRC IV. The full black lines in Figure SI.27 (and Figure 8) and Figure 9 represent the most likely age model based on a water content of 40±10% of the average saturated water content for each unit. All samples which gave average equivalent doses >225 Gy, i.e., all samples from unit 5, but two (see below), from inside the cave, and sample 187307, are considered to be significantly affected by saturation issues and are not included. Model fitting makes use of random uncertainties only - after fitting, the systematic uncertainties associated with beta calibration, internal dose rate, cosmic rays and water content are added to the fitting uncertainty. The resulting total uncertainty is plotted as dotted lines (one standard error). The uncertainties on individual data points are also total standard errors (at 68% confidence).

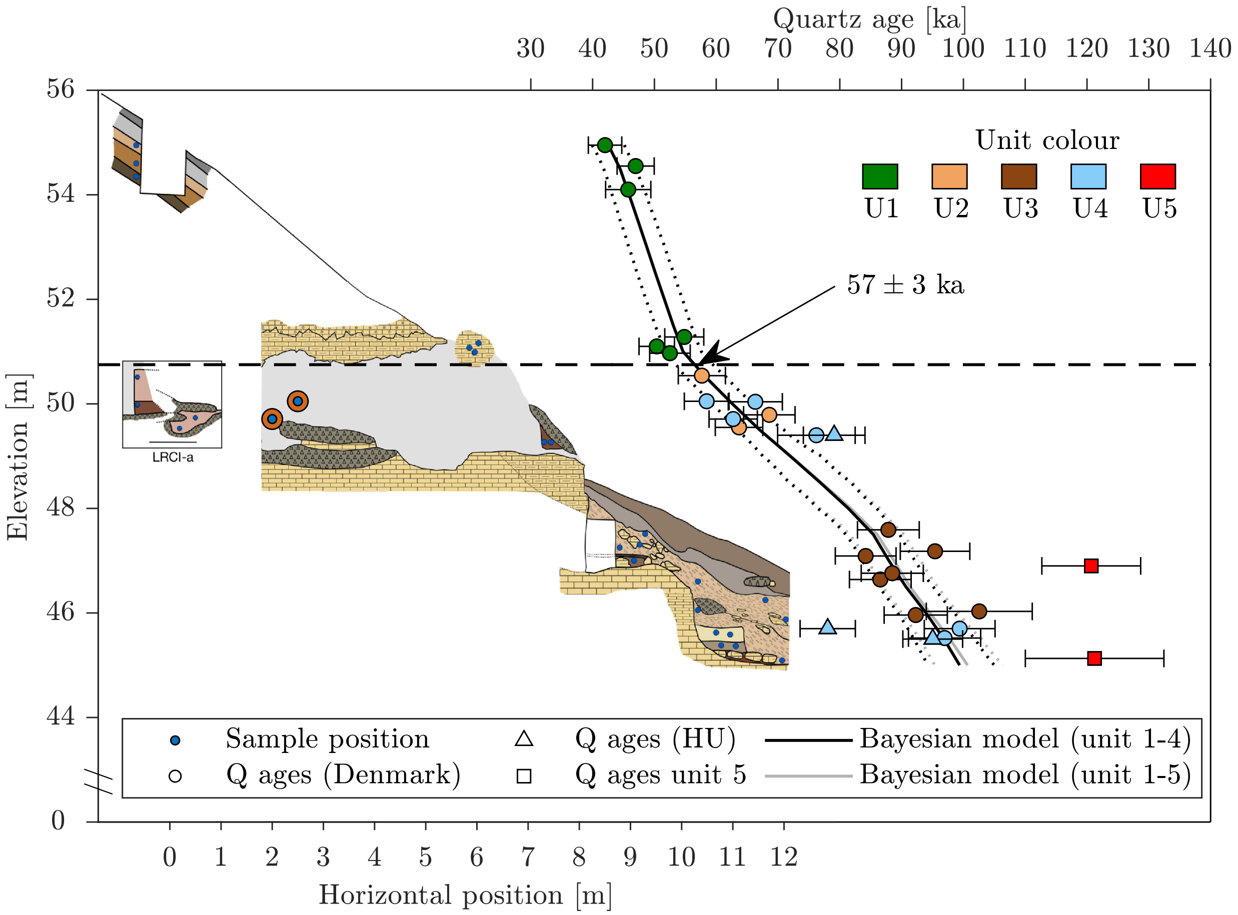

Figure SI.27: Schematic drawing of LRC I and LRC II lithographic section and multi-grain quartz ages from unit 1-4. OSL sample positions (blue points) are shown to the left on the schematic drawing of LRC I and LRC II. The right-hand side of the figure shows the 25 multi-grain quartz ages from unit 1-4 (circles and triangles: measured in Denmark and Hungary, respectively). The individual units can be identified according to colour, i.e., unit 1-5 is green, light brown, dark brown, light blue, and red, respectively. A Bayesian model (Bacon script[59]) using the elevation as prior and only random uncertainties for the individual ages is shown (grey and black lines). The grey line shows the model for unit 1-5, whereas the black line shows the model for unit 1-4 only. Dotted lines show the total uncertainty (including both random and systematic uncertainties) at the 68% confidence interval. The OSL ages measured in Hungary are not included in the Bayesian model. The dashed black horizontal line indicates the cave ceiling elevation in LRC I. The insert shows LRC I-a (see Fig 6 in the main text) which includes two samples inside the cave (167805 and -06) and two outside the cave (187311 and -12). The adopted water content is 40±10 % of the average measured saturated water content for each deposition unit.

OSL samples processed in Hungary (triangles in Figure SI.27, Figure 8 and 9 in main paper) are in good agreement (95% confidence) with the Bayesian model except for one (181362, unit 4 in LRC II).

As mention above, only two samples from unit 5 (187323 and 187304, both LRC II) gave reliable quartz equivalent doses. The two corresponding ages are shown in Figure SI.27 as red squares. These samples have not been included in the Bayesian model (black line), because their elevation does not represent the deposition level with respect to vertical position. However, including them in the model does not change the overall model significantly (see grey line in Figure SI.27)

The limiting ages for deposition units 1-4 are estimated from the Bayesian models from i) LRC I and LRC II and ii) from LRC IV using the vertical positions (see Table SI.1) of the units in the respective sites. The minimum and maximum ages from each unit are given in Table SI.8 (see also Figure 9).
The minimum age for unit 5 is given from the minimum quartz age of sample 187323 and 187304 (LRC II, see Table SI.8). Samples from unit 5 in LRC IV are regarded as minimum ages due to saturation effects. Since these minimum ages are larger that the maximum ages from samples 187323 and 187304 in LRC II, a maximum age cannot be given for unit 5.

Table SI.8: Minimum and maximum ages for LRC. The "Age range" is derived from the minimum ("Min. age") and maximum ("Max. age") ages determined across sites at the 68% confidence level using the Bayesian models for LRC I & II and LRC IV, respectively. "Site" gives the site (LRC I, II, or IV) from which the minimum and maximum ages were determined for the given "elevation". The minimum age for unit 5 is derived from sample 187304. No maximum age can be given for this unit because of quartz saturation effects.

		Min. ages			Max. ages		
Unit/level	Age range [ka]	Elevation [m]	Site	Min. age [ka]	Elevation [m]	Site	Max. age [ka]
Unit 1	25- 66	50.10	LRC IV	28±3	47.00	LRC IV	62±4
Unit 2	54- 80	50.70	LRC I	57±3	44.90	LRC IV	76±4
Unit 3	77-104	48.00	LRC II	81±5	45.13	LRC II	99±5
Unit 4	57-103	50.29	LRC I	60±3	45.30	LRC II	98±5
Unit 5	>110	45.13	LRC II	121±11	-	-	-
Mousterian	65-104	49.30	LRC I	69±4	45.20	LRC II	98±5

The cave (LRC I) was completely closed when the sediment reached an elevation of 50.75 m NGF (see Figure SI.27 and Figure 8). From the age model, this is most likely to have occurred 57 ± 3 ka ago (68% confidence level). This age is based on the assumption of a water content of 40% of saturation. Using the lowest possible water content (current water content, see Table SI.2) gives a burial age for the entrance of 52 ± 3ka (68% confidence level), but this fractional water content is considered highly unlikely.

In conclusion, based on the most likely values of water content, the minimum closure age for the cave entrance is >51 ka at 95% confidence.

Table SI.9: Ages derived from Bayesian model ('Bayesian Ages') for altitudes corresponding to sample positions.

Sample	Elevation [m]	Sample age [ka]	Bayesian age [ka]
187301	54.95	42±3	43±3
187302	54.55	47±3	44±3
187303	54.1	46±4	46±3
197332	51.28	55±3	54±3
197328	51.1	50±3	54±3
197333	50.97	53±3	55±3
197340	50.78	>317	-
197338	50.66	>167	-
197339	50.57	>247	-
167806	50.54	58±4	58±3
167809	50.2	>197	-
167817	50.2	>204	-
167818	50.2	>295	-
227801	50.05	58±4	62±3
167805	50.04	66±4	62±3
187312	49.79	69±4	64±3
227802	49.71	63±4	65±3
187311	49.55	64±4	67±4
161267*	49.4	79±5	68±4
167812	49.4	76±6	68±4
187321	47.59	88±5	85±4
187322	47.18	95±6	87±4
187320	47.09	84±5	87±4
187323	46.9	121±8	88±4
187310	46.76	88±5	89±4
187306	46.64	87±5	90±4
187309	46.03	103±9	94±5
187305	45.96	92±5	94±5
181362*	45.7	78±4	96±5
187308	45.7	99±6	96±5
207307	45.52	97±6	97±5
181361*	45.5	95±5	97±5
187307	45.18	>328	-
187304	45.13	121±11	99±5
161263*	45.2	65±4	-
167831	49.8	27.8±1.7	30±3
167830	48.2	51±3	51±3
161264*	47.2	56±3	60±4
167829	46.5	65±5	66±4
167828	46.2	69±5	69±4
167827	46	71±5	70±4
167826	44.9	79±6	76±4
161266*	44.6	92±5	78±4
167823	44.6	74±5	78±4
167825	44.6	74±7	78±4
167822	44.5	79±5	79±4
167821	44.1	97±7	81±5
167820	43.3	>184	-
161265*	42.5	>176	-
167819	42.5	>153	-

Summary

This study uses the OSL signals from multi- and single-grain quartz aliquots, and the IR_{50} and $pIRIR_{290}$ signals from K-rich feldspar (KF). All equivalent doses are measured using standard SAR protocols. The dose rates are derived from radionuclide concentrations and corrected for heterogeneity in the gamma field (section: *Correction for heterogeneity in the gamma field*). A water content of 40% of the measured saturated water content (averaged for each deposition units and inside LRC I, I.C.) has been adopted, based on the current values for two deepest samples least likely to have been affected since excavation of LRC IV in 2008 (see section: *Water content correction*).
In the dose rate calculations, a grain size of 180-250 µm is used. We assume an internal dose rate to quartz of 0.02±0.01 Gy.ka^{-1} and to KF of 0.10±0.05 Gy.ka^{-1} from U and Th. The cosmic dose rate contribution is calculated for each individual sample (the average contribution for all samples is 0.064 ± 0.003 Gy.ka^{-1}). KF ages also include an internal dose rate component of 0.86±0.06 Gy.ka^{-1} derived from the measured average K-content of 12.60±0.15% (n=21) and a Rb-content of 400 ppm (see section: *Dose rate measurements*).

For those sediment samples taken close to the rock walls or large clasts, the infinite matrix dose rates derived from radionuclide concentrations were modified to take heterogeneity in the gamma radiation field into account, using the principle of superposition. Modelled results were tested against Monte Carlo simulations and in situ dose rate measurements, both of which supported the use of the simpler analytically modelled dose rates.
Note, that in the section, *Comparison with in situ dose rate measurements*, we discussed the reliability of our laboratory calculated dose rates when compared to the dose rates measured in situ. On average, we find a good agreement between the two dose rate estimates (1.02 ± 0.09, n=8), but for sample 167812, there was a significant and large difference between the in situ (LaBr probe) and the laboratory calculated gamma dose rates (with the cosmic contribution removed and current w.c. assumed), i.e., 0.323 ± 0.008 Gy.ka^{-1} and 0.48 ± 0.03 Gy.ka^{-1}, respectively (see Figure SI.10). If we were to use the in situ gamma dose rate in the total dose rate calculation, the burial age would increase by 21% (from 75±6 ka to 91±7 ka) and the age would no longer be in stratigraphic order (the elevation of sample 167812 is 49.4 m). This supports our conclusion in the *Comparison with in situ dose rate measurements* section that the laboratory calculated dose rate is the more reliable, although it must be recognised that it is nevertheless important to correct for heterogeneity in the gamma field.

The quartz luminescence characteristics are satisfactory, although quartz multi-grain natural doses > 225 Gy are not considered to be accurate because of signal saturation issues. In practice, this implies that all samples from inside LRC I and from unit 5 in LRC IV are beyond the age range of multi-grain quartz dating. The main luminescence question remaining is whether the quartz was sufficiently bleached at the time of deposition to allow an accurate age estimate. This has been confirmed in two ways: (i) dose estimates based on quartz single-grain dose distributions are consistent with those from multi-grain aliquots, but only if those grains with a sufficiently large D_c are selected, and the average D_e is determined using the ADM or BayLum models and (ii) both IR_{50} and $pIRIR_{290}$ age estimates are consistently smaller than those based on multi-grain quartz OSL signals, with age ratios with respect to quartz of 0.482 ± 0.011 (n=34) for IR_{50} and 0.811 ± 0.018 (n=34) for $pIRIR_{290}$. The $pIRIR_{290}$ underestimate is surprising, and may be explained by either the use of an inappropriate internal dose rate or possibly by anomalous fading. But whichever explanation is correct, we in any case conclude that, for samples with a quartz D_e <225 Gy (i.e., those unlikely to be affected by saturation) the feldspar ages do not significantly overestimate the quartz ages. Taking these two results (single grain analyses and feldspar/quartz age ratios) together, we conclude that it is very likely that the quartz (and the feldspar) was well-bleached at the time of deposition.
Assuming that the quartz samples found inside the cave in LRC I and at the very bottom of LRC IV (unit 5) were also sufficiently bleached at deposition, quartz ages from these locations should be regarded as minimum ages, because these samples are at, or close to, saturation. Fortunately, the quartz ages from unit 5 in LRC IV and from inside LRC I (I.C. and E.C) are not important to the dating of the closure of the cave and can thus be safely omitted from the Baysian age depth model. This modelling gives a most likely closure age for the cave (LRC I) of 57 ± 3 ka (68% confidence level), and a minimum age for the closure of > 51 ka at the 95% confidence level. We thereby reject our hypothesis that the main cave and shelters were accessible for some time after the arrival of *Homo sapiens* in the region, around 37,000 years ago.
The model also gives an age of 97±5 ka (68% confidence level) for level 7 in LRC II, the location in unit 4, at which an artefact referred to as "The mask of LRC" [60] was found in 1979.

References

[1] Slimak, L. Le Néronien et la structure historique du basculement du Paléolithique moyen au Paléolithiqie supérieur en France méditerranéenne. *C.R. Palevol*, 6:301–309, 2007.

[2] Marquet, J. C., Macaire, J., Bayle, G., Peyouse, J., Guillaud, E., Aubry, T., Liard, M., Bréhéret, J., Thomsen, K. J., Freiesleben, T., Thamóné-Bozsó, E., Guérin, G., and Murray, A. S. Le site Prehistorique de la Roche-Cotard IV (Indre-et-loire, France): Une séquence du Pléistocene moyen et supérieur, référence pour le val de Loire Tourangeau. *Quaternaire*, 30(2):185–209, 2019.

[3] Marquet, J.-C., Lorblanchet, M., Oberlin, C., Thamo-Bozso, E., and Aubry, T. Nouvelle datation du « masque » de La Roche-Cotard (Langeais, Indre-et-Loire, France). *Paleo*, 27:253–263, 2016.

[4] Magaire, J.-J., Gay-Overero, I., Bacchi, M., Cocirta, C., Patryl, L., and Rodrigues, S. Petrography of alluvial sands as a past and present environmental indicator: Case of the Loire River (France). *International Journal of Sediment Research*, 28(3):285–303, 2013.

[5] Bøtter-Jensen, L., Thomsen, K. J., and Jain, M. Review of optically stimulated luminescence (OSL) instrumental developments for retrospective dosimetry. *Radiation Measurements*, 45:253–257, 2010.

[6] Bøtter-Jensen, L., Andersen, C. E., Duller, G. A. T., and Murray, A. S. Developments in radiation, stimulation and observation facilities in luminescence measurements. *Radiation Measurements*, 37:535–541, 2003.

[7] Huntley, D. J., Godfrey-Smith, D. I., and Haskell, E. H. Light induced emission spectra from some quartz and feldspars. *Nuclear Tracks and Radiation Measurements*, 18:127–131, 1991.

[8] Hansen, V., Murray, A. S., Buylaert, J.-P., Yeo, E. Y., and Thomsen, K. J. A new irradiated quartz for beta source calibration. *Radiation Measurements*, 81:123–127, 2015.

[9] Lapp, T., Jain, M., Thomsen, K. J., Murray, A. S., and Buylaert, J. P. New luminescence measurement facilities in retrospective dosimetry. *Radiation Measurements*, 47:803–808, 2012.

[10] Murray, A. S., Marten, R., Johnston, A., and Martin, P. Analysis for naturally occurring radionuclides at environmental concentrations by gamma spectrometry. *J. Radioanalytical Nucl. Chem.*, 115:263–288, 1987.

[11] Murray, A. S., Helsted, L. M., Autzen, M., Jain, M., and Buylaert, J.-P. Measurement of natural radioactivity: Calibration and performance of a high-resolution gamma spectrometry facility. *Radiation Measurements*, 120: 215–220, 2018. doi: 10.1016/j.radmeas.2018.04.006.

[12] Guérin, G., Mercier, N., and Adamiec, G. Dose-rate conversion factors: update. *Ancient TL*, 29:5–8, 2011.

[13] Prescott, J. R. and Hutton, J. T. Cosmic ray contributions to dose rates for luminescence and ESR dating: large depths and long-term variations. *Radiation Measurements*, 23:497–500, 1994.

[14] Guérin, G. and Mercier, N. Determining gamma dose rates by field gamma spectroscopy in sedimentary media: results of Monte Carlo simulations. *Radiation Measurements*, 46(2):190–195, 2011.

[15] Miallier, D., Guérin, G., Mercier, N., Pilleyre, T., and Sanzelle, S. The Clermont radiometric reference rocks: a convenient tool for dosimetric purposes. *Ancient TL*, 27(2):37–42, 2009.

[16] Kreutzer, S., Martin, L., Guérin, G., Tribolo, C., Selva, P., and Mercier, N. Environmental dose rate determination using a passive dosimeter: techniques and workflow for $\alpha - Al_2O_3$:C chips. *Geochronometria*, 45(1): 56–67, 2018.

[17] Kook, M. H., Lapp, T., Murray, A. S., and Thiel, C. Risø XRF attachment for major element analysis of aliquots of quartz and feldspar separates. In *UK Luminescence and ESR Meeting, Aberystwyth, September 2012*, page 37, 2012.

[18] Porat, N., Faerstein, G., Medialdea, A., and Murray, A. S. Re-examination of common extraction and purification methods of quartz and feldspar for luminescence dating. *Ancient TL*, 33:22–30, 2015.

[19] Murray, A. S. and Wintle, A. G. Luminescence dating of quartz using an improved single-aliquot regenerative-dose protocol. *Radiation Measurements*, 32:57–73, 2000.

[20] Duller, G. A. T. Distinguishing quartz and feldspar in single grain luminescence measurements. *Radiation Measurements*, 37:161–165, 2003.

[21] Combès, B., Lanos, P., Philippe, A., Mercier, N., Tribolo, C., Guérin, G., Guibert, P., and Lahaye, C. A Bayesian central equivalent dose model for optically stimulated luminescence dating. *Quaternary Geochronology*, 28:62–70, 2015.

[22] Duller, G. A. T. The Analyst software package for luminescence data: overview and recent improvements. *Ancient TL*, 33:35–42, 2015.

[23] Thomsen, K. J., Murray, A. S., and Bøtter-Jensen, L. Sources of variability in OSL dose measurements using single grains of quartz. *Radiation Measurements*, 39(1):47–61, 2005.

[24] Murray, A. S. and Wintle, A. G. The single aliquot regenerative dose protocol: potential for improvements in reliability. *Radiation Measurements*, 37:377–381, 2003.

[25] Ballarini, M., Wallinga, J., Wintle, A. G., and Bos, A. J. J. A modified SAR protocol for optical dating of individual grains from young quartz samples. *Radiation Measurements*, 42:360–369, 2007.

[26] Duller, G. A. T. Single-grain optical dating of Quaternary sediments: why aliquot size matters in luminescence dating. *Boreas*, 37:589–612, 2008.

[27] Medialdea, A., Thomsen, K. J., Murray, A. S., and Benito, G. Reliability of equivalent-dose determination and age-models in the OSL dating of historical and modern palaeoflood sediments. *Quaternary Geochronology*, 22: 11–24, 2014.

[28] Banerjee, D., Murray, A. S., Bøtter-Jensen, L., and Lang, A. Equivalent dose estimation using a single aliquot of polymineral fine grains. *Radiation Measurements*, 33:73–94, 2001.

[29] Galbraith, R. F., Roberts, R. G., Laslett, G. M., Yoshida, H., and Olley, J. M. Optical dating of single and multiple grains of quartz from Jinmium rock shelter, northern Australia: Part I, experimental design and statistical models. *Archaeometry*, 41:339–364, 1999.

[30] Guérin, G., Christophe, C., Philippe, A., Murray, A. S., Thomsen, K. J., Tribolo, C., Urbanova, P., Jain, M., Guibert, P., Mercier, N., Kreutzer, S., and Lahaye, C. Absorbed dose, equivalent dose, measured dose rates, and implications for OSL age estimates: Introducing the Average Dose Model. *Quaternary Geochronology*, 41: 163–173, 2017.

[31] Guérin, G., Combès, B., Tribolo, C., Lahaye, C., Mercier, N., Guibert, P., and Thomsen, K. J. Testing the accuracy of a single grain OSL Bayesian central dose model with known-age samples. *Radiation Measurements*, 81:62–70, 2015.

[32] Christophe, C., Philippe, A., Kreutzer, S., and Guerin, G. BayLum: Chronological Bayesian Models Integrating Optically Stimulated Luminescence and Radiocarbon Age Dating. R package version 0.1.3. https://CRAN.R-project.org/package=BayLum, 2018.

[33] Philippe, A., Guérin, G., and Kreutzer, S. BayLum - An R package for Bayesian analysis of OSL ages: An introduction. *Quaternary Geochronology*, 49:16–24, 2019. doi: 10.1016/j.quageo.2018.05.009.

[34] Buylaert, J.-P., Jain, M., Murray, A. S., Thomsen, K. J., Thiel, C., and Sohbati, R. A robust feldspar luminescence dating method for Middle and Late Pleistocene sediments. *Boreas*, 419:435–451, 2012.

[35] Vandenberghe, D., De Corte, F., Buylaert, J.-P., and Kučera, J. On the internal radioactivity in quartz. *Radiation Measurements*, 43(2-6):771–775, 2008.

[36] Zhao, H. and Li, S. Internal dose rate to K-feldspar grains from radioactive elements other than potassium. *Radiation Measurements*, 40(1):84–93, 2004.

[37] Huntley, D. J., Hancock, R., and Haskell, E. H. The Rb contents of the K-feldspar grains being measured in optical dating. *Ancient TL*, 19:43–46, 2001.

[38] Huntley, D. J. and Baril, M. R. The K content of the K-feldspars being measured in optical dating or in thermoluminescence dating. *Ancient TL*, 15:11–13, 1997.

[39] Riedesel, S. and Autzen, M. Beta and gamma dose rate attenuation in rocks and sediment. *Radiation Measurements*, 133(106295), 2020.

[40] Sohbati, R., Murray, A. S., Buylaert, J.-P., Almeida, N. A. C., and Cunha, P. P. Optically stimulated luminescence (OSL) dating of quartzite cobbles from the Tapada do Montinho archaeological site (east-central Portugal). *Boreas*, 41:452–462, 2012.

[41] Aitken, M. J. *Thermoluminescence Dating.* Academic Press, 1985. ISBN 0-12-046381-4.

[42] Murray, A. S., Arnold, L. J., Buylaert, J.-P., Guérin, G., Qin, J., Singhvi, A., Smedley, R., and Thomsen, K. Optically stimulated luminescence dating using quartz. *Nat Rev Methods Primers*, 1:72, 2021. doi: 10.1038/s43586-021-00068-5.

[43] Wintle, A. G. and Murray, A. S. A review of quartz optically stimulated luminescence characteristics and their relevance in single-aliquot regeneration dating protocols. *Radiation Measurements*, 41:369–391, 2006.

[44] Chapot, M. S., Roberts, H. M., Duller, G. A. T., and Lai, Z. P. A comparison of natural- and laboratory-generated dose response curves for quartz optically stimulated luminescence signals from Chinese Loess. *Radiation Measurements*, 47(11-12):1045–1052, 2012.

[45] Timar-Gabor, A. and Wintle, A. G. On natural and laboratory generated dose response curves for quartz of different grain sizes from Romanian loess. *Quaternary Geochronology*, 18:34–40, 2013.

[46] Singh, A., Thomsen, K. J., Sinha, R., Buylaert, J.-P., Carter, A., Mark, D. F., Mason, P. J., Densmore, A. L., Murray, A. S., Jain, M., Paul, D., and Gupta, S. Counter-intuitive influence of Himalayan river morphodynamics on Indus Civilisation urban settlements. *Nature Communications*, 8:1–14, 2017.

[47] Jacobs, Z., Duller, G. A. T., and Wintle, A. G. Optical dating of dune sand from Blombos Cave, South Africa: II - single grain data. *Journal of Human Evolution*, 44:613–625, 2003. doi: 10.1016/s0047-2484(03)00049-6.

[48] Thomsen, K. J., Murray, A. S., and Jain, M. The dose dependency of the overdispersion of quartz OSL single grain dose distributions. *Radiation Measurements*, 47:732–739, 2012.

[49] Thomsen, K. J., Murray, A. S., Buylaert, J.-P., Jain, M., Helt-Hansen, J., and Aubry, T. Testing single-grain quartz OSL methods using known age samples from the Bordes-Fitte rockshelter (Roches d'Abilly site, Central France). *Quaternary Geochronology*, 31:77–96, 2016.

[50] Heydari, M. and Guérin, G. OSL signal saturation and dose rate variability: investigating the behaviour of different statistical models. *Radiation Measurements*, 120:96–103, 2018.

[51] Arnold, L. J. and Roberts, R. G. Stochastic modelling of multi-grain equivalent dose (De) distributions: implications for OSL dating of sediment mixtures. *Quaternary Geochronology*, 4:204–230, 2009.

[52] Thomsen, K. J., Murray, A. S., Jain, M., and Bøtter-Jensen, L. Laboratory fading rates of various luminescence signals from feldspar-rich sediment extracts. *Radiation Measurements*, 43:1474–1486, 2008.

[53] Murray, A. S., Thomsen, K. J., Masuda, N., Buylaert, J.-P., and Jain, M. Identifying well-bleached quartz using the different bleaching rates of quartz and feldspar luminescence signals. *Radiation Measurements*, 47: 688–695, 2012.

[54] Thiel, C., Buylaert, J.-P., Murray, A. S., Terhorst, B., Hofer, I., Tsukamoto, S., and Frechen, M. Luminescence dating of the Stratzing loess profile (Austria)–Testing the potential of an elevated temperature post-IR IRSL protocol. *Quaternary International*, 234:23–31, 2011.

[55] Buylaert, J.-P., Murray, A. S., Gebhardt, A. C., Sohbati, R., Ohlendorf, C., Thiel, C., Wastegård, S., Zolitschka, B., and The PASADO Science Team. Luminescence dating of the PASADO core 5022-1D from Laguna Potrok Aike (Argentina) using IRSL signals from feldspar. *Quaternary Science Reviews*, 71:70–80, 2013.

[56] Wintle, A. G. Anomalous fading of thermoluminescence in mineral samples. *Nature*, 245:143–144, 1973.

[57] Huntley, D. J. and Lamothe, M. Ubiquity of anomalous fading in K-feldspars and the measurement and correction for it in optical dating. *Canadian Journal of Earth Sciences*, 38:1093–1106, 2001.

[58] Auclair, M., Lamothe, M., and Huot, S. Measurement of anomalous fading for feldspar IRSL using SAR. *Radiation Measurements*, 37:487–492, 2003.

[59] Blaauw, M. and Christen, J. A. Flexible paleoclimate age-depth models using an autoregressive gamma process. *Bayesian Anal.*, 6:457–474, 2011.

[60] Marquet, J. C. and Lorblanchet, M. A Neanderthal face? The proto-figurine from La Roche-Cotard, Langeais (Indre-et-Loire, France). *Antiquity*, 77(298), 2003.
