# Supplementary material for: The earliest unambiguous Neanderthal engravings on cave walls: La Roche-Cotard, Loire Valley, France
Source: PLoS One. 2023 Jun 21;18(6):e0286568. doi: 10.1371/journal.pone.0286568 (PMC10284424; doi:10.1371/journal.pone.0286568)

La Roche Cotard (+ experimentation) – Statistical Treatments (R Code)

#1. Préparation des données

```
# Importation des données (fichier 'LAPts.txt' sur le bureau avec séparateur "à la française")
don<-read.table("~/Desktop/LAPts.txt",header=T,sep = "\t",dec = ",")

#stat. descriptives pour les variables concernées
print(summary(don))

# Division en deux ensembles : donLRC=données LRC (4 panneaux) et donEXP=données expérimentation (pas de données pour sect = colonne 5)
donLRC <-don[1:116,]
donEXP <- don[c(-1:-116),-5]

library(foreign)

# Construction du tableau croisé type x sect (données LRC)
TC<-table(donLRC$type,donLRC$sect)
# Ajout des marges
addmargins(TC)

# Profils-lignes + eff totaux avec arrondis à 3 chiffres ap. virg
TClign=cbind(round(addmargins(prop.table(addmargins(TC,1),1),2),3),
c(margin.table(TC,1),sum(TC)))

# Insertion du nom des col de totalisation
colnames(TClign)<-c(colnames(TC),"sum_freq","EFFECTIF")
TClign

# Sélection des colonnes 1 à 4 (correspondant aux variables l, a, p et type) des fichiers donLRC et donEXP
don1 <-donLRC[,c(-5,-6)]
don1$type<-factor(don1$type)
don2 <- donEXP[,c(-5)]
don2$type<-factor(don2$type)

# Statistiques descriptives élémentaires (variables l, a et p)
aggregate(don1$l, by = list(type = don1$type), function(x) c(mean = mean(x), sd = sd(x)))
aggregate(don2$l, by = list(type = don2$type), function(x) c(mean = mean(x), sd = sd(x)))

aggregate(don1$a, by = list(type = don1$type), function(x) c(mean = mean(x), sd = sd(x)))
aggregate(don2$a, by = list(type = don2$type), function(x) c(mean = mean(x), sd = sd(x)))

aggregate(don1$p, by = list(type = don1$type), function(x) c(mean = mean(x), sd = sd(x)))
aggregate(don2$p, by = list(type = don2$type), function(x) c(mean = mean(x), sd = sd(x)))
```

#2. Approche univariée (histogrammes et boxplot par type et par variable)

#a. Avec don1 <Fig01>

```
# mfcol=c(i,p) avec i=nombre types et p=nombre variables utilisées (ici 4 types et 3 variables)
par(mfcol=c(4,3))
for (k in 1:3) {
j0 <- names(don1)[k]
br0 <- seq(min(don1[,k]),max(don1[,k]),le=11)
x0 <- seq(min(don1[,k]),max(don1[,k]),le=50)
for (i in 1:4) {
i0 <- levels(don1$type)[i]
x <- don1[don1$type==i0,j0]
hist(x,br=br0,proba=T,col=grey(0.8), main=i0,xlab=j0)
lines(x0,dnorm(x0,mean(x),sd(x)),col="red",lwd=2)
}
}
```

#b. Avec don2 <Fig02>

```
par(mar = c(4.5, 4, 2, 2))
par(mfcol=c(5,3))
for (k in 1:3) {
j0 <- names(don2)[k]
br0 <- seq(min(don2[,k]),max(don2[,k]),le=11)
x0 <- seq(min(don2[,k]),max(don2[,k]),le=50)
for (i in 1:5) {
i0 <- levels(don2$type)[i]
x <- don2[don2$type==i0,j0]
hist(x,br=br0,proba=T,col=grey(0.8), main=i0,xlab=j0)
lines(x0,dnorm(x0,mean(x),sd(x)),col="red",lwd=2)
}
}
```

#c. Détails

```
library(ggplot2)
library(easyGgplot2)

# Histogram l selon type <Fig03>
ggplot(donLRC, aes(x=l, color=type, fill=type)) +
geom_histogram(aes(y=..density..), alpha=0.5, position="identity") +
geom_density(alpha=.2) + scale_fill_manual(values=c("seagreen3", "lightcoral", "gold",
"cornflowerblue")) + scale_color_manual(values= c("seagreen3", "lightcoral", "gold",
"cornflowerblue"))
# Histogram a selon type <Fig04>
ggplot(donLRC, aes(x=a, color=type, fill=type)) +
geom_histogram(aes(y=..density..), alpha=0.5, position="identity") +
```

```
geom_density(alpha=.2) + scale_fill_manual(values=c("seagreen3", "lightcoral", "gold",
"cornflowerblue")) + scale_color_manual(values= c("seagreen3", "lightcoral", "gold",
"cornflowerblue"))
# Histogram p selon type <Fig05>
ggplot(donLRC, aes(x=p, color=type, fill=type)) +
geom_histogram(aes(y=..density..), alpha=0.5, position="identity") +
geom_density(alpha=.2) + scale_fill_manual(values=c("seagreen3", "lightcoral", "gold",
"cornflowerblue")) + scale_color_manual(values= c("seagreen3", "lightcoral", "gold",
"cornflowerblue"))

# Histogram l selon section <Fig06>
ggplot(donLRC, aes(x=l, color=sect, fill=sect)) +
geom_histogram(aes(y=..density..), alpha=0.5, position="identity") +
geom_density(alpha=.2) + scale_fill_manual(values= c("lightpink", "lightcoral",
"skyblue1","skyblue3")) + scale_color_manual(values=c("lightpink", "lightcoral", "skyblue1","
skyblue3"))
# Histogram a selon section <Fig07>
ggplot(donLRC, aes(x=a, color=sect, fill=sect)) +
geom_histogram(aes(y=..density..), alpha=0.5, position="identity") +
geom_density(alpha=.2) + scale_fill_manual(values= c("lightpink", "lightcoral",
"skyblue1","skyblue3")) + scale_color_manual(values=c("lightpink", "lightcoral", "skyblue1","
skyblue3"))
# Histogram a selon section <Fig08>
ggplot(donLRC, aes(x=p, color=sect, fill=sect)) +
geom_histogram(aes(y=..density..), alpha=0.5, position="identity") +
geom_density(alpha=.2) + scale_fill_manual(values= c("lightpink", "lightcoral",
"skyblue1","skyblue3")) + scale_color_manual(values=c("lightpink", "lightcoral", "skyblue1","
skyblue3"))

# Utilisation des "facets" pour diviser le graphique en plusieurs panneaux
histl<-ggplot(donLRC, aes(x=l))+ geom_density()+facet_grid(type ~ .)
plot(histl) #Fig
hista<-ggplot(donLRC, aes(x=a))+ geom_density()+facet_grid(type ~ .)
plot(hista) #Fig
histp<-ggplot(donLRC, aes(x=p))+ geom_density()+facet_grid(type ~ .)
plot(histp) #Fig
```

#d. Boxplots

```
gcbox<- c("darkorange3", "darkseagreen1", " lightpink", "green4", "goldenrod2",
"lightgoldenrod1", "lightblue1", "pink3", "palevioletred")
```

#1. Variable “l” <Fig09>

```
ggplot(don,aes(x=type,l,fill=type)) +
geom_boxplot(outlier.size=0.5,size=0.1,outlier.alpha=0.5) + scale_fill_manual(values= gcbox)
+ scale_color_manual(values= gcbox)
```

#2. Variable “a” <Fig10>

```
ggplot(don,aes(x=type,a,fill=type)) +
geom_boxplot(outlier.size=0.5,size=0.1,outlier.alpha=0.5) + scale_fill_manual(values= gcbox)
+ scale_color_manual(values= gcbox)
```

#3. Variable “p” <Fig11>

```
ggplot(don,aes(x=type,p,fill=type)) +
geom_boxplot(outlier.size=0.5,size=0.1,outlier.alpha=0.5) + scale_fill_manual(values= gcbox)
+ scale_color_manual(values= gcbox)
```

Utilisation des “facets” pour diviser le graphique en plusieurs panneaux

#1. Partition de la fenêtre en 3 panneaux (pour l, a et p) avec don1 = données LRC (donc 4 types)

```
library("cowplot")

bpl <- ggplot(don1, aes(x=type, y=l, fill=type)) + geom_boxplot() +
scale_fill_manual(values=c("seagreen3", "lightcoral", "gold", "cornflowerblue")) +
theme(legend.position = "none")
bpa <- ggplot(don1, aes(x=type, y=a, fill=type)) + geom_boxplot() +
scale_fill_manual(values=c("seagreen3", "lightcoral", "gold", "cornflowerblue")) +
theme(legend.position = "none")
bpp <- ggplot(don1, aes(x=type, y=p, fill=type)) + geom_boxplot() +
scale_fill_manual(values=c("seagreen3", "lightcoral", "gold", "cornflowerblue")) +
theme(legend.position = "none")

#<Fig12>
plot_grid(bpl, bpa, bpp, labels=c("largeur", "angle", "profondeur"), ncol = 3, nrow = 1)
```

#2. Partition de la fenêtre en 3 panneaux (pour l, a et p) avec don2 = données EXP (donc 5 types)

```
bpl2 <- ggplot(don2, aes(x=type, y=l, fill=type)) + geom_boxplot() +
scale_fill_manual(values=c("darkorange3", "olivedrab", "goldenrod2", "pink3",
"palevioletred")) +
theme(legend.position = "none")
bpa2 <- ggplot(don2, aes(x=type, y=a, fill=type)) + geom_boxplot() +
scale_fill_manual(values=c("darkorange3", "olivedrab", "goldenrod2", "pink3",
"palevioletred")) +
theme(legend.position = "none")
bpp2 <- ggplot(don2, aes(x=type, y=p, fill=type)) + geom_boxplot() +
scale_fill_manual(values=c("darkorange3", "olivedrab", "goldenrod2", "pink3",
"palevioletred")) +
theme(legend.position = "none")

#<Fig13>
plot_grid(bpl2, bpa2, bpp2, labels=c("largeur", "angle", "profondeur"), ncol = 3, nrow = 1)
```

#3. Approche bivariée

#a. Analyses de variance à un facteur variable par variable

#1. Variable "l"

```
# Moyennes et écarts-types par groupe
tapply(don1$l,don1$type,mean)
tapply(don1$l,don1$type,sd)
# Analyse de la variance à un facteur
options(show.signif.stars=FALSE)
anova(lm(don1$l~don1$type))
```

#2. Variable "a"

```
# Moyennes et écarts-types par groupe
tapply(don1$a,don1$type,mean)
tapply(don1$a,don1$type,sd)
# Analyse de la variance à un facteur
options(show.signif.stars=FALSE)
anova(lm(don1$a~don1$type))
```

#3. Variable "p"

```
# Moyennes et écarts-types par groupe
tapply(don1$p,don1$type,mean)
tapply(don1$p,don1$type,sd)
# Analyse de la variance à un facteur
options(show.signif.stars=FALSE)
anova(lm(don1$p~don1$type))
```

#b. Représentation de tous les nuages bivariés

```
library(ade4)
library(adegraphics)
```

#1. Avec don1 <Fig14>

```
s.class(don1[,-4], don1$type, xax=1:3, yax=1:3, porigin.include=FALSE,
plabels.cex=1.2, col=c("seagreen3", "lightcoral", "gold", "cornflowerblue"), ppoints.cex=1,
starSize=0.8)
```

#2. Avec don2 <Fig15>

```
s.class(don2[,-4], don2$type, xax=1:3, yax=1:3, porigin.include=FALSE,
plabels.cex=1.2, col=c("darkorange3", "olivedrab", "goldenrod2", "pink3",  "palevioletred"),
ppoints.cex=1, starSize=0.8)
```

```
#4. Approche en dimension 3
library(scatterplot3d)

# Réservation de tout l'espace de la zone « plots » pour les graphiques
par(mfrow=c(1,1))

mar0=c(3,3,2,2) #définition des marges

#a. Source the function
source('http://www.sthda.com/sthda/RDoc/functions/addgrids3d.r')
#b. 3D scatter plot
gc <- c("seagreen3", "lightcoral", "gold", "cornflowerblue")
scatterplot3d(don1[,1],don1[,2],don1[,3],mar=mar0,color=gc[don1$type],
pch=c(16,4,15,17)[don1$type], main="3D Scatter Plot", xlab = "l",  ylab = "a", zlab = "p")
legend("topright", legend = levels(don1$type), pch = c(16, 4, 15, 17), col =  gc)
#c. Add grids <Fig16>
addgrids3d(don1[, 1:3], grid = c("xy", "xz", "yz"))
```

#5a. AFD sur don1 (*ie* sur les variables l, a et p ; discrim sur la variable ‘type’) avec la library “ade4” => **pratique à la française => maximisation du rapport inter/totale**

#a. Préparation des structures (avec écriture en Y et X)

```
# X
X <- don1[,-4] #exclusion de la variable type (qualitative)
# Y
don1$type<-factor(don1$type)
Y <- don1$type
# Fréquences relatives des classes
print(prop.table(table(Y)))

# Nombre de variables
p <- ncol(X)
# Nombre d'observations
n <- nrow(X)
# Nombre de classes
K <- nlevels(Y)

# Avec définitions ci-dessus de X et Y (pour fichier LAPts.txt)
afdLAP1 <- discrimin(dudi.pca(X, scan = F), Y, scan = F)

# Explicitation des résultats de l'analyse discrim
afdLAP1

# Calcul de la “proportion of trace”
trLAP1 <- sum(afdLAP1$eig)
afdLAP1$eig[[1]]/trLAP1
afdLAP1$eig[[2]]/trLAP1

# Graphiques standards de l'analyse discrim (avec ade4) <Fig17>
plot(afdLAP1)

# Galerie des nuages <Fig18>
pairs(don1[,-4], main = " LRC - galerie des nuages", cex.main = 0.8, pch = c(21, 4, 22, 24)[don1$type], bg = c("seagreen3", "lightcoral", "gold", "cornflowerblue")[don1$type], las = 1, gap = 0, labels = c("largeur", "angle", "profondeur"))
legend("topright", legend = levels(don1$type), xpd = TRUE, horiz = TRUE, inset = c(0,0), bty = "n", col = c("seagreen3", "lightcoral", "gold", "cornflowerblue"), pch = c(16, 4, 15, 17), cex = 0.8)
```

#b. Figure de référence de l’AFD

```
# Utilisation de la library adegraphics pour représenter la figure de référence de l'AFD (effectuée sur les 4 variables l, a, p et 'type' du fichier LAPts.txt où 'type' permet de différencier les 4 panneaux (TRI, REC, CLA, CIR))
```

```
gcLAP1<- c("seagreen3", "lightcoral", "gold", "cornflowerblue") #affectation des couleurs par panneau=type

# Graphique avec échelles + titres pour les axes <Fig19>
#mar0=c(4,2,2,2) <HS>
g1a <- s.class(afdLAP1$li, fac = don1$type, col=gcLAP1, pellipses.alpha=0.5, pellipses.lwd=2, pellipses.border=gcLAP1, pellipses.axes.col=gcLAP1, ppoints.cex=0.6, plabels.cex = 1, xlim= c(-2.5,3), ylim=c(-2,4), plines.lwd=0.5, paxes.draw=TRUE, xlab = "LD1 (59.5%)",  ylab = "LD2 (35.3%)")
```

#5b. AFD sur don3 (*ie* sur les variables l, a et p mais pour l’ensemble des données – dont les données expérimentales – avec discrim sur la variable quali = ‘type’)

```
# Fichier LAPts.txt > sélection de toutes les lignes (correspondant aux 4 panneaux + expérimentation)
don3 <-don[,c(-5,-6)]
don3$type<-factor(don3$type)

afdLAP3 <- discrimin(dudi.pca(don3[,-4], scan = F), don3$type, scan = F)
afdLAP3
trLAP3 <- sum(afdLAP3$eig)
afdLAP3$eig[[1]]/trLAP3
afdLAP3$eig[[2]]/trLAP3

# <Fig20>
plot(afdLAP3)

# Coloriage des ellipses (au nombre de 8) et demande séparée du plot
# Affectation des couleurs par type (les couleurs correspondent à l’ordre alpha des types (version english)
gcLAP3<- c("darkorange3", "darkseagreen1", " lightpink", "green4", "goldenrod2", "lightgoldenrod1", "lightblue1", "pink3", "palevioletred") #gc pour ‘colors group’

# <Fig21>
g3a <- s.class(afdLAP3$li, fac = don3$type, col=gcLAP3, pellipses.alpha=0.5, pellipses.lwd=2, pellipses.border=gcLAP3, pellipses.axes.col=gcLAP3, ppoints.cex=0.6, plabels.cex = 1, xlim= c(-2,3.5), ylim=c(-4.5,2.5), plines.lwd=0.5, paxes.draw=TRUE, xlab = "LD1 (49.5%)",  ylab = "LD2 (38.3%)")
```

#6. ACP sur don1 (*ie* sur l, a, p et ‘type’ où ‘type’ permet de différencier les 4 panneaux (TRI, REC, CLA, CIR) (exclusion de la variable quali = ‘sect’ dans don1)

```
library(factoextra)

pcaLAP1 <- dudi.pca(don1[,c(-4,-5)], scan = F)

pcaLAP1 #explicite les résultats de l’analyse en composantes principales

# Valeurs propres
eig.val <- get_eigenvalue(pcaLAP1)
eig.val

# Figure de référence de l’ACP
# Affectation des couleurs par panneau=type
gc_pcaLAP1<- c("seagreen3", "lightcoral", "gold", "cornflowerblue")

# Graphique avec échelles + titres pour les axes <Fig22>
g1pca <- s.class(pcaLAP1$li, fac = don1$type, col= gc_pcaLAP1, pellipses.alpha=0.5,
pellipses.lwd=2, pellipses.border= gc_pcaLAP1, pellipses.axes.col= gc_pcaLAP1,
ppoints.cex=0.6, plabels.cex = 1, xlim= c(-4,3.5), ylim=c(-2.5,4), plines.lwd=0.5,
paxes.draw=TRUE, xlab = "CP1 (53.4%)",  ylab = "CP2 (42.4%)")
```

#7. AFD sur don1 (*ie* sur les variables l, a et p ; discrim sur la variable ‘type’) avec la library “MASS” => **pratique à anglaise => maximisation du rapport inter/intra**

```
library(MASS)
```

#a. Appel de la fonction lda (linear discriminant analysis) de la library MASS

```
don1.lda <-lda(type~l+a+p,data=don1)
# Détail des principaux résultats
don1.lda
```

#b. AFD décomposition des phases essentielles des calculs d’une AFD

```
# Nombre d’observations N (compte les lignes de la matrice en entrée ; ici n=111)
N<-nrow(don1)
N

# Matrice totale à N-1 degrés de liberté
T<- (N-1)*cov(don1[,1:3])
T

# Coordonnées des barycentres des groupes (ici 4 barycentres car 4 groupes)
M<-don1.lda$means
M

# Construction de la matrice de "design" (qui consiste ici à numéroter les 4 types)
X_k <- NULL; for(i in 1:4) X_k <- cbind(X_k, as.numeric(don1$type == levels(don1$type)[i]))
#[,1]=CIR ; #[,2]=CLA ; #[,3]=REC ; #[,4]=TRI (inverse ordre des lignes : TRI, REC, CLA, CIR)

# Construction de la matrice G
G<-X_k %*% M

# Matrice intraclasse ou residuelle (=Within) W
W <- t(as.matrix(don1[,1:3])-G) %*% (as.matrix(don1[,1:3])-G)
W

# Matrice inter classes (=Between) B
B<- T-W
B

# Matrice de covariance intra-classes
Wcov<-W/(N-ncol(X_k))
# Matrice de covariance inter-classes
Bcov<-B/(ncol(X_k)-1)
```

#c. Pratique anglaise : maximisation du rapport inter/intra

```
eigen(solve(Wcov)%*%Bcov)
```

```
# Valeurs de mu1 et mu2
mu=eigen(solve(Wcov)%*%Bcov)$values
mu

# Relation entre mu et lambda
lambda=mu/(1+mu)
lambda

#d. Graphiques importants de l'AFD
V <- eigen(solve(Wcov)%*%Bcov )$vectors[,1:2]
V

# Matrice de normalisation V^TWV = 1
echelle<- sqrt(diag(diag((t(V) %*% Wcov %*% V))))
echelle

#LD1 & LD2 de lda (au signe près)
LD <- V %*% solve(echelle)
LD

# Données centrées
X<- scale(don1[,1:3],scale=FALSE)

# Coordonnées des 107 ind sur les axes discriminants
LD12<- X%*%LD

# Coordonnées des barycentres sur LD1 et LD2
MLD1<- tapply((X%*%LD)[,1],don1$type,mean)
MLD2<- tapply((X%*%LD)[,2],don1$type,mean)
# Graphique du premier plan discriminant (LD1-LD2) <Fig23>
plot(LD12[,1],LD12[,2],xlab="LD1",ylab="LD2",type="n")
mtext(outer=T, "Discrimination des marques (4 types)", side=3, line=-2, cex=1.2)
points(MLD1[1],MLD2[1], pch=19, col="seagreen3", bg="seagreen3", cex=2, lwd=2)
points(MLD1[2],MLD2[2], pch=18, col="lightcoral", bg="lightcoral", cex=2, lwd=2)
points(MLD1[3],MLD2[3], pch=15, col="gold", bg=" gold", cex=2, lwd=2)
points(MLD1[4],MLD2[4], pch=17, col="cornflowerblue", bg="cornflowerblue", cex=2,
lwd=2)
text(LD12[,1],LD12[,2], label=as.character(don1$type),cex=0.5)

#e. Liens entre AFD avec ACC (=Analyse des Correspondances canoniques)
# Vérification : les racines carrées des vp de W^-1B sont égales aux coefficients de corrélation
canonique du couple X, X_K)
cancor(X,X_k)$cor
sqrt(eigen(solve(W)%*%B)$values[1:2]/(1+eigen(solve(W)%*%B)$values[1:2]))
```

#f. Distances de Mahalanobis entre les 111 ind et les barycentres des 4 classes

```
# Première et deuxième coordonnées des barycentres
MLD1<- tapply((X%*%LD)[,1], don1$type,mean)
MLD2<- tapply((X%*%LD)[,2], don1$type,mean)
MLD12<- cbind(MLD1,MLD2)
# Matrice des distances observations * barycentres
round(dist(rbind(X%*%LD,MLD12)),1)
```

#g. Tableau des « resubstitutions » dans les 4 classes

```
pred <- predict(don1.lda)$class
# Matrice de confusion (confrontation avec les classes observées en test)
mc <- table(don1$type, pred)
mc
# Taux de reconnaissance (accuracy)
acc <- sum(diag(mc))/sum(mc)
acc

# Calcul du taux d'erreur
tx_err <- 1 - sum(diag(mc)) / sum(mc)
tx_err

# Sensibilité par classe
sensTest <- diag(mc)/rowSums(mc)
sensTest
```

#h. Validation croisée (cross-validation) par Jackknife du classement dans les 4 classes (LRC)

```
# Jackknife : on refait le calcul n=111 fois en omettant une à une les données et on reclasse successivement chaque donnée omise
don1.lda.jack <-lda(type~.,data=don1, CV=TRUE)
tabl <- table(don1$type, don1.lda.jack$class)
tabl

#nombre et taux d’erreur
errj <- sum(tabl) - sum(diag(tabl))
errj
tx_errj <- 1 - sum(diag(tabl)) / sum(tabl)
tx_errj
binom.test(errj,sum(mc))
```

#i. Discrimination sur une seule variable discriminante (anova)

```
# Valeurs de LD1 sur l’échantillon pour les n=107 marques
ld1 <- predict(don1.lda)$x[,1]
anova(lm(ld1 ~ don1$type))
# Valeurs de LD2 sur l’échantillon pour les n=107 marques
ld2 <- predict(don1.lda)$x[,2]
anova(lm(ld2 ~ don1$type))
```

#8. Analyse factorielle des données mixtes (AFDM) sur donLRC (*ie* sur les variables l, a, p, type et sect) avec la library "FactoMineR"

```
library(ggplot2)
library(easyGgplot2)
library(FactoMineR)
library(factoextra)

# Utilisation de la variable "id" pour les noms des lignes via rows.names (assignation comme nom d'une variable d'identifiant en lieu et place des numéros d'ordre)
row.names(donLRC) <- donLRC$id

# Analyse factorielle des données mixtes en utilisant la fonction 'FAMD' de "FactoMineR"
res.famd <- FAMD(donLRC[,-6], graph = FALSE)
print(res.famd) #affichage des résultats (sous forme d'une liste)
```

#a. Valeurs propres / Variances

```
# Proportion de variances expliquées par les différentes dimensions (axes)
eig.val <- get_eigenvalue(res.famd)
head(eig.val)

# Visualisation des proportions de variances expliquées par les différents axes
fviz_screeplot(res.famd) #scree plot = ébouli des vp
```

#b. Graphique des variables

```
var <- get_famd_var (res.famd)
var

# Coordonnées des variables
head(var$coord)
# Cos2 (qualité de la représentation)
head(var$cos2)
# Contributions aux dimensions
head(var$contrib)
```

#1. Toutes les variables

```
fviz_famd_var (res.famd, repel = TRUE) #graph variables-FAMD <Fig>
# Contribution à la première dimension
fviz_contrib (res.famd, "var", axes = 1) #graph contribution of var to dim1
# Contribution à la deuxième dimension
fviz_contrib (res.famd, "var", axes = 2) #graph contribution of var to dim2
```

#2. Variables quanti

```
quanti.var <- get_famd_var(res.famd, "quanti.var")
quanti.var
```

```
# Cercle des corrélations variables quanti (FAMD)
fviz_famd_var(res.famd, "quanti.var", repel = TRUE, col.var = "black")

# Variante1 : gradient de couleurs selon la contribution aux axes <Fig>
fviz_famd_var(res.famd, "quanti.var", col.var = "contrib",
        gradient.cols = c("#00AFBB", "#E7B800", "#FC4E07"),
        repel = TRUE)

# Variante2 : gradient de couleur par valeurs cos2 (qualité sur le plan des facteurs) <Fig>
fviz_famd_var(res.famd, "quanti.var", col.var = "cos2",
        gradient.cols = c("#00AFBB", "#E7B800", "#FC4E07"),
        repel = TRUE)


#3. Variables quali
quali.var <- get_famd_var(res.famd, "quali.var")
quali.var

# Graph “qualitative variable categories” (FAMD) : gradient de couleurs selon la contribution
<Fig24>
fviz_famd_var(res.famd, "quali.var", col.var = "contrib",
        gradient.cols = c("#00AFBB", "#E7B800", "#FC4E07")
        )

#c. Graphique des individus
ind <- get_famd_ind(res.famd)
ind

# Étiquetage des individus par numéro d’ordre
# Variante1 : coloriage des individus en fonction de leur contribution <Fig>
fviz_famd_ind(res.famd, col.ind = "contrib",
        gradient.cols = c("#00AFBB", "#E7B800", "#FC4E07"),
        repel = TRUE)
# Variante2 : coloriage des individus par valeurs cos2 (qualité sur le plan des facteurs) <Fig>
fviz_famd_ind(res.famd, col.ind = "cos2",
        gradient.cols = c("#00AFBB", "#E7B800", "#FC4E07"),
        repel = TRUE)

#d. Graphique de référence : coloriage des individus (=marques) en utilisant la variable “type”
du tableau de données initial
gc <- c("seagreen3", "lightcoral", "gold", "cornflowerblue") #palette couleur

# Avec ellipses à 95%
gfamd <- fviz_mfa_ind(res.famd,
        axes = c(1, 2),
        geom = c("point", "text"), #si sans étiquette : geom = c("point"),
        label = "all",
```

```
invisible = "none",
        labelsize = 2,
        pointsize = 1,
        habillage = "type", #color by groups
        palette = gc,
        addEllipses = TRUE, ellipse.level = 0.95,
        ellipse.type = "norm", ellipse.alpha = 0.1,
        #col.ind = "blue",
        #col.ind.sup = "darkblue",
        alpha.ind = 1,
        shape.ind = 19,
        #col.var, alpha.var, shape.var, col.quali.var,
        repel = TRUE, #repel = repulsive textual annotations (optimisation étiquetage points)
        axes.linetype = "dashed",
        #select.ind = list(name = NULL, cos2 = NULL, contrib = NULL), title = "bip"
        )
gfamd
```

Graphics

Fig01. Histogrammes (LRC) : p. 17
Fig02. Histogrammes (EXP) : p. 18
Fig03. Densité variable l par type (LRC) : p. 19
Fig04. Densité variable a par type (LRC) : p. 20
Fig05. Densité variable p par type (LRC) : p. 21
Fig06. Densité variable l par section (LRC) : p. 22
Fig07. Densité variable a par section (LRC) : p. 23
Fig08. Densité variable p par section (LRC) : p. 24
Fig09. Boxplot variable l (LRC+EXP) : p. 25
Fig10. Boxplot variable a (LRC+EXP) : p. 26
Fig11. Boxplot variable p (LRC+EXP) : p. 27
Fig12. Boxplot variables l-a-p (LRC) : p. 28
Fig13. Boxplot variables l-a-p (EXP) : p. 29
Fig14. Nuages bivariés (LRC) : p. 30
Fig15. Nuages bivariés (EXP) : p. 31
Fig16. 3D Scatter Plot (LRC) : p. 32
Fig17. Graphiques ade4 AFD (LRC) : p. 33
Fig18. Galerie des nuages (LRC) : p. 34
Fig19. AFD (LRC) : p. 35
Fig20. Graphiques ade4 AFD (LRC+EXP) : p. 36
Fig21. AFD (LRC+EXP) : p. 37
Fig22. ACP (LRC) : p. 38
Fig23. AnalyseFactorielleDonnéesMixtes1 (LRC) : p. 39
Fig24. AnalyseFactorielleDonnéesMixtes2 (LRC) : p. 40

Nota :
LRC = only the data from the cave
EXP = only the data from the experiment
LRC+EXP = all data

AFD = Discriminant factor analysis (LDA in english)
ACP = Principal component analysis (PCA in english)
AFDM = Factorial Analysis of Mixed Data (FAMD in english)

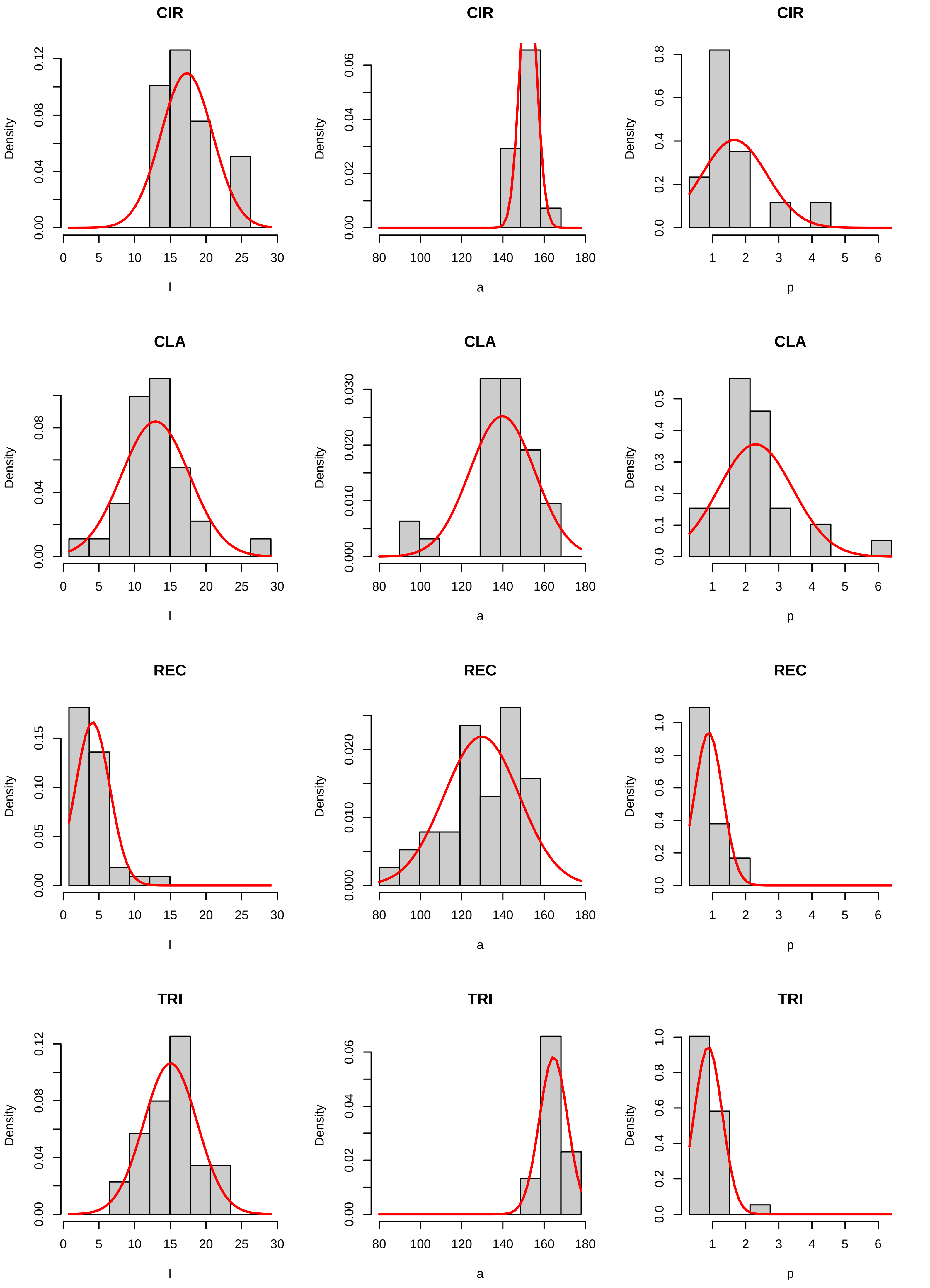

CIR
CIR
CIR
CLA
CLA
CLA
REC
REC
REC
TRI
TRI
TRI
Density
l
a
p
0 5 10 15 20 25 30
80 100 120 140 160 180
1 2 3 4 5 6
0.00 0.04 0.08 0.12
0.00 0.02 0.04 0.06
0.0 0.2 0.4 0.6 0.8
0.000 0.010 0.020 0.030
0.0 0.1 0.2 0.3 0.4 0.5
0.00 0.05 0.10 0.15
0.000 0.010 0.020
0.0 0.2 0.4 0.6 0.8 1.0

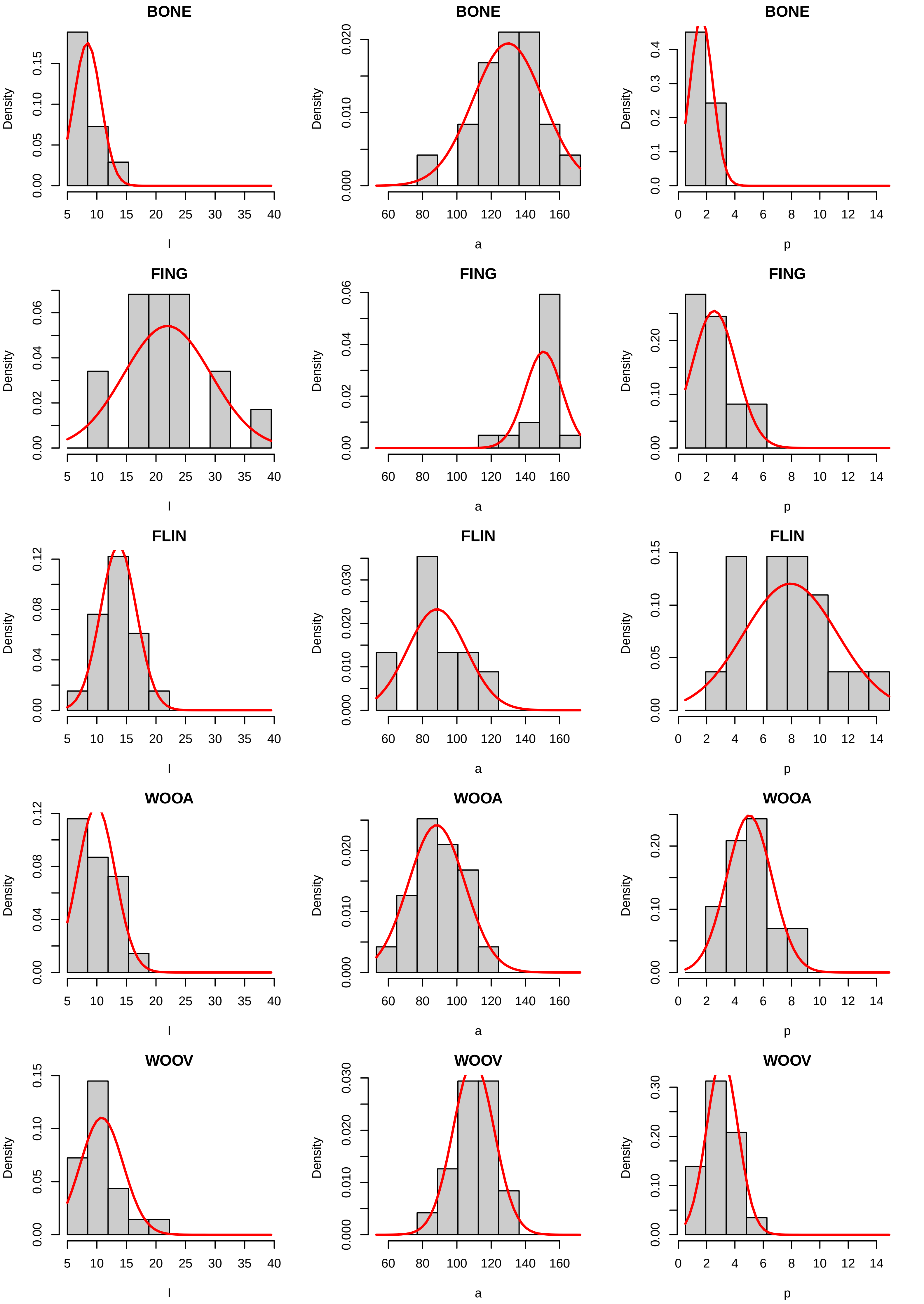
BONE
Density
l
BONE
Density
a
BONE
Density
p
FING
Density
l
FING
Density
a
FING
Density
p
FLIN
Density
l
FLIN
Density
a
FLIN
Density
p
WOOA
Density
l
WOOA
Density
a
WOOA
Density
p
WOOV
Density
l
WOOV
Density
a
WOOV
Density
p

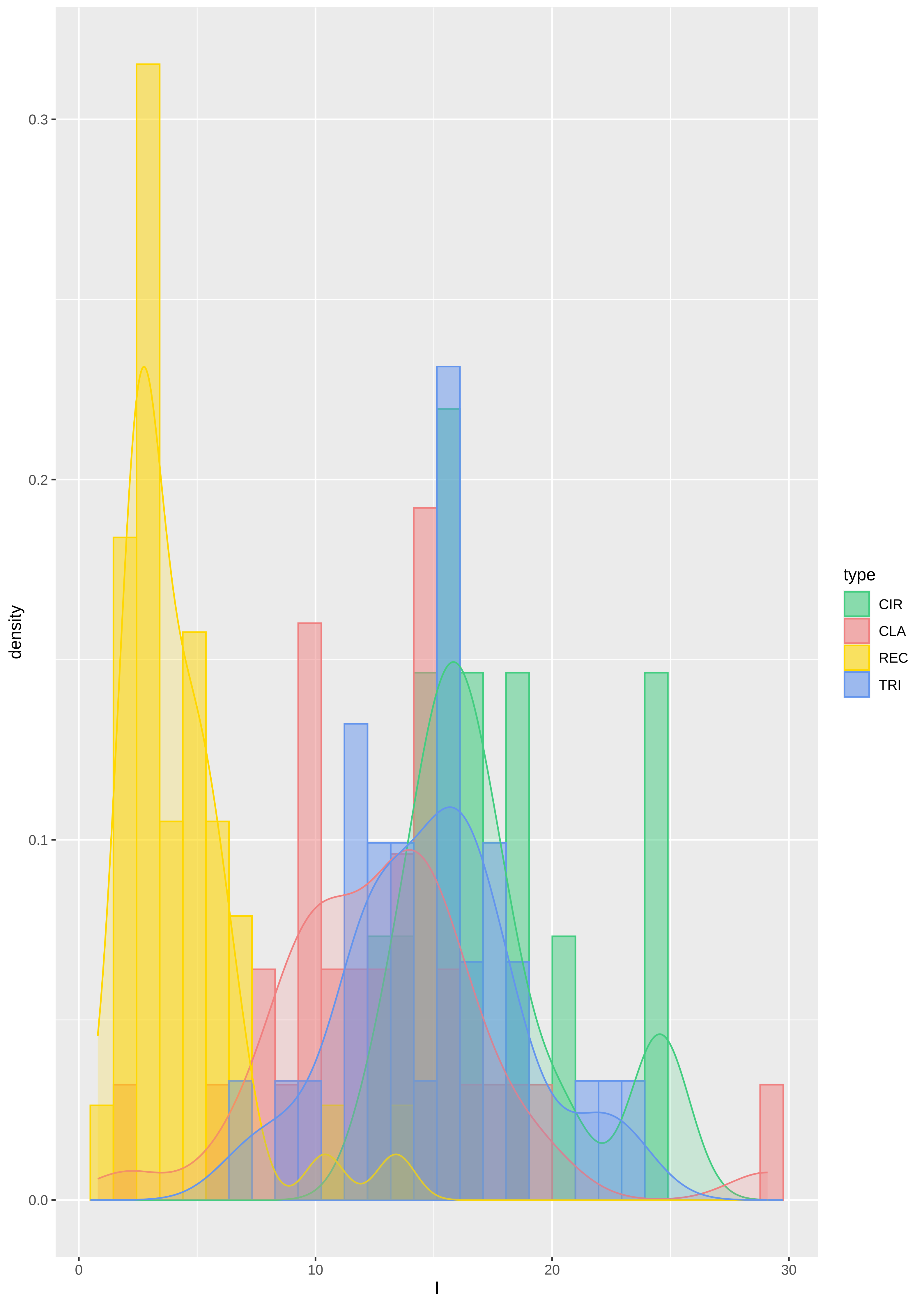

0.3
0.2
0.1
0.0
density
0
10
20
30
l
type
CIR
CLA
REC
TRI

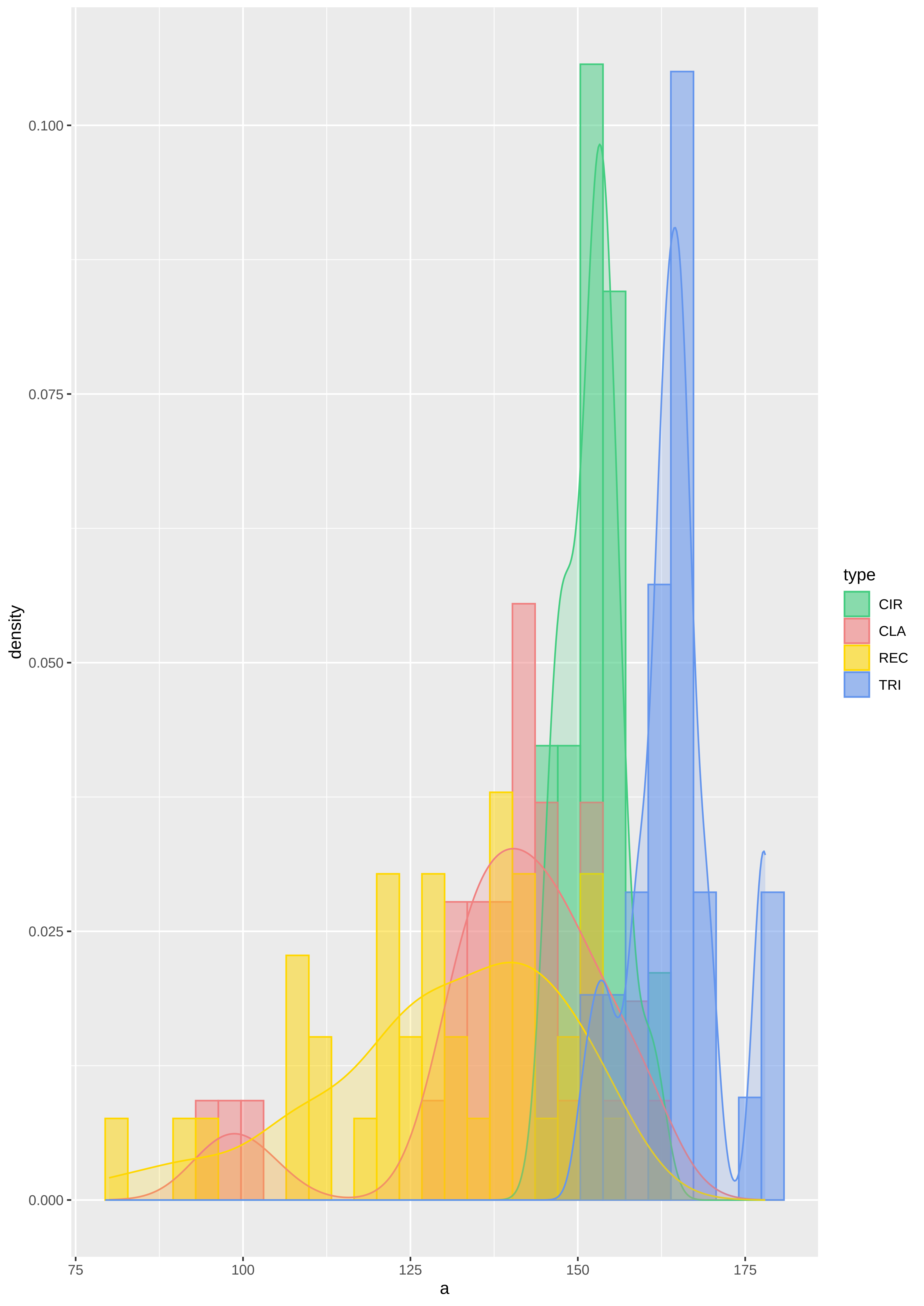
density
0.100
0.075
0.050
0.025
0.000
75
100
125
150
175
a
type
CIR
CLA
REC
TRI

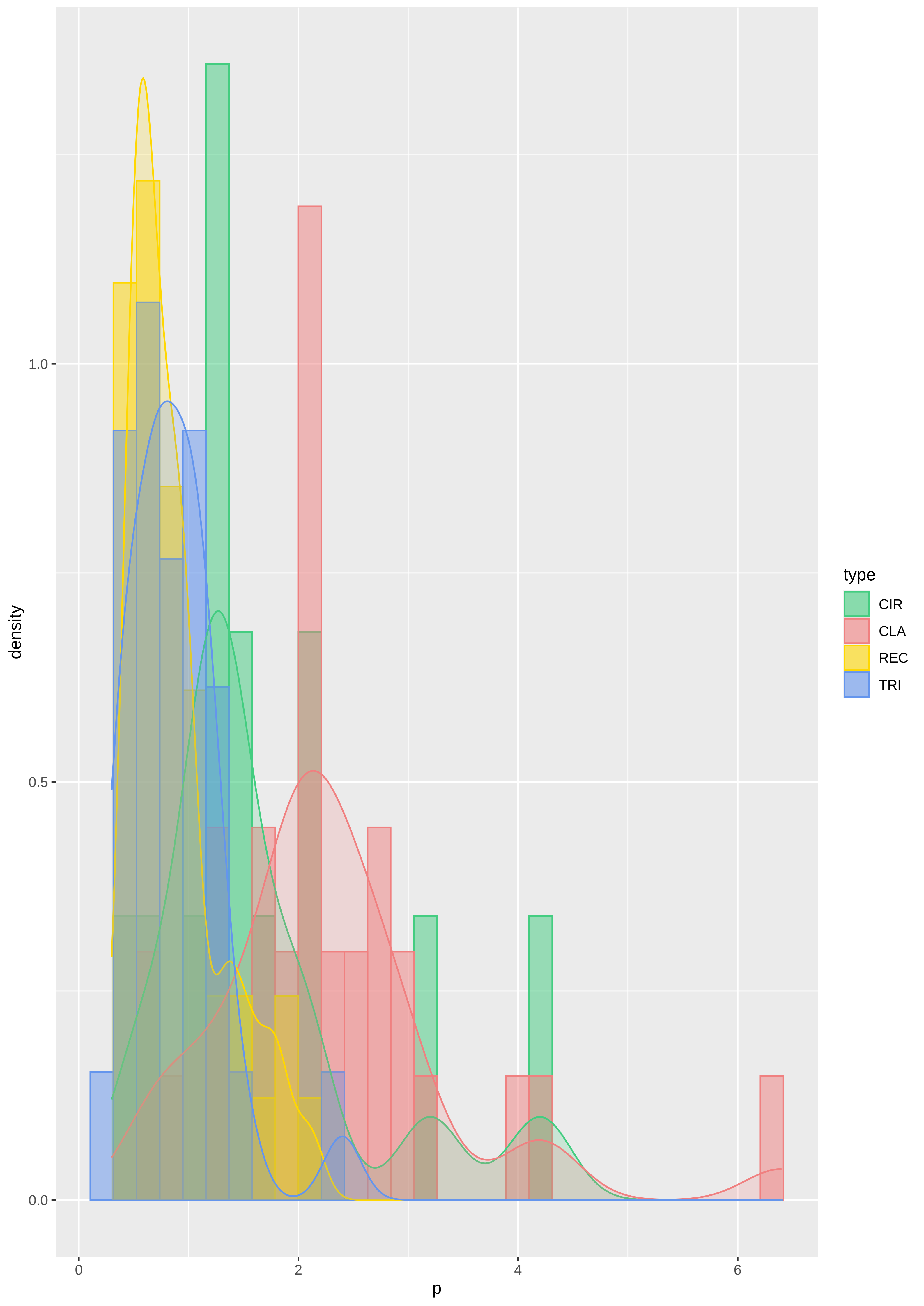

density
0.0
0.5
1.0
0
2
4
6
p
type
CIR
CLA
REC
TRI

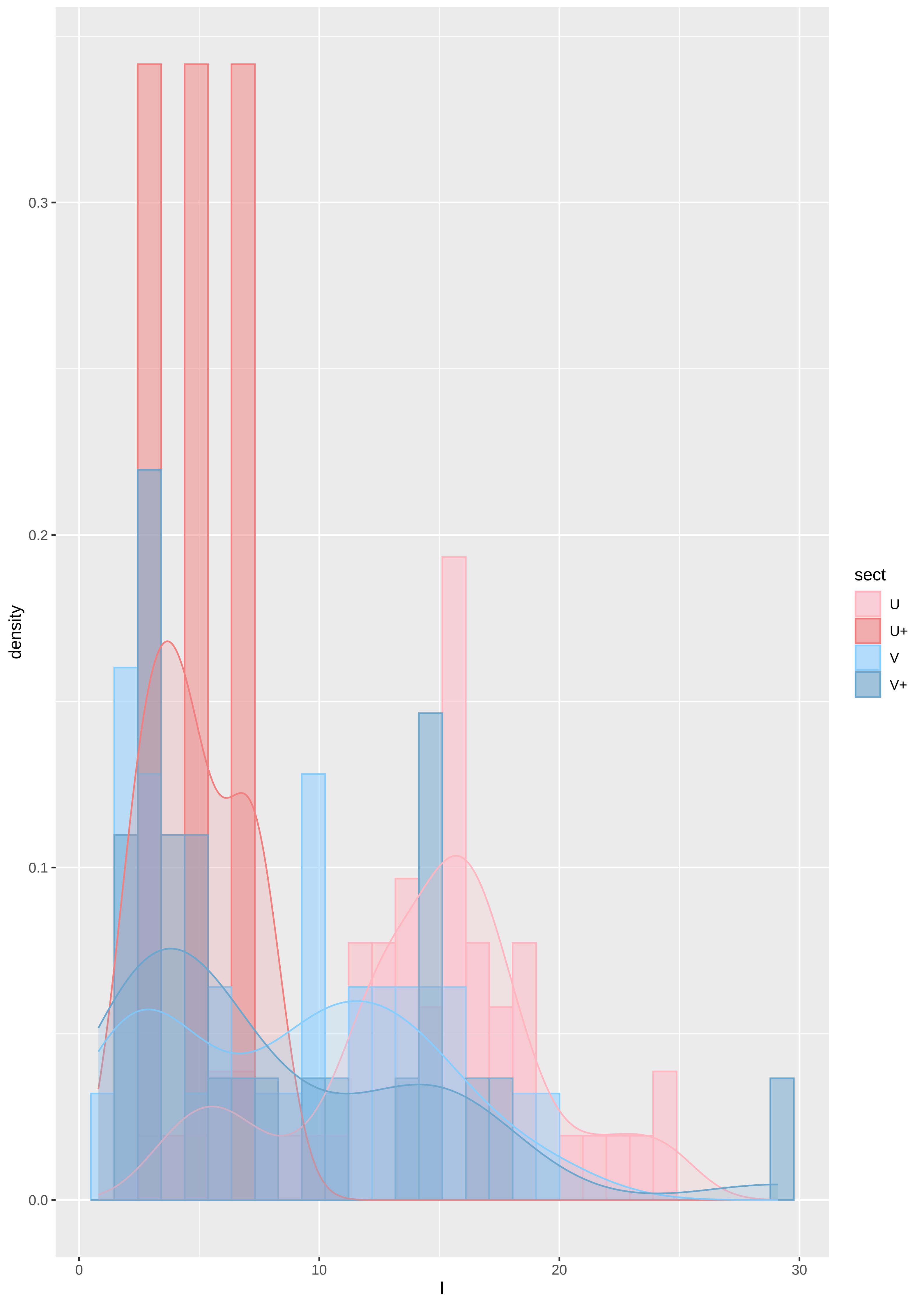

0.3
0.2
0.1
0.0
density
0
10
20
30
l
sect
U
U+
V
V+

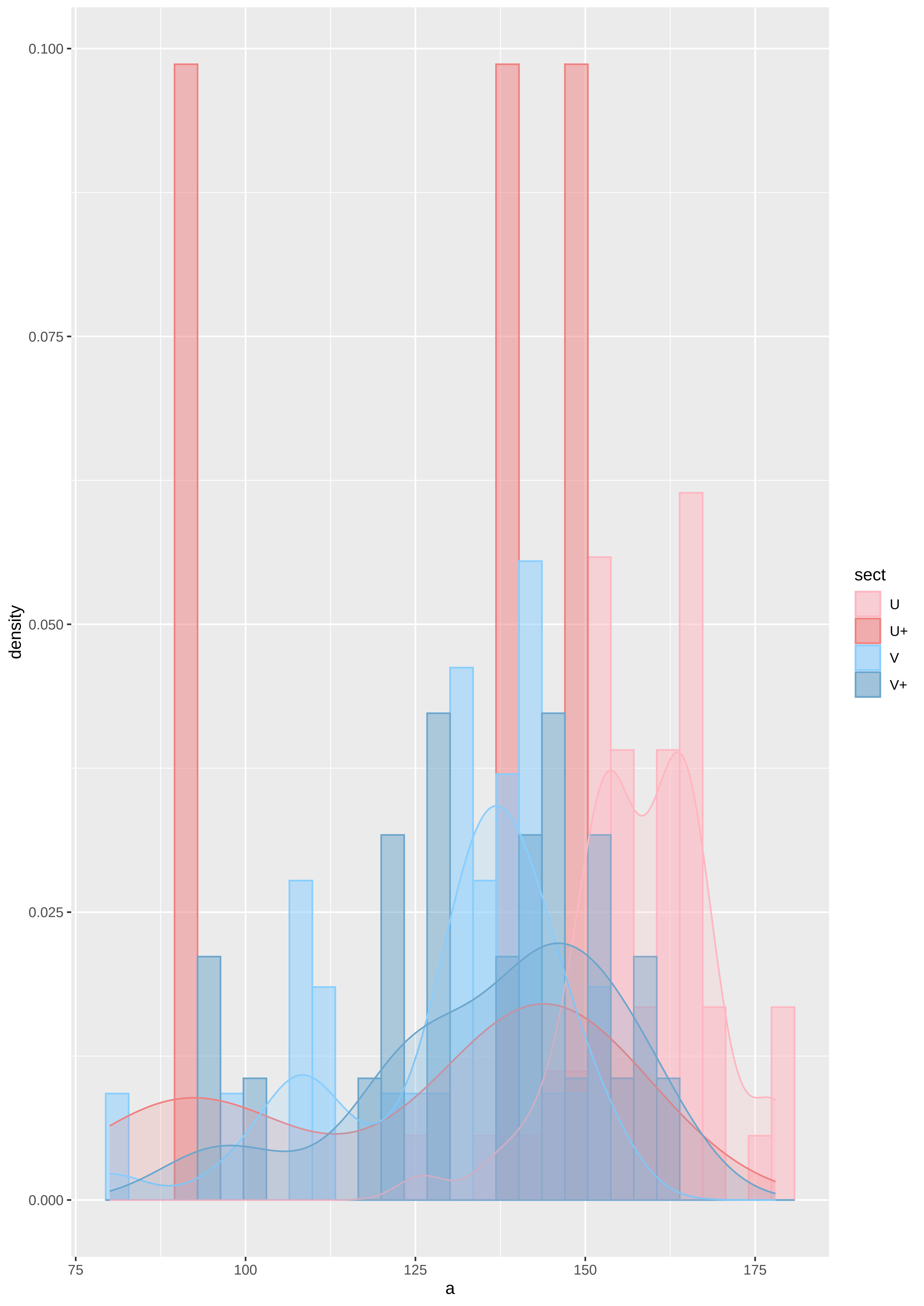

0.100
0.075
0.050
0.025
0.000
density
75
100
125
150
175
a
sect
U
U+
V
V+

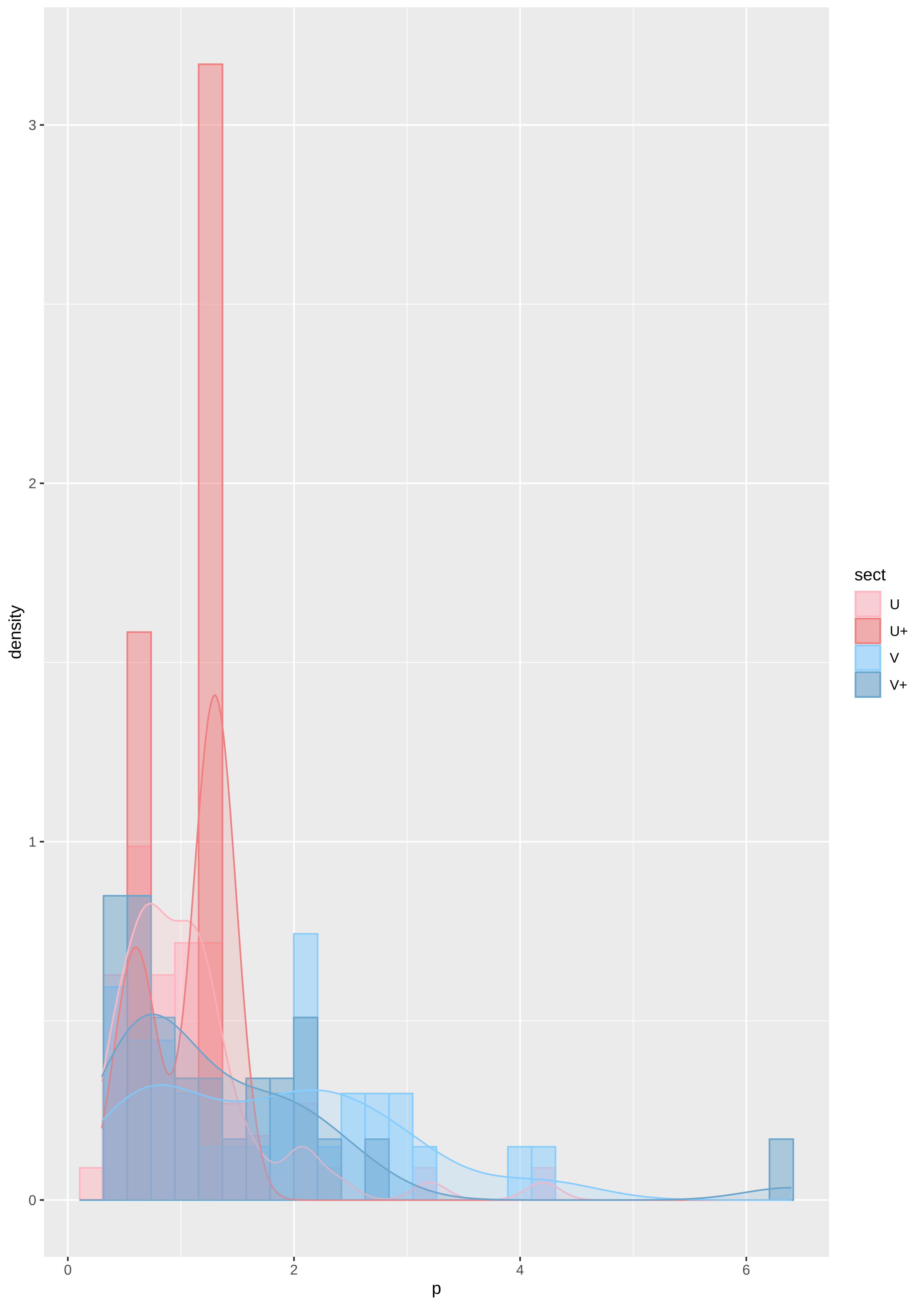

density
p
0
1
2
3
0
2
4
6
sect
U
U+
V
V+

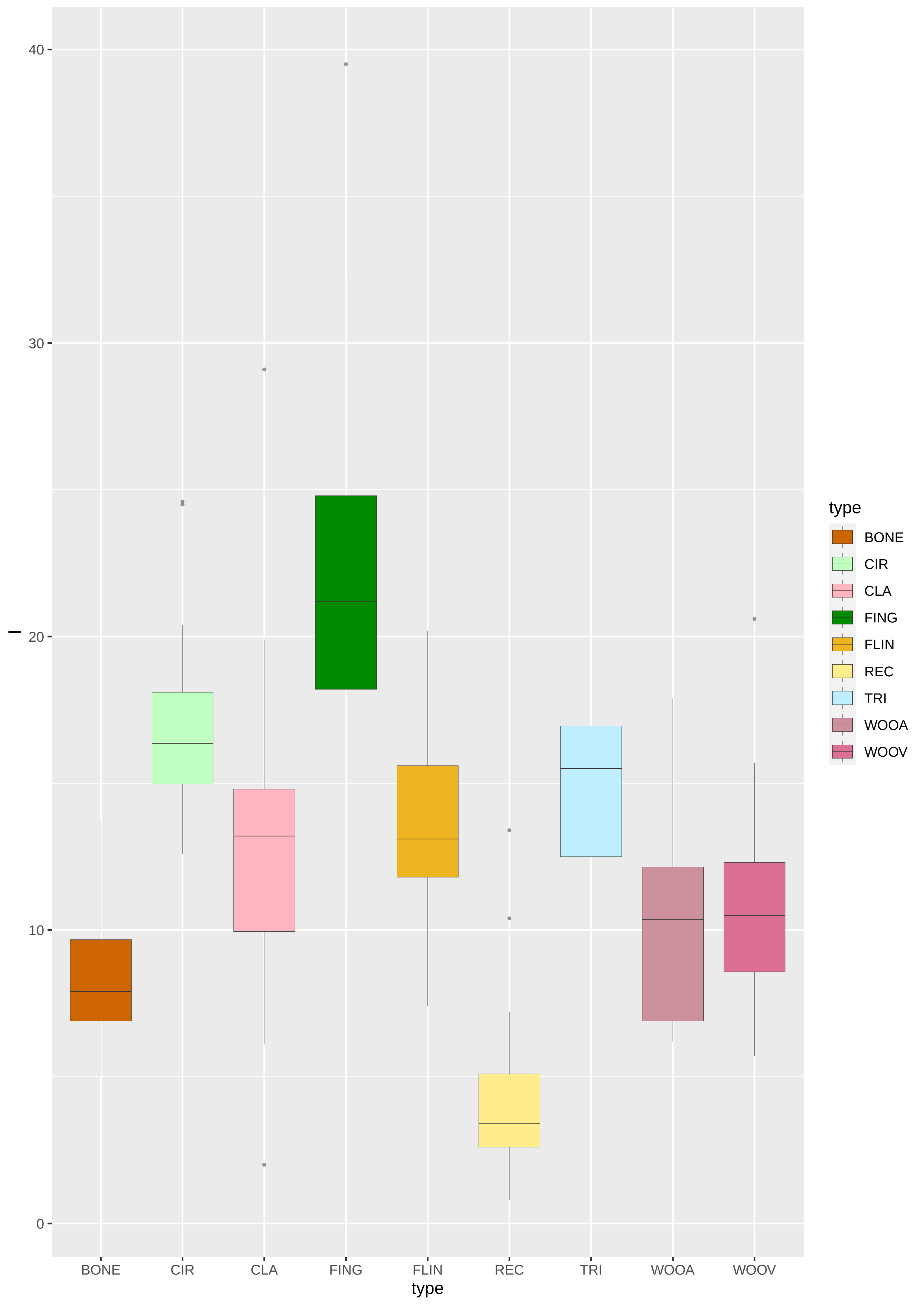

40
30
20
10
0
l
BONE
CIR
CLA
FING
FLIN
REC
TRI
WOOA
WOOV
type
type
BONE
CIR
CLA
FING
FLIN
REC
TRI
WOOA
WOOV

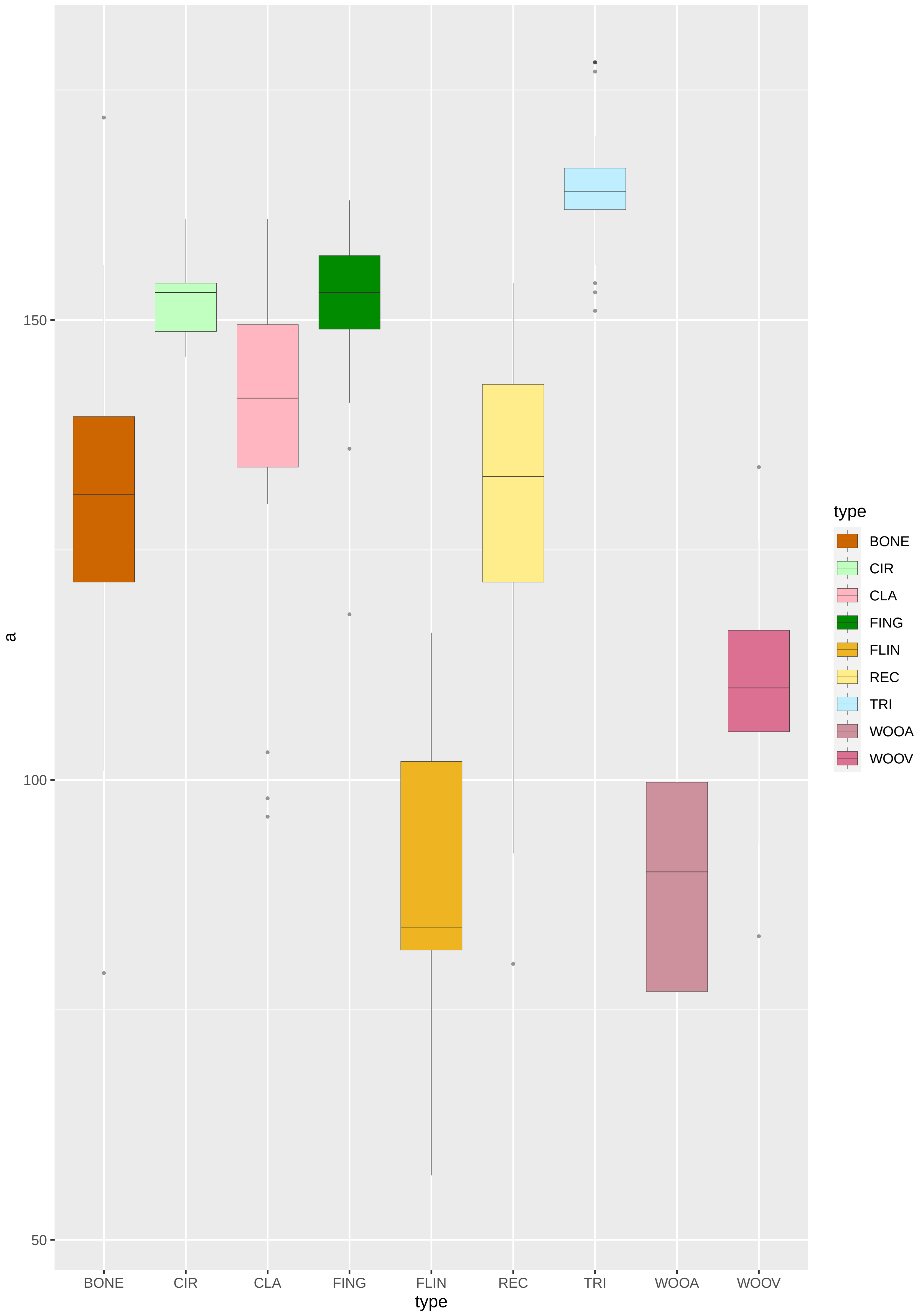
150
100
50
a
BONE
CIR
CLA
FING
FLIN
REC
TRI
WOOA
WOOV
type
type
BONE
CIR
CLA
FING
FLIN
REC
TRI
WOOA
WOOV

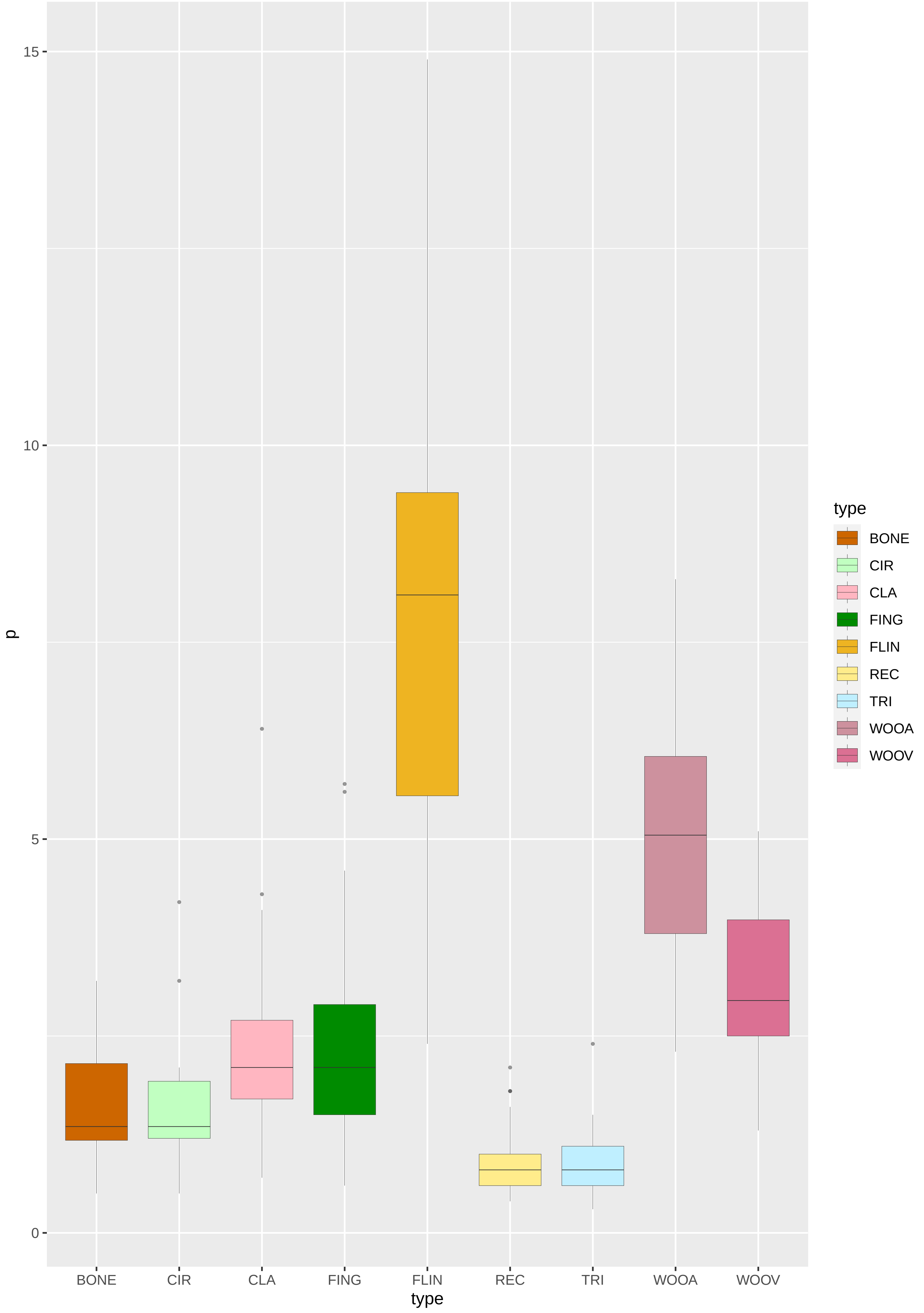

15
10
5
0
p
BONE
CIR
CLA
FING
FLIN
REC
TRI
WOOA
WOOV
type
type
BONE
CIR
CLA
FING
FLIN
REC
TRI
WOOA
WOOV

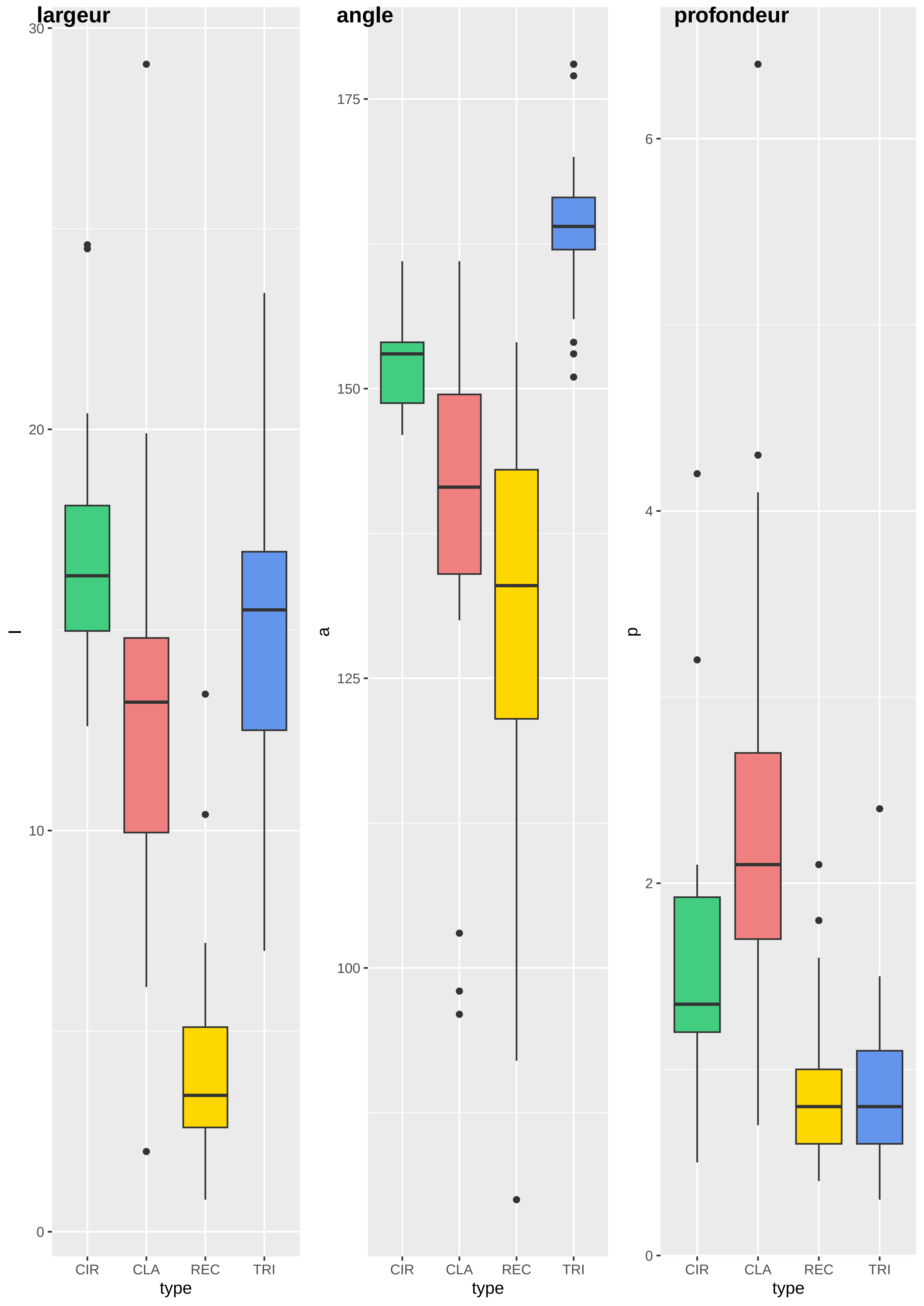
largeur
angle
profondeur
30
20
10
0
175
150
125
100
6
4
2
0
l
a
p
CIR
CLA
REC
TRI
type
CIR
CLA
REC
TRI
type
CIR
CLA
REC
TRI
type

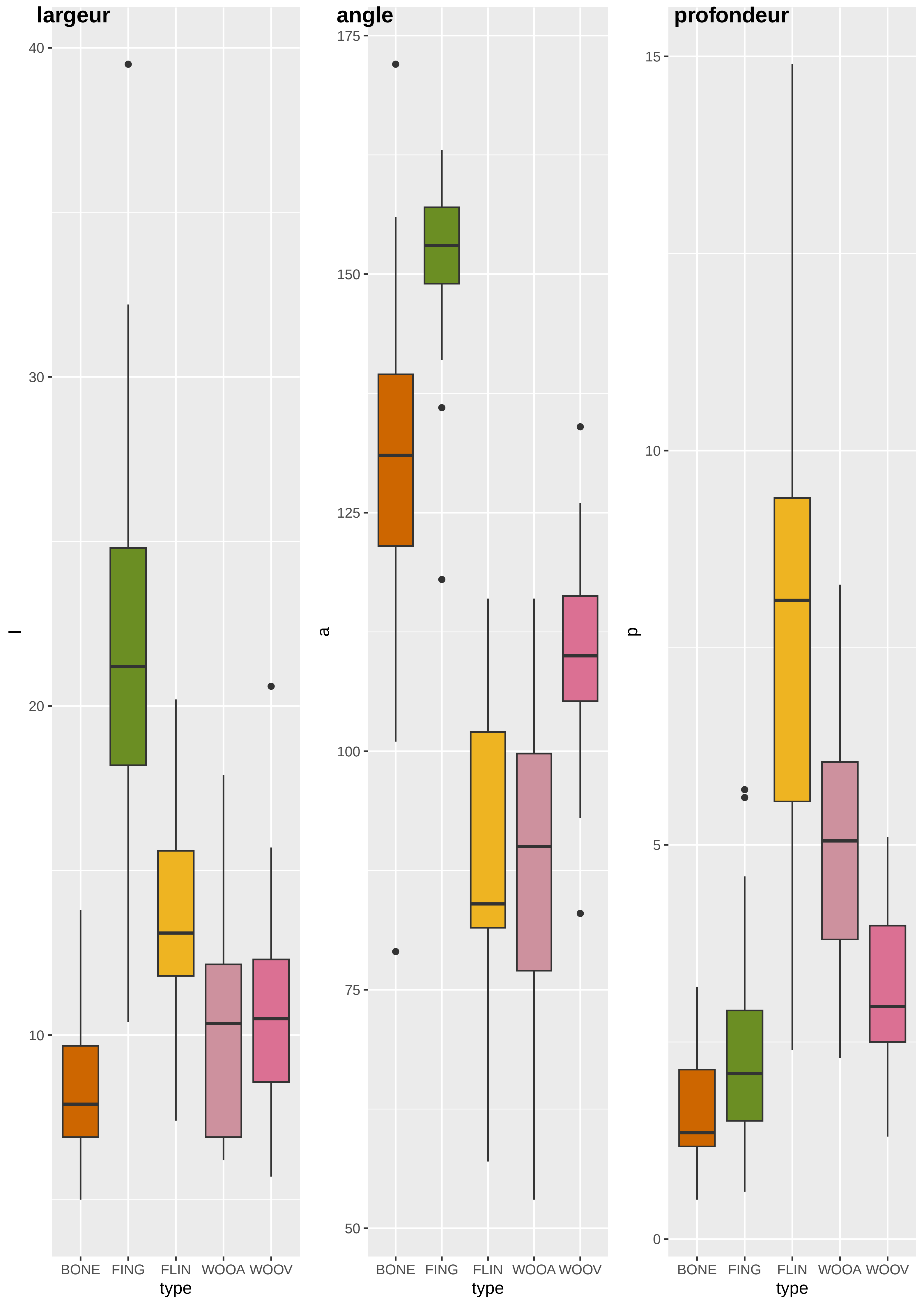

largeur
angle
profondeur
l
a
p
40
30
20
10
175
150
125
100
75
50
15
10
5
0
BONE FING FLIN WOOA WOOV
type

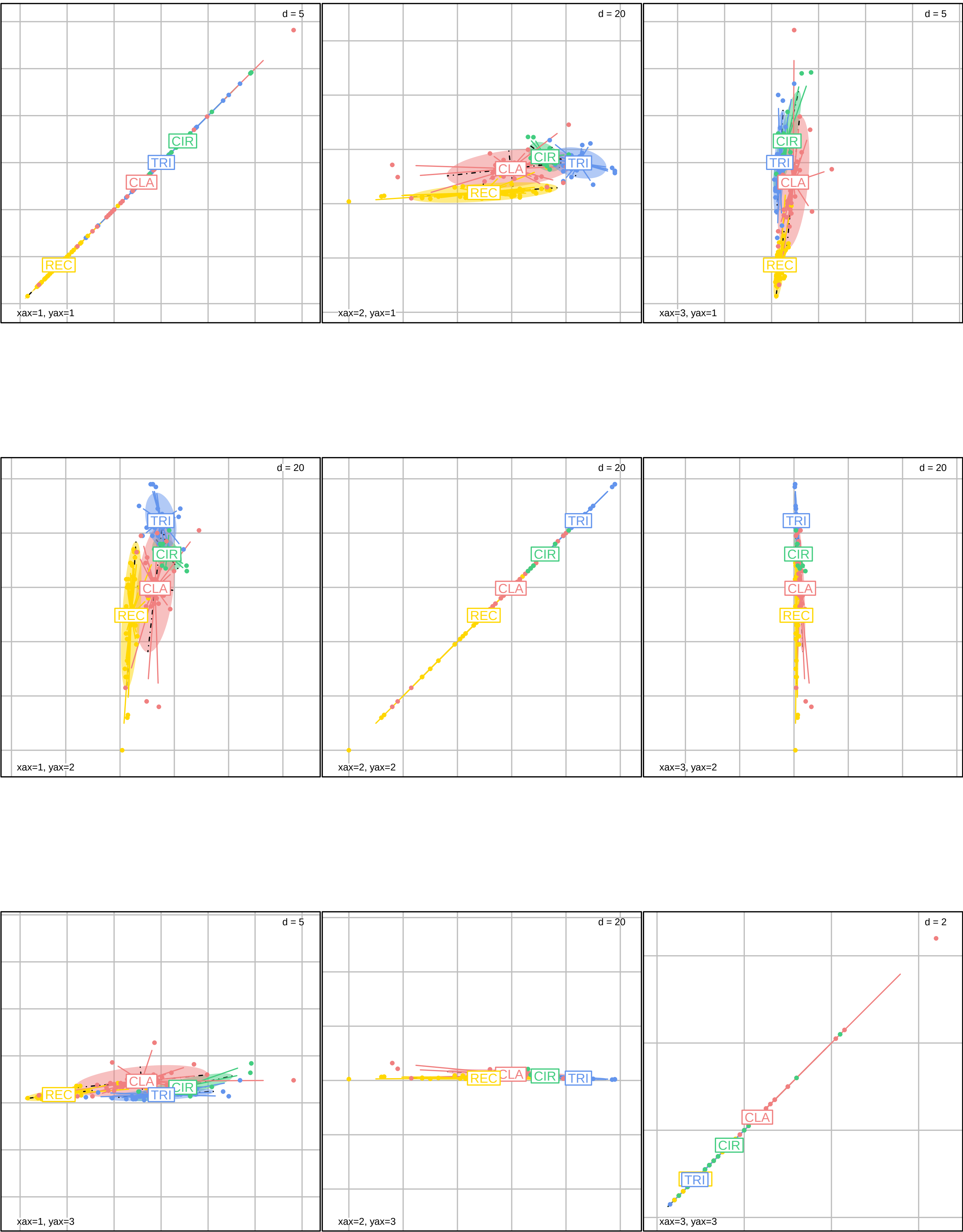
d = 5
CIR
TRI
CLA
REC
xax=1, yax=1
d = 20
CLA
CIR
TRI
REC
xax=2, yax=1
d = 5
CIR
TRI
CLA
REC
xax=3, yax=1
d = 20
TRI
CIR
CLA
REC
xax=1, yax=2
d = 20
TRI
CIR
CLA
REC
xax=2, yax=2
d = 20
TRI
CIR
CLA
REC
xax=3, yax=2
d = 5
CLA
CIR
REC
TRI
xax=1, yax=3
d = 20
CLA
CIR
REC
TRI
xax=2, yax=3
d = 2
CLA
CIR
TRI
xax=3, yax=3

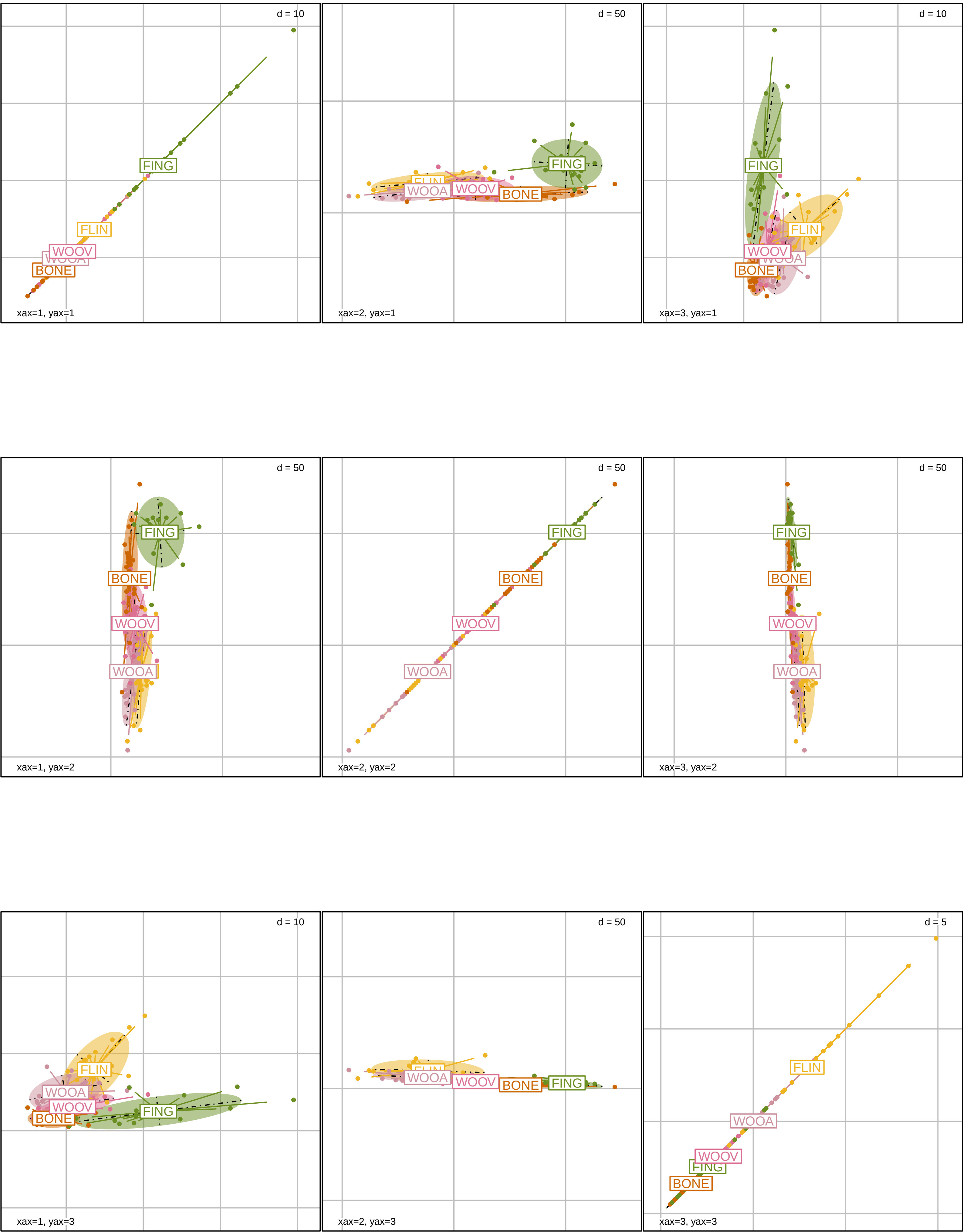
d = 10
FING
FLIN
WOOV
WOOA
BONE
xax=1, yax=1
d = 50
FING
FLIN
WOOA
WOOV
BONE
xax=2, yax=1
d = 10
FING
FLIN
WOOV
WOOA
BONE
xax=3, yax=1
d = 50
FING
BONE
WOOV
WOOA
xax=1, yax=2
d = 50
FING
BONE
WOOV
WOOA
xax=2, yax=2
d = 50
FING
BONE
WOOV
WOOA
xax=3, yax=2
d = 10
FLIN
WOOA
WOOV
FING
BONE
xax=1, yax=3
d = 50
WOOA
WOOV
BONE
FING
xax=2, yax=3
d = 5
FLIN
WOOA
WOOV
FING
BONE
xax=3, yax=3

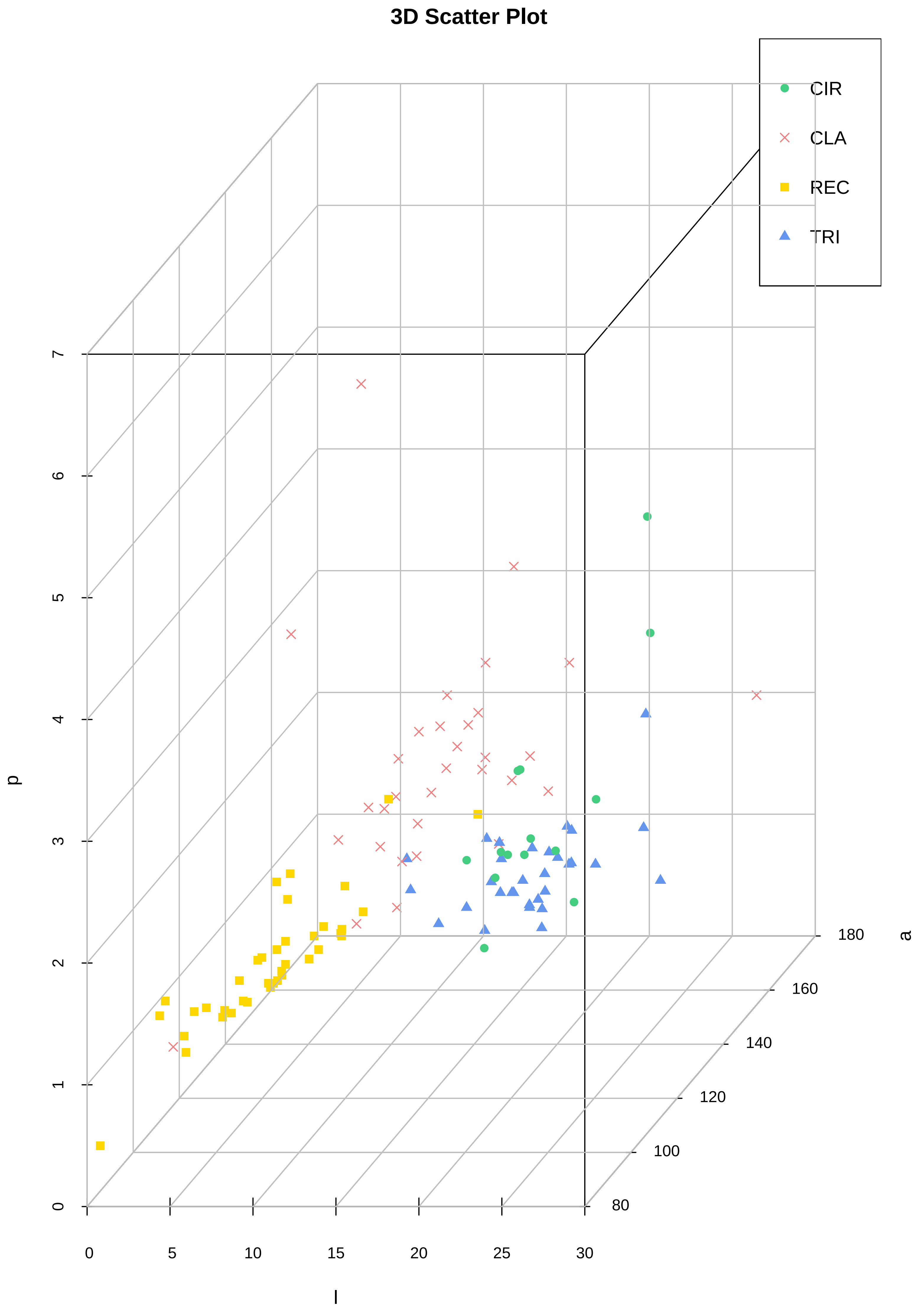
3D Scatter Plot
CIR
CLA
REC
TRI
p
0
1
2
3
4
5
6
7
l
0
5
10
15
20
25
30
a
80
100
120
140
160
180

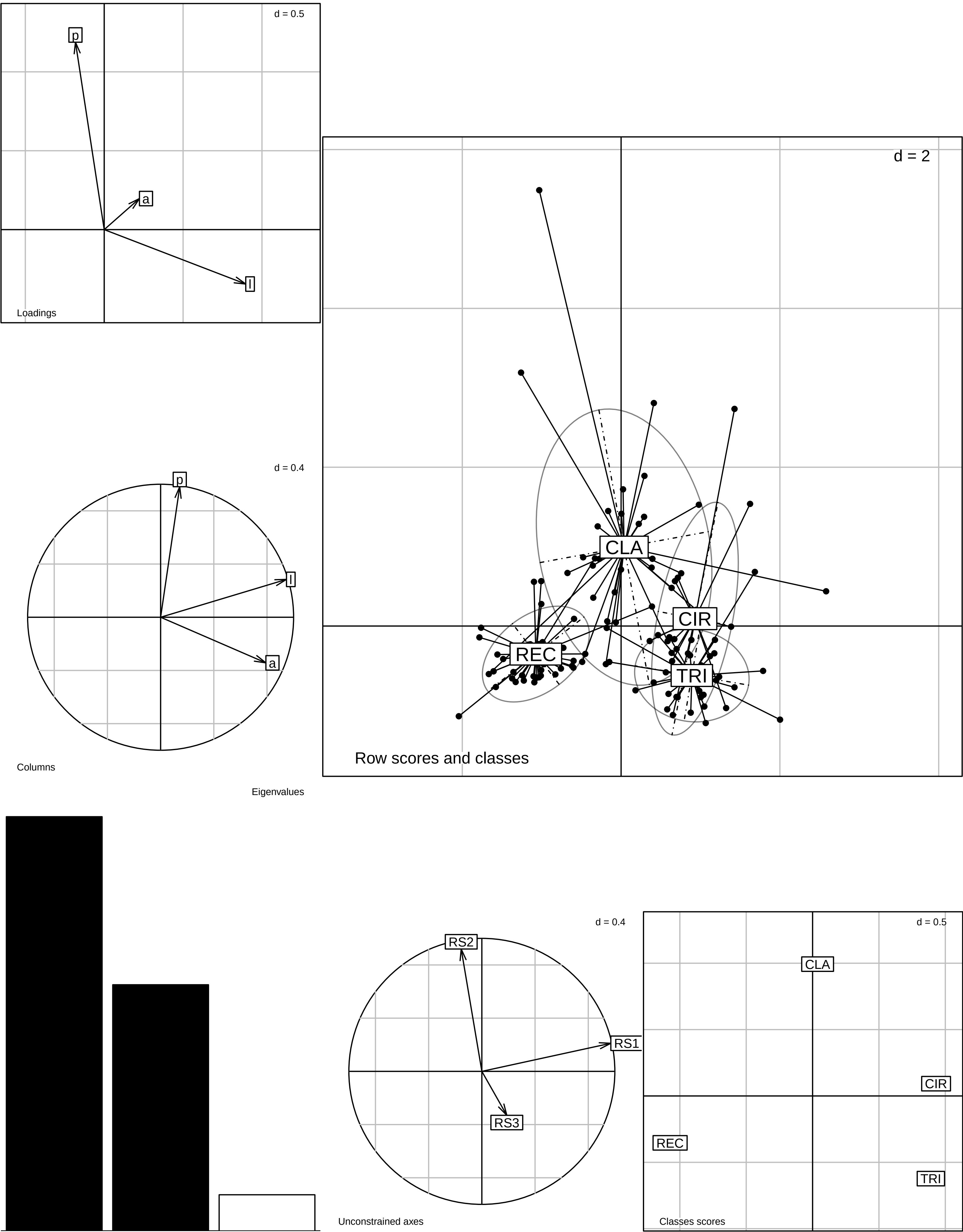
d = 0.5
p
a
l
Loadings
d = 2
CLA
CIR
REC
TRI
Row scores and classes
d = 0.4
p
l
a
Columns
Eigenvalues
d = 0.4
RS2
RS1
RS3
Unconstrained axes
d = 0.5
CLA
CIR
REC
TRI
Classes scores

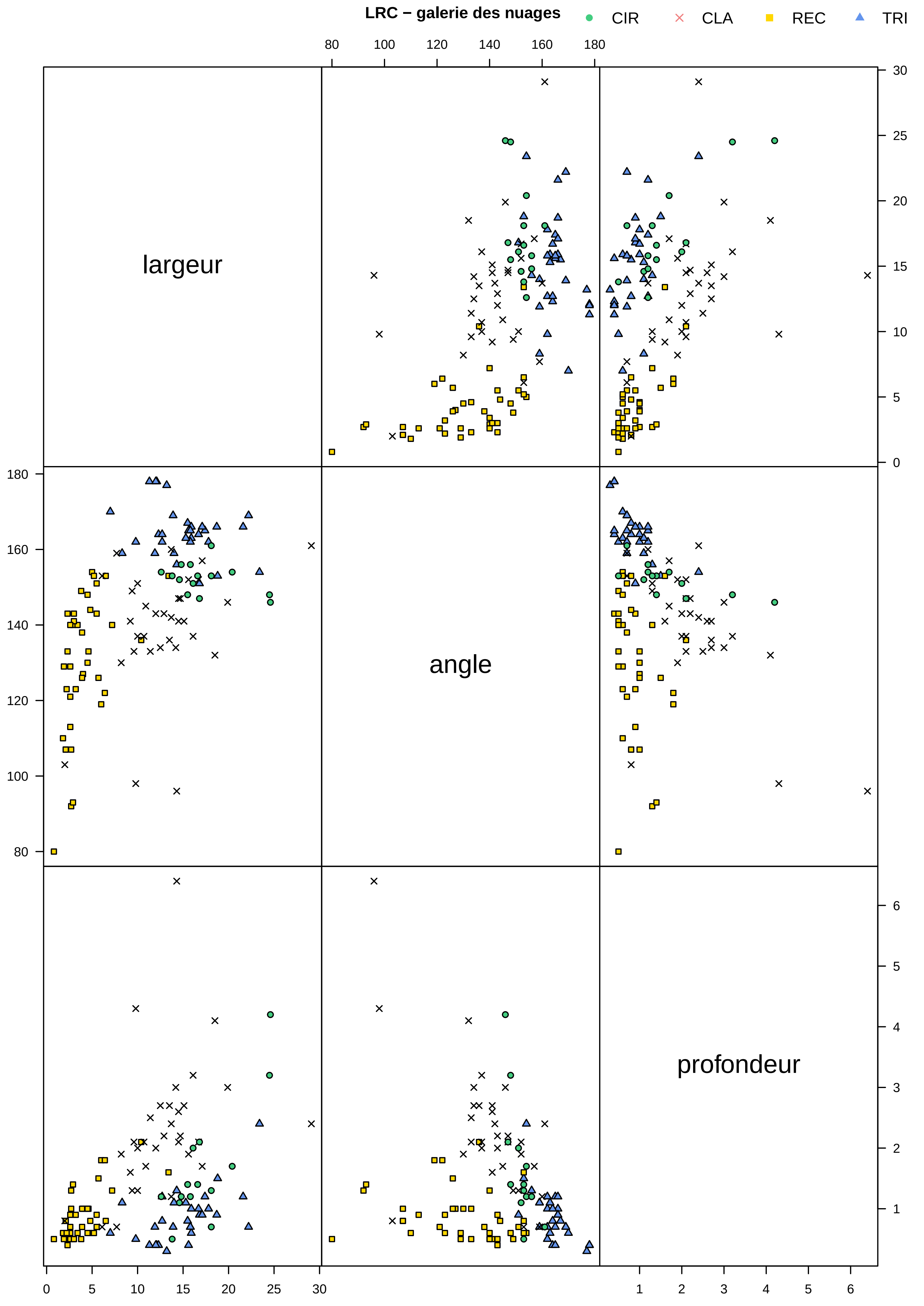

LRC − galerie des nuages
CIR
CLA
REC
TRI
largeur
angle
profondeur
80 100 120 140 160 180
0 5 10 15 20 25 30
1 2 3 4 5 6

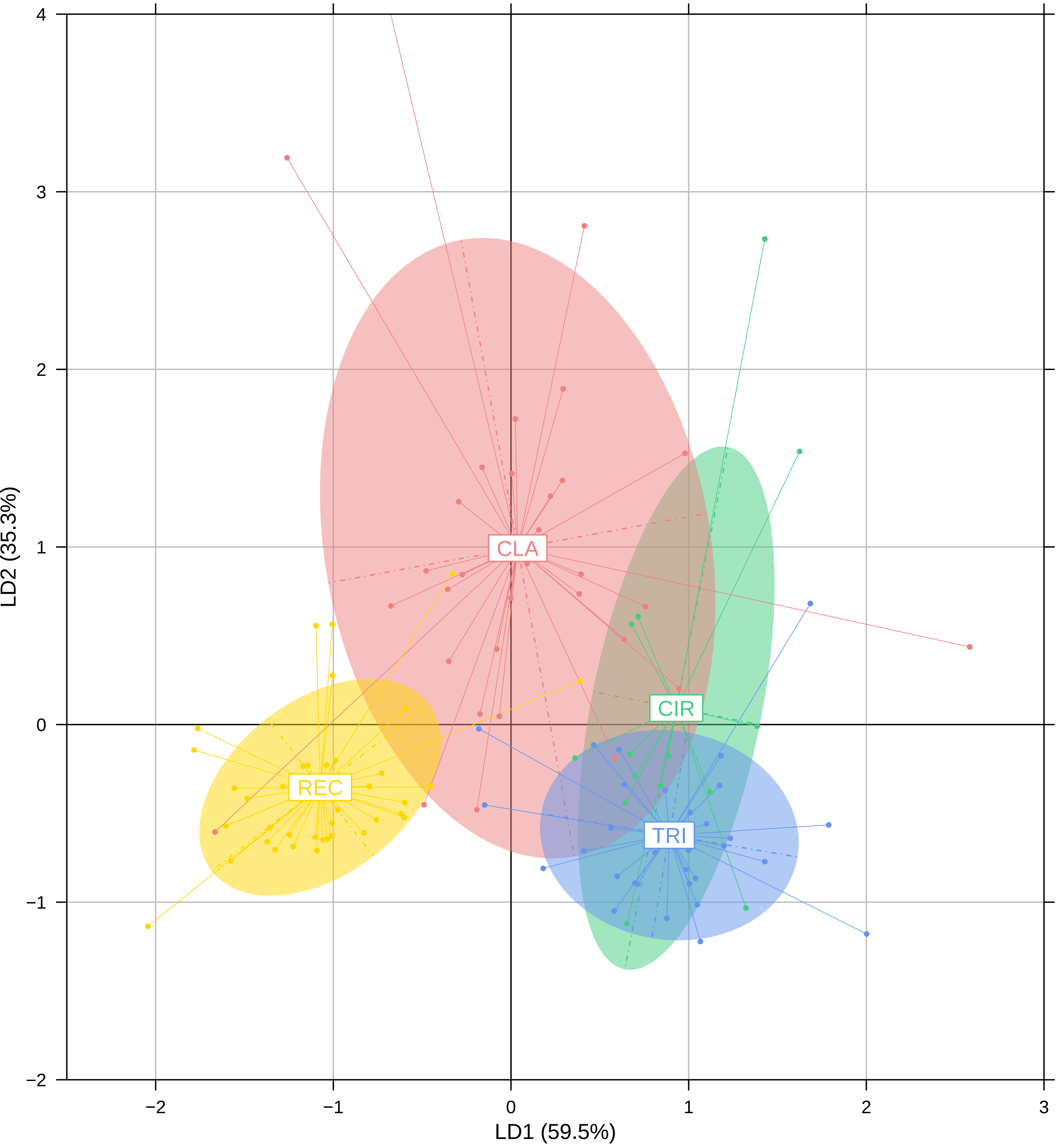

CLA
CIR
REC
TRI
LD1 (59.5%)
LD2 (35.3%)
−2
−1
0
1
2
3
4

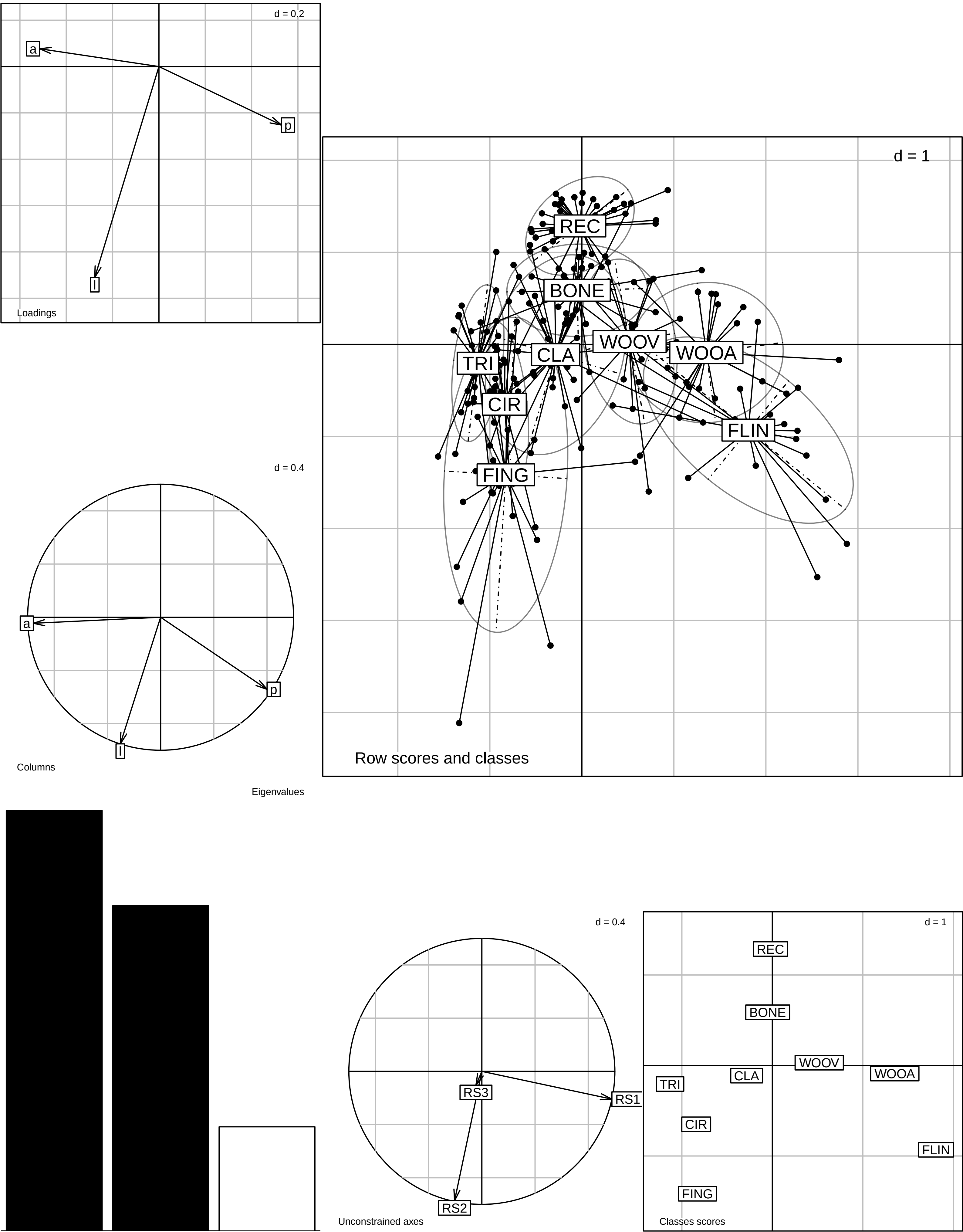

d = 0.2
a
p
l
Loadings
d = 1
REC
BONE
WOOV
WOOA
TRI
CLA
CIR
FLIN
FING
Row scores and classes
d = 0.4
a
p
l
Columns
Eigenvalues
d = 0.4
RS3
RS1
RS2
Unconstrained axes
d = 1
REC
BONE
WOOV
CLA
WOOA
TRI
CIR
FLIN
FING
Classes scores

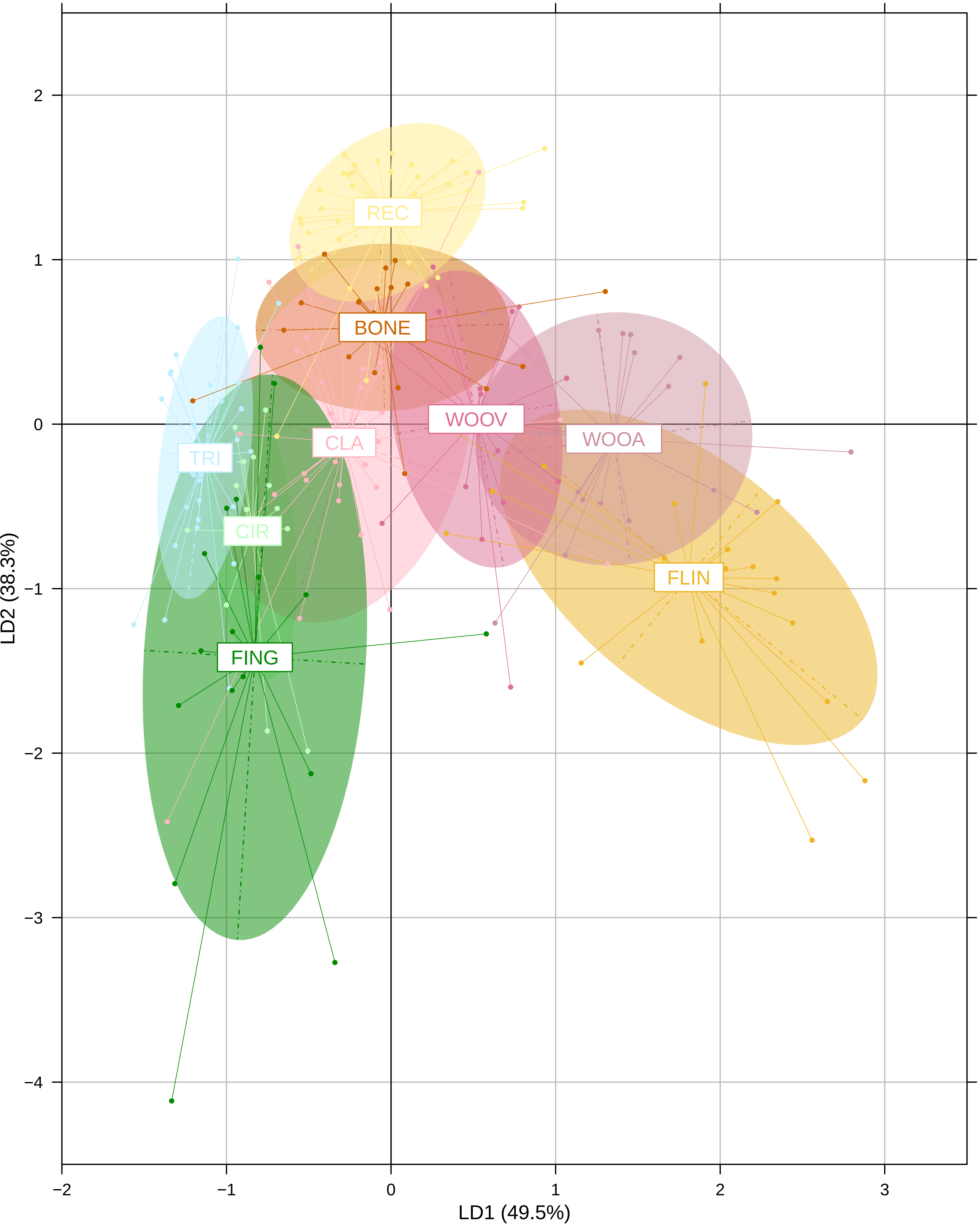
REC
BONE
WOOV
WOOA
CLA
TRI
CIR
FLIN
FING
2
1
0
−1
−2
−3
−4
−2
−1
0
1
2
3
LD1 (49.5%)
LD2 (38.3%)

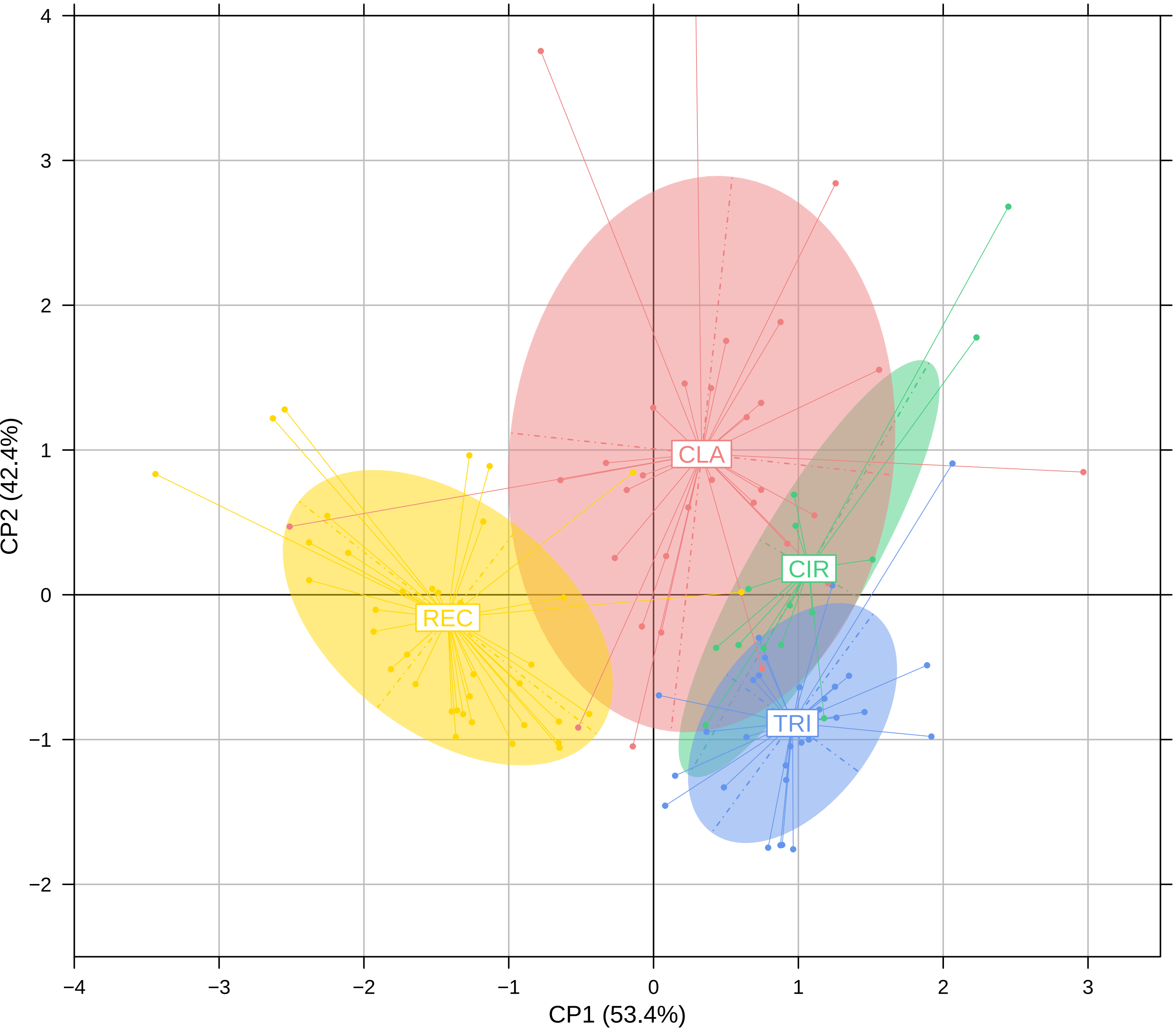

CLA
CIR
REC
TRI
CP1 (53.4%)
CP2 (42.4%)

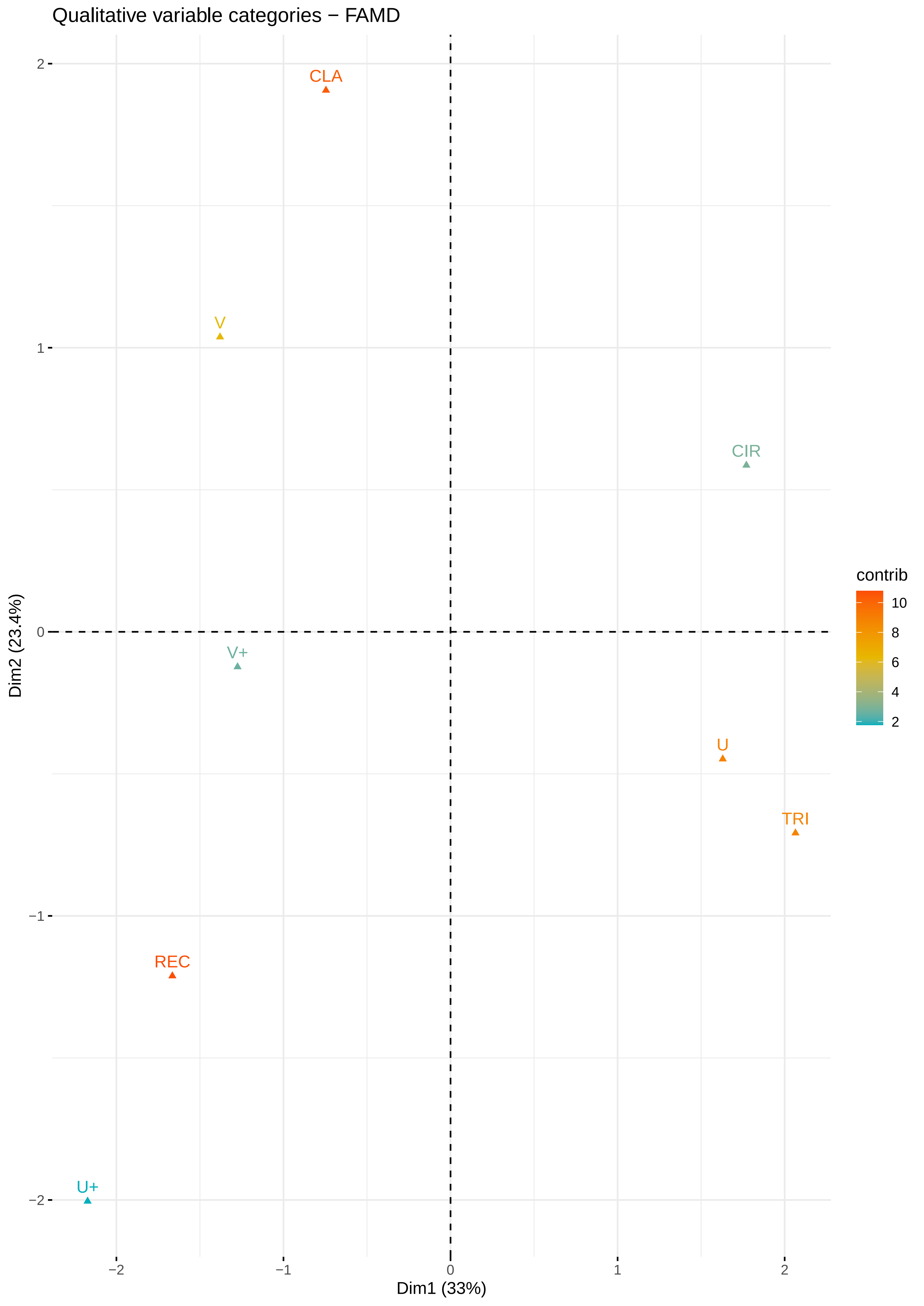

Qualitative variable categories − FAMD
CLA
V
CIR
V+
U
TRI
REC
U+
contrib
10
8
6
4
2
Dim2 (23.4%)
Dim1 (33%)
2
1
0
−1
−2
−2
−1
0
1
2

Individuals − FAMD

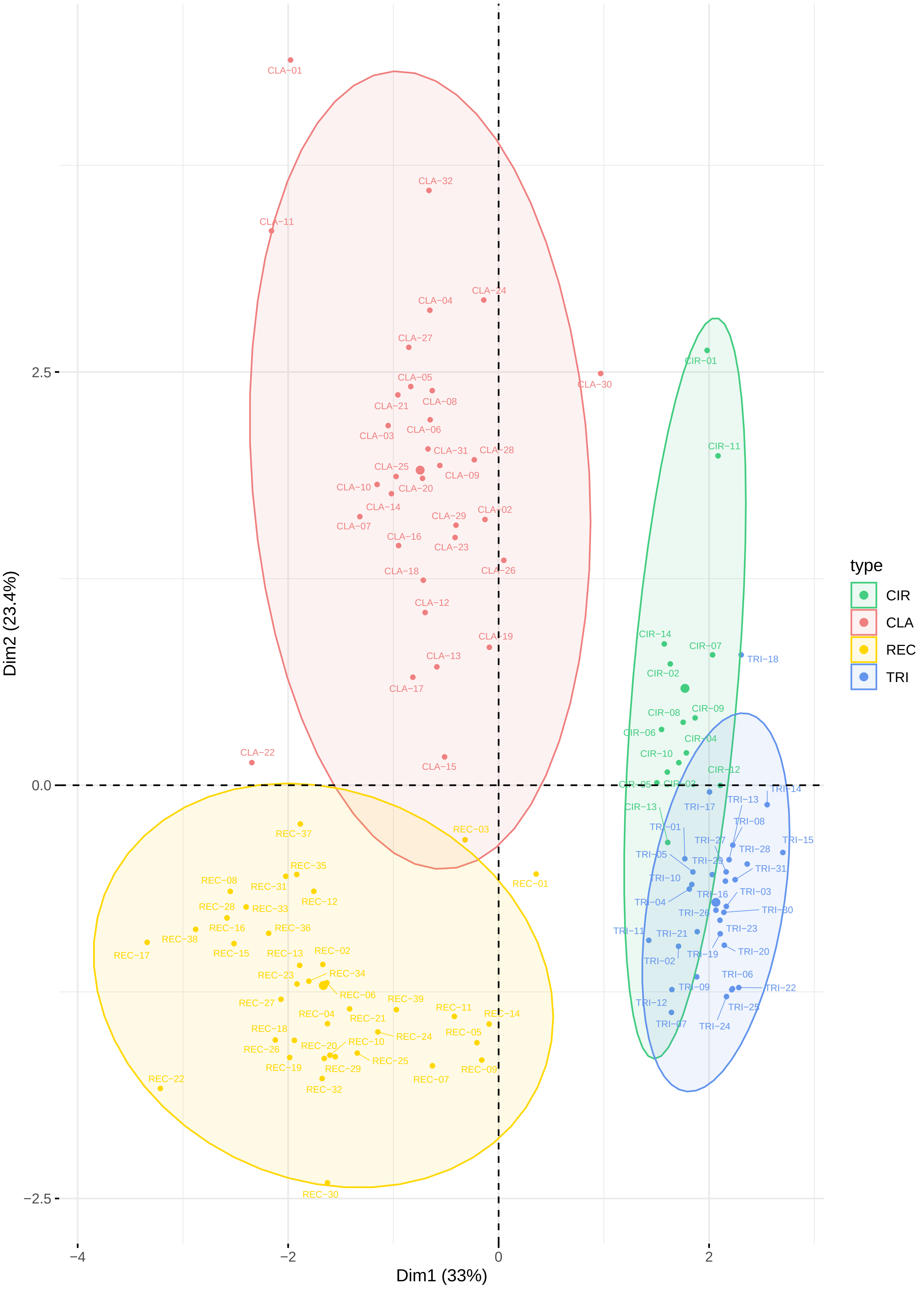

Supplement: S3 Text — (PDF) [file pone.0286568.s003.pdf]
