# Supplementary material for: The earliest unambiguous Neanderthal engravings on cave walls: La Roche-Cotard, Loire Valley, France
Source: PLoS One. 2023 Jun 21;18(6):e0286568. doi: 10.1371/journal.pone.0286568 (PMC10284424; doi:10.1371/journal.pone.0286568)
Supplement: S4 Text — (PDF) [file pone.0286568.s004.pdf]

Curvature analysis of the triangular panel

Y. Egels

The initial goal was to extract a geometric local characteristic of the surface in relation with the shape of the lines. The surface curvature is a good candidate, being independent of the location and orientation of the lines, and easily extractable from the 3D model produced by photogrammetry.

A first attempt, obtained with the well known 3D software Meshlab, showed that the curvature give effectively pertinent indication of the depth, the width, the profile and the relative position of the lines. But the software used has no possibility of adjusting some important parameters.

First, the smoothing of the surface was very high, giving a too regular description of the shape. In this case, smoothing is necessary, due to the granularity of the support, and the high density of the 3D model (circa 0,7mm between points). But a realistic smoothing radius must be similar to the width of the lines, 5 to 10 mm.

Next, Meshlab gives either mean curvature or Gaussian curvature, while the most meaningful would be the maximum curvature, witch is perpendicular to the line (the minimum being along the line, essentially related to the shape of the support.

An other traditional 3D software – Cloudcompare – can export the maximum curvature, but only in absolute value, preventing the distinction between concavity and convexity.

To pursue the objective, we decided to develop a specific function in the software Cumulus, developed by one of the authors. Cumulus is a 3D software oriented to archaeological applications of 3D models, essentially photogrammetric or lidar. It can produce georeferencing, cross sections, orthophotos, geometric control, blocs management for anastylosis, etc. It was used in La Roche Cotard to produce a majority of the geometric documentation and GIS. With that support, it was only necessary to write a specific module for estimating the principal curvatures by quadratic approximation by least squares in a neighborhood controlled by a parameter.

The source code (in Pascal) is available below.

```
Procedure TObjet.Courbure(Index: ptrint; Data: Pointer; Item:
TMultiThreadProcItem);
var
  i,j,k,i1,i2,N : integer;
  NV            : integer;
  DP            : TVector9f;
  G             : TVector3f;  // barycentre, valeurs propres
  M,V           : TVector3f;
  Mat           : MC_Plein;
  PosInc        : array[1..4] of integer;
  Observation   : array[1..4] of type_terme;
  A,B,C,D,C1,C2,K1,K2,S : double;
// Attention, ces variables doivent être accessibles
// si l'option recherche par voisinage est activée
  NTV             : integer;            // nombre de triangles connectés
  NPV             : integer;            // Nombre de points connectés
  IV              : array of integer;   // indices des points connectés
  PV              : array of TVector3f; // Coord des points connectés
  NMoy            : TVector3f;

Procedure VoisinSphere;
var j : integer;
begin
  with Nuage,Delaunay do for j := 0 to pred(ListePoint.count) do begin
    msub(Positions[j],V,M);
    if Norme(M) > MaxDist then continue;
    IV[NPV] := j;
    PV[NPV] := Positions[j];
    madd(G,PV[NPV],G);
// Normale du premier triangle
    madd(NMoy,Normale[Sommets[j].PointD.Trp],NMoy);
    inc(NPV);
  end;
end;
begin

  Mat := Mc_Plein.Cree_Matrice_Normale(3);
  for k := 1 to 4 do PosInc[k] := k;
  with Nuage,Delaunay do begin
    N := ListePoint.Count;
    K := N div NbThread;
    I1 := Index*K;
    i2 := min(N,(Index+1)*k-1);

    NPV := 10000; //Au pif
    setlength(IV,3*NPV);
    setlength(PV,3*NPV);

// Points dans la sphère

    with ListePoint do for i := i1 to i2 do  with Sommet[i] do begin
      if double then actif := false;
      if not actif then  continue;
      V := positions[i];
      NPV := 0;
      MRaz(G);
      MRaz(NMoy);
      VoisinSphere;
      mdps(G,NPV,G);
      mdps(Nmoy,NPV,NMoy);
//      Notes.note;
//FCumulus.StatusBar.Panels[6].Text :=  format('%4d %8d %6d',[index,i,npv]);

// compensation plan moyen
```

```
      Mat.Remise_a_Zero;
      for k := 0 to pred(NPV) do if IV[K] >= 0 then begin
        msub(PV[K],G,M);
        observation[1] := M[0];
        observation[2] := M[1];
        observation[3] := M[2];
        observation[4] := PScal(M,NMoy);
        Mat.Normalisation(Observation,PosInc,4,1.0);
      end;
      mat.Resolution;

      M := NMoy;
      for k := 0 to 2 do NMoy[k] := Mat.Solution[k+1];
      msub(M,NMoy,M);

// Base du plan

      D := 1;
      // La + petite coordonnée donne le vecteur le plus indépendant
      for j := 0 to 2 do if abs(NMoy[j]) < D then begin
        D := abs(NMoy[j]);
        k := j;
      end;
      mraz(M); M[k] := sign(NMoy[j])*1.0;
      Pvect(NMoy,M,M);
      VUnite(M,M);
      mmov(M,DP[0..2]);
      mmov(NMoy,DP[6..8]);
      PVect(NMoy,M,M);
      mmov(M,DP[3..5]);

// compensation courbure

      G := Positions[i];
      Mat.Remise_a_Zero;
      for k := 0 to pred(NPV) do if IV[K] >= 0 then begin
        msub(PV[k],G,M);  // dans la base du plan
        mprd(DP,M,M);
        PV[k] := M;
        observation[1] := sqr(M[0]);   //
        observation[2] := 2*M[0]*M[1];
        observation[3] := sqr(M[1]);
        observation[4] := M[2];
        Mat.Normalisation(Observation,PosInc,4,1.0);
      end;
      Mat.resolution;
      A := Mat.Solution[1];
      B := Mat.Solution[2];
      C := Mat.Solution[3];
      D := Mat.Sigma_0;
      S := 0;

      for k := 0 to pred(NPV) do if IV[K] >= 0 then begin       // résidus
         M := PV[k];
         D := A*sqr(M[0])+2*B*M[0]*M[1]+C*sqr(M[1]);
         D := M[2]-D;S := S+sqr(D);
         //Notes.Note('%8d    %15.4f', [IV[k],D]);
      end;
      S := sqrt(S/NV);

// Valeurs propres

      D := sqrt(sqr(A)+4*sqr(B)+sqr(C)-2*A*C);
```

```
      C1 := (A+C+D);
      C2 := (A+C-D);
      if abs(C1) > abs(C2) then begin K1 := C1; K2 := C2; end
      else begin  K1 := C2; K2 := C1; end;
      with Sommets[i].PointD do begin X := K1; Y := K2; Z := X; end;
    end;
  end;
  mat.free;
end;
```
