# Supplementary figures and images for: The earliest unambiguous Neanderthal engravings on cave walls: La Roche-Cotard, Loire Valley, France
Source: PLoS One. 2023 Jun 21;18(6):e0286568. doi: 10.1371/journal.pone.0286568 (PMC10284424; doi:10.1371/journal.pone.0286568)

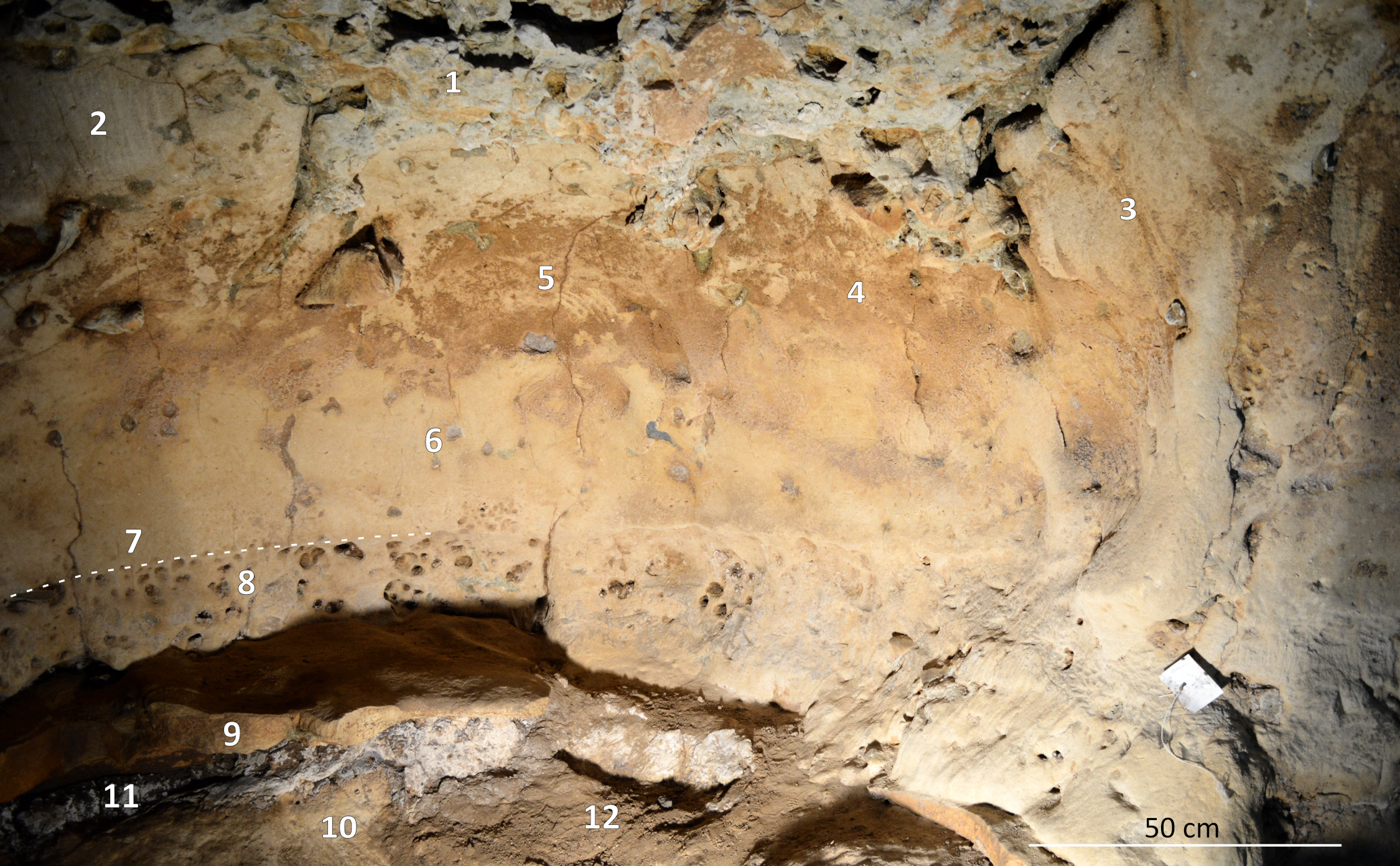

Supplement: S1 Fig — 1: Coniacian quartzitic sandstone; 2 and 3: Graphic entities; 4 and 5: tuff wall still covered with a light brown film showing local removal of some of the film due to erosion. The six traces (5) are due to a metal tool used by the excavators in 1912; 6: tuff wall with the brown film removed; 7: overhang; 8: small decarbonation recesses; 9: chert layer; 10: yellow Turonian tuff; 11: cavity filled with compact red decarbonation clay; 12: modern sedimentary layer covering the compact layer. (TIF) [file pone.0286568.s006.tif]

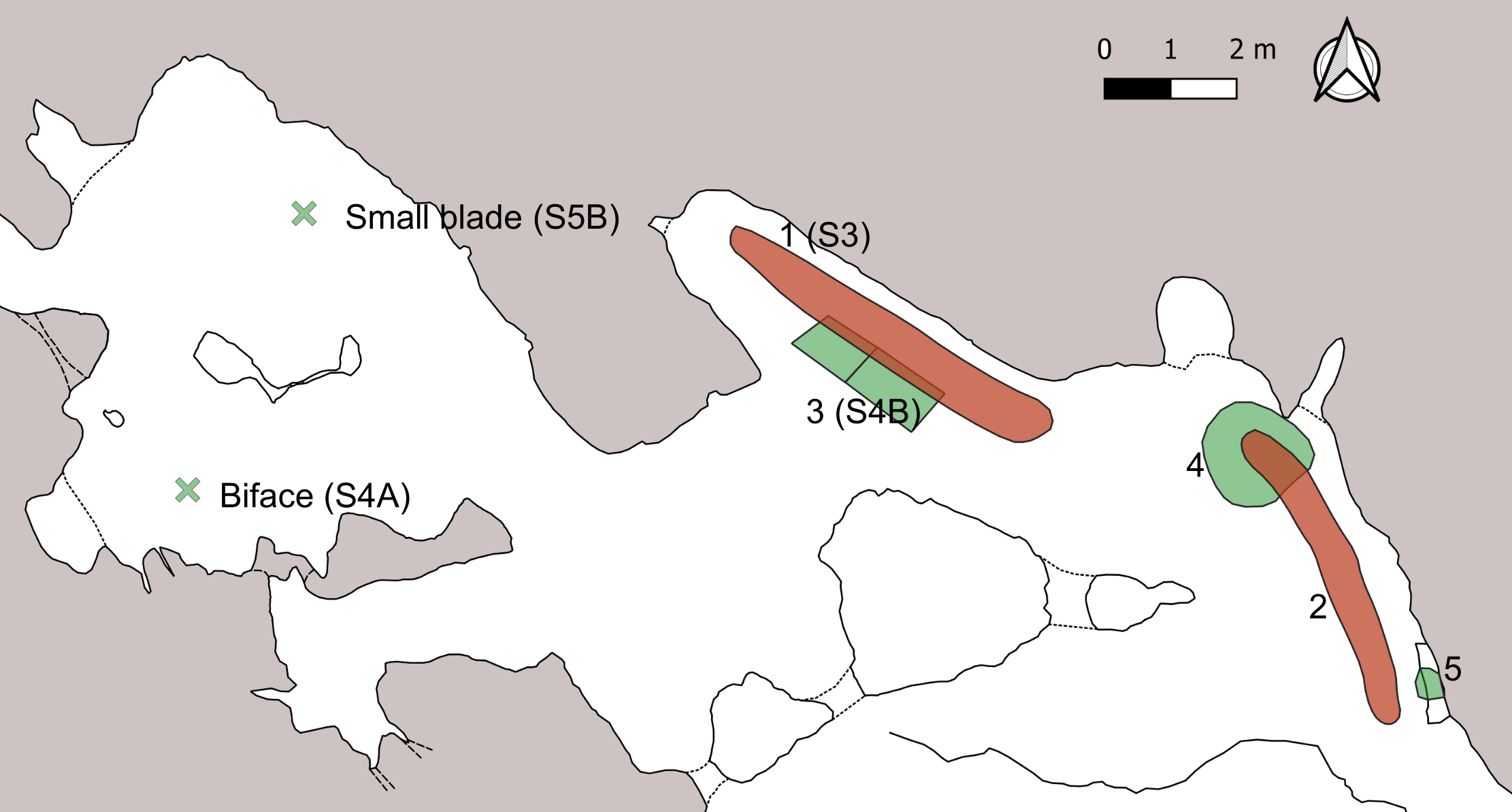

Supplement: S2 Fig — The two brown zones 1 and 2 locate F. d’Achon’s discoveries (1. typical Mousterian with Levallois flaking, surmounted by the Mousterian of Acheulean tradition, 2. typical Mousterian with Levallois flaking). The three green zones locate recent discoveries (3, 4 and 5. Typical Mousterian with Levallois debitage). S3 indicates the place of lithic industry drawings in S3 Fig. S4B indicates the place of typical Mousterian with Levallois flaking in S4 Fig. S4A indicates the place where the Mousterian of Acheulean tradition triangular broken biface has been discovered as well as its drawing and photograph in S4 Fig. S5B indicates the place where the flint blade has been discovered and its macro and microphotography in S5 Fig. (TIF) [file pone.0286568.s007.tif]

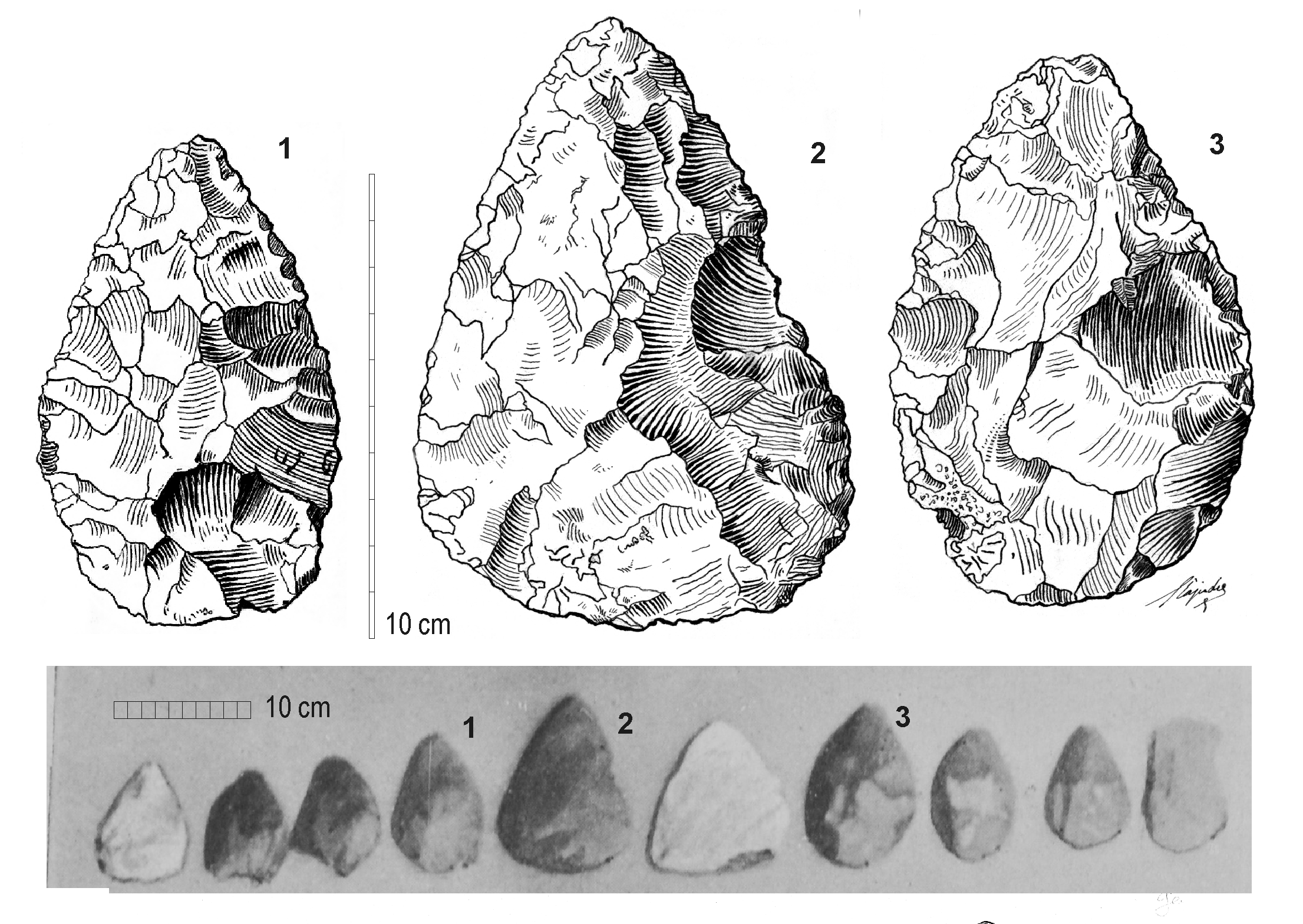

Supplement: S3 Fig — Mousterian of Acheulean Tradition bifaces (1, 2, 3) discovered in 1912 (drawing M. Lajudie in Dubreuil-Chambardel, La Touraine préhistorique. 1923). Pictures of ten bifaces [19]. (TIF) [file pone.0286568.s008.tif]

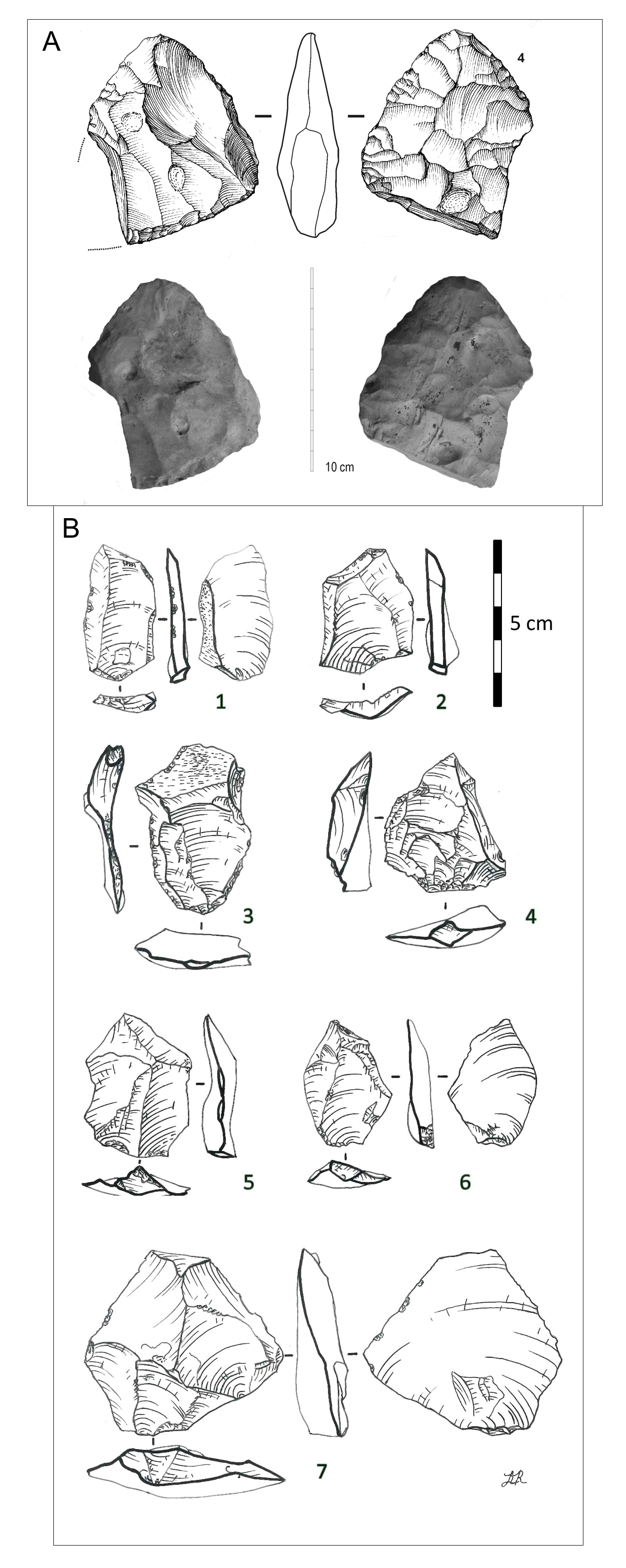

Supplement: S4 Fig — A. Acheulean Tradition Mousterian biface discovered in the South Pillar Chamber (S2 Fig), photo and drawing T. Aubry. B. Typical Levallois flakes discovered in the Mousterian Gallery and in front of the entrance (S2 Fig Zones 3, 4 et 5), drawing L.A. Millet-Richard. (TIF) [file pone.0286568.s009.tif]

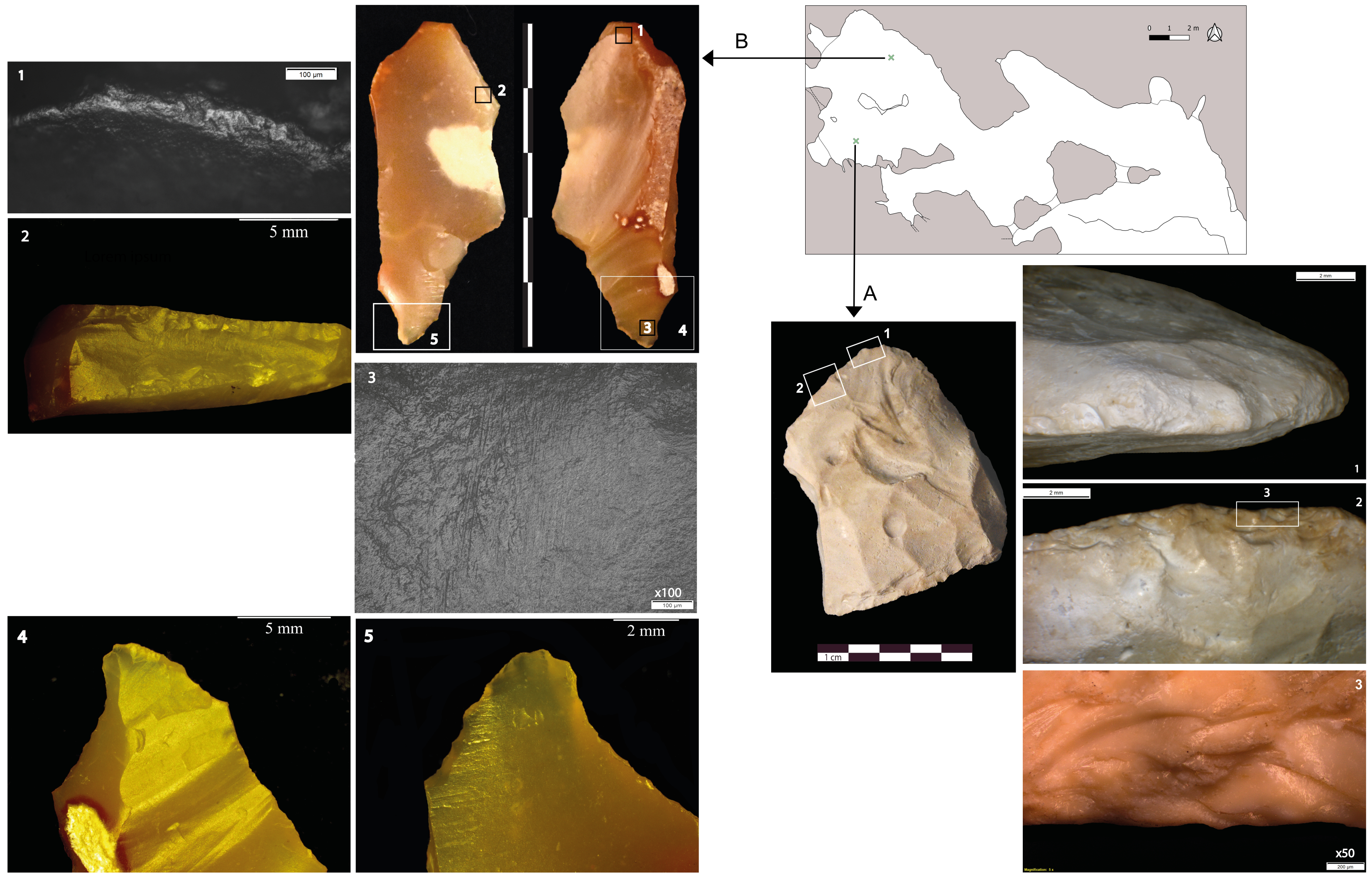

Supplement: S5 Fig — A. Broken triangular flint biface discovered not deeply in the sediment accumulated in a window of the chert layer. It shows significant rounding of its distal part (1, 2, 3), creating a dull, abrupt edge most probably produced by transverse contact with mineral matter. The other edges of this implement do not display such characteristics. B. Second stone implement, made on a blade, used to process mineral matter and hide. Three zones of use are identified: the first suggests hide processing (1). The second and third use-areas present features which indicate scraping soft, abrasive, mineral matter (2, 3). (TIF) [file pone.0286568.s010.tif]

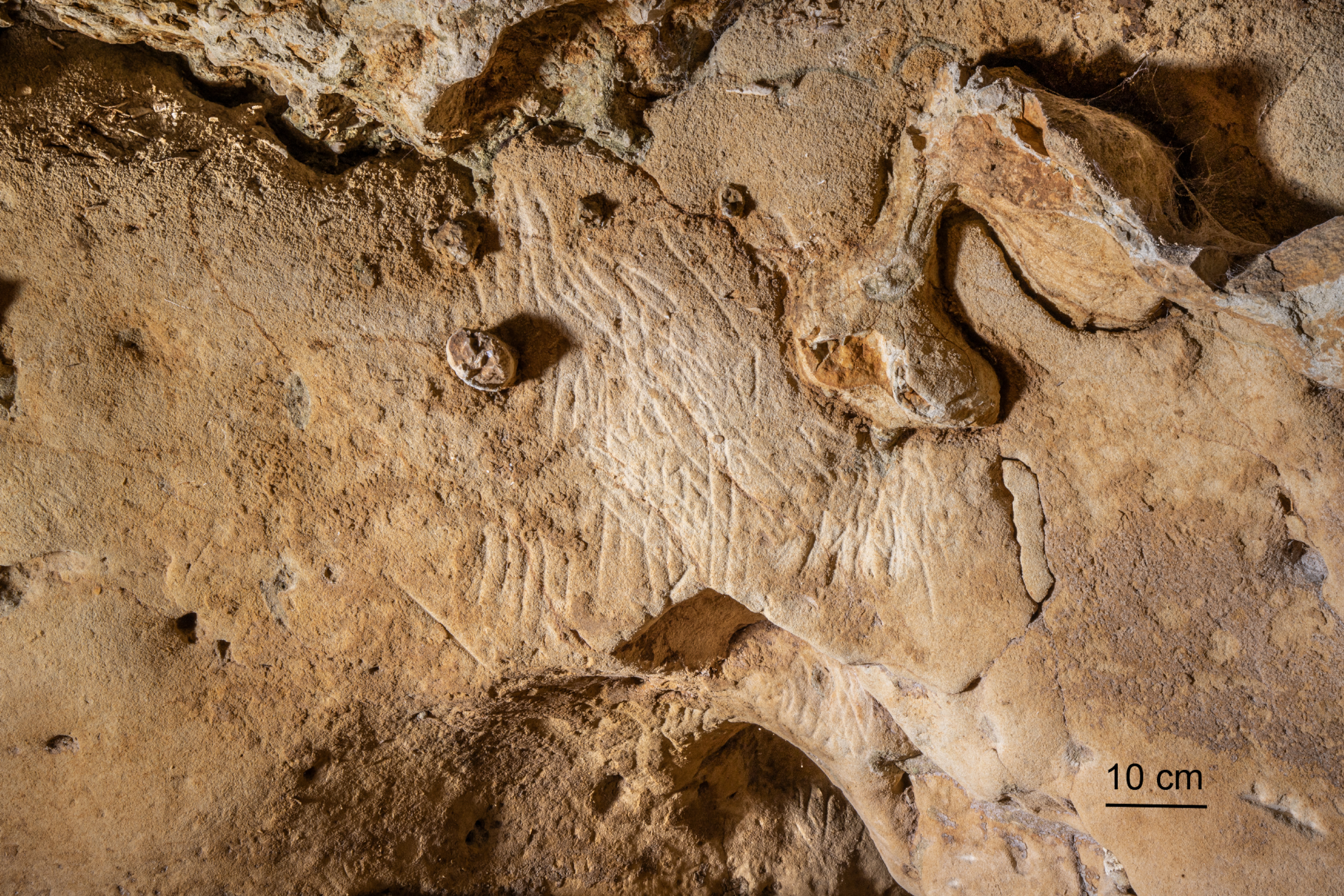

Supplement: S6 Fig — The shape and distance between marks are consistent with traces of Ursus sp. (TIF) [file pone.0286568.s011.tif]

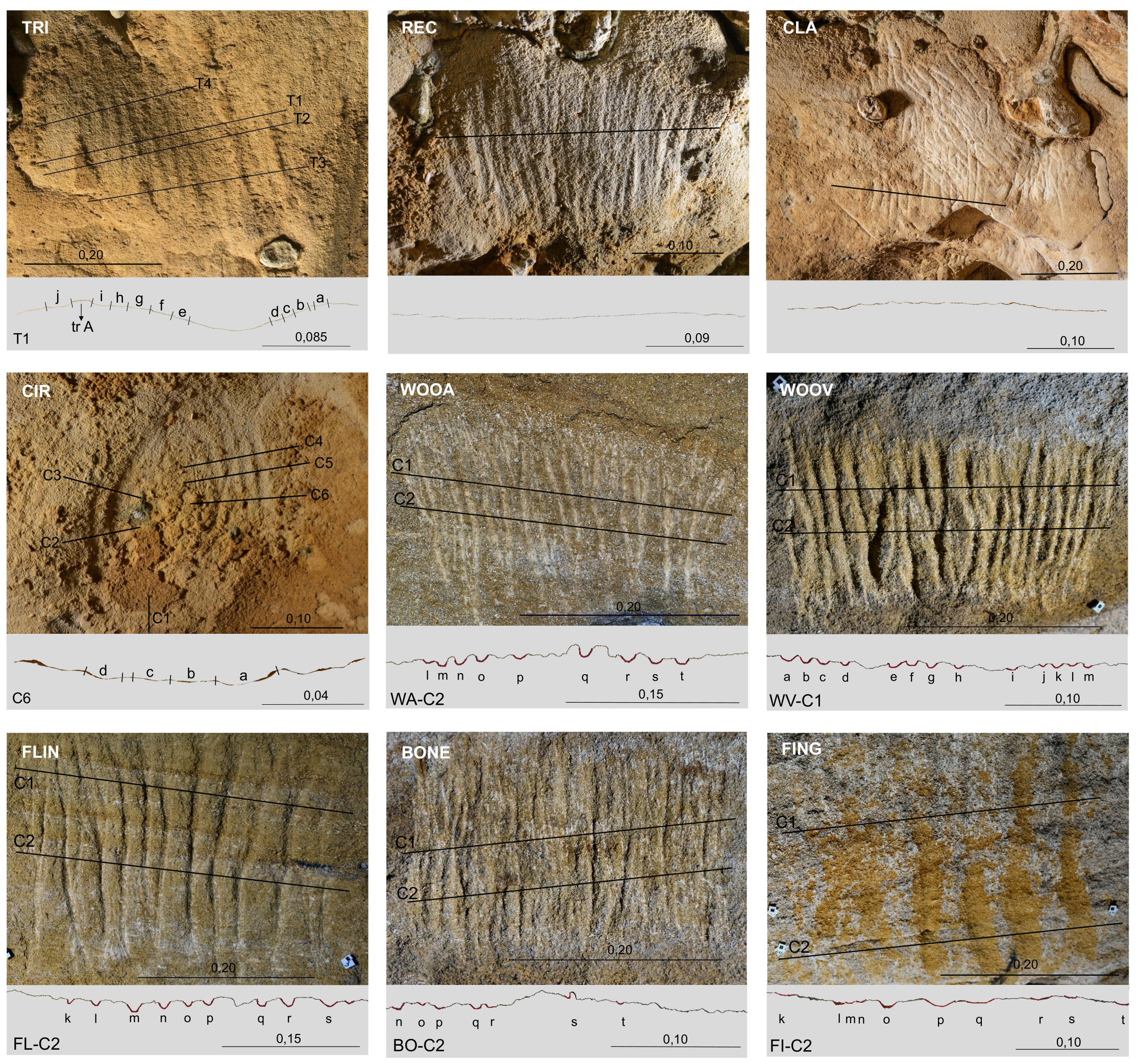

Supplement: S7 Fig — The first four images show the Triangular Panel (TRI), the Rectangular Panel (REC), the bear scratched area (CLA) and finally the Circular Panel (CIR); at the bottom of each picture, the cross-sections made with CloudCompare on the photogrammetries. Four cross-sections were made on the Triangular Panel, T1 to T4 and six on the circular, C1 to C6. Only T1 and C6 are presented below each panel. On these two sections the limits and names of the different plots are indicated, their measurements can be found in Table 2 (T1a corresponds to section a of cut 1 of the Triangular). The cross-sections on the Rectangular Panel have too little relief to allow measurements. The measurements of the width and the angle of incision of the line were carried out using the CloudCompare application and the depth was calculated using a simple mathematical formula: depth = width / 2x tangent (incision angle/2). The same method was used for the scratched space because of the multiple crossings of the traces on the wall. The following five photos show experimental traces made to hypothesise which tool or tools might have been used to make the tracings of the Rectangular Panel. Among the 7 tools that were used for this experiment (S1 Text), we present here only 5 panels, WOOA traced with an antler point, WOOV with a wood point, FLIN with a flint point, BONE with a bone point and FING with a finger positioned flat. In each image the location of the cross-sections that have been made can be seen; only one is presented below the photo. The iron point tracings were less interesting and the finger positioned on edge was used very little as blood was easily lost when the finger passed over the very aggressive surface of the wall. On these 5 panels, all measurements were made directly on the sections that appear on the sections made. All the scales are in meter. (TIF) [file pone.0286568.s012.tif]

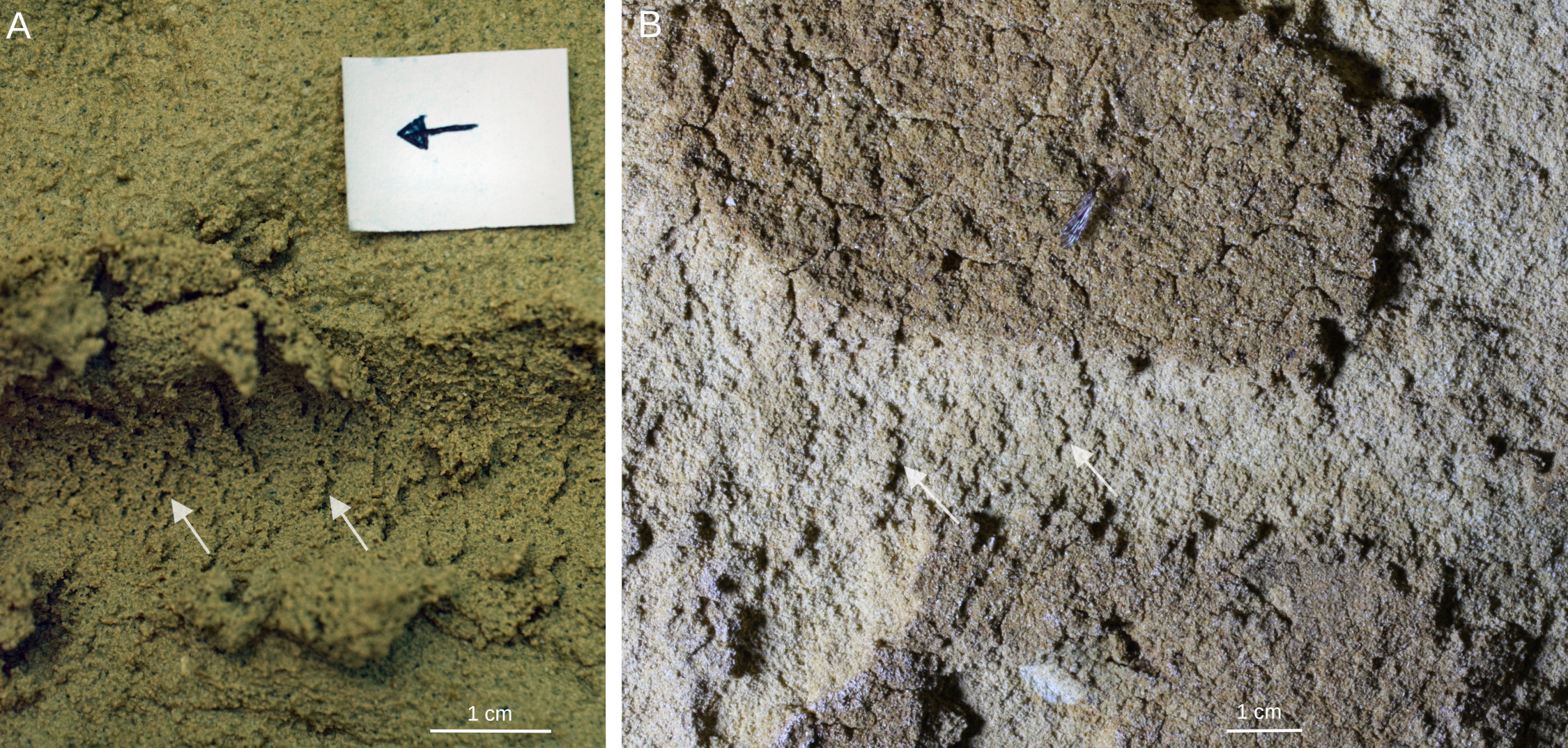

Supplement: S8 Fig — A. Experiment: some crushed is prepared tuff, then placed in a small flat container, moistened a bit, beat and grooved with a finger on its surface. Result: on the bottom of the trace, some reliefs like scales lifted up in the opposite direction of the finger passage are observable. The black arrow indicates the direction of the trace; white arrows the scales. B. Circular Panel, trace C1a. The scales are visible on the bottom of trace (white arrows) and show the direction of the finger fluting. (TIF) [file pone.0286568.s013.tif]

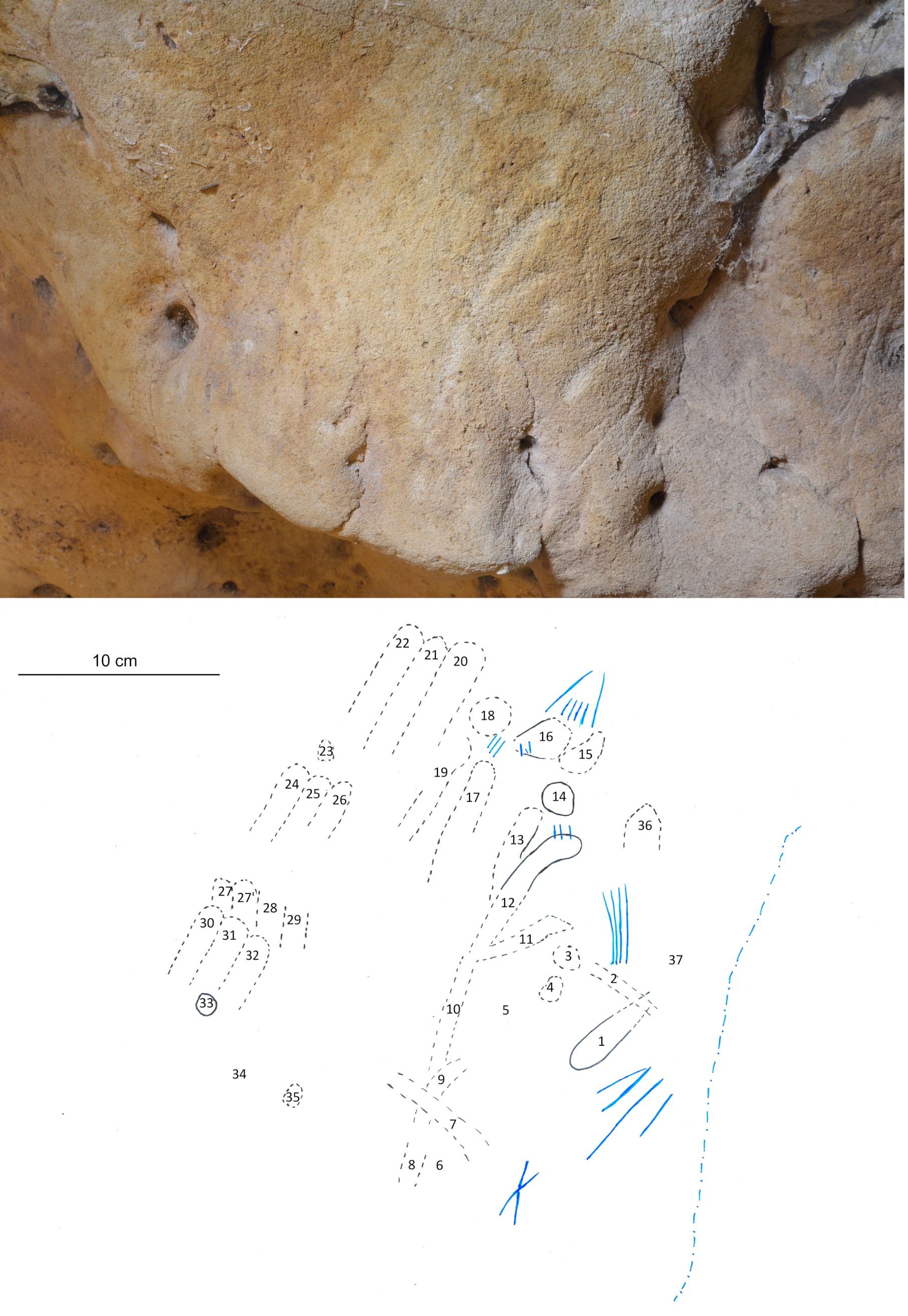

Supplement: S9 Fig — Panel a. (panel in entrance of Pillar Chamber). From top to bottom, photograph and survey of the ancient anthropic traces in black and animal traces in blue, numbering of the anthropic traces. The clear traces are in continuous line, when the trace is deep the line is thicker. Traces that are more difficult to read are dashed. (TIF) [file pone.0286568.s014.tif]

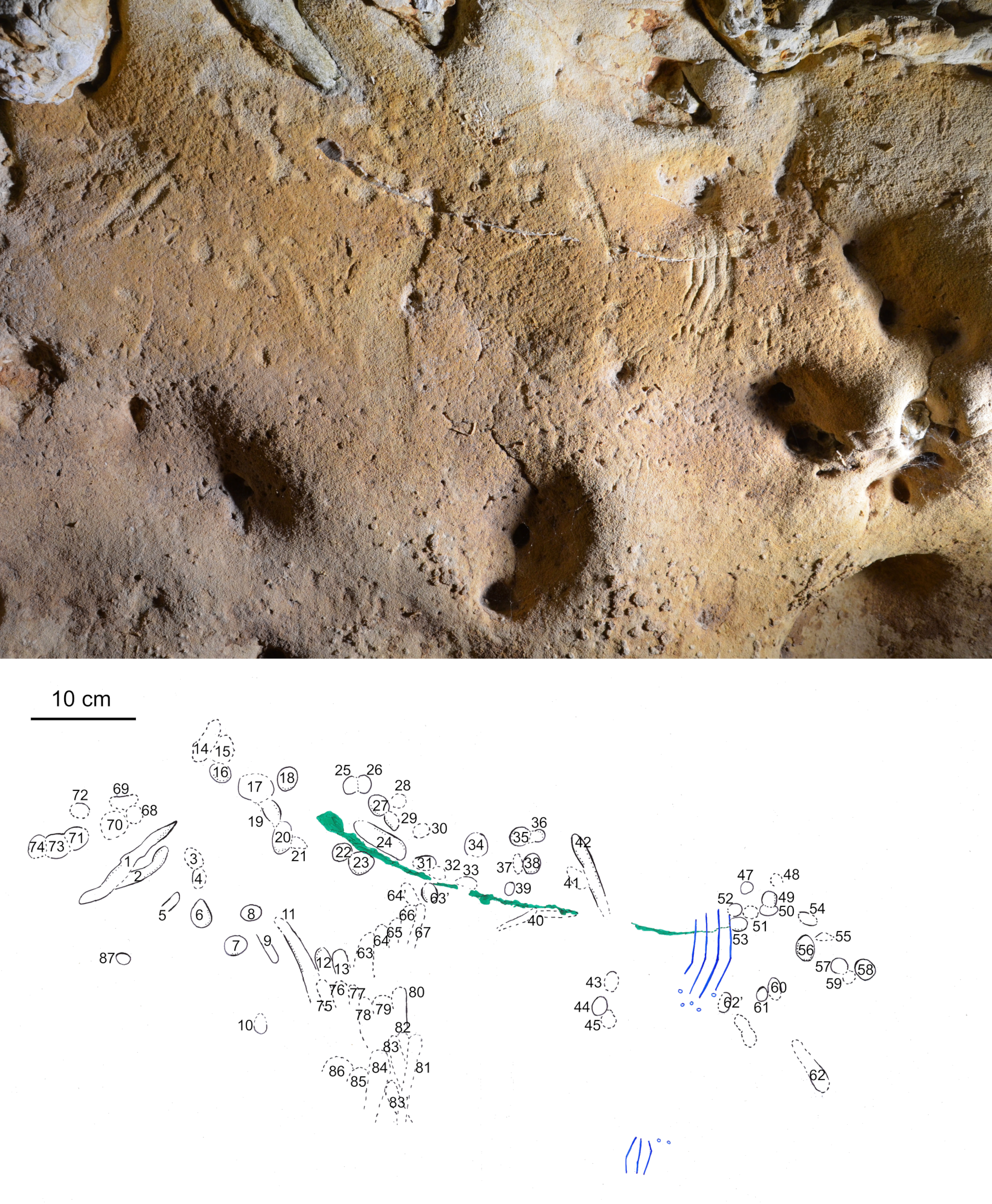

Supplement: S10 Fig — Panel b. (panel of the fossil). From top to bottom, photograph and survey of ancient anthropic traces in black, animal traces in blue, surface of the fossil section in green, numbering of the traces. The clear traces are in continuous line, when the trace is deep the line is thicker. Traces that are more difficult to read are dashed. (TIF) [file pone.0286568.s015.tif]

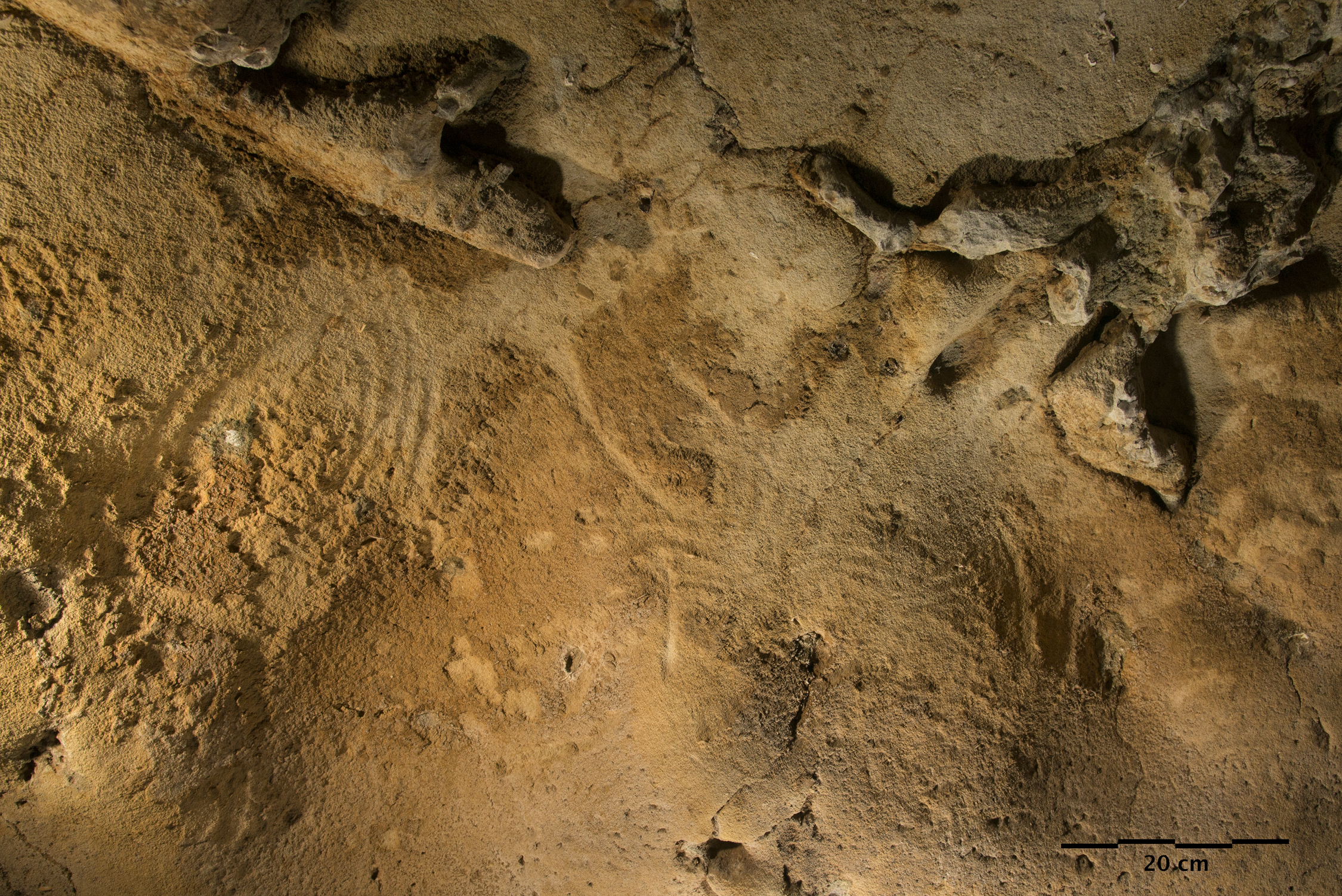

Supplement: S11 Fig — Photograph of the Undulated and Circular Panels (panels d and e). The close proximity of these two panels suggests a connection between them. We do not rule out possible contemporaneity between them. Photo E. Lesvignes. (TIF) [file pone.0286568.s016.tif]

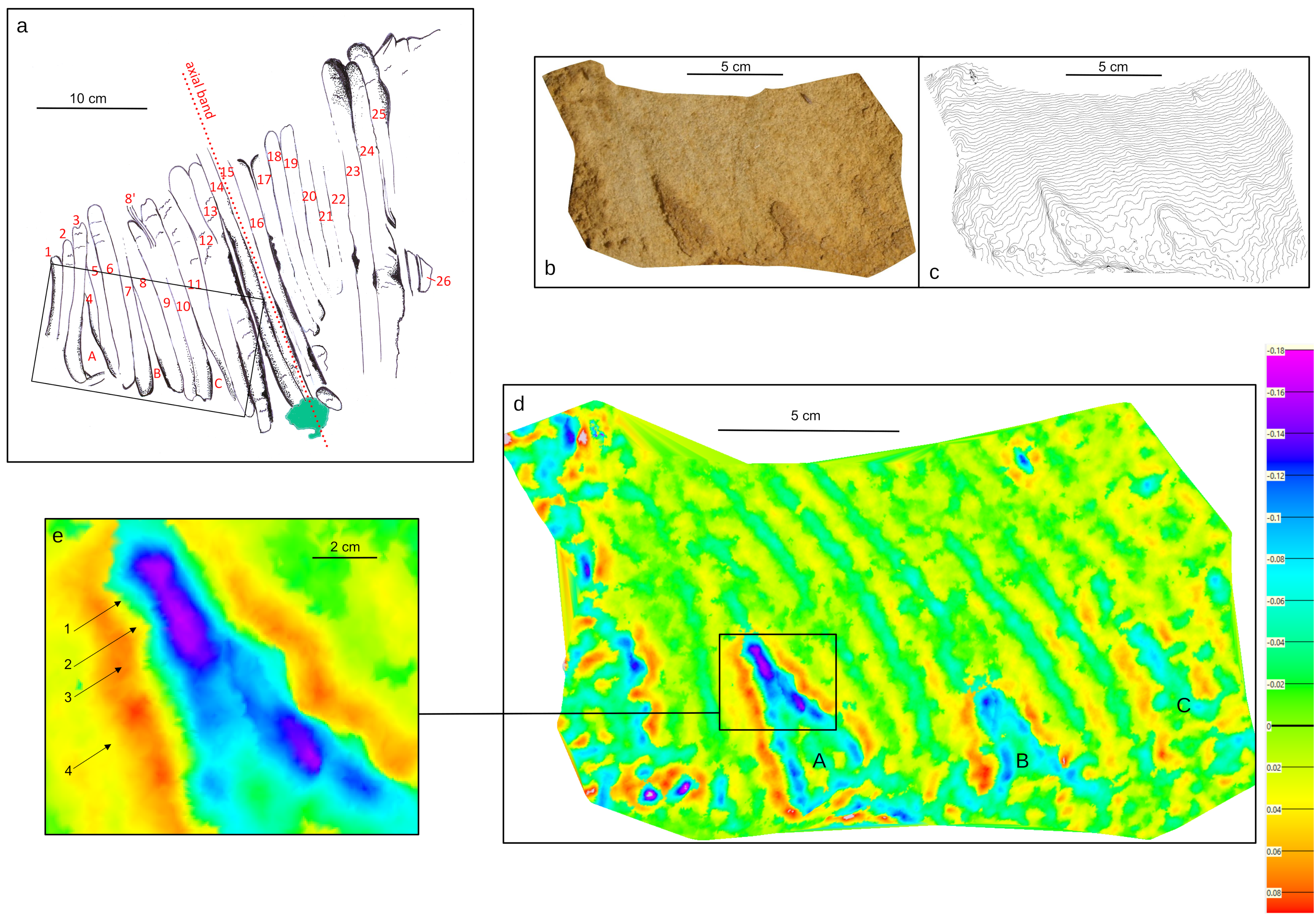

Supplement: S12 Fig — a). Survey of the finger flutings of the totality of the panel. It permit to situate the left part of the panel which has been studied particularly and specially the three preserved triangles A, B and C. b). Orthophoto from the photogrammetry of the left part of the panel. The two triangles A and B are clearly visible, the triangle C with some difficulty due to its alteration. c). The same surface with its contour lines which give the surface relief. The lines are equidistant sections (1mm) parallel to the average plane of the panel, not horizontal. d). A coloured model representing the microrelief of the panel. Red indicates concave surface (relative to the observer’s axis of vision), blue indicates convex surface. Thick line at 0 of the scale indicates flatness of the surface. The units of bending intensity are given in colour range from -0.18 (convex) to +0.08 (concave) for curvature, i.e., from 8 mm to 10 mm for radii of curve. The colour range on the right shows the range and gradation of the panel’s colouration: red and yellow are the concave surfaces (for the observer), green and blue the convex surfaces and, at the limit of yellow and green, the areas without curvature. e). Detail of the groove along the left side of the triangle A. Arrow 1 shows the beginning of the strong slope, Arrow 2 shows a narrow band corresponding to the part of the groove on the side of the triangle. Arrow 3 shows a red band corresponding to the deep part of the groove, Arrow 4 shows a wide yellow stripe corresponding to the other side of the groove with a very gentle slope. (Y. Egels, see S4 Text). (TIF) [file pone.0286568.s017.tif]

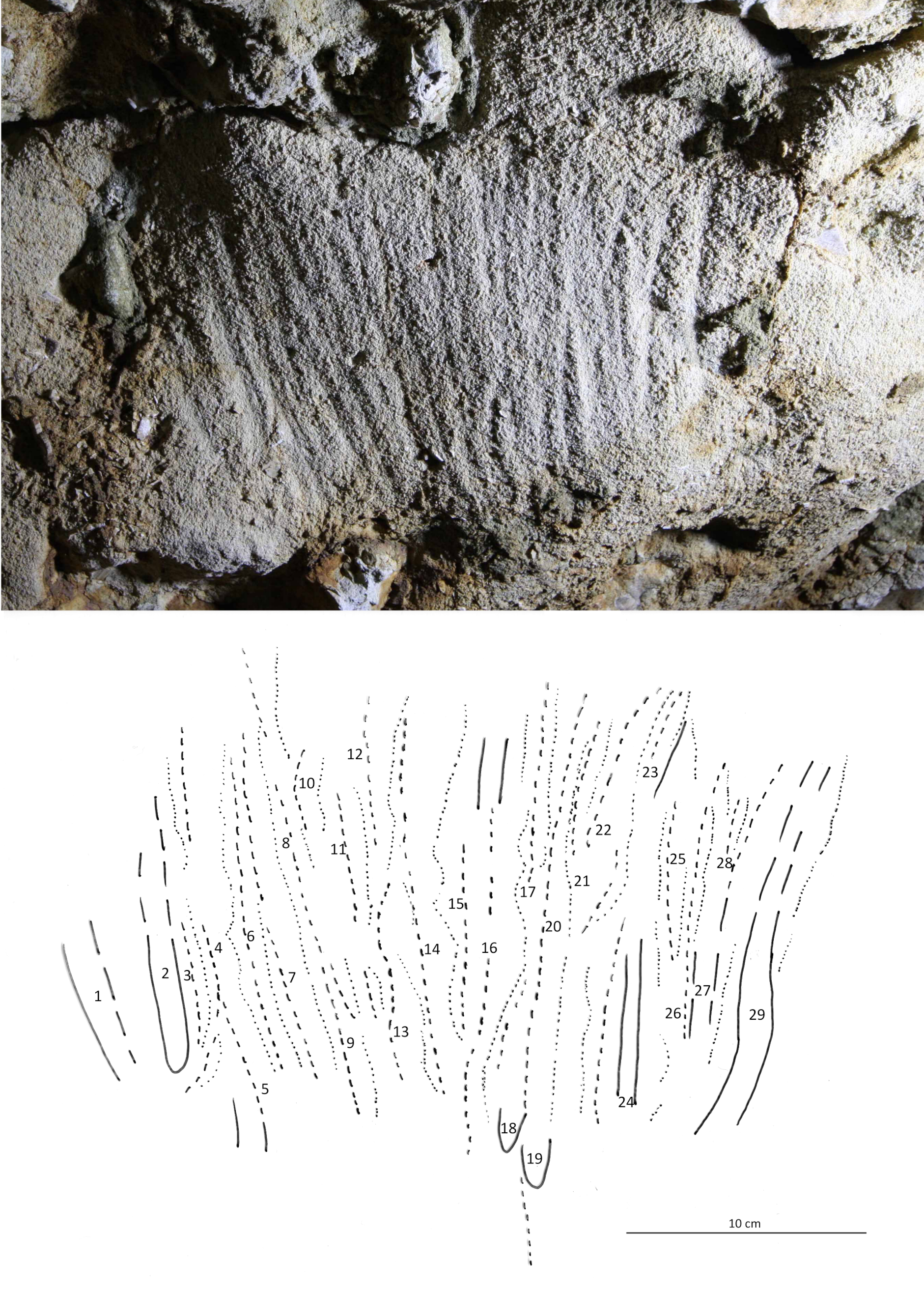

Supplement: S13 Fig — The Rectangular Panel (panel g). Top, photograph of the Rectangular Panel in oblique light from the right. Bottom, sketch of the survey of the ancient anthropic traces of the panel and numbering. The continuous lines depict finger traces, the long dashed lines depict finger traces that are difficult to recognise. The short dashed lines are the lines of the pointed base of the V-section of most traces that are not made with the flat finger. Dotted lines are the ridge lines between two parallel V-section traces (survey S. Audouy). (TIF) [file pone.0286568.s018.tif]

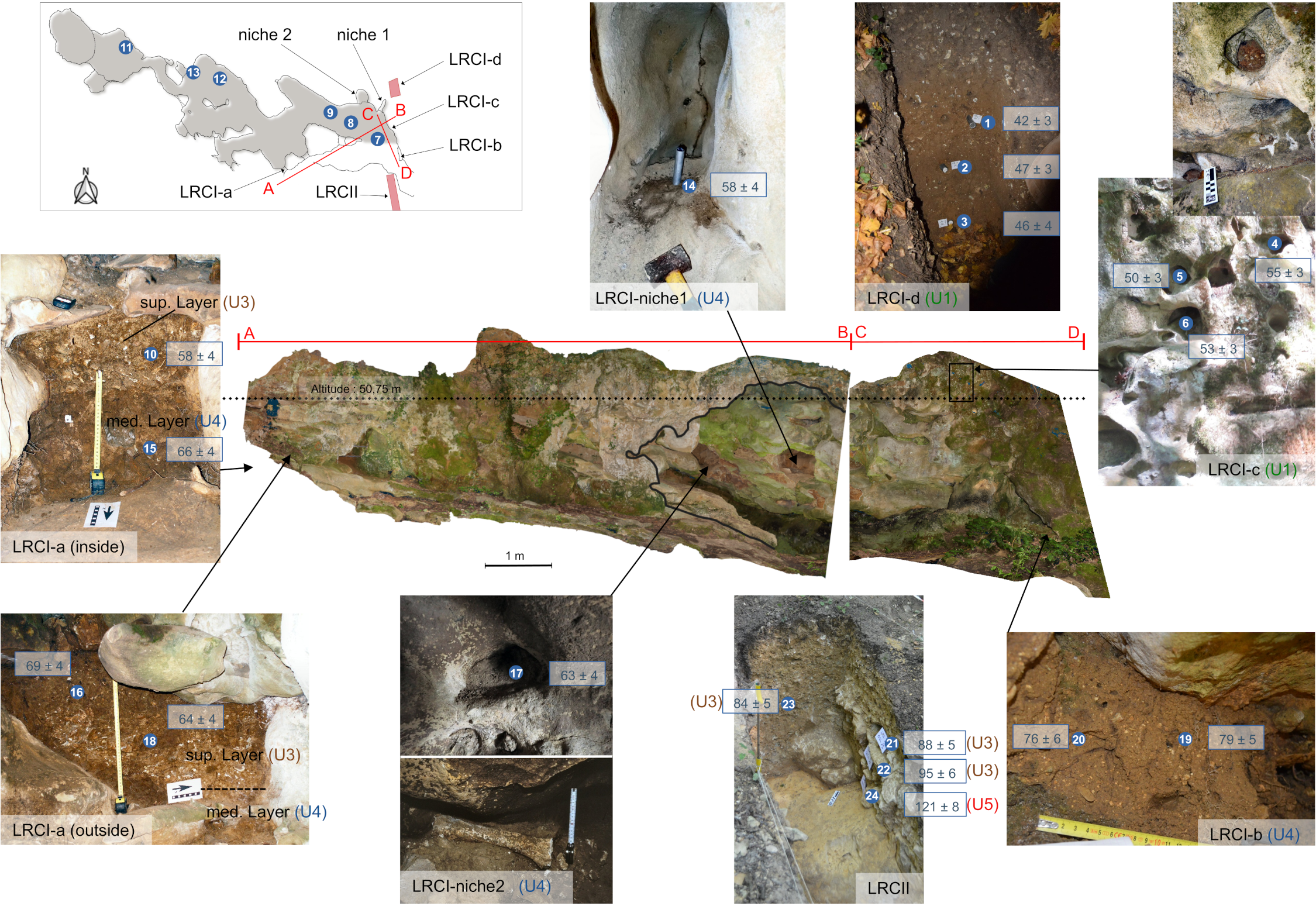

Supplement: S14 Fig — Same figure than Fig 5 in main text integrating numbering of the samples dated by OSL and results of the datings (Table 5). (TIF) [file pone.0286568.s019.tif]

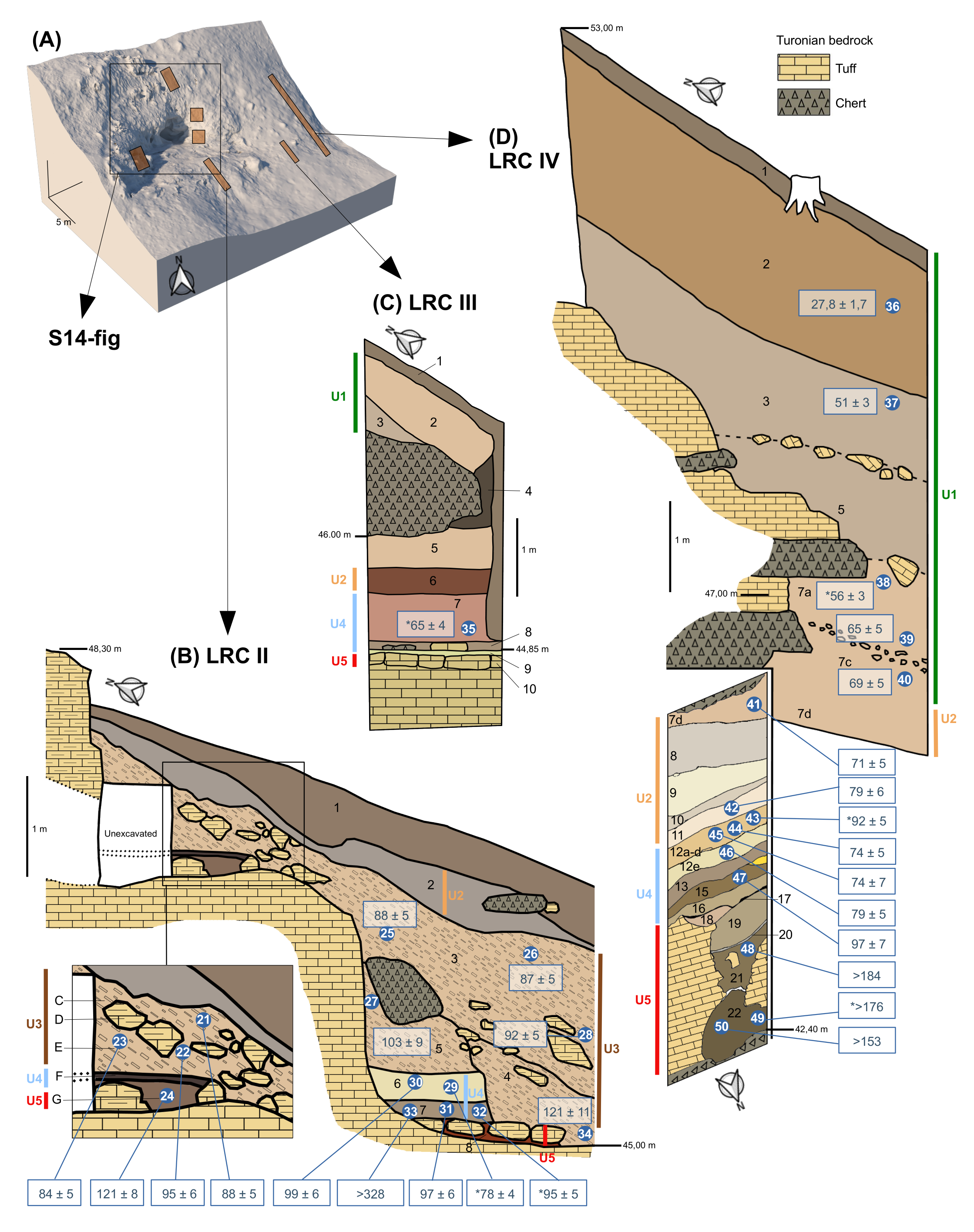

Supplement: S15 Fig — Same figure than Fig 5 in main text integrating numbering of the samples dated by OSL and results of the datings (Table 5). (TIF) [file pone.0286568.s020.tif]
